# Supplementary material for: A selenium-catalysed para-amination of phenols
Source: Nat Commun. 2018 Oct 16;9:4293. doi: 10.1038/s41467-018-06763-4 (PMC6191425; doi:10.1038/s41467-018-06763-4)
Supplement: Supplementary file 1 — Supplementary Information [file 41467_2018_6763_MOESM1_ESM.pdf]

A selenium-catalysed *para*-amination of phenols

Yan *et al*.

Supplementary Methods

General considerations

Commercially available chemicals were obtained from Adamas, Energy, TCI and Bide and used as received unless otherwise stated. *N*-phenylselanylphthalimide (**C1)** was synthesized according to a previous reported literature[1].

Reactions were monitored with analytical thin-layer chromatography (TLC) on silica. ^{1}H NMR and ^{13}C NMR data were recorded on Bruker nuclear resonance (300 MHz, 400 MHz and 500MHz) spectrometers unless otherwise specified, respectively. Chemical shifts (δ) are given in ppm relative to TMS. The residual solvent signals were used as references and the chemical shifts converted to the TMS scale ($CDCl_3$: δ_H=7.26 ppm, δ_C=77.16 ppm; CD_2Cl_2: δ_H=5.32 ppm, δ_C=54.00 ppm; DMSO-d_6: δ_H=2.50 ppm, δ_C=39.52 ppm; MeOD-d_4: δ_H=3.31 ppm, δ_C=49.00 ppm; Acetone-d_6: δ_H=2.05 ppm, δ_C=29.84 ppm, 206.26 ppm). HRMS (ESI) analysis was performed by The Analytical Instrumentation Center at Peking University, Shenzhen Graduate School and (HRMS) data were reported with ion mass/charge (m/z) ratios as values in atomic mass units. Fluorescence spectra were measured on a Shimadzu RF-5301PC spectrometer with a slit width of 3 nm for excitation and 3 nm for emission.

Method A to prepare 1:

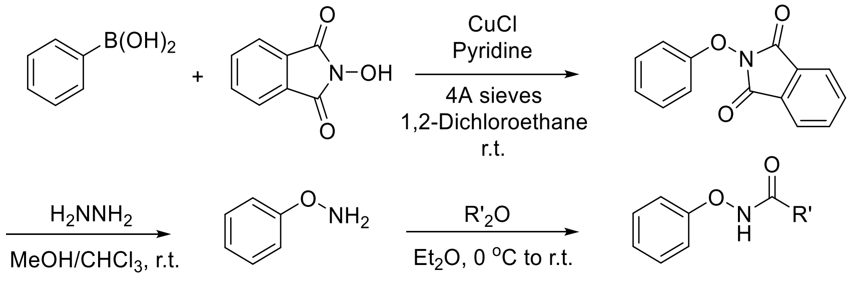

1

Following a literature report[2], in a 50mL round-bottom flask, *N*-hydroxyphthalimide (1.63 g, 10 mmol), cooper (I) chloride (0.99 g, 10 mmol), freshly activated 4 Å molecular sieves (2.5 g), and phenylboronic acid (2.44 g, 20 mmol) were combined in 1,2-dichloroethane (0.2 M). The pyridine (0.8 mL, 11 mmol) was then added to the suspension. The reaction mixture was open to the atmosphere and stirred at room temperature over 24-48 h. Upon completion, silica gel was added to the flask and the solvent was removed under vacuum. The desired *N*-aryloxyphthalimide were obtained by flash column chromatography on silica gel.

Hydrazine monohydrate (1.5 mL, 30 mmol) was added to the solution of *N*-aryloxyphthalimide (1.68 g, 7 mmol) in 10% MeOH in $CHCl_3$ (0.1 M). The reaction was allowed to stir at room temperature over 12 h. Upon completion, the reaction mixture was filtered off and washed with CH_2Cl_2.The filtrate was concentrated under reduced pressure, and purified by flash silica gel column chromatography to give the corresponding *N*-aryloxyamine.

In a 20 mL round-bottom flask, *N*-aryloxyamine (0.6 g, 6 mmol) was dissolved in ether (0.2 M). The flask was cooled in an ice bath, to which corresponding anhydride (1.3 mL, 12 mmol) was slowly added. The ice bath was allowed to warm to room

temperature and the mixture was stirred for 3 h at room temperature. The reaction mixture was concentrated under reduced pressure and purified by flash silica gel column chromatography to give *N*-phenoxyacetamide (0.85 g, 56%).

1w was synthesized according to a literature reported[3].

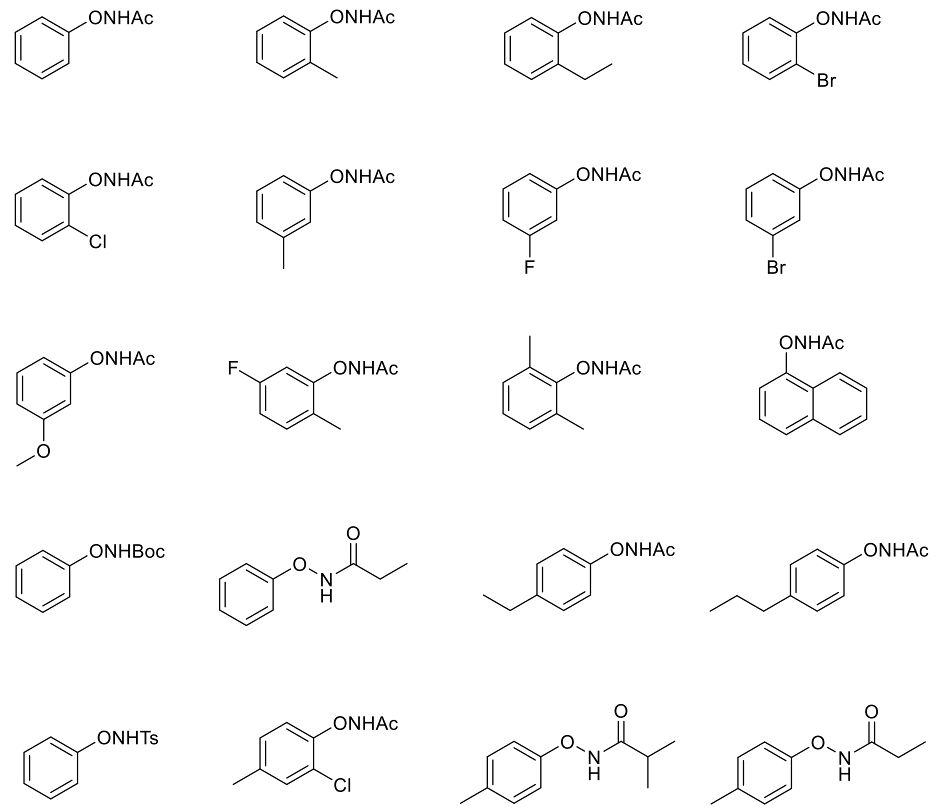

Above starting materials were synthesized according to Method A.

Method B to prepare 1:

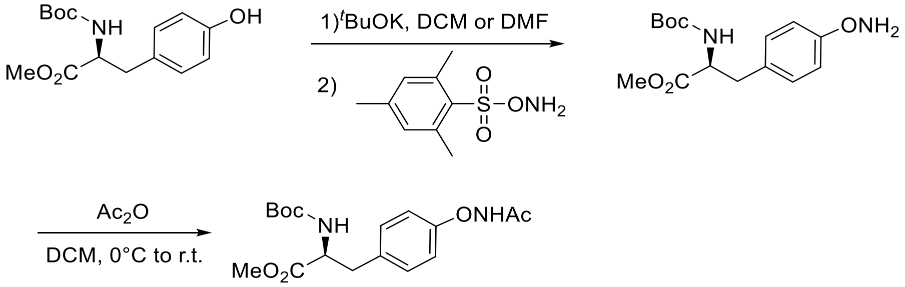

Following literature reports[4], L-tyrosine (2.95 g, 10 mmol) was dissolved in 10 mL of methanol, and then potassium *tert*-butoxide (1.12 g, 10 mmol) was added. The mixture was allowed to stir for 0.5 h under N_2 atmosphere. The methanol was removed, and the residue was taken up in 2 mL of DCM. Then the freshly prepared *O*-mesitylsulfonylhydroxyl-amine (2.15 g, 10 mmol) was added under ice cooling. The mixture was allowed to stir for 1 h, dichloromethane was then removed under reduce pressure to afford the corresponding *N*-aryloxyamine.

In a 20 mL round-bottom flask, *N*-aryloxyamine (1.5 g, 5 mmol) was dissolved in

ether (0.2 M).The flask was cooled in an ice bath, to which acetic anhydride (1.1 mL, 10 mmol) was slowly added. The ice bath was allowed to warm to room temperature and the mixture was stirred for 3 h at room temperature. The reaction mixture was concentrated under reduced pressure and purified by flash silica gel column chromatography to give the corresponding *N*-phenoxyacetamide (1.4 g, 40% overall yield).

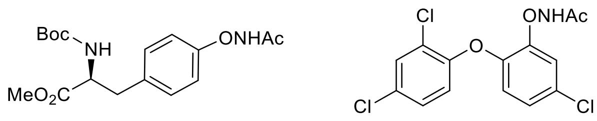

Above starting materials were synthesized according to Method B.

Method C to prepare 1:

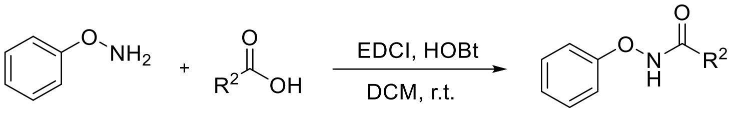

1

To a solution of *N*-aryloxyamine (3.0 mmol) and corresponding acid (3.0 mmol) in CH_2Cl_2 (12.0 ml) at 0℃ were added HOBt (3.3 mmol) and EDCI (3.3 mmol). The reaction mixture was stirred at room temperature for 10 h, then washed with 5% aqueous HCl (3×15 ml), 5% aqueous $NaHCO_3$ (20.0 ml), H_2O (20.0 ml), and brine (20.0 ml), and dried (Na_2SO_4). Purification by flash chromatography afforded the corresponding *N*-phenoxyamides.

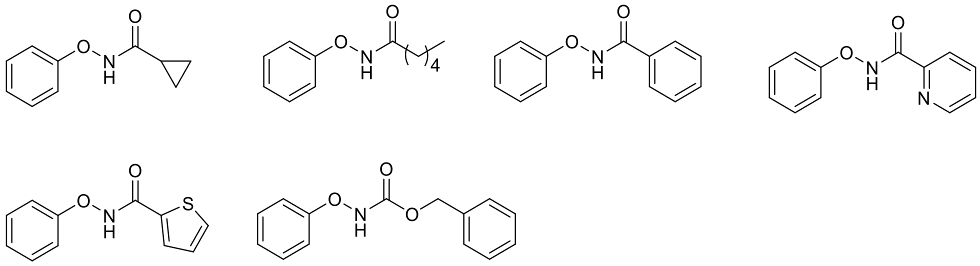

Above starting materials were synthesized according to Method C.

Method to prepare substrate *d*$_8$-1a:

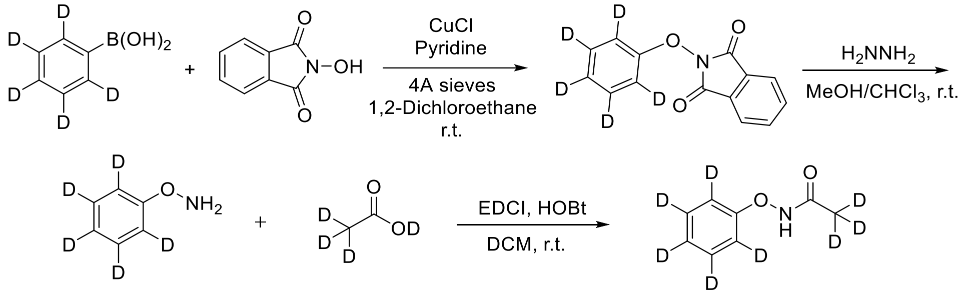

d$_8$-1a

d$_8$-**1a** was synthesized using the method combined Method A and Method C.

Method to prepare substrates 4:

Following a literature report[5], sulfuryl chloride (1.35g, 10 mmol; ca. 5 M in CH_2Cl_2)

was added dropwise via a dropping funnel to a solution of thiophenol (1.1g, 10 mmol; ca. 1 M in CH_2Cl_2) and Et_3N (0.14 mL, 1 mmol) at 0 ℃. After stirring for 15 min, the mixture was warmed to r.t. for 30 min and then cooled to 0 ℃. The resulting solution was transferred dropwise via cannula to a solution of phthalimide (1.36g, 10 mmol; ca. 1 M in CH_2Cl_2) and Et_3N (1.5 mL. 11 mmol) at 0 ℃ and the mixture was then warmed to r.t. over 1 h. The solution was diluted with H_2O, extracted with CH_2Cl_2 (3×) before being dried over Na_2SO_4, and then concentrated to give crude product that was purified using recrystallization. For samples with appreciable amounts of phthalimide present, the crude was dissolved with CH_2Cl_2, diluted with 1.0 M NaOH, extracted with CH_2Cl_2 (3×) before being dried over Na_2SO_4, then concentrated before being purified by recrystallization.

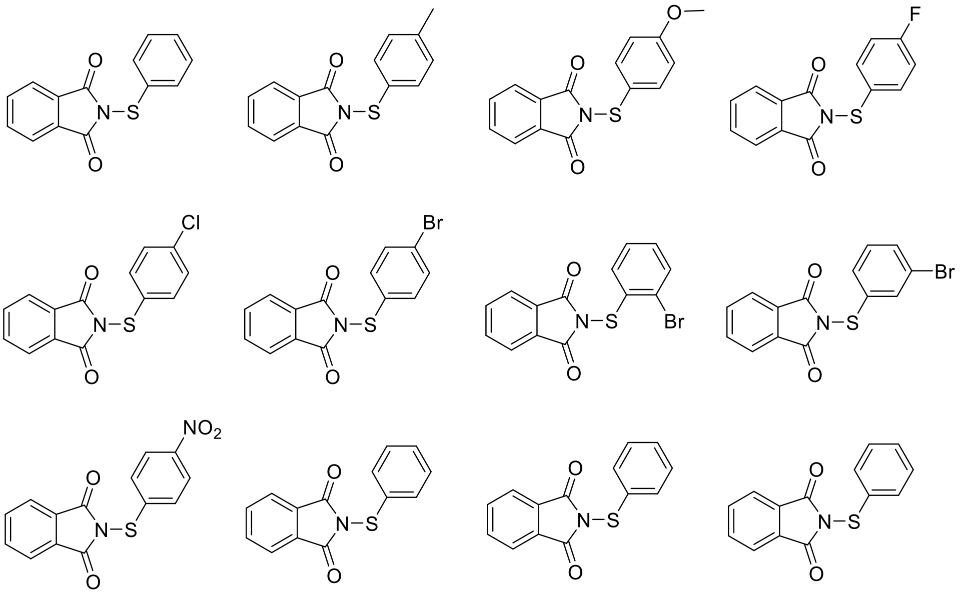

Theoretical studies

All electronic structure calculations were performed with the Gaussion 09 package[6]. All of the geometries were optimized by the B3LYP functional[7] augmented with Grimmes D3 dispersion correction (B3LYP-D3)[8]. Two kinds of basis sets were used. The smaller basis set (BS1) was used for geometry optimizations and vibrational frequency calculations. In BS1, the effective core potential LANL2DZdp[9] was used for Br and Se, and the 6-31G (d, p) basis set was used for all the other atoms. Single-point energies of various stationary points in the solvent were calculated by employing a larger basis set aug-cc-PVTZ-pp[10] for Br and Se atoms and 6-311++G(2d,2p) basis set for all other atoms. Geometry optimizations for the type I and type II reaction are conducted without any constraint in 2,2,2-trifluoroethanol (TFE) and 1,4-dioxane, respectively, using the polarizable continuum model (PCM)[11]. Harmonic vibrational frequency calculations show that the stationary points located were either minima or transition state (single imaginary frequency).

All free energy profiles are reported at 298.15 K and 1.00 bar. Activation free energy

barriers here are defined as the free energy difference between the transition state and the lowest-energy stationary point before it in the reaction pathways.

Characterization data for the new compounds

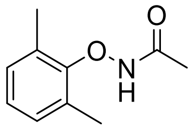

***N*-(2,6-dimethylphenoxy)acetamide, 1b, white solid, 0.84 g, 4.7 mmol, yield: 47%**
^{1}H NMR (400 MHz, DMSO): δ 11.33 (s, 1H), 7.05–6.92 (m, 3H), 2.30 (s, 6H), 1.79 (s, 3H). **13C NMR (101 MHz, DMSO): δ** 166.98, 155.52, 130.79, 129.29, 125.45, 19.92, 16.96. HRMS (ESI) calculated for $C_{10}H_{13}NO_2Na$ $[M+Na]^+$: 202.0838; Found: 202.0844.

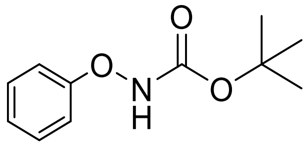

***tert*-butyl phenoxycarbamate, 1c, brown solid, 1.36 g, 6.5 mmol, yield: 65%**
^{1}H NMR (400 MHz, $CDCl_3$): δ 7.58 (s, 1H), 7.32 (t, *J* = 8.0 Hz, 2H), 7.15–7.09 (m, 2H), 7.04 (t, *J* = 7.3 Hz, 1H), 1.52 (s, 9H). **^{13}C NMR (101 MHz, $CDCl_3$):** δ 159.98, 156.52, 129.35, 122.64, 113.28, 82.75, 28.14. HRMS (ESI) calculated for $C_{11}H_{15}NO_3Na$ $[M+Na]^+$: 232.0944; Found: 232.0946.

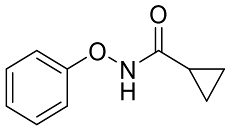

***N*-phenoxycyclopropanecarboxamide, 1d, white solid, 1.3 g, 7.4 mmol, yield: 74%**
^{1}H NMR (500 MHz, DMSO): δ 11.86 (s, 1H), 7.32 (m, 2H), 7.00 (m, 3H), 1.59 (s, 1H), 0.85–0.72 (m, 4H). **^{13}C NMR (126 MHz, DMSO)**: δ 171.73, 160.13, 129.89, 122.67, 113.30, 11.50, 6.91. **HRMS (ESI)** calculated for $C_{10}H_{11}NO_2Na$ $[M+Na]^+$: 200.0682; Found: 200.0685.

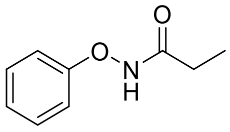

***N*-phenoxypropionamide, 1e, white solid, 0.74 g, 4.5 mmol, yield: 45%**
^{1}H NMR (400 MHz, DMSO): δ 11.67 (s, 1H), 7.32 (m, 2H), 7.00 (d, *J* = 7.3 Hz, 3H), 2.19 (q, *J* = 4.0 Hz, 2H), 1.09 (t, *J* = 7.1 Hz, 3H). **^{13}C NMR (126 MHz, DMSO)**: δ 171.42, 160.04, 129.84, 122.62, 113.25, 25.86, 9.90. **HRMS (ESI)** calculated for $C_9H_{11}NO_2Na$ $[M+Na]^+$: 188.0682; Found: 188.0685.

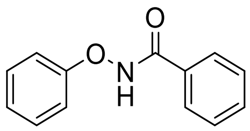

***N*-phenoxybenzamide, 1f, white solid, 1.02 g, 4.8 mmol, yield: 48%**

^{1}H NMR (300 MHz, DMSO-d$_6$): δ 12.49 (s, 1H), 7.94–7.81 (m, 2H), 7.62 (m, 1H), 7.53 (t, *J* = 7.3 Hz, 2H), 7.41–7.28 (m, 2H), 7.06 (dd, *J* = 16.8, 7.9 Hz, 3H). **^{13}C NMR (75 MHz, DMSO-d$_6$):** δ 160.07, 132.57, 131.94, 130.00, 129.13, 127.76, 122.83, 113.45. **HRMS (ESI)** calculated for $C_{13}H_{12}NO_2$ [M+H]$^+$: 214.0863; Found: 214.0863.

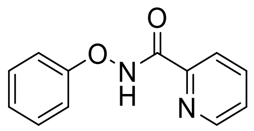

***N*-phenoxypicolinamide, 1g, white solid, 1.2 g, 5.7 mmol, yield: 57%**

^{1}H NMR (400 MHz, CDCl$_3$): δ 10.94 (s, 1H), 8.6–8.51 (m, 1H), 8.20 (d, *J* = 7.8 Hz, 1H), 7.88 (m, 1H), 7.49 (m, 1H), 7.37–7.24 (m, 2H), 7.16 (d, *J* = 8.0 Hz, 2H), 7.05 (dd, *J* = 10.5, 4.1 Hz, 1H). **^{13}C NMR (101 MHz, CDCl$_3$):** δ 162.85, 159.57, 148.50, 137.69, 129.50, 127.18, 123.11, 122.84, 113.43. **HRMS (ESI)** calculated for $C_{12}H_{11}N_2O_2$ [M+H]$^+$: 215.0755; Found: 215.0760.

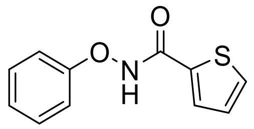

***N*-phenoxythiophene-2-carboxamide, 1h, white solid, 1.16 g, 5.3 mmol, yield: 53%**

^{1}H NMR (500 MHz, DMSO): δ 12.59 (s, 1H), 7.86 (dd, *J* = 6.5, 4.6 Hz, 2H), 7.34 (t, *J* = 7.8 Hz, 2H), 7.21 (t, *J* = 4.2 Hz, 1H), 7.09 (d, *J* = 8.0 Hz, 2H), 7.03 (t, *J* = 7.2 Hz, 1H). **^{13}C NMR (126 MHz, DMSO):** δ 160.69, 160.03, 135.61, 132.35, 129.99, 128.60, 122.98, 113.45. **HRMS (ESI)** calculated for **HRMS (ESI)** calculated for $C_{11}H_9NO_2SNa$ [M+Na]$^+$: 242.0246; Found: 242.0251.

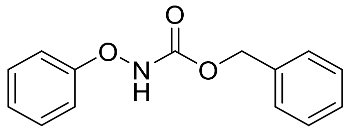

***N*-phenoxy-2-phenylacetamide, 1i, white solid, 1.26 g, 5.2 mmol, yield: 52%**

^{1}H NMR (400 MHz, CDCl$_3$): δ 7.78 (s, 1H), 7.38 (s, 4H), 7.33 (dd, *J* = 8.5, 7.5 Hz, 2H), 7.12 (d, *J* = 8.0 Hz, 2H), 7.07 (m, 1H), 5.26 (s, 2H). **^{13}C NMR (101 MHz, CDCl$_3$):** δ 159.75, 157.36, 135.24, 129.44, 128.65, 128.58, 128.34, 122.95, 113.31, 68.11. **HRMS (ESI)** calculated for $C_{14}H_{13}NO_3Na$ [M+Na]$^+$: 266.0788; Found: 266.0789.

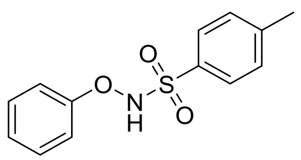

4-methyl-*N*-phenoxybenzenesulfonamide, 1j, white solid, 1.03 g, 3.9 mmol, yield: 39%

^{1}H NMR (400 MHz, CDCl$_3$): δ 7.88 (d, *J* = 8.2 Hz, 2H), 7.75 (s, 1H), 7.35 (d, *J* = 8.1 Hz, 2H), 7.29 (t, *J* = 7.9 Hz, 2H), 7.15 (d, *J* = 8.3 Hz, 2H), 7.05 (s, 1H), 2.46 (s, 3H). **^{13}C NMR (101 MHz, CDCl$_3$):** δ 159.36, 145.39, 133.11, 129.90, 129.36, 128.80, 123.27, 114.32, 21.74. **HRMS (ESI)** calculated for $C_{13}H_{13}NO_3SNa$ [M+Na]$^+$: 286.0508;

Found: 286.0510.

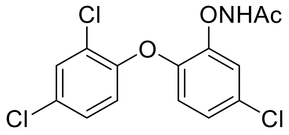

***N*-(5-chloro-2-(2,4-dichlorophenoxy)phenoxy)acetamide, 1k, white solid, 1.79 g, 5.2 mmol, yield: 52%**

^{1}H NMR (500 MHz, DMSO): δ 11.81 (s, 1H), 7.71 (d, *J* = 2.5 Hz, 1H), 7.35 (dd, *J* = 8.9, 2.6 Hz, 1H), 7.25 (s, 1H), 7.10 (s, 2H), 6.88 (d, *J* = 8.8 Hz, 1H), 1.91 (s, 3H). **^{13}C NMR (126 MHz, DMSO):** δ 151.90, 151.69, 130.29, 129.98, 128.94, 127.87, 124.02, 123.26, 122.81, 119.40, 114.76, 40.52, 40.35, 40.18, 39.52, 19.77. **HRMS (ESI)** calculated for $C_{14}H_{10}Cl_3NO_3Na$ $[M+Na]^+$ 367.9618; Found: 367.9614.

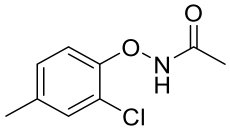

***N*-(2-chloro-4-methylphenoxy)acetamide, 1l, white solid, 0.86 g, 4.3 mmol, yield: 43%**

^{1}H NMR (500 MHz, DMSO): δ 11.79 (s, 1H), 7.24 (s, 1H), 7.06 (m, 2H), 2.23 (s, 3H), 1.93 (s, 3H). **^{13}C NMR (126 MHz, DMSO):** δ 167.96, 152.99, 133.12, 130.60, 128.93, 118.57, 114.10, 20.14, 19.78. **HRMS (ESI)** calculated for $C_9H_{10}ClNO_2Na$ $[M+Na]^+$: 222.0292; Found: 222.0301.

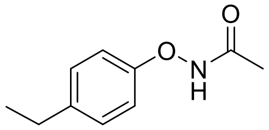

***N*-(4-ethylphenoxy)acetamide, 1m, white solid, 0.81 g, 4.5 mmol, yield: 45%**

^{1}H NMR (300 MHz, DMSO): δ 11.62 (s, 1H), 7.12 (d, *J* = 8.3 Hz, 2H), 6.90 (d, *J* = 8.3 Hz, 2H), 2.53 (q, *J* = 7.6 Hz, 2H), 1.89 (s, 3H), 1.13 (t, *J* = 7.6 Hz, 3H). **^{13}C NMR (300 MHz, DMSO):** δ 167.54, 158.13, 138.04, 128.99, 113.26, 27.78, 19.87, 16.37. **HRMS (ESI)** calculated for $C_{10}H_{13}NO_2Na$ $[M+Na]^+$: 202.0838; Found: 202.0844.

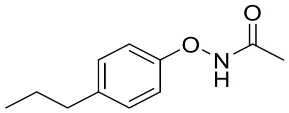

***N*-phenoxypropionamide, 1n, white solid, 0.98 g, 5.1 mmol, yield: 51%**

^{1}H NMR (300 MHz, DMSO): δ 11.61 (s, 1H), 7.10 (d, *J* = 8.0 Hz, 2H), 6.90 (d, *J* = 7.9 Hz, 2H), 2.48 (t, *J* = 7.4 Hz, 2H), 1.89 (s, 3H), 1.54 (q, *J* = 7.4 Hz, 2H), 0.86 (t, *J* = 7.3 Hz, 3H). **^{13}C NMR (101 MHz, DMSO):** δ 167.54, 158.16, 136.36, 129.55, 118.19, 36.83, 24.78, 19.86, 14.00. **HRMS (ESI)** calculated for $C_{11}H_{15}NO_2Na$ $[M+Na]^+$: 216.0995; Found: 216.1008.

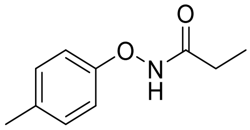

***N*-(p-tolyloxy)propionamide, 1o, white solid, 0.72 g, 4 mmol, yield: 40%**

^{1}H NMR (300 MHz, DMSO): δ 11.61 (s, 1H), 7.10 (d, *J* = 8.0 Hz, 2H), 6.90 (d, *J* = 7.9 Hz, 2H), 2.48 (t, *J* = 7.4 Hz, 2H), 1.89 (s, 3H), 1.54 (q, *J* = 5.6 Hz, 2H), 0.86 (t, *J* = 7.3 Hz, 3H). **^{13}C NMR (75 MHz, DMSO):** δ 167.54, 158.16, 136.36, 129.55, 113.19, 36.83, 24.78, 19.86, 14.00. **HRMS (ESI)** calculated for $C_{10}H_{13}NO_2Na$ $[M+Na]^+$: 202.0838; Found: 202.0837.

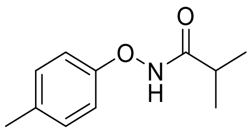

***N*-(p-tolyloxy)isobutyramide, 1p, white solid, 1.12 g, 5.8 mmol, yield: 58%**

^{1}H NMR (300 MHz, DMSO): δ 11.67 (s, 1H), 7.10 (d, *J* = 8.4 Hz, 2H), 6.86 (d, *J* = 8.4 Hz, 2H), 2.42 (m, 1H), 2.23 (s, 3H), 1.08 (d, *J* = 6.8 Hz, 6H). **^{13}C NMR (75 MHz, DMSO):** δ 174.28, 158.10, 131.46, 130.17, 113.16, 31.79, 20.54, 19.69. **HRMS (ESI)** calculated for $C_{11}H_{15}NO_2Na$ $[M+Na]^+$: 216.0995; Found: 216.0978.

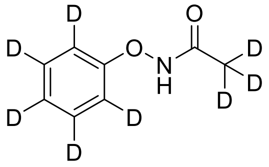

d$_8$-**1a**

***d*$_8$-*N*-phenoxyacetamide, *d*$_8$-1a, white solid, 99 mg, 0.62 mmol, yield: 62%**

^{1}H NMR (400 MHz, DMSO) δ 11.67 (s, 1H). **^{13}C NMR (101 MHz, DMSO) δ** 167.67, 159.92, 129.62, 129.38, 129.14, 122.17, 121.94, 113.18, 112.93, 112.69. **HRMS (ESI)** calculated for $C_8HD_8NO_2Na$ $[M+Na]^+$:182.1028; Found: 182.1032.

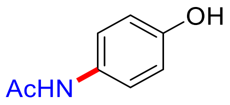

2a

***N*-(4-hydroxyphenyl)acetamide, 2a, white solid, 27.2 mg, 0.18 mmol, yield: 90%**

^{1}H NMR (500 MHz, DMSO): δ 9.63 (s, 1H), 9.11 (s, 1H), 7.33 (d, *J* = 8.9 Hz, 2H), 6.67 (d, *J* = 8.9 Hz, 2H), 1.97 (s, 3H); **^{13}C NMR (126 MHz, DMSO):** δ 167.92, 153.54, 131.45, 121.25, 115.41, 24.15. **HRMS (ESI)** calculated for $C_8H_9NO_2Na$ $[M+Na]^+$: 174.0525; Found: 174.0528.

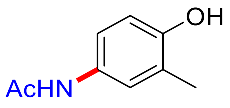

2b

***N*-(4-hydroxy-3-methylphenyl)acetamide, 2b, white solid, 24.1 mg, 0.146 mmol, yield: 73%**

^{1}H NMR (500 MHz, DMSO): δ 9.59 (s, 1H), 9.02 (s, 1H), 7.23 (d, *J* = 2.3 Hz, 1H), 7.15 (dd, *J* = 8.5, 2.5 Hz, 1H), 6.67 (d, *J* = 8.6 Hz, 1H), 2.07 (s, 3H), 1.96 (s, 3H); **^{13}C NMR (126 MHz, DMSO):** δ 167.87, 151.63, 131.29, 124.00, 122.57, 118.51, 114.79,

24.15, 16.54. **HRMS (ESI)** calculated for $C_9H_{11}NO_2Na$ $[M+Na]^+$: 188.0682; Found: 188.0684.

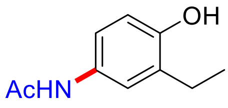

2c

***N*-(3-ethyl-4-hydroxyphenyl)acetamide, 2c, white solid, 29.7 mg, 0.166 mmol, yield: 83%**

^{1}H NMR (500 MHz, DMSO): δ 9.59 (s, 1H), 8.99 (s, 1H), 7.23 (d, *J* = 2.4 Hz, 1H), 7.19 (dd, *J* = 8.5, 2.5 Hz, 1H), 6.67 (d, *J* = 8.5 Hz, 1H), 2.48 (q, *J* = 7.5 Hz, 2H), 1.97 (s, 3H), 1.10 (t, *J* = 7.5 Hz, 3H); **^{13}C NMR (126 MHz, DMSO):** δ 151.19, 131.46, 130.12, 120.92, 118.47, 114.96, 24.15, 23.23, 14.55. **HRMS (ESI)** calculated for $C_{10}H_{13}NO_2Na$ $[M+Na]^+$: 202.0838; Found: 202.0804.

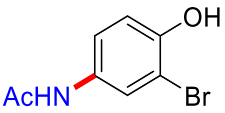

2d

***N*-(3-bromo-4-hydroxyphenyl)acetamide, 2d, white solid, 35.7 mg, 0.156 mmol, yield: 78%**

^{1}H NMR (400 MHz, DMSO): δ 9.98 (s, 1H), 9.87 (s, 1H), 7.83 (d, *J* = 2.3 Hz, 1H), 7.27 (dd, *J* = 8.7, 2.4 Hz, 1H), 6.89 (d, *J* = 8.7 Hz, 1H), 1.99 (s, 3H); **^{13}C NMR (101 MHz, DMSO):** δ 168.33, 150.26, 132.56, 123.92, 120.19, 116.54, 109.00, 24.22. **HRMS (ESI)** calculated for $C_8H_8BrNO_2Na$ $[M+Na]^+$: 251.9631; Found: 251.9627.

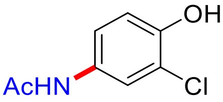

2e

***N*-(3-chloro-4-hydroxyphenyl)acetamide, 2e, white solid, 24.1 mg, 0.13 mmol, yield: 65%**

^{1}H NMR (500 MHz, DMSO): δ 9.85 (s, 1H), 9.32 (s, 1H), 7.28 (d, *J* = 8.7 Hz, 1H), 6.84 (s, 1H), 6.70 (d, *J* = 8.5 Hz, 1H), 2.00 (s, 3H); **^{13}C NMR (126 MHz, DMSO):** δ 169.08, 156.04, 129.00, 128.90, 126.80, 115.97, 114.77, 23.32. **HRMS (ESI)** calculated for $C_8H_8ClNO_2Na$ $[M+Na]^+$: 208.0136; Found: 208.0139.

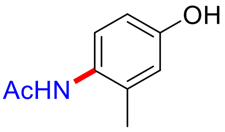

2f

***N*-(4-hydroxy-2-methylphenyl)acetamide, 2f, white solid, 27.1 mg, 0.164 mmol, yield: 82%**

^{1}H NMR (500 MHz, DMSO): δ 9.20 (s, 1H), 9.10 (s, 1H), 7.02 (d, *J* = 8.5 Hz, 1H), 6.59 (d, *J* = 2.6 Hz, 1H), 6.53 (dd, *J* = 8.5, 2.7 Hz, 1H), 2.07 (s, 3H), 1.98 (s, 3H); **^{13}C NMR (126 MHz, DMSO):** δ 168.58, 155.30, 134.34, 128.32, 127.44, 116.94, 112.94,

23.39, 18.36. **HRMS (ESI)** calculated for $C_9H_{11}NO_2Na$ $[M+Na]^+$: 188.0682; Found: 188.0688.

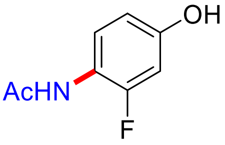

2g

***N*-(2-fluoro-4-hydroxyphenyl)acetamide, 2g, white solid, 28.1 mg, 0.166 mmol, yield: 83%**

^{1}H NMR (500 MHz, DMSO): δ 9.73 (s, 1H), 9.39 (s, 1H), 7.38 (t, *J* = 9.0 Hz, 1H), 6.59 (dd, *J* = 12.3, 2.6 Hz, 1H), 6.54 (dd, *J* = 8.7, 2.3 Hz, 1H), 2.00 (s, 3H); **^{13}C NMR (126 MHz, DMSO):** δ 168.74, 155.91 (d, J = 11.0 Hz), 155.79 (dd, J = 127.7, 116.7 Hz), 126.97 (d, J = 3.4 Hz), 117.50, 111.23, 103.09 (d, J = 22.3 Hz), 23.45; **^{19}F NMR (376 MHz, DMSO):** δ -121.94. **HRMS (ESI)** calculated for $C_8H_8FNO_2Na$ $[M+Na]^+$: 192.0431; Found: 192.0434.

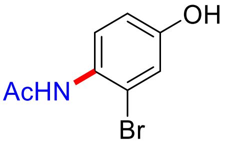

2h

***N*-(2-bromo-4-hydroxyphenyl)acetamide, 2h, white solid, 30.7 mg, 0.134 mmol, yield: 67%**

^{1}H NMR (500 MHz, DMSO): δ 9.82 (s, 1H), 9.29 (s, 1H), 7.21 (d, *J* = 8.7 Hz, 1H), 7.01 (d, *J* = 2.6 Hz, 1H), 6.74 (dd, *J* = 8.7, 2.7 Hz, 1H), 1.99 (s, 3H); **^{13}C NMR (126 MHz, DMSO)** δ 168.87, 156.37, 129.44, 128.22, 120.13, 118.98, 115.31, 23.33. **HRMS (ESI)** calculated for $C_8H_8BrNO_2Na$ $[M+Na]^+$: 251.9631; Found: 251.9632.

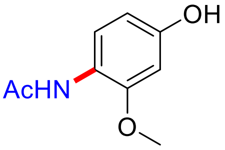

2i

***N*-(4-hydroxy-2-methoxyphenyl)acetamide, 2i, white solid, 26.8 mg, 0.148 mmol, yield: 74%**

^{1}H NMR (500 MHz, DMSO): δ 9.31 (s, 1H), 8.87 (s, 1H), 7.44 (d, *J* = 8.6 Hz, 1H), 6.42 (d, *J* = 2.5 Hz, 1H), 6.28 (dd, *J* = 8.6, 2.5 Hz, 1H), 3.73 (s, 3H), 1.98 (s, 3H); **^{13}C NMR (126 MHz, DMSO):** δ 168.36, 155.38, 152.12, 124.83, 119.51, 106.55, 99.76, 55.81, 23.88. **HRMS (ESI)** calculated for $C_9H_{11}NO_3Na$ $[M+Na]^+$: 204.0631; Found: 204.0638.

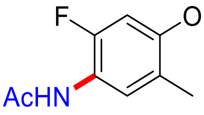

2j

***N*-(2-fluoro-4-hydroxy-5-methylphenyl)acetamide, 2j, white solid, 22.7 mg, 0.124**

mmol, yield: 62%

^{1}H NMR (500 MHz, DMSO): δ 9.68 (s, 1H), 9.36 (s, 1H), 7.25 (d, *J* = 9.1 Hz, 1H), 6.59 (d, *J* = 12.0 Hz, 1H), 2.04 (s, 3H), 1.99 (s, 3H); **^{13}C NMR (126 MHz, DMSO):** δ 168.88, 154.68, 153.44 (d, *J* = 10.1 Hz), 152.75, 127.92 (d, *J* = 2.8 Hz), 119.89 (d, *J* = 2.9 Hz), 116.83 (d, *J* = 13.8 Hz), 102.43, 102.25, 23.39, 15.71. **^{19}F NMR** (376 MHz, DMSO) δ -125.85. **HRMS (ESI)** calculated for $C_9H_{10}FNO_2Na$ $[M+Na]^+$: 206.0588; Found: 206.0583.

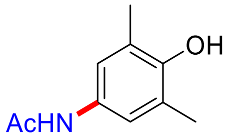

2k

***N*-(4-hydroxy-3,5-dimethylphenyl)acetamide, 2k, white solid, 32.9 mg, 0.184 mmol, yield: 92%**

^{1}H NMR (400 MHz, DMSO): δ 9.55 (s, 1H), 7.95 (s, 1H), 7.10 (s, 2H), 2.11 (s, 6H), 1.96 (s, 3H); **^{13}C NMR (101 MHz, DMSO):** δ 167.92, 149.36, 131.53, 124.74, 119.99, 24.25, 17.26. **HRMS (ESI)** calculated for $C_{10}H_{13}NO_2Na$ $[M+Na]^+$: 202.0838; Found: 202.0847.

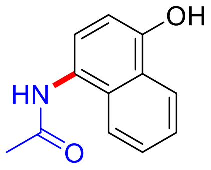

2l

***N*-(4-hydroxy-3,5-dimethylphenyl)acetamide, 2l, white solid, 21.7 mg, 0.108 mmol, yield: 54%**

^{1}H NMR (400 MHz, DMSO): δ 10.16 (s, 1H), 9.67 (s, 1H), 8.23–8.15 (m, 1H), 7.91 (d, *J* = 7.9 Hz, 1H), 7.50 (m, 2H), 7.34 (d, *J* = 8.0 Hz, 1H), 6.87 (d, *J* = 8.0 Hz, 1H), 2.15 (s, 3H); **^{13}C NMR (101 MHz, DMSO):** δ 169.52, 151.72, 130.32, 126.48, 125.42, 125.20, 125.05, 124.19, 123.31, 122.76, 107.76, 23.61. **HRMS (ESI)** calculated for $C_{12}H_{11}NNaO_2$ $[M+Na]^+$: 224.0682; Found: 224.0690.

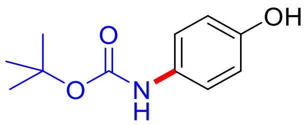

2m

***tert*-butyl (4-hydroxyphenyl)carbamate, 2m, white solid, 31.8 mg, 0.152 mmol, yield: 76%**

^{1}H NMR (500 MHz, DMSO): δ 9.09 (s, 1H), 8.92 (s, 1H), 7.19 (d, *J* = 7.2 Hz, 2H), 6.64 (d, *J* = 8.7 Hz, 2H), 1.43 (s, 9H); **^{13}C NMR (126 MHz, DMSO):** δ 153.49, 152.95, 131.40, 120.58, 115.46, 78.89, 28.59. **HRMS (ESI)** calculated for $C_{11}H_{15}NO_3Na$ $[M+Na]^+$: 232.0944; Found: 232.0943.

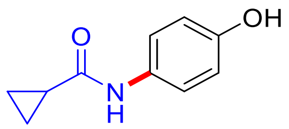

2n

***N*-(4-hydroxyphenyl)cyclopropanecarboxamide, 2n, white solid, 30.1 mg, 0.17 mmol, yield: 85%**

^{1}H NMR (500 MHz, DMSO): δ 9.90 (s, 1H), 9.12 (s, 1H), 7.35 (d, *J* = 8.8 Hz, 2H), 6.67 (d, *J* = 8.8 Hz, 2H), 1.71 (m, 1H), 0.80–0.67 (m, 4H). **^{13}C NMR (126 MHz, DMSO):** δ 171.27, 153.49, 131.55, 121.22, 115.42, 14.74, 7.15. **HRMS (ESI)** calculated for $C_{10}H_{11}NO_2Na$ [M+Na] $^+$: 200.0682; Found: 200.0688.

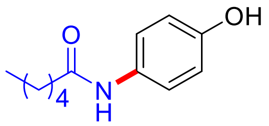

2o

***N*-(4-hydroxyphenyl)hexanamide, 2o, white solid, 21.9 mg, 0.106 mmol, yield: 53%**

^{1}H NMR (500 MHz, DMSO): δ 9.56 (s, 1H), 9.10 (s, 1H), 7.38–7.30 (m, 2H), 6.70–6.61 (m, 2H), 2.22 (t, *J* = 7.4 Hz, 2H), 1.61–1.52 (m, 2H), 1.33–1.24 (m, 4H), 0.86 (t, *J* = 7.0 Hz, 3H). **^{13}C NMR (126 MHz, DMSO):** δ 170.92, 153.48, 131.48, 121.24, 115.38, 36.64, 31.36, 25.35, 22.33, 14.29. **HRMS (ESI)** calculated for $C_{12}H_{17}NO_2Na$ [M+Na] $^+$: 230.1151; Found: 230.1151.

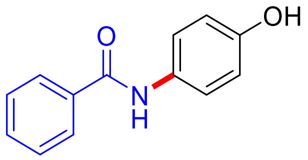

2p

***N*-(4-hydroxyphenyl)benzamide, 2p, white solid, 37.1 mg, 0.174 mmol, yield: 87%**

^{1}H NMR (500 MHz, DMSO): δ 10.03 (s, 1H), 9.33 (s, 1H), 7.92 (d, *J* = 7.3 Hz, 2H), 7.52 (m, 5H), 6.75 (d, *J* = 8.8 Hz, 2H); **^{13}C NMR (126 MHz, DMSO):** δ 165.51, 154.17, 135.53, 131.70, 131.06, 128.74, 127.89, 122.82, 115.43. **HRMS (ESI)** calculated for $C_{13}H_{11}NO_2Na$ [M+Na] $^+$: 236.0682; Found: 236.0685.

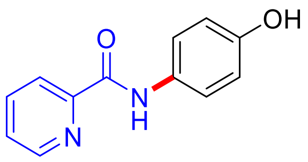

2q

***N*-(4-hydroxyphenyl)picolinamide, 2q, white solid, 34.2 mg, 0.16 mmol, yield: 80%**

^{1}H NMR (500 MHz, DMSO): δ 10.39 (s, 1H), 9.29 (s, 1H), 8.70 (dd, *J* = 4.7, 0.6 Hz, 1H), 8.13 (d, *J* = 7.8 Hz, 1H), 8.04 (m, 1H), 7.68–7.65 (m, 2H), 7.64 (m, 1H), 6.79–6.71 (m, 2H); **^{13}C NMR (126 MHz, DMSO):** δ 162.24, 154.34, 150.62, 148.76, 138.44,

130.40, 127.04, 122.54, 122.32, 115.51. **HRMS (ESI)** calculated for $C_{12}H_{10}N_2O_2Na$ $[M+Na]^+$: 237.0634; Found: 237.0636.

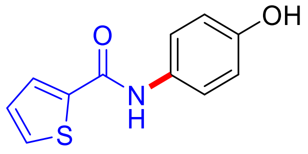

2r

***N*-(4-hydroxyphenyl)thiophene-2-carboxamide, 2r, white solid, 29.3 mg, 0.134 mmol, yield: 67%**

^{1}H NMR (400 MHz, DMSO): δ 10.07 (s, 1H), 9.32 (s, 1H), 8.00 (d, *J* = 3.6 Hz, 1H), 7.78 (t, *J* = 8.9 Hz, 1H), 7.49 (d, *J* = 8.8 Hz, 2H), 7.23–7.13 (m, 1H), 6.75 (d, *J* = 8.8 Hz, 2H); **^{13}C NMR (101 MHz, DMSO):** δ 159.91, 154.31, 140.91, 131.71, 130.59, 129.06, 128.44, 122.86, 115.51. **HRMS (ESI)** calculated for $C_{11}H_9NO_2SNa$ $[M+Na]^+$: 242.0246; Found: 242.0255.

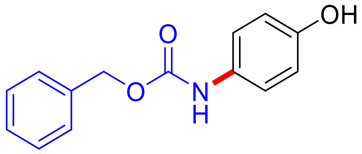

2s

***N*-(4-hydroxyphenyl)acetamide, 2s, white solid, 31.1 mg, 0.128 mmol, yield: 64%**

^{1}H NMR (500 MHz, DMSO): δ 9.42 (s, 1H), 9.14 (s, 1H), 7.43–7.35 (m, 4H), 7.35–7.31 (m, 1H), 7.24 (d, *J* = 7.7 Hz, 2H), 6.71–6.66 (m, 2H), 5.11 (s, 2H); **^{13}C NMR (126 MHz, DMSO):** δ 190.49, 154.02, 153.36, 137.32, 130.96, 128.83, 128.37, 120.56, 115.61, 65.87. **HRMS (ESI)** calculated for $C_{14}H_{13}NO_3Na$ $[M+Na]^+$: 266.0788; Found: 266.0789.

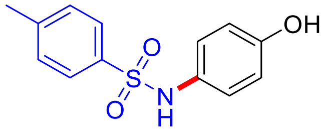

2t

***N*-(4-hydroxyphenyl)-4-methylbenzenesulfonamide, 2t, white solid, 33.7 mg, 0.128 mmol, yield: 64%**

^{1}H NMR (500 MHz, DMSO): δ 9.66 (s, 1H), 9.32 (s, 1H), 7.55 (d, *J* = 8.2 Hz, 2H), 7.28 (d, *J* = 8.1 Hz, 2H), 6.86 (d, *J* = 8.8 Hz, 2H), 6.62 (d, *J* = 8.8 Hz, 2H), 2.29 (s, 3H). **^{13}C NMR (126 MHz, DMSO):** δ 155.22, 143.25, 137.16, 129.86, 129.05, 127.18, 124.38, 115.96, 21.33. **HRMS (ESI)** calculated for $C_{13}H_{13}NO_3SNa$ $[M+Na]^+$: 286.0508; Found: 286.0511.

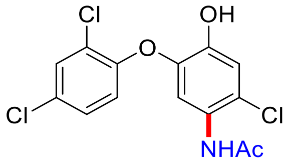

2u

***N*-(2-chloro-5-(2,4-dichlorophenoxy)-4-hydroxyphenyl)acetamide, 2u, white solid, 57.3 mg, 0.166 mmol, yield: 83%**

^{1}H NMR (500 MHz, $CDCl_3$): δ 7.77 (s, 1H), 7.45 (d, *J* = 10.2 Hz, 1H), 7.42 (d, *J* = 2.4 Hz, 1H), 7.16 (dt, *J* = 14.1, 7.0 Hz, 1H), 7.04 (s, 1H), 6.93 (d, *J* = 8.8 Hz, 1H), 6.50 (s, 1H), 2.15 (s, 3H).**^{13}C NMR (126 MHz, $CDCl_3$):** δ 168.42, 150.39, 143.91, 141.96, 130.52, 129.75, 128.18, 127.27, 125.79, 120.42, 118.70, 116.65, 112.15 , 77.27, 77.01, 76.76, 24.41. **HRMS (ESI)** calculated for $C_{14}H_{10}Cl_3NO_3Na$ $[M+Na]^+$: 367.9618; Found: 367.9614.

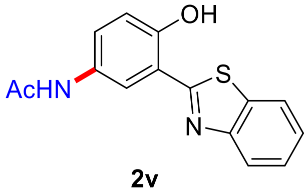

2v

***N*-(3-(benzo[d]thiazol-2-yl)-4-hydroxyphenyl)acetamide, 2v, white solid, 18.9 mg, 0.072 mmol, yield: 72%**

^{1}H NMR (300 MHz, DMSO): δ 11.30 (s, 1H), 9.96 (s, 1H), 8.44 (d, *J* = 2.5 Hz, 1H), 8.12 (d, *J* = 7.6 Hz, 1H), 8.04 (d, *J* = 7.9 Hz, 1H), 7.62 (dd, *J* = 8.8, 2.6 Hz, 1H), 7.57 – 7.47 (m, 1H), 7.43 (dd, *J* = 11.1, 4.0 Hz, 1H), 7.01 (d, *J* = 8.8 Hz, 1H), 2.03 (s, 3H). **^{13}C NMR (75 MHz, DMSO)**: δ 168.45, 165.26, 152.53, 151.92, 134.92, 132.26, 130.09, 126.92, 125.51, 124.45, 122.56, 119.02, 118.50, 117.46, 24.29. **HRMS (ESI)** calculated for C15H13N2O2S $[M+H]^+$ Exact Mass: 285.0692; Found: 285.0685.

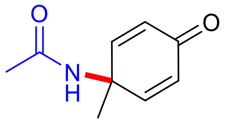

3a

***N*-(1-methyl-4-oxocyclohexa-2,5-dien-1-yl)acetamide, 3a, white solid, 23.6 mg, 0.156 mmol, yield: 78%**

^{1}H NMR (400 MHz, Acetone): δ 7.54 (s, 1H), 6.95 (d, *J* = 10.1 Hz, 2H), 6.08 (d, *J* = 10.1 Hz, 2H), 1.87 (s, 3H), 1.47 (s, 3H). **^{13}C NMR (101 MHz, Acetone):** δ 184.56, 168.98, 152.75, 126.96, 52.19, 25.59, 22.20. **HRMS (ESI)** calculated for $C_9H_{11}NO_2Na$ $[M+Na]^+$: 188.0682; Found: 188.0682.

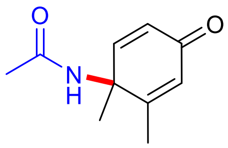

3b

***N*-(1,2-dimethyl-4-oxocyclohexa-2,5-dien-1-yl)acetamide, 3b, white solid, 30.4 mg, 0.17 mmol, yield: 85%**

^{1}H NMR (500 MHz, DMSO): δ 8.47 (s, 1H), 6.81 (d, *J* = 9.9 Hz, 1H), 5.99 (dd, *J* = 9.9, 1.4 Hz, 1H), 5.94 (s, 1H), 1.81 (s, 6H), 1.29 (s, 3H). **^{13}C NMR (126 MHz, DMSO):** δ 185.58, 169.30, 163.71, 155.84, 54.66, 26.36, 22.77, 18.66. **HRMS (ESI)** calculated for $C_{10}H_{13}NO_2Na$ $[M+Na]^+$: 202.0838; Found: 202.0839.

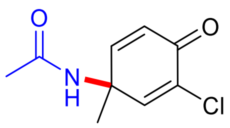

3c

***N*-(3-chloro-1-methyl-4-oxocyclohexa-2,5-dien-1-yl)acetamide, 3c, white solid, 31 mg, 0.156 mmol, yield: 78%**

^{1}H NMR (400 MHz, DMSO): δ 8.60 (s, 1H), 7.23 (d, *J* = 2.7 Hz, 1H), 6.96 (dd, *J* = 9.9, 2.7 Hz, 1H), 6.21 (d, *J* = 9.9 Hz, 1H), 1.82 (s, 3H), 1.42 (s, 3H). **^{13}C NMR (101 MHz, DMSO)**: δ 178.55, 169.87, 155.41, 151.07, 130.07, 125.70, 54.48, 25.71, 23.03. **HRMS (ESI)** calculated for $C_9H_{10}ClNO_2Na$ [M+Na] $^+$: 222.0292; Found: 222.0285.

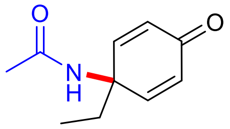

3d

***N*-(1-ethyl-4-oxocyclohexa-2,5-dien-1-yl)acetamide, 3d, white solid, 26.9 mg, 0.15 mmol, yield: 75%**

^{1}H NMR (500 MHz, DMSO): δ 8.33 (s, 1H), 6.81 (d, *J* = 10.0 Hz, 1H), 6.13 (d, *J* = 10.0 Hz, 1H), 1.81 (s, 1H), 1.76 (q, *J* = 7.4 Hz, 1H), 0.70 (t, *J* = 7.4 Hz, 1H). **^{13}C NMR (126 MHz, DMSO):** δ 185.70, 169.61, 152.87, 128.33, 56.26, 30.69, 23.21, 7.85. **HRMS (ESI)** calculated for $C_{10}H_{13}NO_2Na$ [M+Na] $^+$: 202.0838; Found: 202.0837.

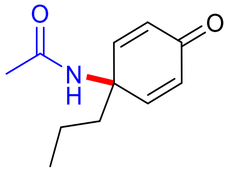

3e

***N*-(4-oxo-1-propylcyclohexa-2,5-dien-1-yl)acetamide, 3e, white solid, 31.7 mg, 0.164 mmol, yield: 82%**

^{1}H NMR (400 MHz, DMSO): δ 8.32 (s, 1H), 6.87 (d, *J* = 9.9 Hz, 2H), 6.12 (d, *J* = 9.9 Hz, 2H), 1.82 (s, 3H), 1.75–1.66 (m, 2H), 1.20–1.09 (m, 2H), 0.83 (t, *J* = 7.3 Hz, 3H). **^{13}C NMR (101 MHz, DMSO):** δ 185.74, 169.65, 153.28, 128.07, 55.92, 23.26, 16.65, 14.38. **HRMS (ESI)** calculated for $C_{11}H_{15}NO_2Na$ [M+Na]$^+$: 216.0995; Found: 216.0987.

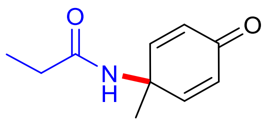

3f

***N*-(1-methyl-4-oxocyclohexa-2,5-dien-1-yl)propionamide, 3f, white solid, 28.6 mg, 0.16 mmol, yield: 80%**

^{1}H NMR (400 MHz, DMSO) δ 8.35 (s, 1H), 6.91 (d, *J* = 10.0 Hz, 2H), 6.06 (d, *J* = 10.0 Hz, 2H), 2.10 (q, *J* = 7.6 Hz, 2H), 1.36 (s, 3H), 0.94 (t, *J* = 7.6 Hz, 3H). **^{13}C NMR**

(101 MHz, DMSO) δ 185.43, 173.33, 154.78, 126.90, 52.32, 28.70, 26.26, 10.11. **HRMS (ESI)** calculated for $C_{10}H_{13}NO_2Na$ $[M+Na]^+$: 202.0838; Found: 202.0840.

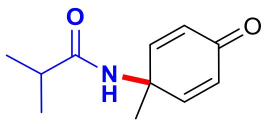

3g

***N*-(1-methyl-4-oxocyclohexa-2,5-dien-1-yl)isobutyramide, 3g, white solid, 23.5 mg, 0.122 mmol, yield: 61%**

^{1}H NMR (400 MHz, DMSO) δ 8.22 (s, 1H), 6.90 (d, J = 9.9 Hz, 2H), 6.07 (d, J = 9.9 Hz, 2H), 2.42 (dt, J = 13.5, 6.7 Hz, 1H), 1.37 (s, 3H), 0.97 (d, J = 6.8 Hz, 6H). **^{13}C NMR (101 MHz, DMSO):** δ 185.36, 176.56, 154.75, 126.95, 52.24, 34.09, 26.27, 19.94. **HRMS (ESI)** calculated for $C_{11}H_{15}NO_2Na$ $[M+Na]^+$: 216.0995; Found: 216.1008.

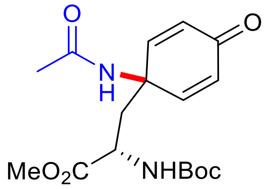

3h

methyl-3-(1-acetamido-4-oxocyclohexa-2,5-dien-1-yl)-2-((*tert*-butoxycarbonyl)amino)propanoate, 3h, white solid, 39.4 mg, 0.112 mmol, yield: 56%

^{1}H NMR (400 MHz, DMSO): δ 8.35 (s, 1H), 7.30 (d, J = 8.6 Hz, 1H), 6.87 (d, J = 9.8 Hz, 2H), 6.18 (d, J = 9.7 Hz, 1H), 6.03 (d, J = 9.9 Hz, 1H), 3.91 (t, J = 8.1 Hz, 1H), 3.60 (s, 3H), 2.41 (d, J = 12.3 Hz, 1H), 2.17 (dd, J = 13.9, 10.0 Hz, 1H), 1.81 (s, 3H), 1.34 (s, 9H). **^{13}C NMR (101 MHz, DMSO)**: δ 185.31, 172.76, 169.63, 155.22, 150.97, 150.58, 129.15, 127.74, 78.78, 54.95, 52.60, 49.66, 28.63, 23.42. **HRMS (ESI)** calculated for $C_{17}H_{24}N_2O_6Na$ $[M+Na]^+$: 375.1527; Found: 375.1531.

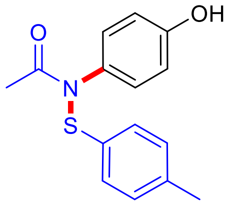

5a

***N*-(4-hydroxyphenyl)-*N*-(p-tolylthio)acetamide, 5a, white solid, 43.7 mg, 0.168 mmol, yield: 84%**

^{1}H NMR (400 MHz, DMSO): δ 9.66 (s, 1H), 7.21 (s, 4H), 7.03 (d, J = 7.7 Hz, 2H), 6.72 (d, J = 7.9 Hz, 2H), 2.28 (s, 3H), 2.12 (s, 3H); **^{13}C NMR (101 MHz, DMSO)**: δ 173.68, 157.07, 137.28, 134.50, 130.37, 128.57, 126.45, 116.03, 22.99, 21.06; **HRMS (ESI)** calculated for $C_{15}H_{16}NO_2S$ $[M+H]^+$: 274.0896; Found: 274.0906.

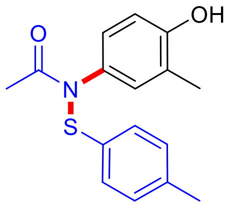

5b

***N*-(4-hydroxy-3-methylphenyl)-*N*-(*p*-tolylthio)acetamide, 5b, white solid, 47 mg, 0.164 mmol, yield:82%**

^{1}H NMR (400 MHz, DMSO) δ 9.56 (s, 1H), 7.25–7.17 (m, 4H), 6.97 (d, *J* = 2.1 Hz, 1H), 6.85 (dd, *J* = 8.4, 2.6 Hz, 1H), 6.73 (d, *J* = 8.5 Hz, 1H), 2.28 (s, 3H), 2.12 (s, 3H), 2.08 (s, 3H). **^{13}C NMR (101 MHz, DMSO)** δ 173.29, 155.20, 137.18, 137.02, 134.62, 130.33, 129.53, 126.30, 125.66, 125.11, 115.09, 22.98, 21.33, 16.42.**; HRMS (ESI)** calculated for $C_{16}H_{18}NO_2S$ $[M+H]^+$: 288.1053; Found: 288.1062.

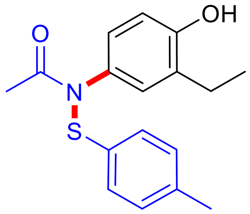

5c

***N*-(3-ethyl-4-hydroxyphenyl)-*N*-(*p*-tolylthio)acetamide, 5c, white solid, 50 mg, 0.166 mmol, yield: 83%**

^{1}H NMR (400 MHz, DMSO): δ 9.53 (s, 1H), 7.26–7.17 (m, 4H), 6.91 (d, *J* = 2.3 Hz, 1H), 6.85 (dd, *J* = 8.4, 2.5 Hz, 1H), 6.73 (d, *J* = 8.4 Hz, 1H), 2.48 (q, *J* = 7.4 Hz, 2H), 2.29 (s, 3H), 2.13 (s, 3H), 1.07 (t, *J* = 7.5 Hz, 3H). **^{13}C NMR (101 MHz, DMSO)** δ 173.76, 154.75, 137.29, 134.5, 131.03, 130.31, 127.92, 126.63, 125.59, 115.31, 105.12， 22.99, 21.07, 14.26. **HRMS (ESI)** HRMS (ESI) calculated for $C_{17}H_{20}NO_2S$ $[M+H]^+$: 302.1209; Found: 302.1205.

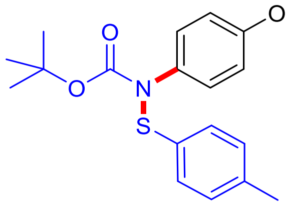

5d

***tert*-butyl (4-hydroxyphenyl)(p-tolylthio)carbamate, 5d, white solid, 27.8 mg, 0.084 mmol, yield: 42%**

^{1}H NMR (500 MHz, DMSO): δ 9.49 (s, 1H), 7.21–7.16 (m, 4H), 6.94 (d, *J* = 8.8 Hz, 2H), 6.64 (d, *J* = 8.8 Hz, 2H), 2.27 (s, 3H), 1.36 (s, 9H); **^{13}C NMR (126 MHz, DMSO)**: δ 156.33, 155.61, 137.30, 137.14, 135.28, 130.28, 127.99, 126.38, 115.55, 81.95, 28.10, 21.04; **HRMS (ESI)** calculated for $C_{18}H_{21}NNaO_3S$ $[M+Na]^+$: 354.1041; Found: 354.1047.

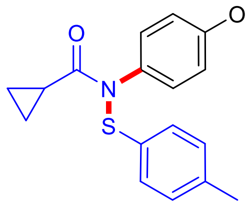

5e

***N*-(4-hydroxyphenyl)-*N*-(p-tolylthio)cyclopropanecarboxamide, 5e, white solid, 26.9 mg, 0.09 mmol, yield: 45%**

^{1}H NMR (400 MHz, DMSO): δ 9.65 (s, 1H), 7.21 (s, 4H), 7.01 (d, *J* = 8.4 Hz, 2H), 6.72 (d, *J* = 8.5 Hz, 2H), 2.29 (s, 3H), 2.04–1.89 (m, 1H), 0.88– 0.80 (m, 4H); **^{13}C NMR (101 MHz, DMSO):** δ 176.60, 157.05, 137.49, 136.87, 134.67, 130.37, 128.67, 126.82, 116.10, 21.07, 12.66, 9.81; **HRMS (ESI)** calculated for $C_{17}H_{18}NO_2S$ $[M+H]^+$: 300.1053; Found: 300.1064.

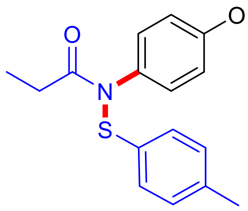

5f

***N*-(4-hydroxyphenyl)-*N*-(p-tolylthio)cyclopropanecarboxamide, 5f, white solid, 42.4 mg, 0.16 mmol, yield: 80%**

1H NMR (500 MHz, DMSO): δ 9.64 (s, 1H), 7.19 (s, 4H), 7.02–6.98 (m, 2H), 6.71 (d, *J* = 8.7 Hz, 2H), 2.39 (s, 2H), 2.27 (s, 3H), 0.99 (t, *J* = 7.3 Hz, 3H). **13C NMR (126 MHz, DMSO)**: δ 176.65, 157.05, 137.23, 136.91, 134.61, 130.29, 128.62, 126.47, 116.02, 27.64, 21.01, 10.01. **HRMS (ESI)** calculated for $C_{16}H_{18}NO_2S$ $[M+H]^+$: 288.1053; Found: 288.1049.

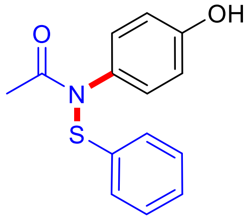

5g

***N*-(4-hydroxyphenyl)-*N*-(phenylthio)acetamide, 5g, white solid, 44 mg, 0.17 mmol, yield: 85%**

^{1}H NMR (400 MHz, DMSO): δ 9.65 (s, 1H), 7.47–7.21 (m, 5H), 7.10 (d, *J* = 7.4 Hz, 2H), 6.75 (d, *J* = 7.5 Hz, 2H), 2.16 (s, 3H); **^{13}C NMR (101 MHz, DMSO)**: δ 173.67, 157.16, 138.20, 137.18, 129.75, 128.54, 127.17, 124.95, 116.08, 22.93; **HRMS (ESI)** calculated for $C_{14}H_{14}NO_2S$ $[M+H]^+$: 260.0740; Found: 260.0742.

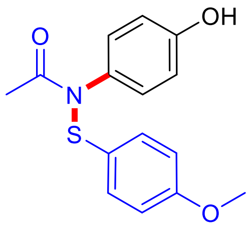

5h

***N*-(4-hydroxyphenyl)-*N*-((4-methoxyphenyl)thio)acetamide, 5h, white solid, 43.4 mg, 0.165 mmol, yield: 75%**

^{1}H NMR (500 MHz, DMSO): δ 9.61 (s, 1H), 7.32 (d, *J* = 8.4 Hz, 2H), 6.94 (d, *J* = 8.5 Hz, 2H), 6.89 (d, *J* = 8.4 Hz, 2H), 6.69 (d, *J* = 8.4 Hz, 2H), 2.01 (s, 3H); **^{13}C NMR (126 MHz, DMSO)**: δ 173.33, 160.20, 157.01, 137.17, 131.83, 128.70, 128.07, 116.05, 115.33, 55.74, 23.06; **HRMS (ESI)** calculated for $C_{15}H_{16}NO_3S$ $[M+H]^+$: 290.0845; Found: 290.0857.

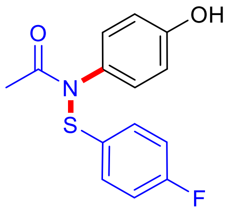

5i

***N*-((4-fluorophenyl)thio)-*N*-(4-hydroxyphenyl)acetamide, 5i, white solid, 34.9 mg, 0.126 mmol, yield: 63%**

^{1}H NMR (400 MHz, DMSO): δ 9.66 (s, 1H), 7.43–7.34 (m, 2H), 7.24 (t, *J* = 8.9 Hz, 2H), 7.03 (d, *J* = 8.7 Hz, 2H), 6.72 (d, *J* = 8.7 Hz, 2H), 2.11 (s, 3H); **^{13}C NMR (101 MHz, DMSO)**: δ 161.94 (d, *J* = 244.8 Hz), 157.20, 137.05, 133.50 (d, *J* = 2.9 Hz), 129.13, 128.64, 116.82 (d, *J* = 22.2 Hz), 116.11, 23.03; **^{19}F NMR (376 MHz, DMSO)**: δ -114.63; HRMS (ESI) calculated for $C_{14}H_{13}FNO_2S$ $[M+H]^+$: 278.0646; Found: 278.0644.

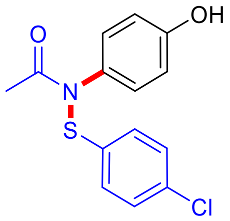

5j

***N*-((4-chlorophenyl)thio)-*N*-(4-hydroxyphenyl)acetamide, 5j, white solid, 46.3 mg, 0.158 mmol, yield: 79%**

^{1}H NMR (400 MHz, DMSO): δ 9.67 (s, 1H), 7.46–7.42 (m, 2H), 7.35–7.30 (m, 2H), 7.10 (d, *J* = 8.6 Hz, 2H), 6.73 (d, *J* = 8.8 Hz, 2H), 2.11 (s, 3H); **^{13}C NMR (101 MHz, DMSO)**: δ 173.66, 157.28, 137.28, 136.98, 131.72, 129.66, 128.59, 126.85, 116.12, 22.96; **HRMS (ESI)** calculated for $C_{14}H_{13}ClNO_2S$ $[M+H]^+$: 294.0350; Found: 294.0356.

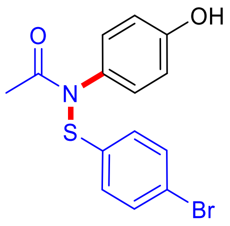

5k

***N*-((4-bromophenyl)thio)-*N*-(4-hydroxyphenyl)acetamide, 5k, white solid, 48.5 mg, 0.144 mmol, yield: 72%**

^{1}H NMR (400 MHz, DMSO): δ 9.69 (s, 1H), 7.55 (d, *J* = 8.5 Hz, 2H), 7.26 (d, *J* = 8.5 Hz, 2H), 7.10 (d, *J* = 8.5 Hz, 2H), 6.74 (d, *J* = 8.4 Hz, 2H), 2.12 (s, 3H); **^{13}C NMR (101 MHz, DMSO):** δ 173.69, 157.28, 137.85, 136.97, 132.52, 128.58, 126.96, 119.98, 116.15, 22.97; **HRMS (ESI)** calculated for $C_{14}H_{13}BrNO_2S$ $[M+H]^+$: 337.9845; Found: 337.9845.

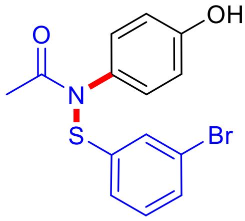

5l

***N*-((3-bromophenyl)thio)-*N*-(4-hydroxyphenyl)acetamide, 5l, white solid, 49.9 mg, 0.148 mmol, yield: 74%**

^{1}H NMR (400 MHz, DMSO): δ 9.72 (s, 1H), 7.45 (s, 1H), 7.41 (m, 1H), 7.36–7.28 (m, 2H), 7.13 (d, *J* = 8.6 Hz, 2H), 6.75 (d, *J* = 8.7 Hz, 2H), 2.13 (s, 3H); **^{13}C NMR (101 MHz, DMSO):** δ 173.53, 157.32, 141.12, 136.91, 131.68, 129.73, 128.55, 126.51, 123.47, 122.87, 116.18, 23.01; **HRMS (ESI)** calculated for $C_{14}H_{13}BrNO_2S$ $[M+H]^+$: 337.9845; Found: 337.9843.

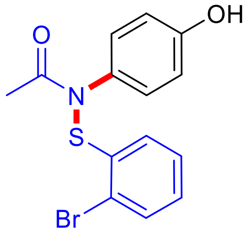

5m

***N*-((2-bromophenyl)thio)-*N*-(4-hydroxyphenyl)acetamide, 5m, white solid, 44.5 mg, 0.132 mmol, yield: 66%**

^{1}H NMR (500 MHz, DMSO): δ 9.72 (s, 1H), 7.55 (d, *J* = 7.9, 1H), 7.45 (m, 1H), 7.36 (d, *J* = 7.4 Hz, 1H), 7.25 (d, *J* = 8.1 Hz, 2H), 7.12 (m, 1H), 6.74 (d, *J* = 8.7 Hz, 2H), 2.12 (s, 3H); **^{13}C NMR (126 MHz, DMSO)**: δ 173.34, 157.37, 138.59, 136.73, 133.18, 128.96, 128.63, 127.66, 124.16, 116.17, 115.54, 22.88; **HRMS (ESI)** calculated for $C_{14}H_{12}BrNNaO_2S$ $[M+Na]^+$: 359.9664; Found: 359.9668.

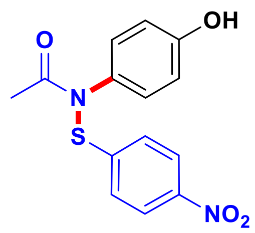

5n

***N*-(4-hydroxyphenyl)-*N*-((4-nitrophenyl)thio)acetamide, 5n, white solid, 47.7 mg, 0.144 mmol, yield: 72%**

^{1}H NMR (500 MHz, DMSO): δ 9.74 (s, 1H), 8.20 (d, *J* = 8.5 Hz, 2H), 7.53 (d, *J* = 8.4 Hz, 2H), 7.25 (d, *J* = 7.8 Hz, 2H), 6.75 (d, *J* = 8.3 Hz, 2H), 2.14 (s, 3H); **^{13}C NMR (126 MHz, DMSO)**: δ 173.34, 157.55, 148.30, 145.77, 136.64, 128.67, 124.70, 123.43, 116.27, 22.87; **HRMS (ESI)** calculated for $C_{18}H_{21}NNaO_3S$ $[M+Na]^+$: 354.1134; Found: 354.1147.

Supplementary Tables

Supplementary Table 1. Screening of Se-catalyzed *para*-amination of phenols[a]:

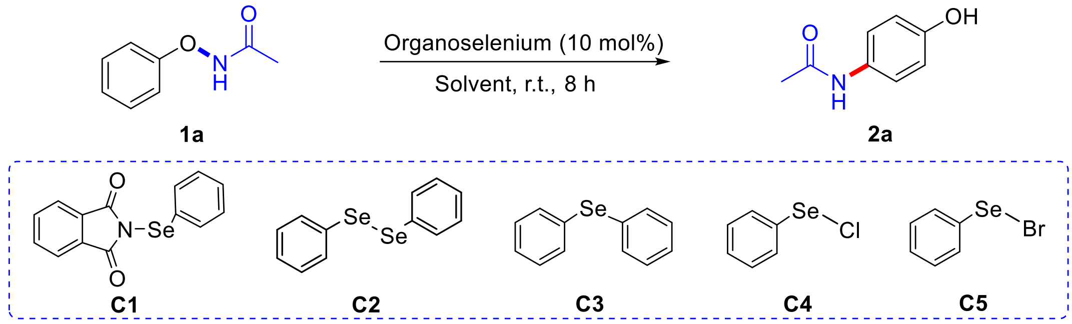

Entry	Catalyst	Solvent	Yield[b] (%)
1[c]	**C1**	TFE	47
2[d]	**C1**	TFE	42
3	**C1**	TFE	38
4	**C2**	TFE	N.D.
5	**C3**	TFE	N.D.
6	**C4**	TFE	60
7	**C5**	TFE	79
8	**C5**	MeOH	N.D.
9	**C5**	DMSO	N.D.
10	**C5**	THF	73
11	**C5**	MeCN	86
12	**C5**	EA	78
13	**C5**	1,4-dioxane	93(90[e])
14[f]	**C5**	1,4-dioxane	92
15	-	1,4-dioxane	N.D.

[a]Standard conditions: **1a** (0.10 mmol), catalysts (10 mol%), solvents (1.0 mL), at ambient temperature for 8 h; [b]NMR yield used 1,4-dimethoxybenzene as internal standard. [c]0.5 equiv. CsOAc and 1.0 equiv. **C1** were used; [d]1.0 equiv. **C1** were used; [e]Isolated yield; [f]Run under N_2; TFE, 2,2,2-trifluoroethanol; N.D. = not detected.

Supplementary Table 2. Screening of S-mediated conditions[a]:

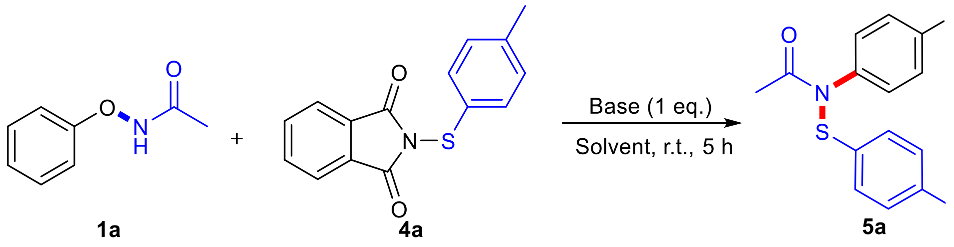

Entry	Base	Solvent	Yield[b](%)
1[c]	Et_3N	MeOH	< 5
2	Et_3N	MeOH	38
3	Pyridine	MeOH	46
4	CsOAc	MeOH	trace
5	2,6-lutidine	MeOH	53
6	DMAP	MeOH	31
7	Na_2CO_3	MeOH	11
8	DBU	MeOH	35
9	DIPEA	MeOH	33
10	2,6-lutidine	DMSO	17
11	2,6-lutidine	1,4-dioxane	64
12	2,6-lutidine	CH_3CN	85
13	2,6-lutidine	DCE	38
14	2,6-lutidine	TFE	87(84[d])
15	2,6-lutidine	THF	42
16	2,6-lutidine	Toluene	N.D.
17[e]	2,6-lutidine	TFE	73
18[f]	2.6-lutidine	TFE	88

[a]Standard conditions: **1a** (0.1 mmol), **4a** (0.12 mmol), base (1.0 eq.), solvents (1mL), at ambient temperature for 5h; [b]NMR yield used 1,4-dimethoxybenzene as internal standard; [c]10 mol% **4a** was used; [d]Isolated yield; [e]Run for 3 h; [f]Run for 8 h; TFE, 2,2,2-trifluoroethanol; N.D. = not detected.

Supplementary Table 3. Crystal data and structure refinement for **2n.**

Identification code	**2n**
Empirical formula	$C_{10}H_{11}NO_2$
Formula weight	177.20
Temperature/K	296.15
Crystal system	orthorhombic
Space group	P b c a
a/Å	12.521(9)
b/Å	10.988(9)
c/Å	13.396(11)
α/°	90
β/°	90

$\gamma/°$	90
Volume/$Å^3$	1843(3)
Z	8
$\rho_{calc}g/cm^3$	1.277
μ/mm^{-1}	0.090
F(000)	752.0
Crystal size/mm^3	0.26 × 0.25 × 0.22
Radiation	Mo Kα (λ = 0.71073)
2Θ range for data collection/°	5.794 to 50.004
Index ranges	$-13 \leq h \leq 14$, $-13 \leq k \leq 9$, $-15 \leq l \leq 15$
Reflections collected	9500
Independent reflections	1622 [R_{int} = 0.0391, R_{sigma} = 0.0263]
Data/restraints/parameters	1622/0/119
Goodness-of-fit on F^2	1.112
Final R indexes [I>=2σ (I)]	R_1 = 0.0393, wR_2 = 0.1168
Final R indexes [all data]	R_1 = 0.0517, wR_2 = 0.1282
Largest diff. peak/hole / e $Å^{-3}$	0.19/-0.15

Supplementary Table 4. Fractional Atomic Coordinates (×10^4) and Equivalent Isotropic Displacement Parameters ($Å^2$×10^3) for **2n**. U(eq) is defined as 1/3 of the trace of the orthogonalized U^{ij} tensor.

Atom	*x*	*y*	*z*	U(eq)
O1	4999.3(8)	5326.4(12)	6384.1(9)	47.0(4)
O2	1946.8(10)	8678.5(12)	3574.6(9)	53.2(4)
N1	3384.4(10)	6049.1(13)	6873.8(10)	39.4(4)
C1	4926.8(17)	3689(2)	8131(2)	74.9(7)
C2	5523(2)	4739(3)	8442(2)	83.0(8)
C3	4421.5(15)	4912.0(19)	8036.5(14)	52.2(5)
C4	4305.5(13)	5443.3(15)	7031.1(12)	37.3(4)
C5	3046.6(12)	6699.1(14)	6013.1(12)	34.9(4)
C6	3511.8(14)	6577.0(18)	5082.2(13)	46.8(5)
C7	3124.7(14)	7237.1(19)	4287.1(13)	51.6(5)
C8	2273.5(12)	8029.0(16)	4399.9(12)	39.4(4)
C9	1797.3(14)	8142.6(17)	5319.7(13)	45.3(5)
C10	2184.3(13)	7488.9(17)	6117.9(13)	43.6(5)

Supplementary Table 5. Anisotropic Displacement Parameters ($Å^2$×10^3) for **2n**. The Anisotropic displacement factor exponent takes the form: $-2\pi^2 [h^2a^{*2}U_{11}+2hka^*b^*U_{12}+\ldots]$.

Atom	U_{11}	U_{22}	U_{33}	U_{23}	U_{13}	U_{12}
O1	34.0(7)	63.1(9)	43.8(7)	-3.1(6)	4.7(5)	3.1(5)
O2	44.9(7)	78.4(10)	36.2(7)	13.1(6)	6.5(5)	15.3(6)
N1	37.3(7)	49.3(9)	31.7(8)	2.3(6)	7.3(6)	5.9(6)
C1	62.0(14)	69.5(16)	93.3(17)	29.7(13)	9.8(12)	12.9(12)
C2	66.2(15)	111(2)	71.6(16)	36.8(15)	-25.2(12)	-17.0(15)
C3	50.4(11)	61.5(13)	44.7(11)	8.8(9)	4.5(9)	13.7(9)
C4	36.0(8)	38.1(9)	37.6(9)	-4.1(7)	1.3(7)	-2.0(7)
C5	32.7(8)	40.3(9)	31.5(9)	-2.4(7)	2.8(6)	-0.3(7)
C6	42.8(10)	58.6(12)	39.1(10)	0.1(8)	8.9(7)	15.2(9)
C7	48.8(10)	74.2(14)	31.8(10)	1.1(9)	12.1(8)	15.2(10)
C8	35.5(9)	50.4(10)	32.3(9)	3.3(7)	1.1(7)	1.0(7)
C9	39.6(9)	56.2(12)	40.1(10)	-0.3(8)	6.9(8)	12.8(8)
C10	42.1(9)	56.6(11)	32.1(9)	-1.0(8)	9.5(7)	10.6(8)

Supplementary Table 6. Bond Lengths for **2n.**

Atom	Atom	Length/Å	Atom	Atom	Length/Å
O1	C4	1.234(2)	C3	C4	1.475(3)
O2	C8	1.378(2)	C5	C6	1.383(3)
N1	C4	1.348(2)	C5	C10	1.392(2)
N1	C5	1.421(2)	C6	C7	1.377(3)
C1	C2	1.436(4)	C7	C8	1.384(3)
C1	C3	1.491(3)	C8	C9	1.374(2)
C2	C3	1.494(3)	C9	C10	1.376(3)

Supplementary Table 7. Bond Angles for **2n.**

Atom	Atom	Atom	Angle/°	Atom	Atom	Atom	Angle/°
C4	N1	C5	129.04(13)	C6	C5	C10	118.55(16)
C2	C1	C3	61.33(18)	C10	C5	N1	117.54(14)
C1	C2	C3	61.14(15)	C7	C6	C5	119.89(17)
C1	C3	C2	57.53(15)	C6	C7	C8	121.18(16)
C4	C3	C1	118.42(18)	O2	C8	C7	117.80(15)
C4	C3	C2	118.21(18)	O2	C8	C9	122.92(16)
O1	C4	N1	122.94(15)	C9	C8	C7	119.28(15)
O1	C4	C3	122.07(15)	C10	C9	C8	119.76(16)
N1	C4	C3	114.99(14)	C9	C10	C5	121.33(15)
C6	C5	N1	123.89(15)				

Supplementary Table 8. Torsion Angles for **2n.**

A	B	C	D	Angle/°	A	B	C	D	Angle/°
O2	C8	C9	C10	-178.25(17)	C4	N1	C5	C10	-164.63(17)
N1	C5	C6	C7	179.42(17)	C5	N1	C4	O1	-3.5(3)
N1	C5	C10	C9	-179.20(16)	C5	N1	C4	C3	177.24(16)
C1	C2	C3	C4	-107.4(2)	C5	C6	C7	C8	0.1(3)
C1	C3	C4	O1	-38.2(3)	C6	C5	C10	C9	-0.2(3)
C1	C3	C4	N1	141.09(18)	C6	C7	C8	O2	178.55(18)
C2	C1	C3	C4	107.0(2)	C6	C7	C8	C9	-1.0(3)
C2	C3	C4	O1	28.1(3)	C7	C8	C9	C10	1.3(3)
C2	C3	C4	N1	-152.64(19)	C8	C9	C10	C5	-0.7(3)
C4	N1	C5	C6	16.4(3)	C10	C5	C6	C7	0.5(3)

Supplementary Table 9. Hydrogen Atom Coordinates (Å×10^4) and Isotropic Displacement Parameters (Å^2×10^3) for **2n.**

**Atom**	***x***	***y***	***z***	**U(eq)**
H2	1367	8996	3689	80
H1	2940	6041	7363	47
H1A	4659	3150	8647	90
H1B	5149	3284	7522	90
H2A	6118	4993	8027	100
H2B	5628	4859	9152	100
H3	3862	5110	8522	63
H6	4085	6050	4993	56
H7	3441	7149	3663	62
H9	1216	8659	5402	54
H10	1863	7577	6740	52

Supplementary Table 10. Crystal data and structure refinement for **5a**.

Identification code	**5a**	
Empirical formula	$C_{15}H_{15}NO_2S$	
Formula weight	273.34	
Temperature	296(2) K	
Wavelength	0.71073 Å	
Crystal system	Triclinic	
Space group	P-1	
Unit cell dimensions	a = 5.897(5) Å	α= 90.141(14)°.

	b = 14.261(12) Å β= 92.047(13) °.
	c = 16.469(14) Å γ = 96.973(13) °.
Volume	1374(2) $Å^3$
Z	4
Density (calculated)	1.321 Mg/m^3
Absorption coefficient	0.232 mm^{-1}
F(000)	576
Crystal size	0.260 x 0.210 x 0.190 mm^3
Theta range for data collection	1.439 to 25.008 °.
Index ranges	-6<=h<=7, -16<=k<=16, -19<=l<=19
Reflections collected	6670
Independent reflections	4679 [R(int) = 0.0511]
Completeness to theta = 25.008 °	97.1 %
Refinement method	Full-matrix least-squares on F^2
Data / restraints / parameters	4679 / 0 / 349
Goodness-of-fit on F^2	1.072
Final R indices [I>2 σ (I)]	R1 = 0.0935, wR2 = 0.2411
R indices (all data)	R1 = 0.1375, wR2 = 0.2640
Extinction coefficient	n/a
Largest diff. peak/hole	0.447 and -0.315 e.$Å^{-3}$

Supplementary Table 11. Atomic coordinates (x 10^4) and equivalent isotropic displacement parameters ($Å^2$x 10^3) for **5a**. U_{eq} is defined as one third of the trace of the orthogonalized U_{ij} tensor.

Atom	x	y	z	U_{eq}
C(1)	2208(13)	-1487(5)	8812(4)	62(2)
C(2)	3863(11)	-609(5)	8773(3)	47(2)
C(3)	6002(11)	-543(5)	9125(4)	50(2)
C(4)	7500(11)	276(5)	9099(3)	48(2)
C(5)	6895(9)	1052(4)	8692(3)	39(1)
C(6)	4751(10)	1003(5)	8335(4)	49(2)
C(7)	3254(10)	173(4)	8359(4)	46(2)
C(8)	6140(10)	3415(4)	8636(3)	42(1)
C(9)	5814(14)	3240(5)	9517(4)	69(2)

C(10)	7919(9)	3060(4)	7362(3)	35(1)
C(11)	10053(10)	3393(4)	7120(4)	46(2)
C(12)	10412(10)	3546(5)	6302(4)	54(2)
C(13)	8663(10)	3365(4)	5741(4)	44(1)
C(14)	6520(10)	3014(4)	5980(3)	43(1)
C(15)	6182(10)	2855(4)	6799(3)	45(2)
C(16)	12762(13)	10900(5)	5588(5)	77(2)
C(17)	11252(11)	10185(4)	6033(4)	52(2)
C(18)	9335(12)	10420(5)	6416(4)	58(2)
C(19)	7899(11)	9757(4)	6803(4)	51(2)
C(20)	8346(9)	8834(4)	6836(3)	40(1)
C(21)	10241(11)	8583(4)	6479(4)	53(2)
C(22)	11698(11)	9255(5)	6079(4)	57(2)
C(23)	5859(10)	6567(4)	6407(3)	42(1)
C(24)	6153(12)	5560(5)	6204(4)	59(2)
C(25)	8441(8)	6471(4)	7614(3)	33(1)
C(26)	7886(9)	6301(4)	8406(3)	40(1)
C(27)	9168(10)	5802(4)	8904(3)	46(2)
C(28)	11125(10)	5470(4)	8621(3)	40(1)
C(29)	11735(10)	5665(4)	7832(3)	42(1)
C(30)	10406(9)	6167(4)	7331(3)	40(1)
N(1)	7549(8)	2895(3)	8226(3)	38(1)
N(2)	7006(7)	6968(3)	7076(3)	38(1)
O(1)	5190(7)	4015(3)	8264(2)	51(1)
O(2)	9101(8)	3539(4)	4941(3)	66(1)
O(3)	4678(7)	7020(4)	5981(2)	60(1)
O(4)	12338(8)	4954(3)	9128(2)	61(1)
S(1)	8949(3)	2059(1)	8690(1)	50(1)
S(2)	6417(3)	8047(1)	7382(1)	46(1)

Supplementary Table 12. Bond Lengths for **5a**.

Atom	Atom	Length/Å	Atom	Atom	Length/Å
C1	C2	1.494(9)	C17	C22	1.385(9)
C2	C3	1.362(9)	C17	C18	1.390(10)
C2	C7	1.385(8)	C18	C19	1.366(9)
C3	C4	1.378(9)	C19	C20	1.373(8)
C4	C5	1.372(8)	C20	C21	1.366(8)
C5	C6	1.369(8)	C20	S2	1.769(6)
C5	S1	1.764(6)	C21	C22	1.391(9)

C6	C7	1.390(9)	C23	O3	1.213(7)
C8	O1	1.231(7)	C23	N2	1.360(7)
C8	N1	1.372(7)	C23	C24	1.505(9)
C8	C9	1.488(8)	C25	C26	1.372(7)
C10	C15	1.362(7)	C25	C30	1.382(8)
C10	C11	1.364(8)	C25	N2	1.448(7)
C10	N1	1.462(7)	C26	C27	1.356(8)
C11	C12	1.384(9)	C27	C28	1.395(8)
C12	C13	1.361(8)	C28	O4	1.356(6)
C13	O2	1.369(7)	C28	C29	1.380(7)
C13	C14	1.373(8)	C29	C30	1.379(8)
C14	C15	1.386(8)	N1	S1	1.699(5)
C16	C17	1.483(9)	N2	S2	1.697(5)

Supplementary Table 13. Bond Angles for **5a**.

**Atom**	**Atom**	**Atom**	**Angle/°**	**Atom**	**Atom**	**Atom**	**Angle/°**
C3	C2	C7	117.5(6)	C18	C19	C20	120.4(6)
C3	C2	C1	122.4(6)	C21	C20	C19	119.5(6)
C7	C2	C1	120.1(6)	C21	C20	S2	124.1(5)
C2	C3	C4	122.0(6)	C19	C20	S2	116.3(5)
C5	C4	C3	120.5(6)	C20	C21	C22	120.3(6)
C6	C5	C4	118.6(6)	C17	C22	C21	120.8(6)
C6	C5	S1	124.6(5)	O3	C23	N2	120.5(6)
C4	C5	S1	116.8(4)	O3	C23	C24	121.5(5)
C5	C6	C7	120.5(6)	N2	C23	C24	118.0(5)
C2	C7	C6	120.8(6)	C26	C25	C30	119.1(5)
O1	C8	N1	119.0(5)	C26	C25	N2	120.9(5)
O1	C8	C9	121.9(6)	C30	C25	N2	119.9(5)
N1	C8	C9	119.1(5)	C27	C26	C25	121.3(5)
C15	C10	C11	119.9(5)	C26	C27	C28	120.2(5)
C15	C10	N1	120.8(5)	O4	C28	C29	122.5(5)
C11	C10	N1	119.2(5)	O4	C28	C27	118.6(5)
C10	C11	C12	119.4(5)	C29	C28	C27	118.9(5)
C13	C12	C11	120.7(5)	C30	C29	C28	120.4(5)
C12	C13	O2	118.4(5)	C29	C30	C25	120.1(5)
C12	C13	C14	120.1(6)	C8	N1	C10	120.2(5)
O2	C13	C14	121.4(5)	C8	N1	S1	122.3(4)
C13	C14	C15	118.7(5)	C10	N1	S1	117.5(3)
C10	C15	C14	121.1(5)	C23	N2	C25	123.6(5)
C22	C17	C18	117.5(6)	C23	N2	S2	118.3(4)

C22	C17	C16	121.1(7)	C25	N2	S2	117.5(4)
C18	C17	C16	121.4(6)	N1	S1	C5	103.9(3)
C19	C18	C17	121.5(6)	N2	S2	C20	103.2(3)

Supplementary Table 14. Anisotropic displacement parameters (Å^{2}x 10^3) for **5a**. The anisotropic displacement factor exponent takes the form: $-2\pi^2 2[h^2 a^{*2}U_{11} + ... + 2 h k a^* b^* U_{12}]$

**Atom**	**U_{11}**	**U_{22}**	**U_{33}**	**U_{23}**	**U_{13}**	**U_{12}**
C(1)	68(5)	55(4)	63(4)	3(3)	6(3)	6(4)
C(2)	49(4)	53(4)	42(3)	-6(3)	6(3)	13(3)
C(3)	57(4)	46(4)	52(4)	5(3)	4(3)	25(3)
C(4)	43(4)	58(4)	46(3)	-3(3)	-10(3)	19(3)
C(5)	37(3)	38(3)	44(3)	-6(2)	-2(2)	12(3)
C(6)	44(4)	47(4)	60(4)	2(3)	-8(3)	23(3)
C(7)	38(3)	53(4)	49(4)	1(3)	-5(3)	14(3)
C(8)	47(4)	38(3)	39(3)	-7(3)	-11(3)	9(3)
C(9)	105(6)	64(5)	45(4)	-5(3)	-9(4)	37(5)
C(10)	33(3)	34(3)	40(3)	-6(2)	-8(2)	9(3)
C(11)	31(3)	52(4)	55(4)	-6(3)	-9(3)	3(3)
C(12)	22(3)	69(5)	69(4)	-3(3)	-4(3)	-4(3)
C(13)	39(3)	47(4)	46(3)	-1(3)	2(3)	7(3)
C(14)	38(3)	41(4)	47(3)	-4(3)	-9(3)	-2(3)
C(15)	28(3)	57(4)	47(3)	-2(3)	-6(2)	-7(3)
C(16)	69(5)	55(5)	100(6)	3(4)	-13(4)	-12(4)
C(17)	48(4)	38(4)	67(4)	1(3)	-18(3)	-5(3)
C(18)	66(5)	34(4)	73(5)	-1(3)	-13(4)	14(4)
C(19)	53(4)	34(4)	68(4)	-9(3)	-7(3)	16(3)
C(20)	34(3)	43(4)	45(3)	-3(3)	-8(2)	13(3)
C(21)	51(4)	34(4)	78(5)	6(3)	8(3)	15(3)
C(22)	41(4)	57(5)	72(4)	-3(3)	5(3)	3(3)
C(23)	34(3)	49(4)	40(3)	-1(3)	-1(3)	0(3)
C(24)	62(4)	56(5)	56(4)	-9(3)	1(3)	-6(4)
C(25)	23(3)	36(3)	39(3)	1(2)	-1(2)	0(2)
C(26)	29(3)	51(4)	42(3)	-3(3)	1(2)	12(3)
C(27)	49(4)	55(4)	37(3)	4(3)	7(3)	13(3)
C(28)	40(3)	40(3)	42(3)	3(3)	0(3)	12(3)
C(29)	40(3)	44(4)	44(3)	5(3)	11(3)	14(3)
C(30)	36(3)	45(4)	41(3)	4(3)	7(2)	8(3)
N(1)	37(3)	39(3)	42(3)	-6(2)	-8(2)	15(2)

N(2)	32(2)	42(3)	42(3)	-2(2)	0(2)	7(2)
O(1)	57(3)	49(3)	49(2)	-3(2)	-6(2)	22(2)
O(2)	57(3)	93(4)	47(3)	6(2)	10(2)	0(3)
O(3)	52(3)	81(3)	48(3)	8(2)	-13(2)	14(3)
O(4)	74(3)	74(3)	43(2)	7(2)	-2(2)	41(3)
S(1)	42(1)	48(1)	63(1)	3(1)	-18(1)	17(1)
S(2)	44(1)	46(1)	53(1)	1(1)	9(1)	19(1)

Supplementary Table 15. Hydrogen coordinates (Å x 10^4) and isotropic displacement parameters (Å^2 x 10^{3}) for **5a.**

**Atom**	**x**	**y**	**z**	**U(eq)**
H(1A)	2980	-2027	8704	93
H(1B)	990	-1458	8414	93
H(1C)	1591	-1541	9344	93
H(3)	6465	-1066	9391	60
H(4A)	8932	303	9359	58
H(6)	4292	1530	8075	59
H(7)	1827	141	8095	56
H(9A)	7238	3410	9813	104
H(9B)	5304	2583	9598	104
H(9C)	4691	3614	9707	104
H(11)	11256	3517	7501	56
H(12)	11863	3774	6135	65
H(14)	5318	2887	5599	52
H(15)	4746	2604	6966	54
H(16A)	12071	11475	5549	115
H(16B)	12977	10667	5052	115
H(16C)	14215	11020	5874	115
H(18)	9023	11044	6409	69
H(19)	6612	9931	7046	62
H(21)	10557	7960	6503	64
H(22)	12988	9077	5839	68
H(24A)	4822	5272	5904	89
H(24B)	6348	5217	6697	89
H(24C)	7475	5549	5882	89
H(26)	6606	6532	8606	48
H(27)	8741	5682	9435	55
H(29)	13050	5457	7638	50

H(30)	10834	6300	6802	48
H(2)	8011	3304	4655	99
H(4)	13185	4667	8864	91

Supplementary Figures

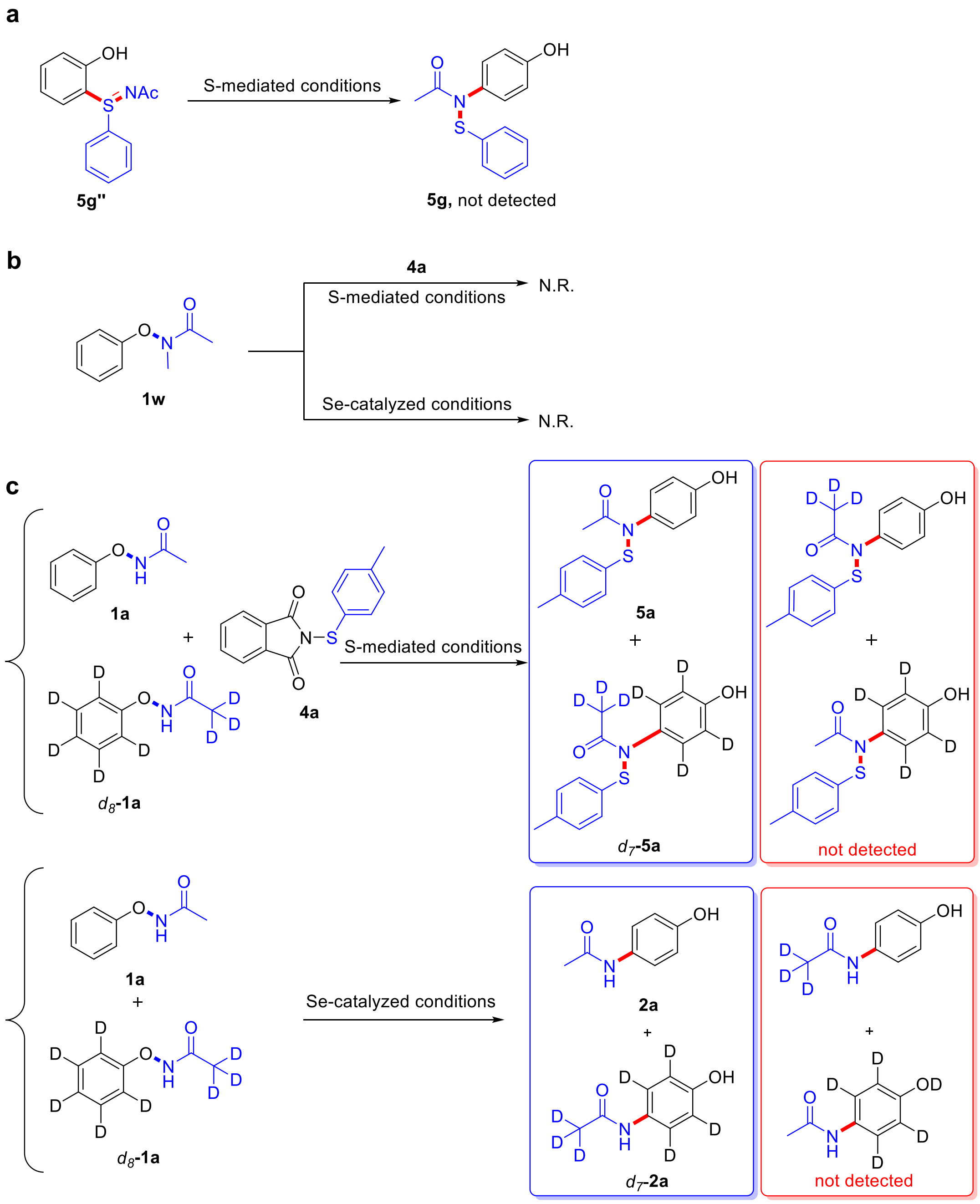

Supplementary Figure 1. **(a)** Control experiments. **(b)** Experiments to prove the importance of N–H bond. **(c)** Two crossover experiments to probe the mechanism. The results of crossover experiments were detected by HRMS. S-mediated reaction conditions: **1a** (0.2 mmol), d_8-**1a** (0.2 mmol), **4a** (0.48 mmol), TFE (2.0 mL), at ambient temperature for 5 h; Se-catalyzed reaction conditions: **1a** (0.2 mmol), d_8-**1a** (0.2 mmol), PhSeBr (10 mol%), 1,4-dioxane (2.0 mL), at ambient temperature for 8 h.

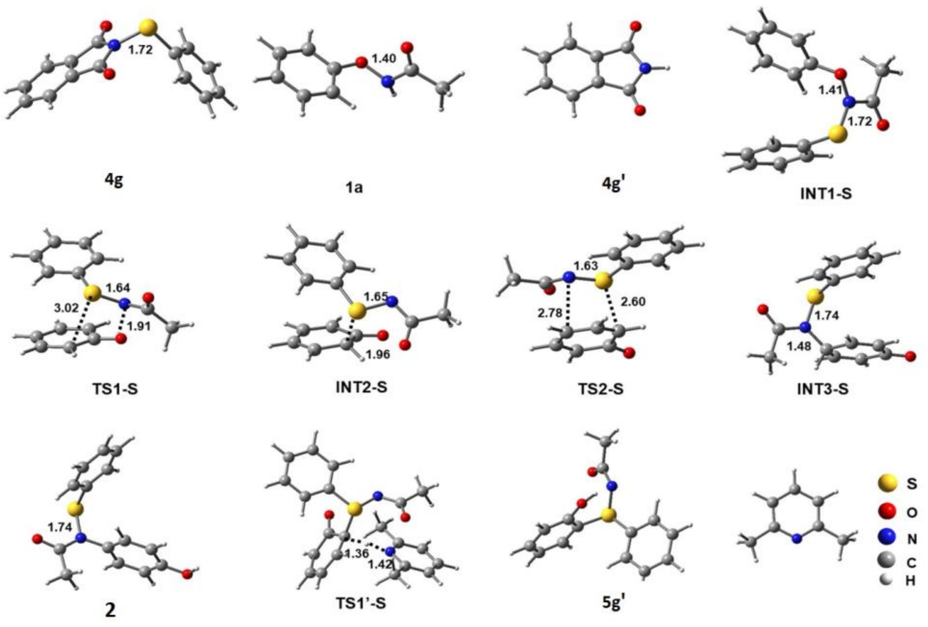

Supplementary Figure 2. Optimized structures of all stationary points involved in S-mediated reaction in TFE. All bond lengths are in Å.

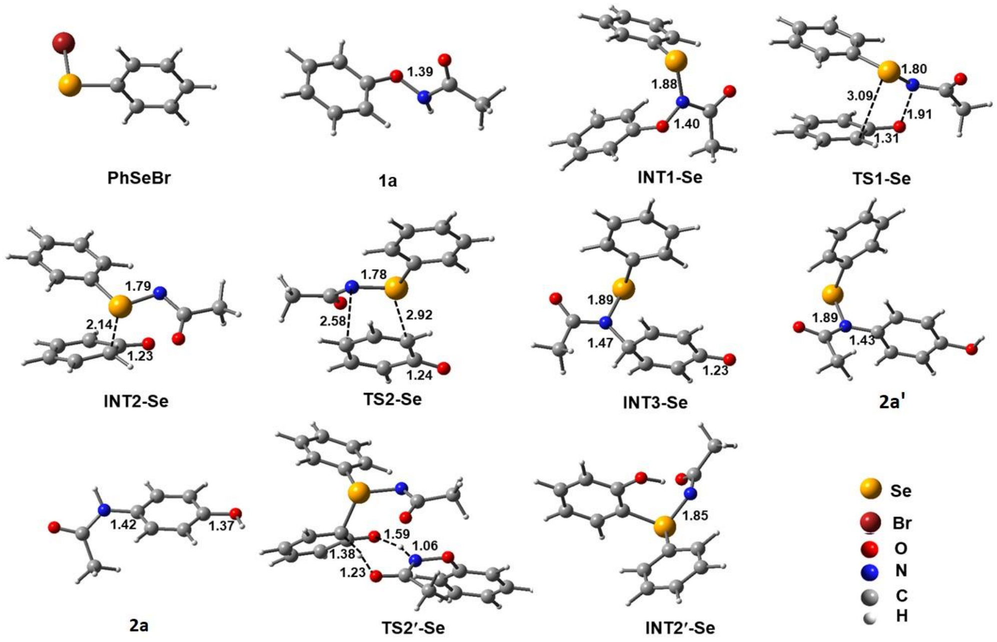

Supplementary Figure 3. Optimized structures of all stationary points for the reaction between the *N*-phenoxyacetamide **1a** and PhSeBr in the solvent (1,4-dioxane). All bond lengths are in Å.

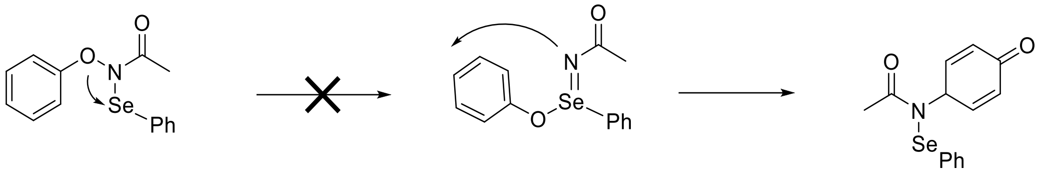

INT1-Se **INT1'-Se** **INT3-Se**

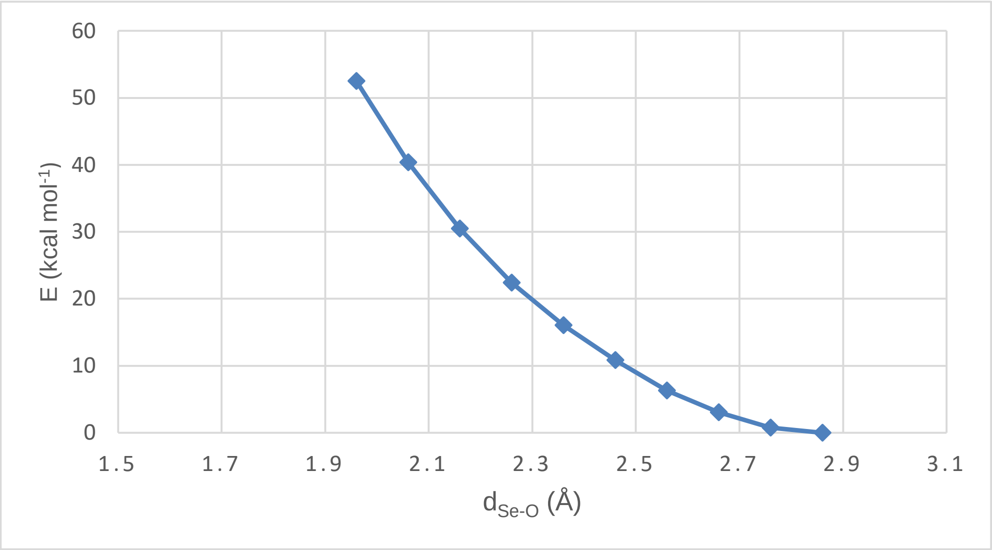

Supplementary Figure 4. Relaxed potential energy surface scan for the [1,2]-migration of PhO to the Se center at the B3LYP-D3 level (the mixed basis set is used: LANL2DZdp for Se, and 6-31g(d,p) for other atoms).

Attempts to locate a transition state for the [1,2]-migration of PhO to the Se center to generate the O–Se–N intermediate failed. As shown in Supplementary Figure 4, a relax scan of the migration of PhO group to the Se atom indicates that the energy increases monotonously as the Se–O(Ph) distance decreases from 2.86 to 1.96 Å. This result implies that the intermediate **INT1′-Se** can’t be generated through this pathway.

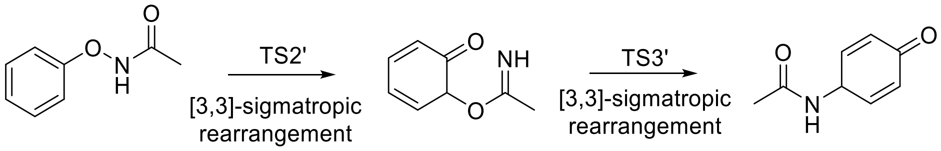

1a **INT5** 4a'

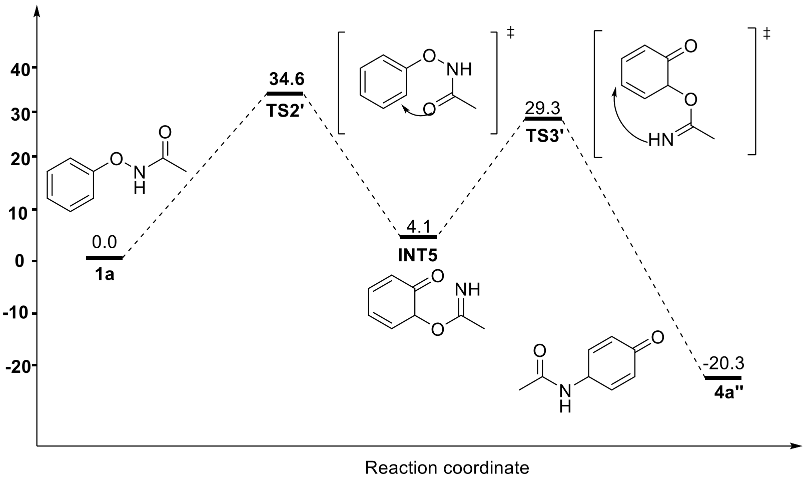

Supplementary Figure 5. Calculated Gibbs energy (in kcal mol^{-1}) profile for the generation of the *p*-amination phenol *via* a [3,3]-sigmatropic rearrangement pathway.

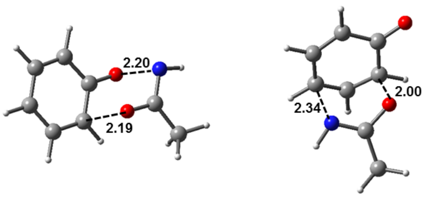

TS2’ TS3’

Supplementary Figure 6. Transition state for [3,3]-sigmatropic rearrangement pathway.

The possibility for the formation of **2a’** from **1a** *via* [3,3]-sigmatropic rearrangement is also calculated. As shown in Figure 5, the rate-determining-step for the generation of **2a’** is **TS2’** (with a barrier of 34.6 kcal/mol), which is about 17.5 kcal/mol higher than **TS2**, leading to the same **2a’**. The high activation energy of **TS2’** indicated that this pathway is unfavorable.

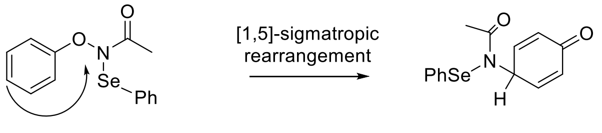

INT1-Se **INT3-Se**

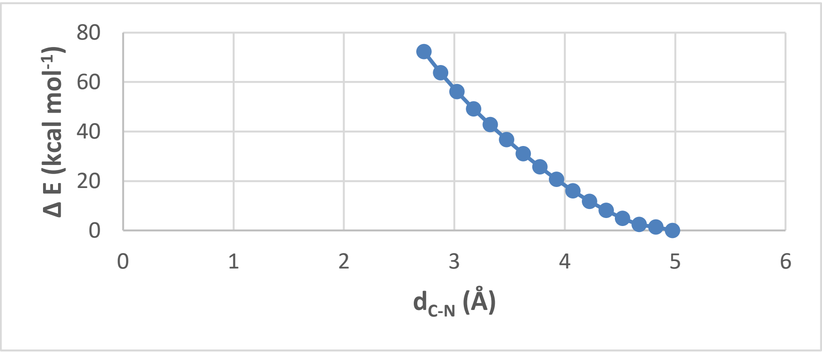

Supplementary Figure 7. Relaxed potential energy surface scan for the generation of the *p*-amination phenol via [1,5]-migration of **INT1-Se** at the B3LYP-D3 level (the mixed basis set is used: LANL2DZdp for Se, and 6-31g(d,p) for other atoms).

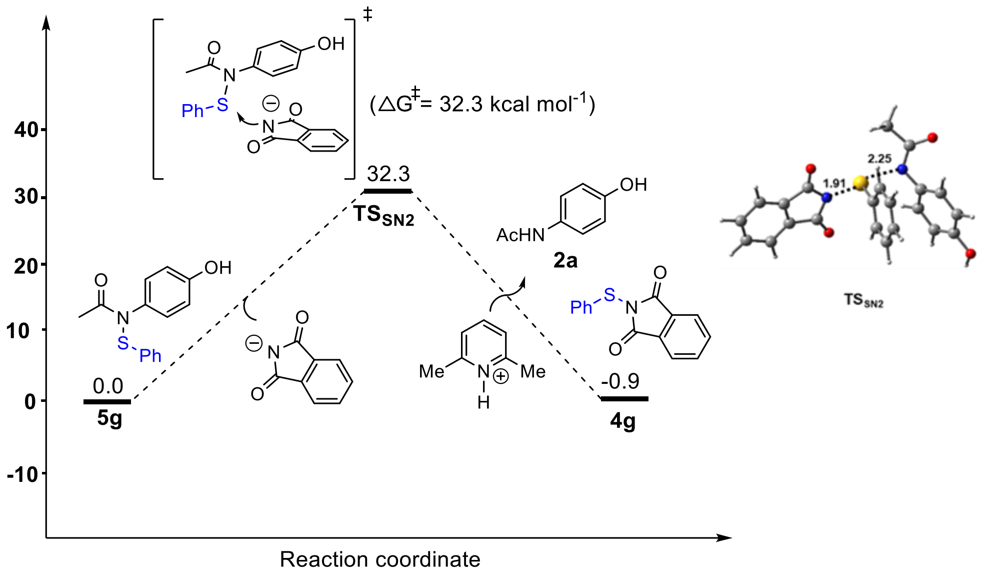

Supplementary Figure 8. Computed Gibbs energy (in kcal mol^{-1}) profile for the regeneration of **2g** from **3g** mediated by 2,6-lutidine in the solvent (TFE). All distances are given in Å.

The nucleophilic substitution by the phthalimide anion to **5g** may regenerate the *N*-phenylthiophthalimide **4g**, as shown in Supplementary Figure 8. However, the corresponding barrier of this process is up to 32.3 kcal mol^{-1}, and the regeneration of **4g** is slightly exothermic by 0.9 kcal mol^{-1}, suggesting the turnover of **4g** is difficult even under basic condition. Therefore, for S-mediated reaction, a stoichiometric amount of *N*-phenylthiophthalimide is required.

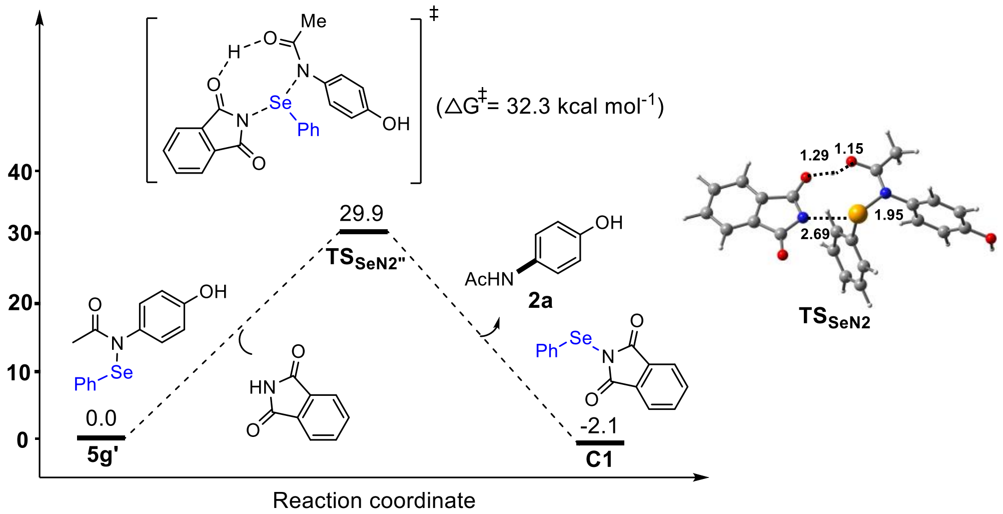

Supplementary Figure 9. Computed Gibbs energy (in kcal mol^{-1}) profile for the regeneration of **C1** from **5g'** in the solvent (TFE). All distances are given in in Å.

The nucleophilic substitution by the phthalimide to **5g'** may regenerate the *N*-phenylthiophthalimide, as shown in Supplementary Figure 9. However, the corresponding barrier of this process is 29.9 kcal mol-1, and the regeneration of *N*-phenylselanylphthalimide (**C1**) is exothermic by 2.1 kcal mol-1, suggesting the turnover of *N*-phenylselanylphthalimide (**C1**) is feasible under neutral condition. Therefore, *N*-phenylselanylphthalimide might be a possible catalyst for the *para*-selective nitrogen migration of *N*-aryloxyacetamides, which has a lower regeneration barrier.

Attempts to locate a transition state for the generation of the *p*-amination phenol via [1,5]-migration of **INT1-Se** failed. As shown in Supplementary Figure 9, a relax scan of the migration of N(SePh)Ac group to the *para*-position of **INT1-Se** indicates that the energy increases monotonously as the C–N distance decreases from 4.97 to 2.72 Å. This result implies that the intermediate **INT3-Se** can't be generated through this pathway.

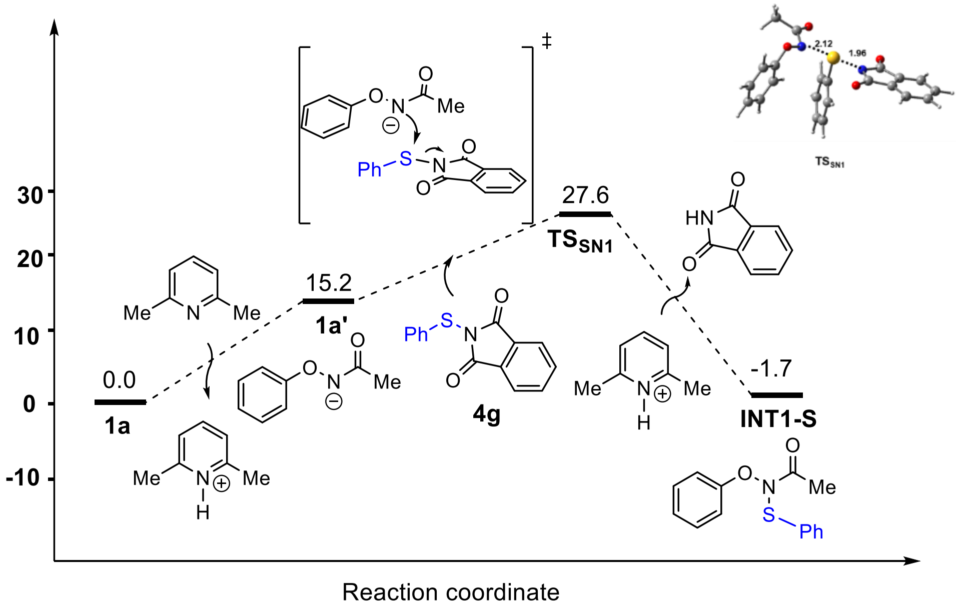

Supplementary Figure 10. Computed Gibbs energy (in kcal mol^{-1}) profile for the reaction between **1a** and **4g** to generate the reactive intermediate **INT1-S** mediated by 2,6-lutidine in TFE. All distances are given in in Å.

For S-mediated reaction, the mechanistic details for the generation of **INT1-S** is investigated, the calculated free energy is shown in Supplementary Figure 10. First, the deprotonation of **1a** by 2,6-lutidine generates a nitrogen anionic species **1a′**. Then, the nucleophilic attack of **1a'** to *N*-phenylthiophthalimide **4g** via **TS$_{SN1}$** generates **INT1-S**. This process is exothermic by 1.7 kcal mol^{-1}, which a barrier of 27.6 kcal mol^{-1}. This result suggests that the formation of **INT1-S** using phenoxyacetamide (**1a**) and *N*-phenylthiophthalimide (**4g**) in the present of 2,6-lutidine is possible.

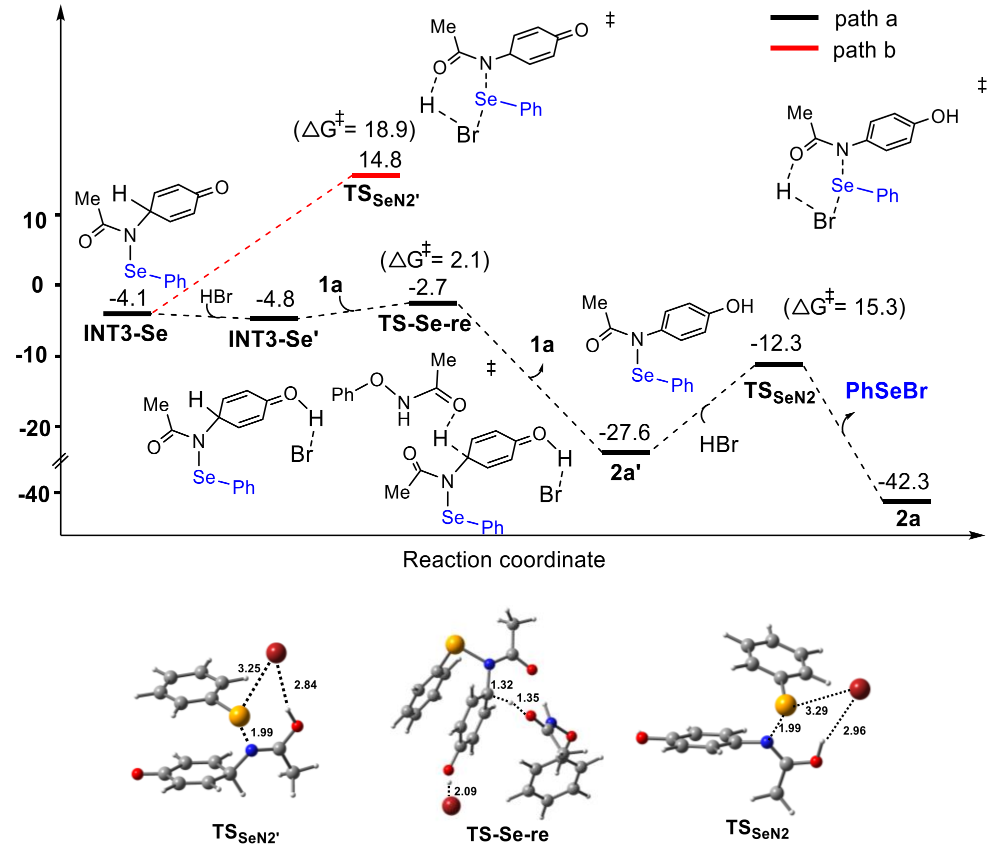

Supplementary Figure 11. Calculated Gibbs energy (in kcal mol^{-1}) profile for the regeneration of catalyst PhSeBr.

For Se-catalyzed reaction, the free energy profile for the regeneration of catalyst PhSeBr from **2a′** is also investigated. As show in Fig. 11, the rearomatization of **INT3-Se** with the assistance of HBr and **1a** generates the intermediate **2a′** via transition state **TS-Se-re**, the activation barrier of this process is only 2.1 kcal mol^{-1} (**path-a**). Then, in the protonolysis of **2a′** to regenerate the catalyst PhSeBr, the corresponding barrier is only 15.3 kcal mol^{-1}. The regeneration of PhSeBr is strongly exothermic by 14.7 kcal mol^{-1}, suggesting the turnover of PhSeBr is feasible. This calculation is consistent with our experimental results. While the regeneration of PhSeBr *via* the direct protonolysis of **INT3-Se** (**TS$_{\text{SeN2'}}$**) is possible (**path-b**), the activation barrier of this pathway (18.9 kcal mol^{-1}) is higher than that of **path-a** (15.3 kcal mol^{-1}). Thus, our data suggest the aromatization step precede the Se–N bond protonolysis.

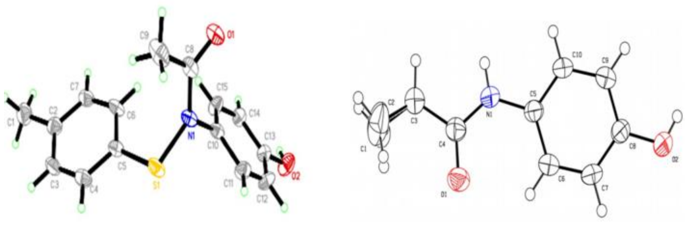

5a 2n

Supplementary Figure 12. X-ray diffraction structures of compound **2n** (CCDC-1549814) and **5a** (CCDC-1570955).

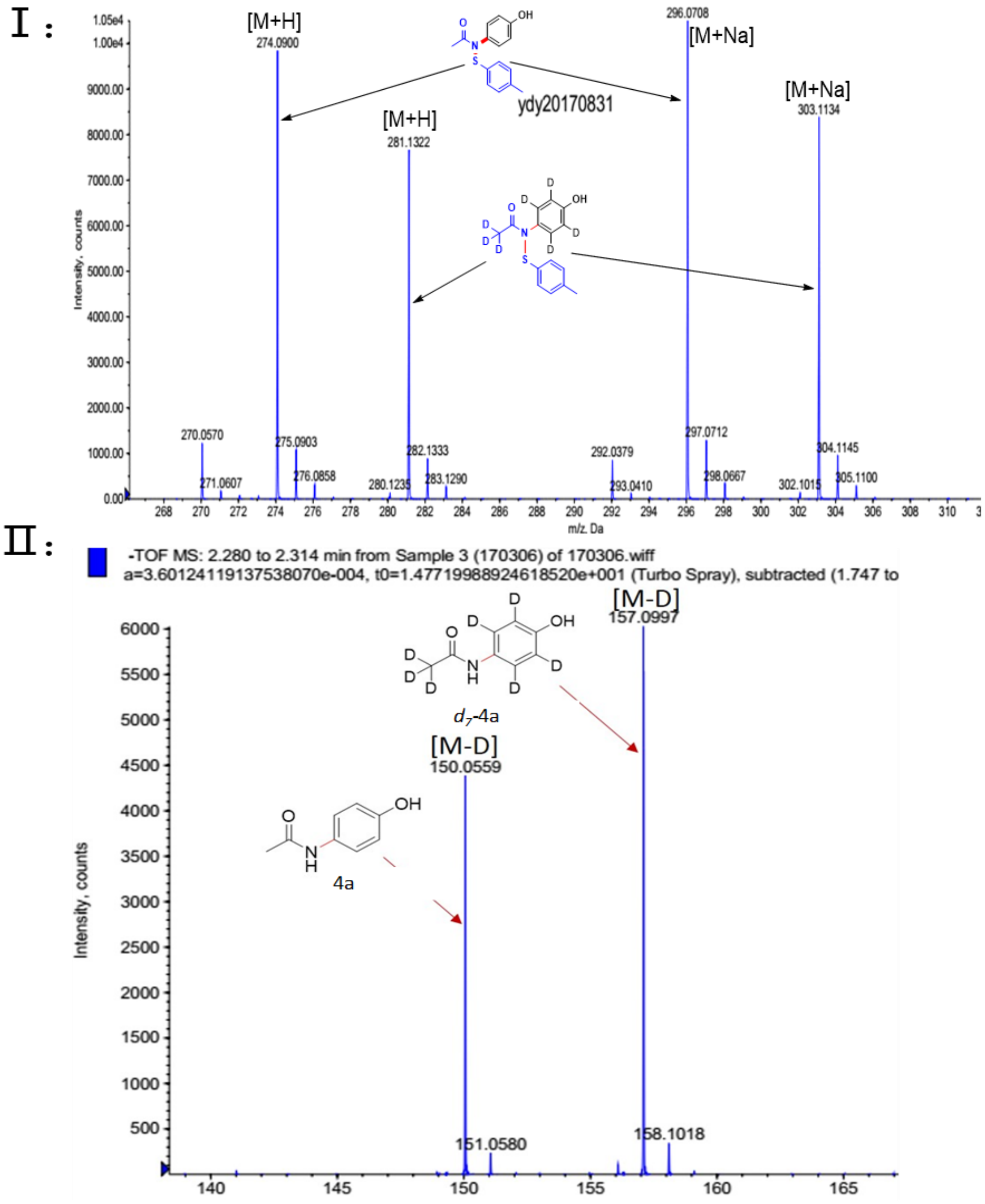

Supplementary Figure 13. Crossover experiment of S/Se-mediated reactions detected by HRMS.

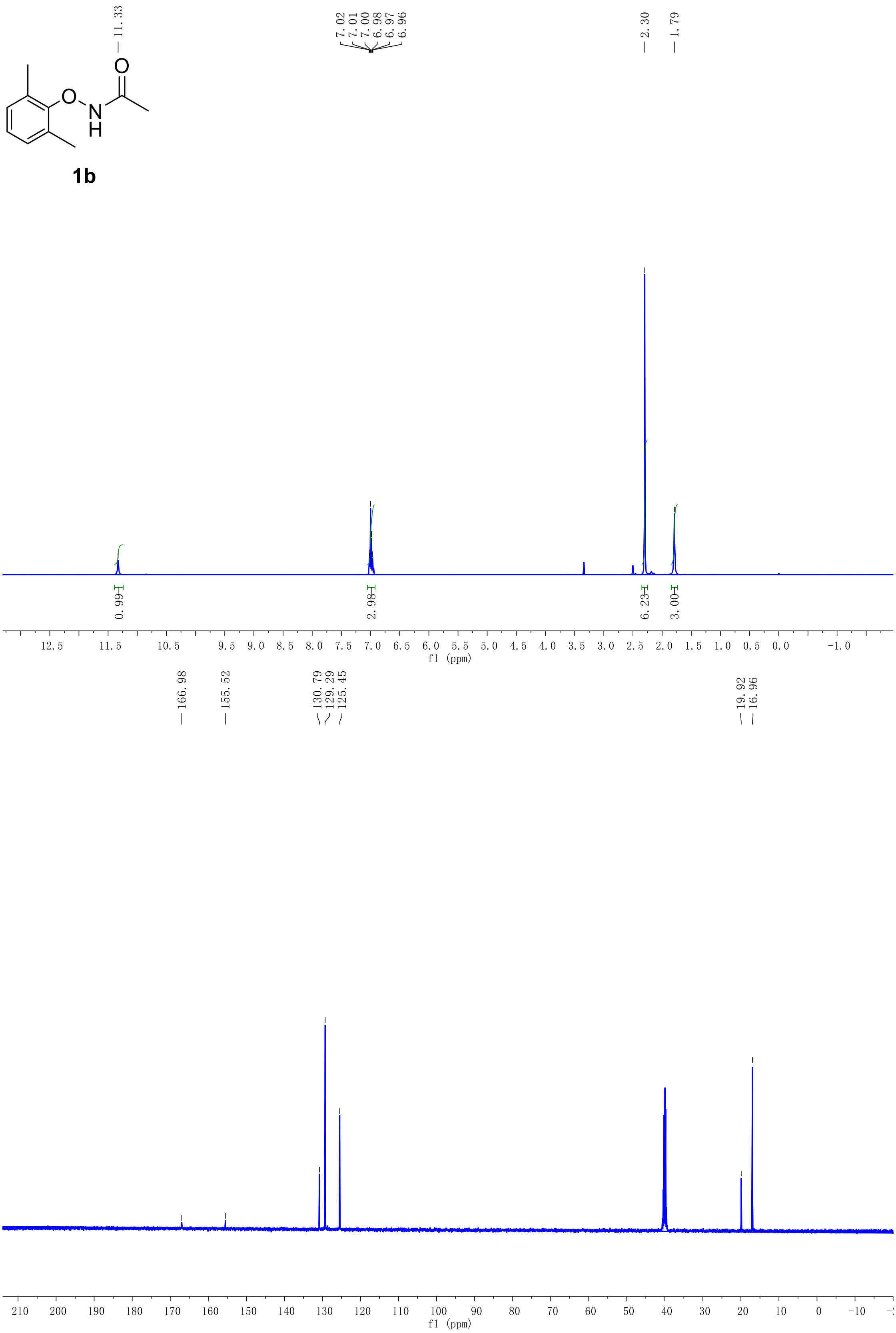

Supplementary Figure 14. ^{1}H and ^{13}C NMR spectra for compound **1b**

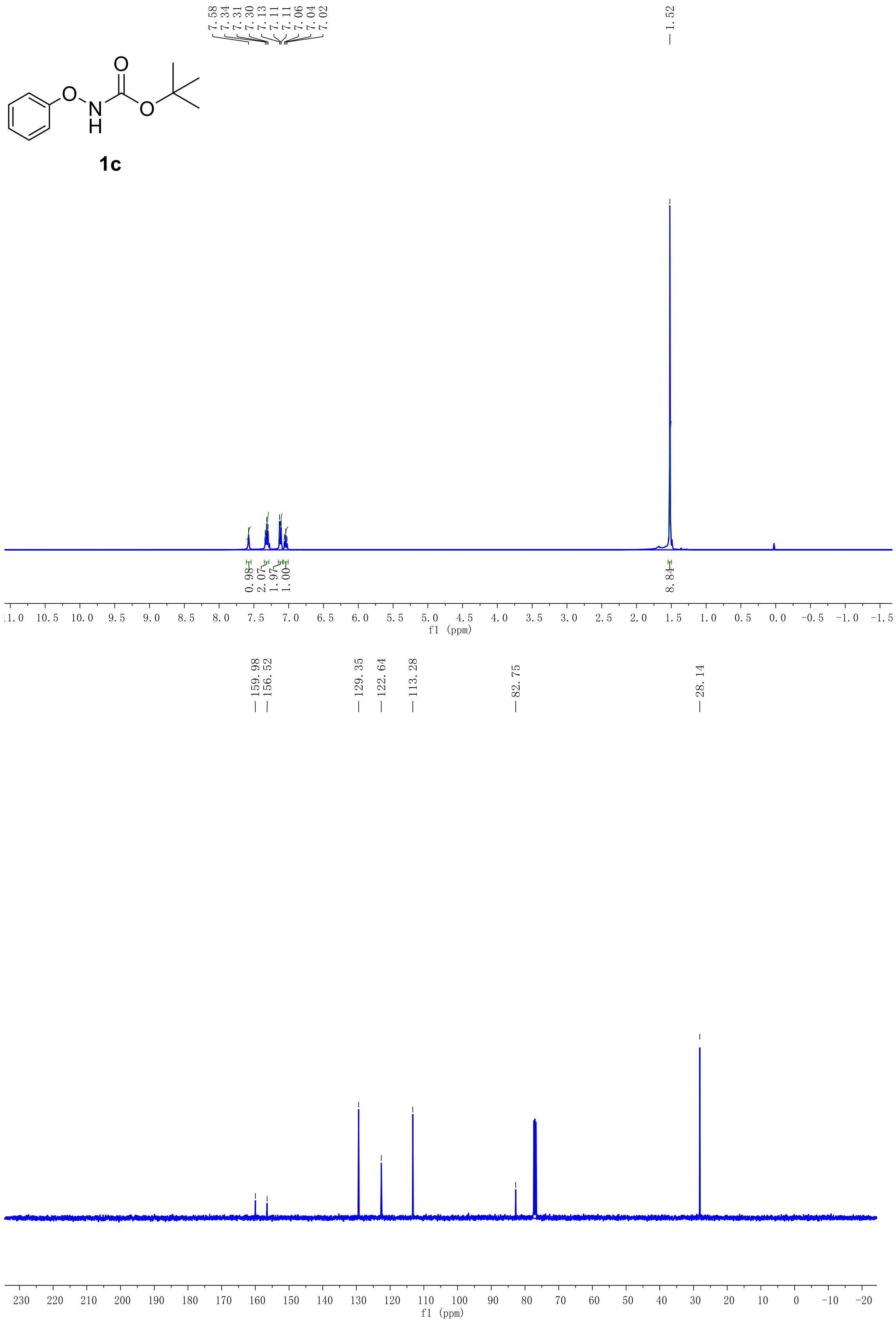

Supplementary Figure 15. ^{1}H and ^{13}C NMR spectra for compound **1c**

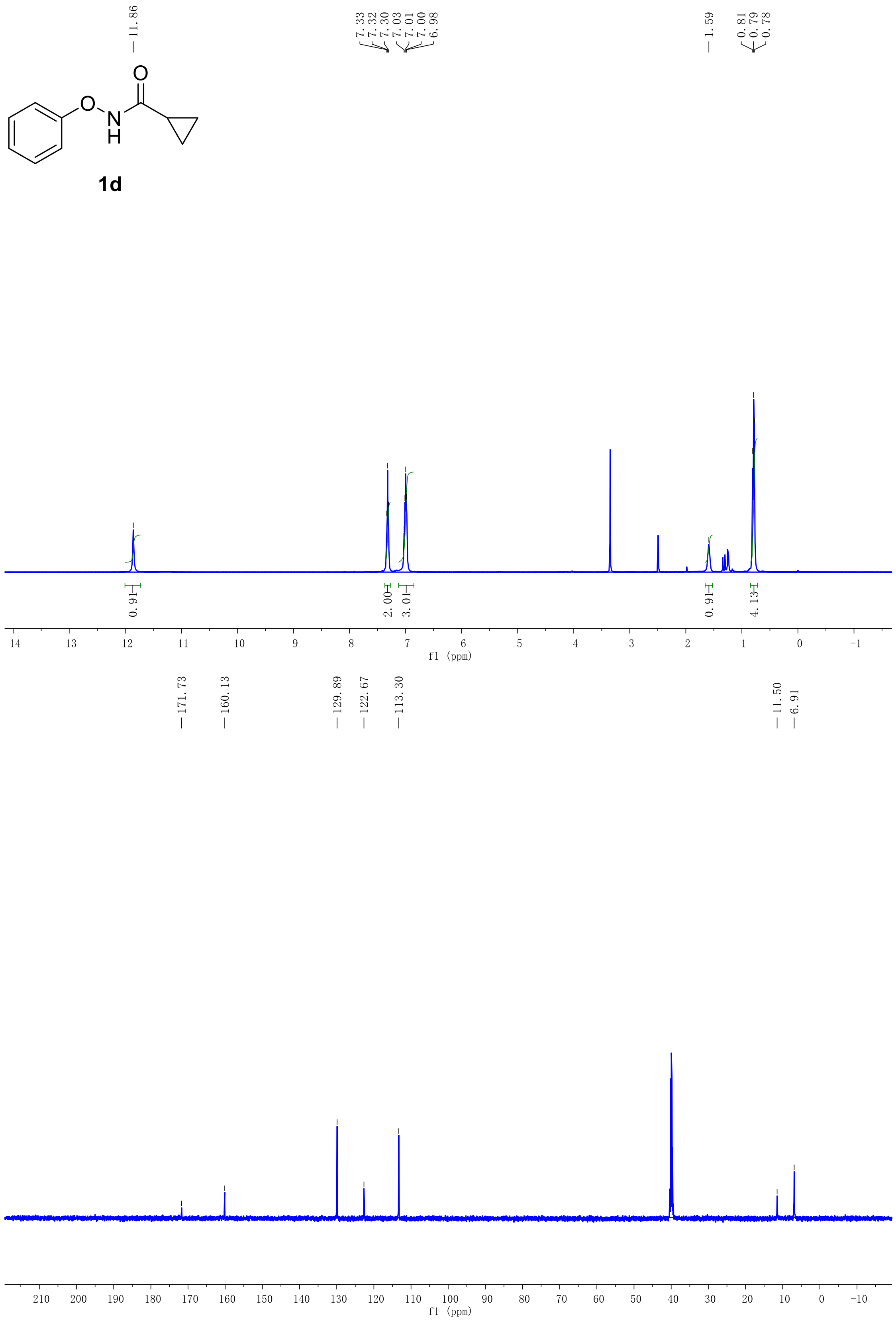

Supplementary Figure 16. ^{1}H and ^{13}C NMR spectra for compound **1d**

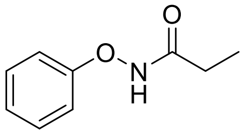

1e

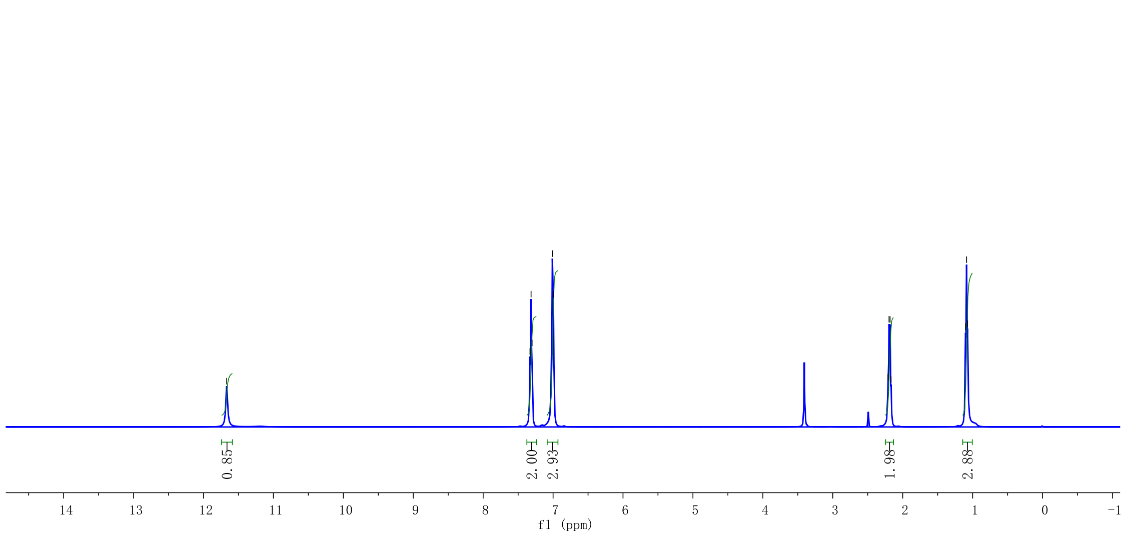

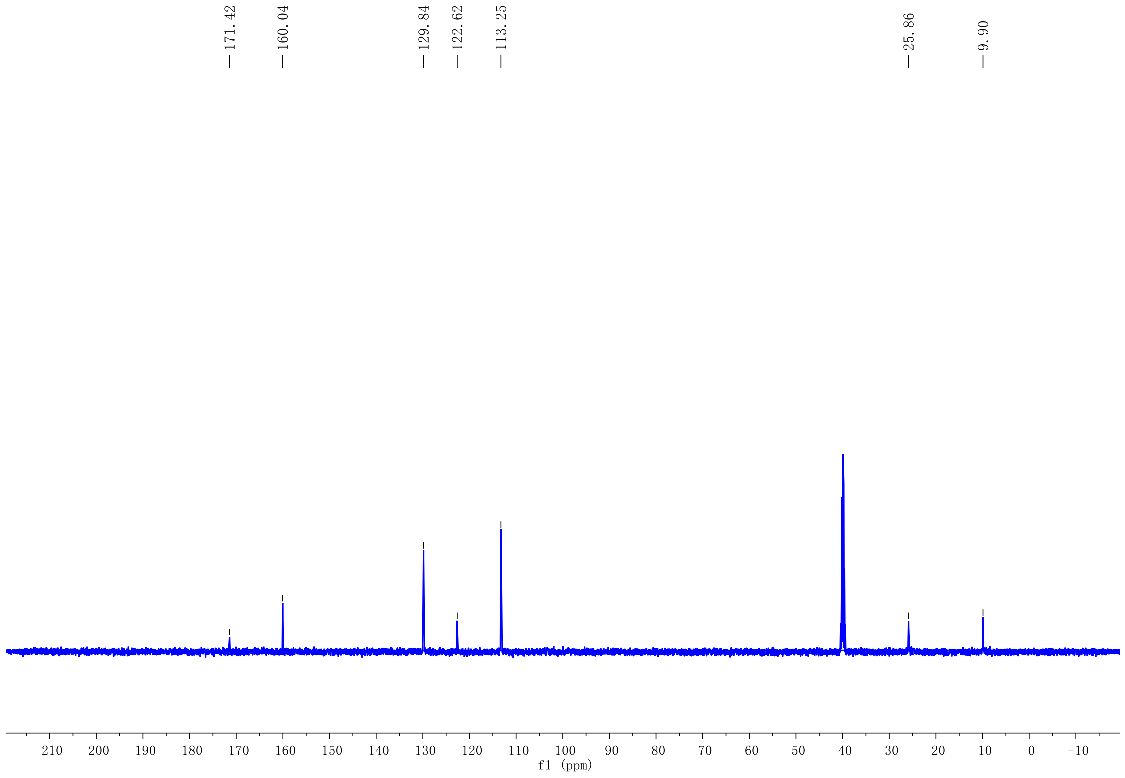

Supplementary Figure 17. ^{1}H and ^{13}C NMR spectra for compound **1e**

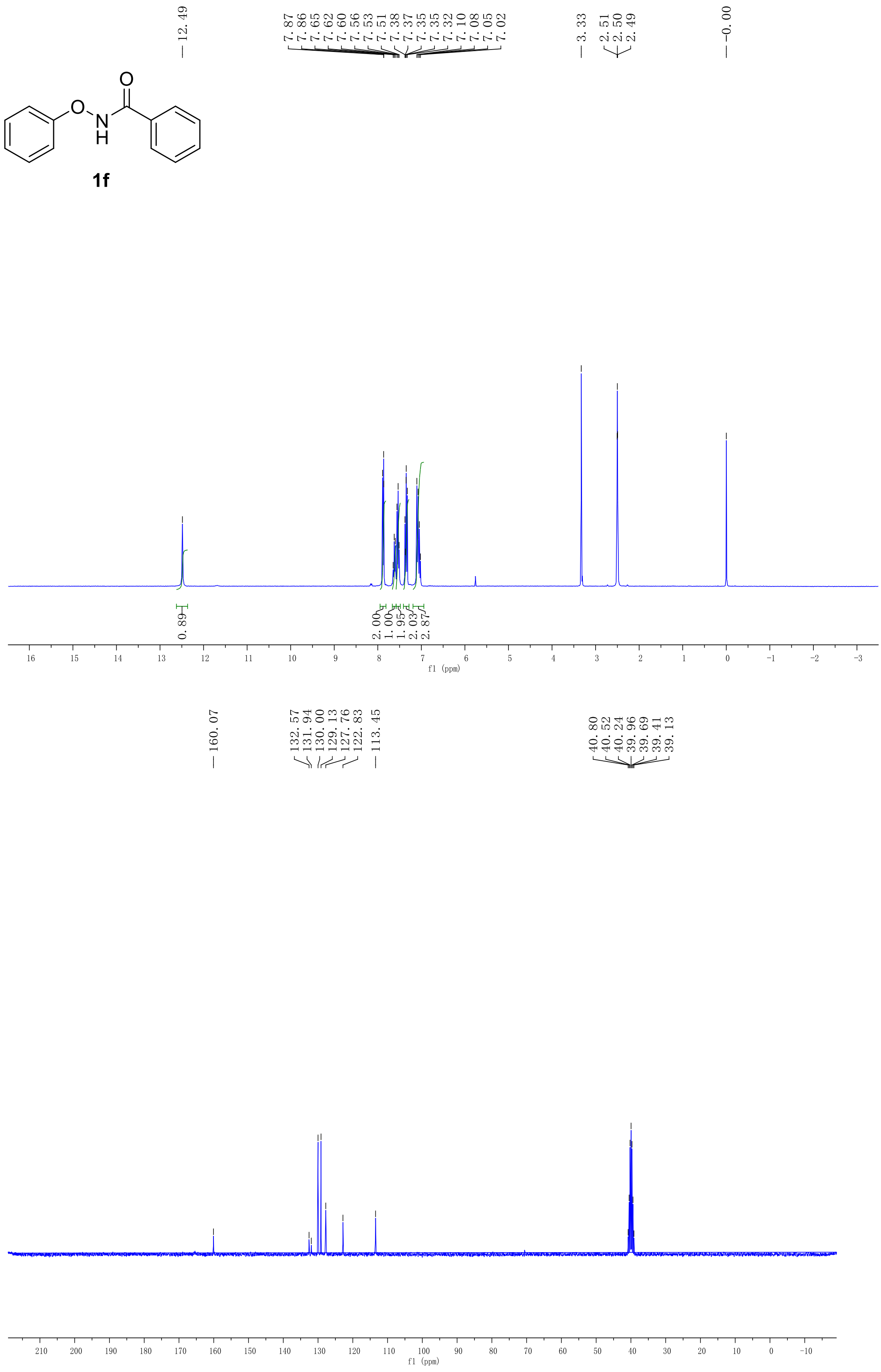

Supplementary Figure 18. ^{1}H and ^{13}C NMR spectra for compound **1f**

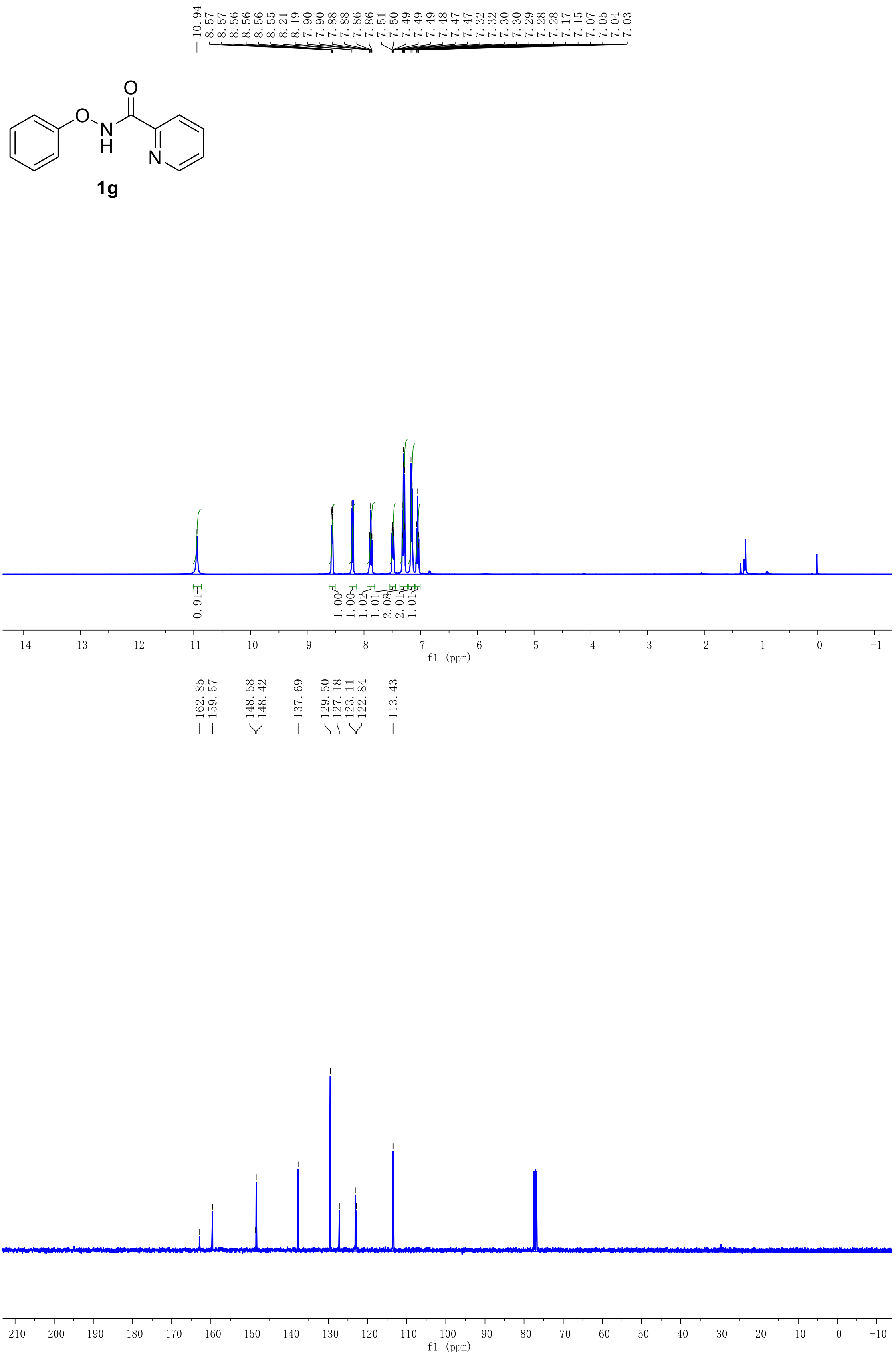

Supplementary Figure 19. ^{1}H and ^{13}C NMR spectra for compound **1g**

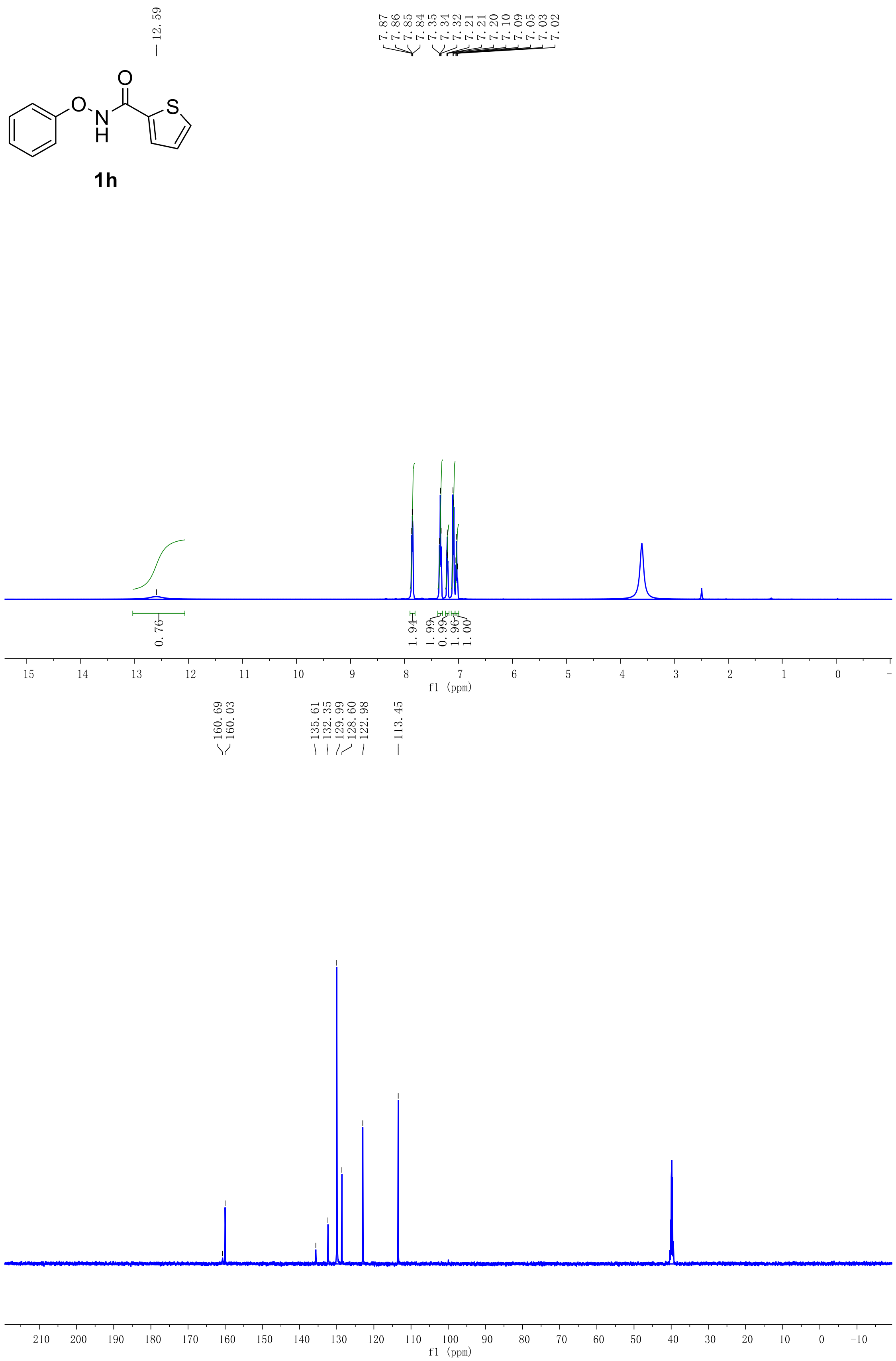

Supplementary Figure 20. ^{1}H and ^{13}C NMR spectra for compound **1h**

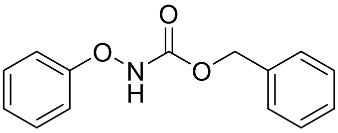

1i

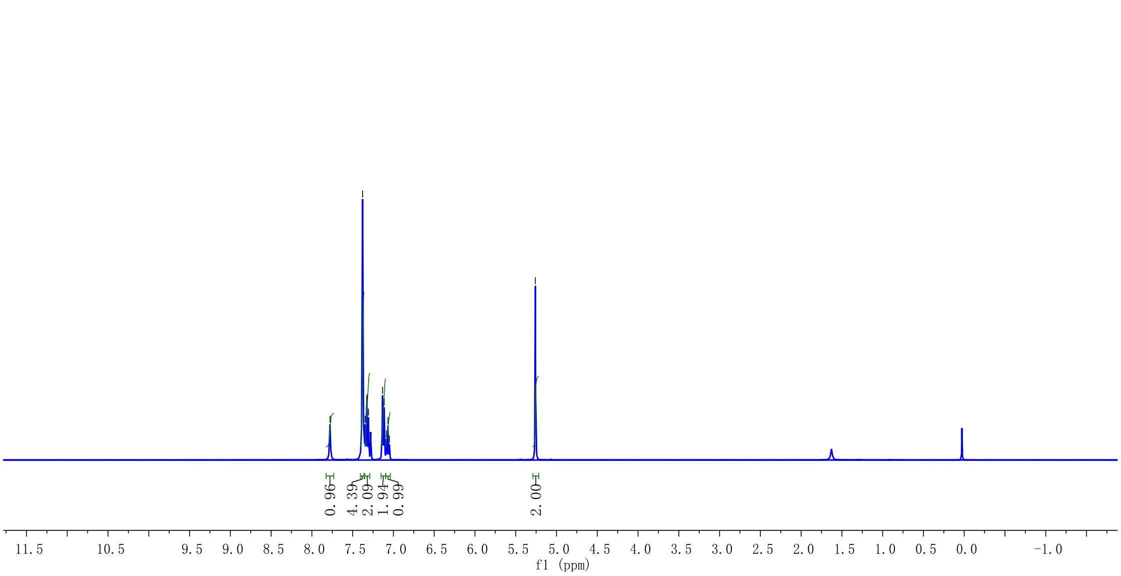

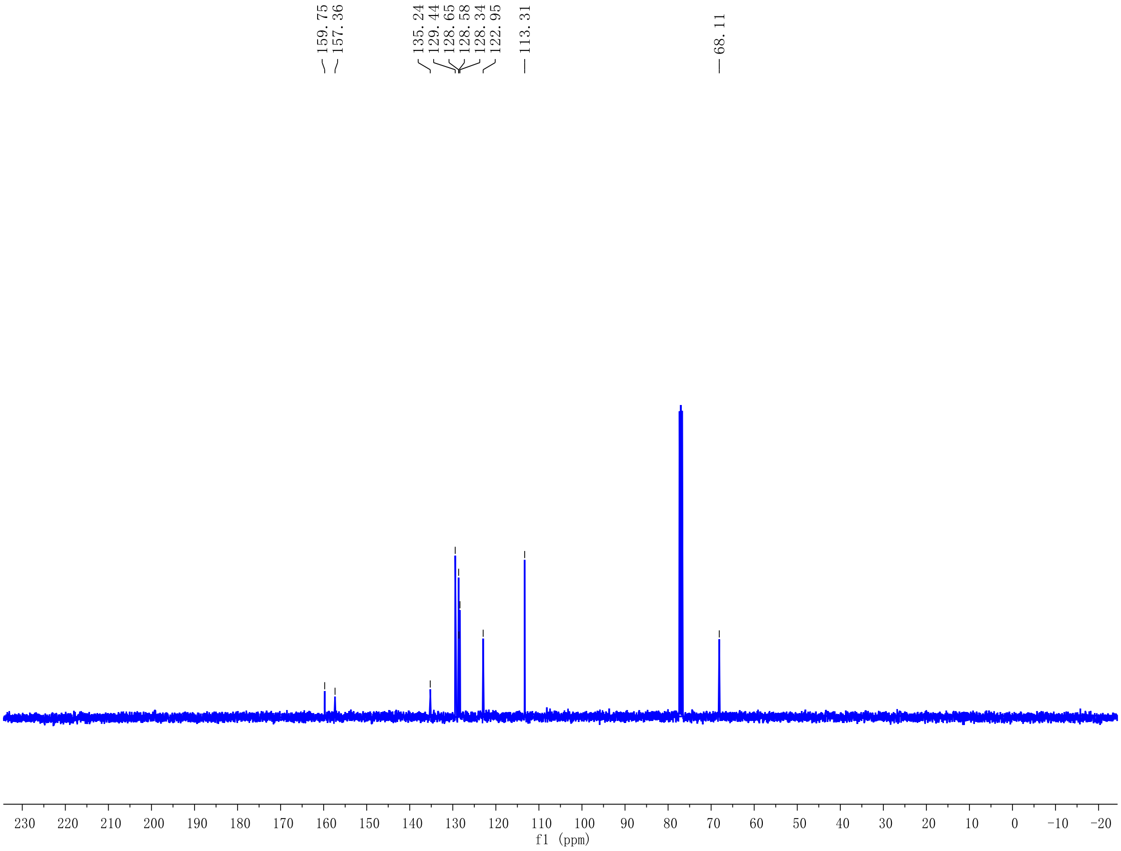

Supplementary Figure 21. ^{1}H and ^{13}C NMR spectra for compound **1i**

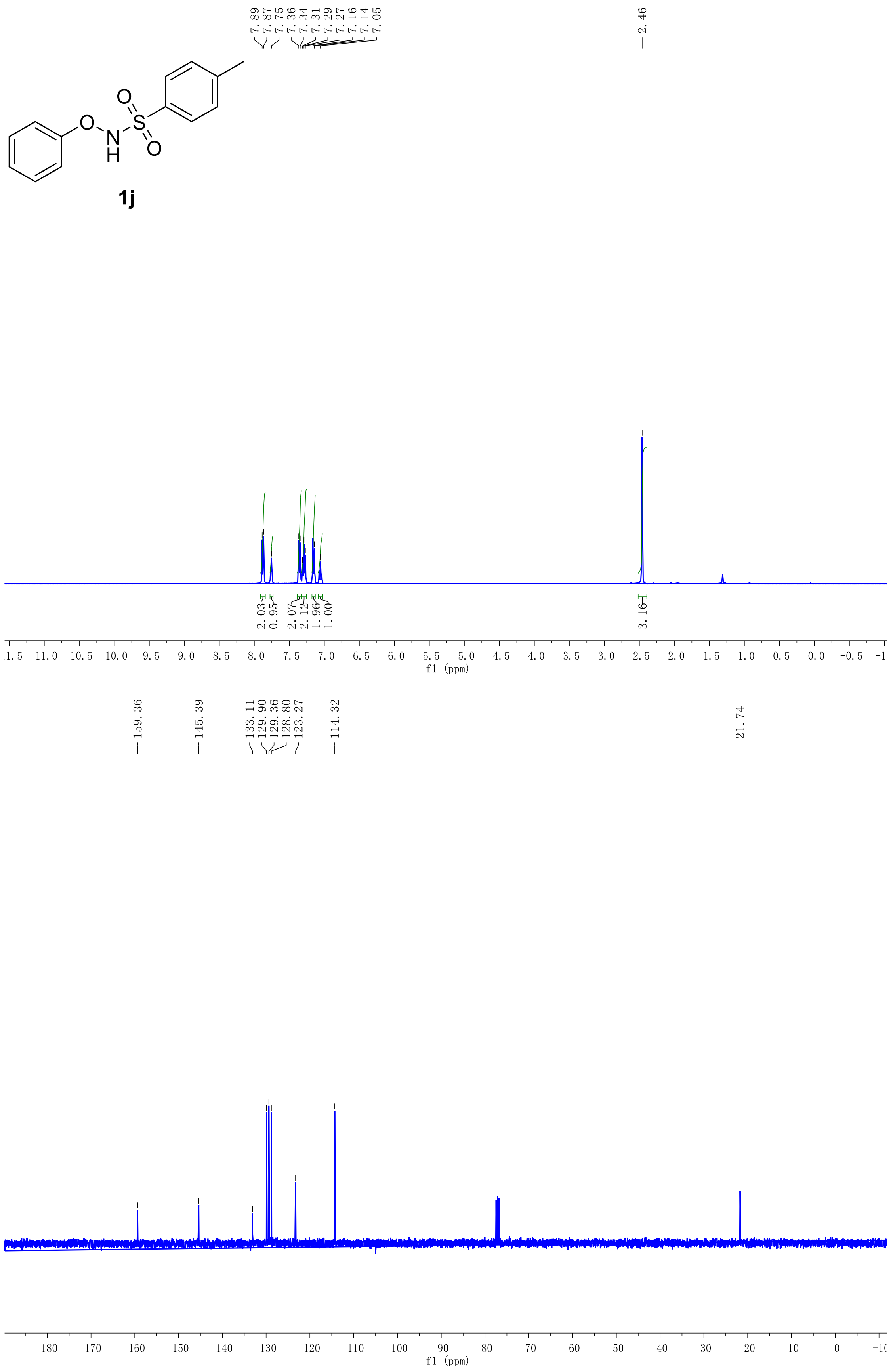

Supplementary Figure 22. ^{1}H and ^{13}C NMR spectra for compound **1j**

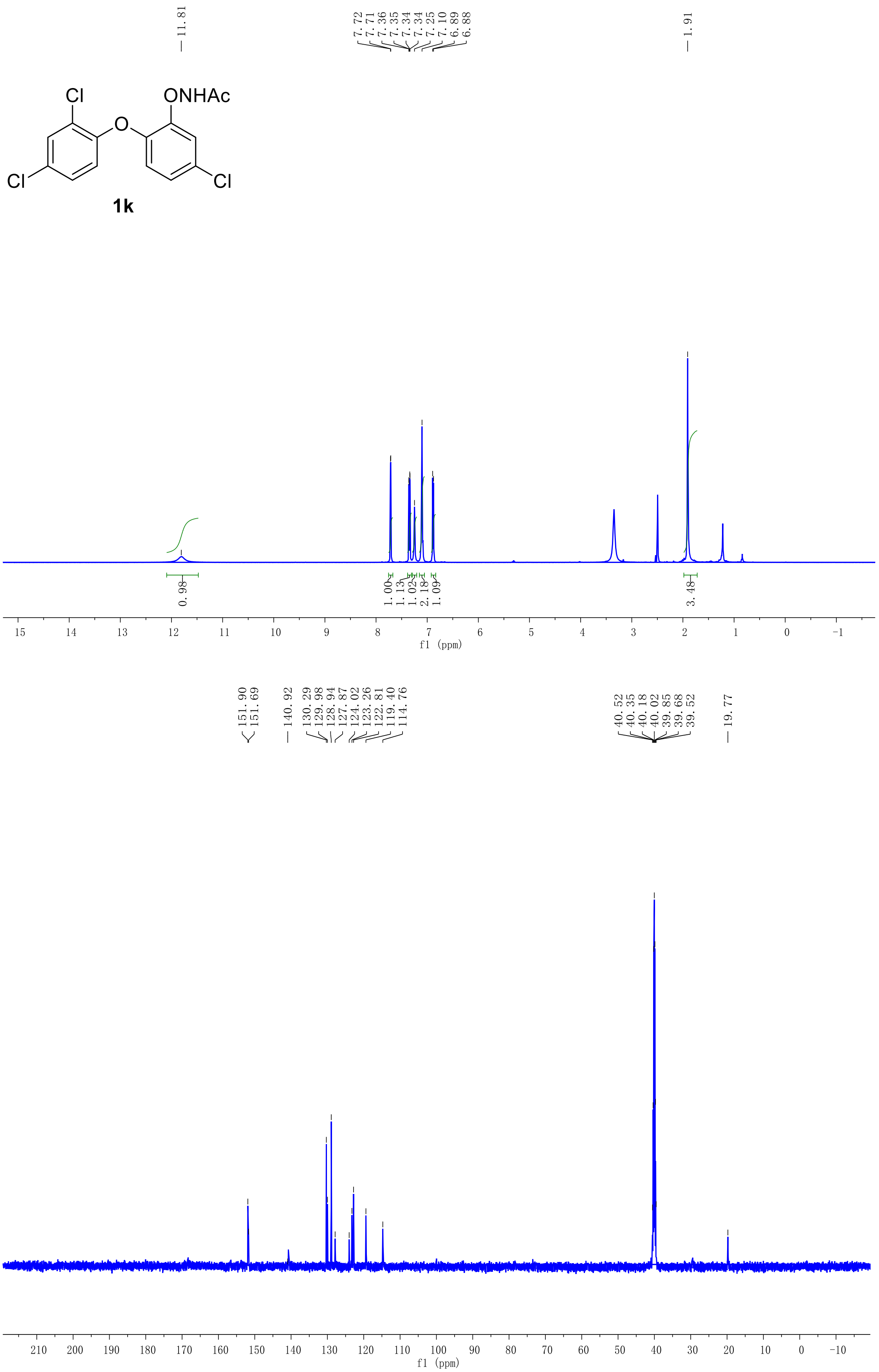

Supplementary Figure 23. ^{1}H and ^{13}C NMR spectra for compound **1k**

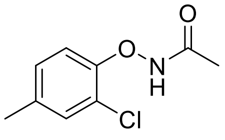

1l

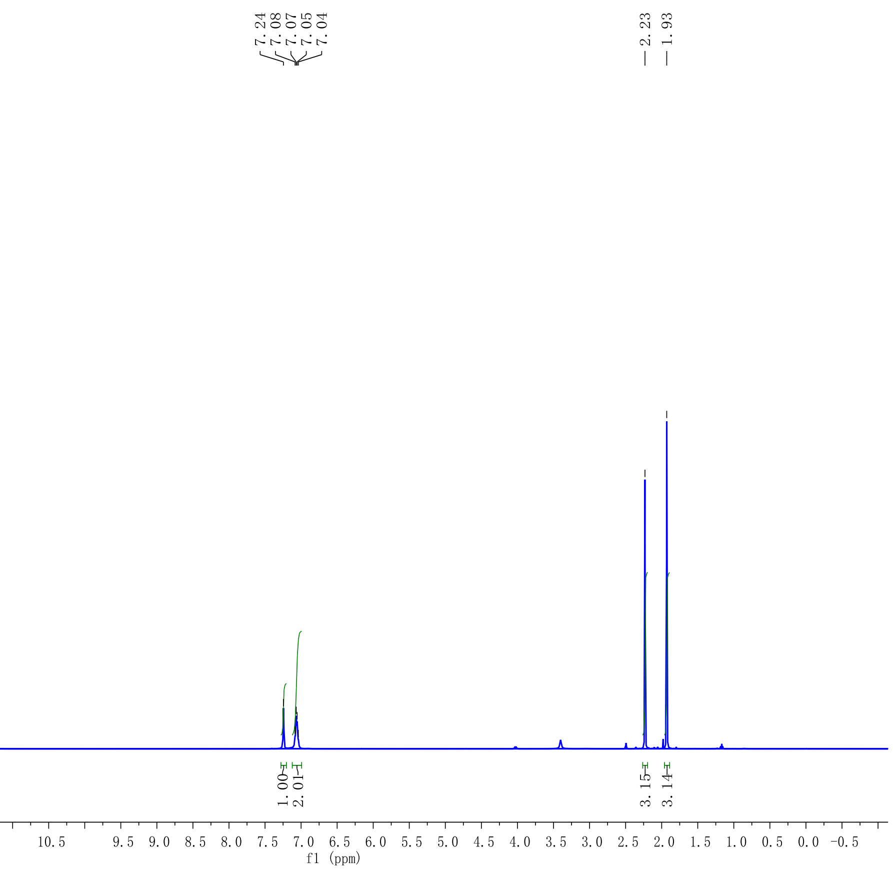

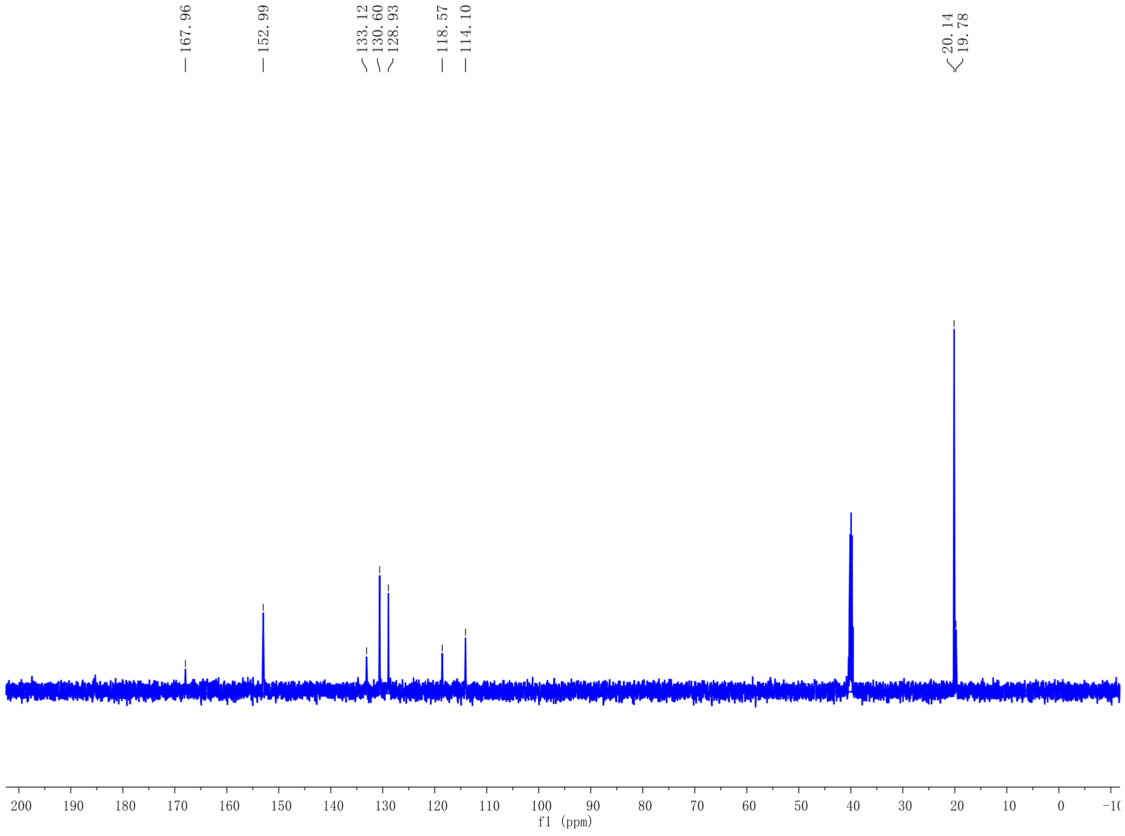

Supplementary Figure 24. ^{1}H and ^{13}C NMR spectra for compound **1l**

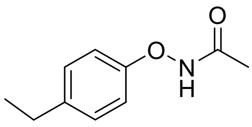

1m

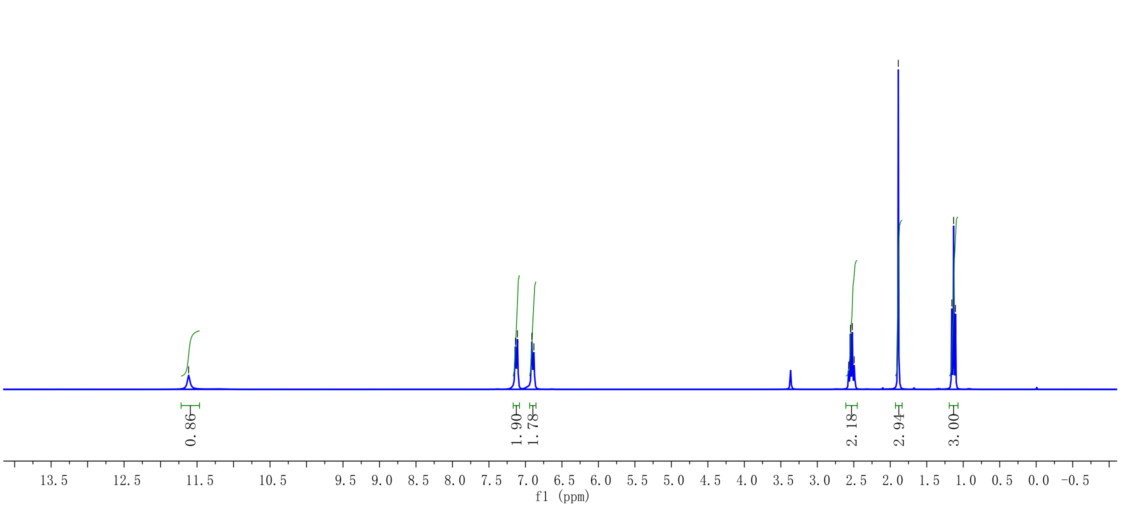

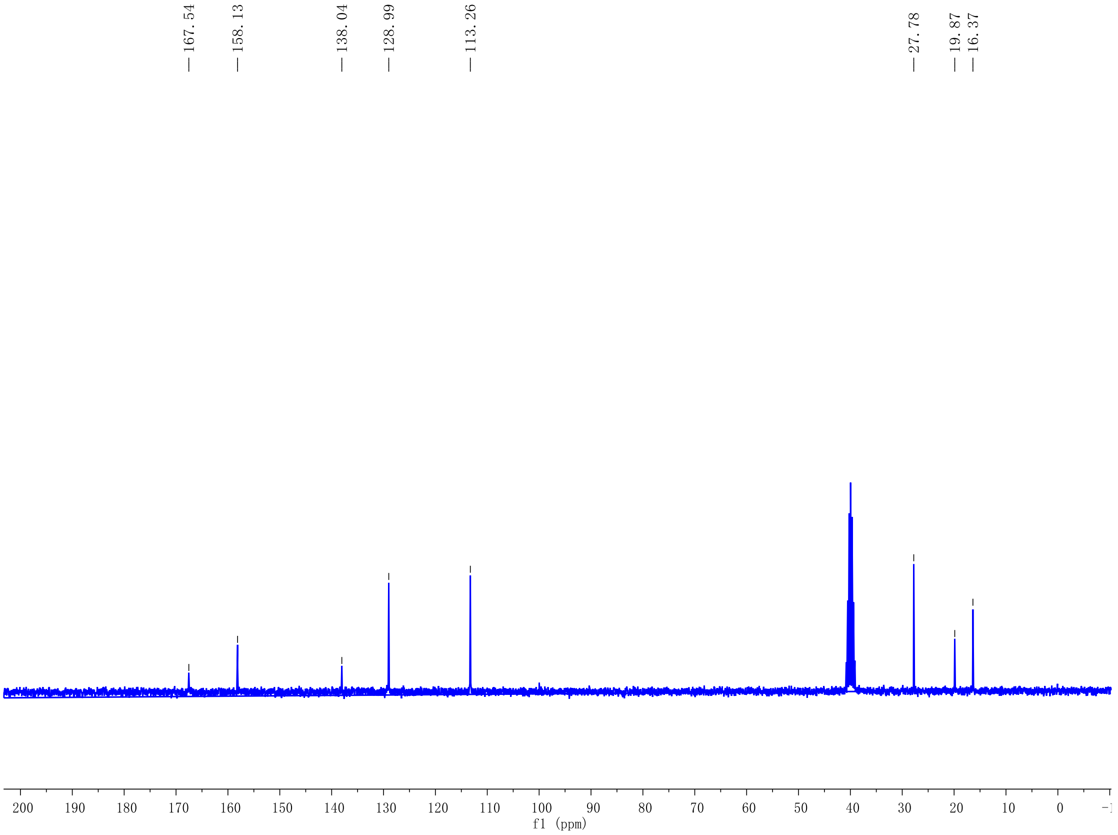

Supplementary Figure 25. ^{1}H and ^{13}C NMR spectra for compound **1m**

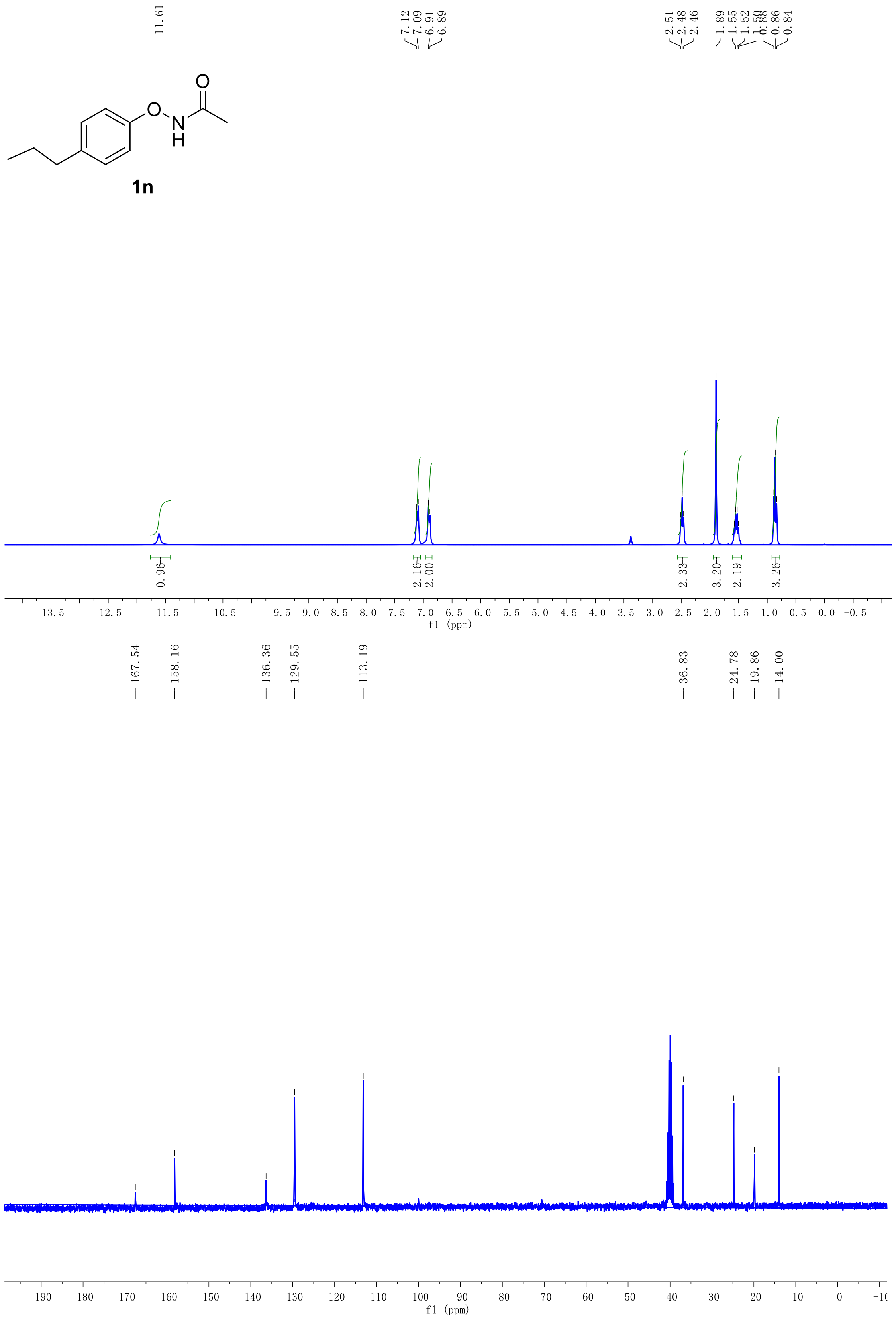

Supplementary Figure 26. ^{1}H and ^{13}C NMR spectra for compound **1n**

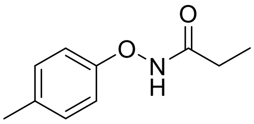

1o

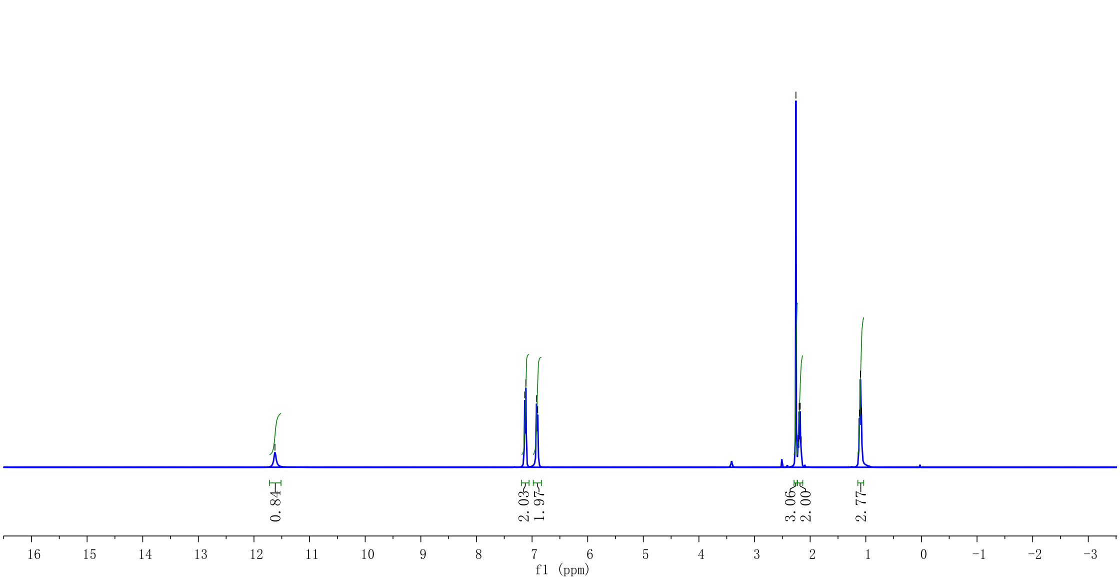

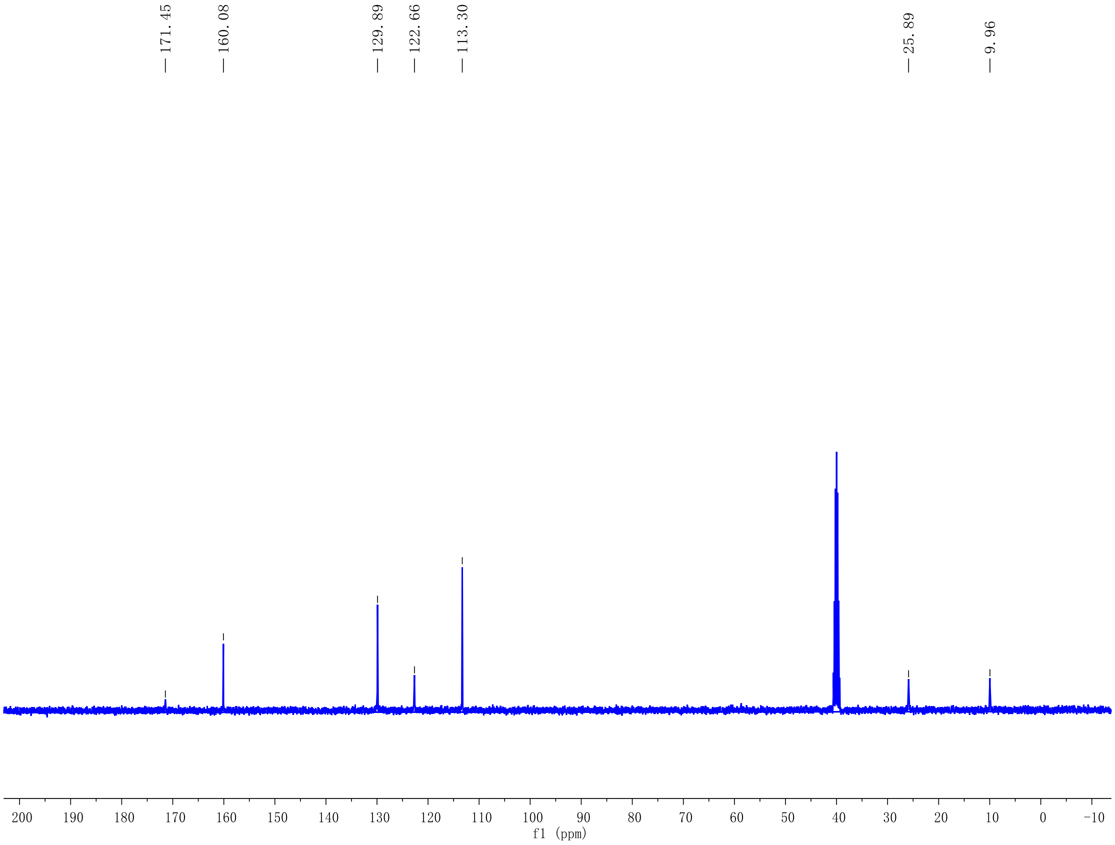

Supplementary Figure 27. ^{1}H and ^{13}C NMR spectra for compound **1o**

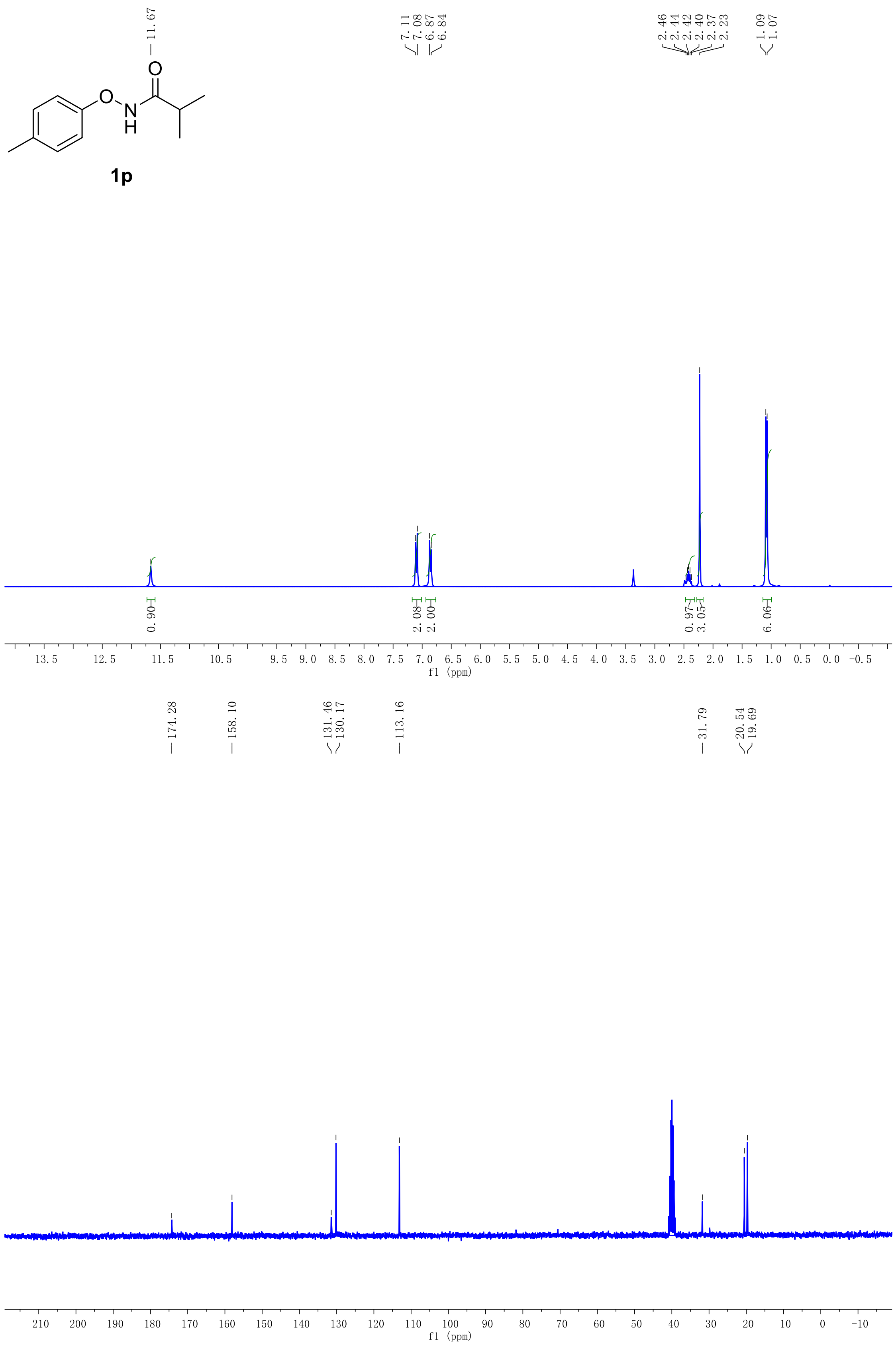

Supplementary Figure 28. ^{1}H and ^{13}C NMR spectra for compound **1p**

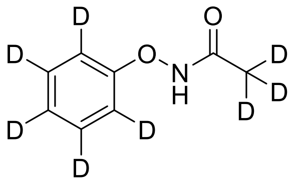

*d*₈-**1a**

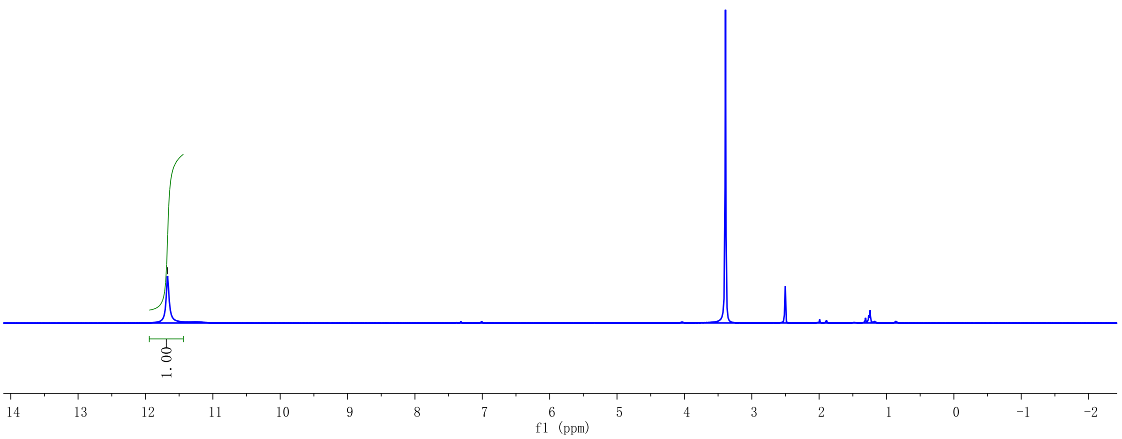

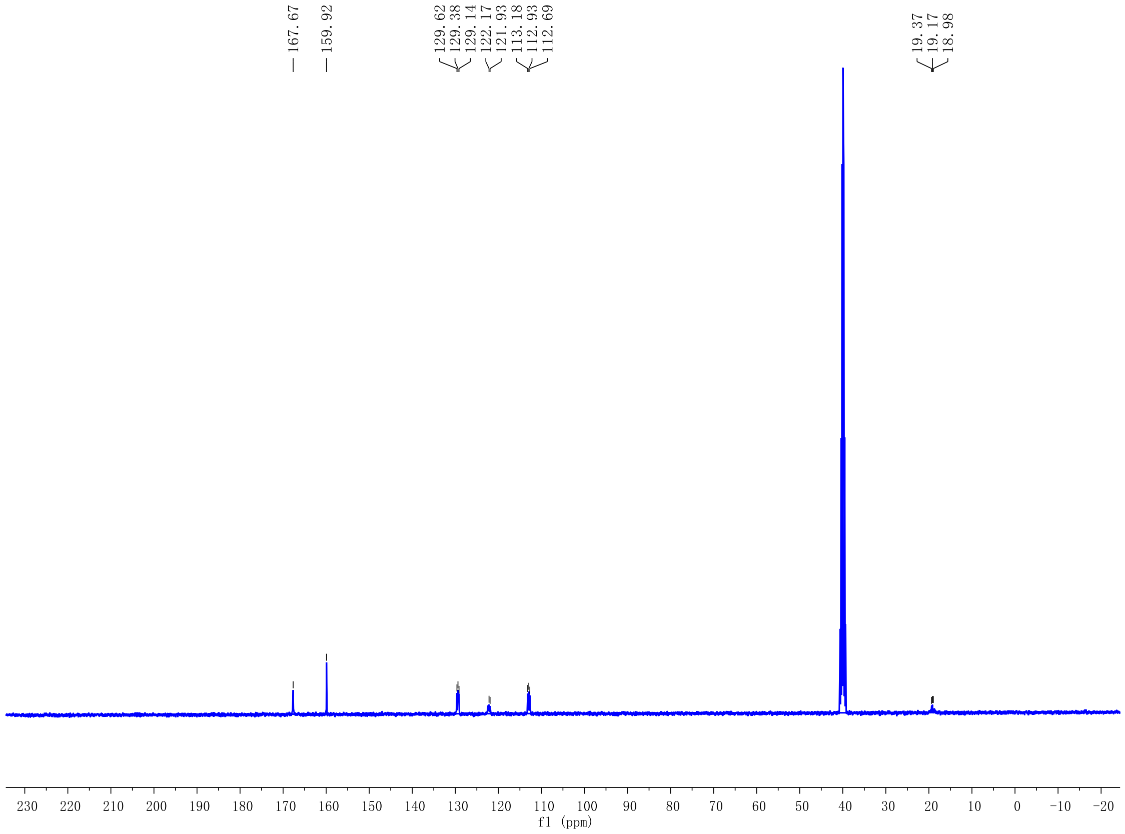

Supplementary Figure 29. ^{1}H and ^{13}C NMR spectra for compound *d*$_8$-**1a**

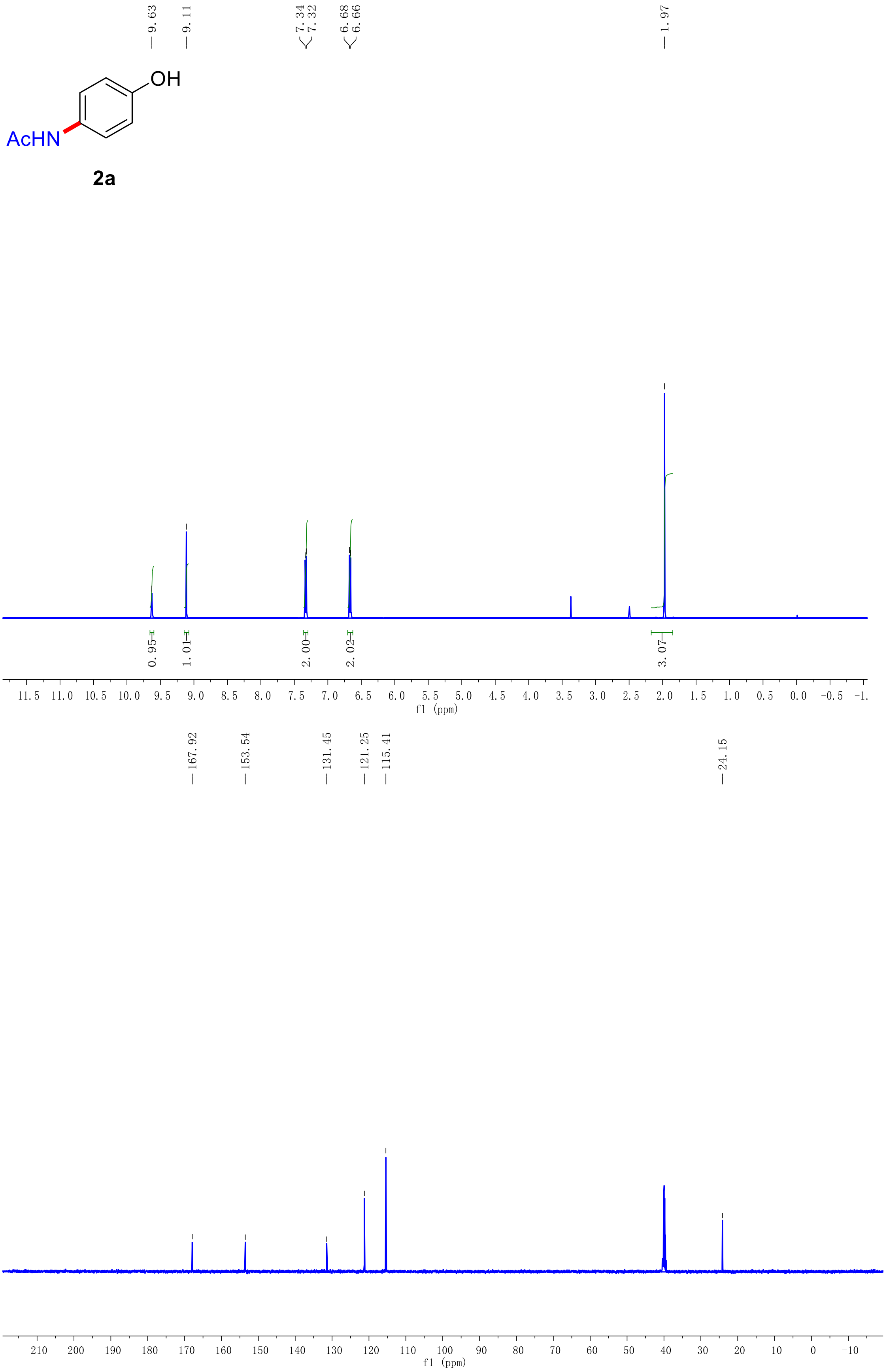

Supplementary Figure 30. ^{1}H and ^{13}C NMR spectra for compound **2a**

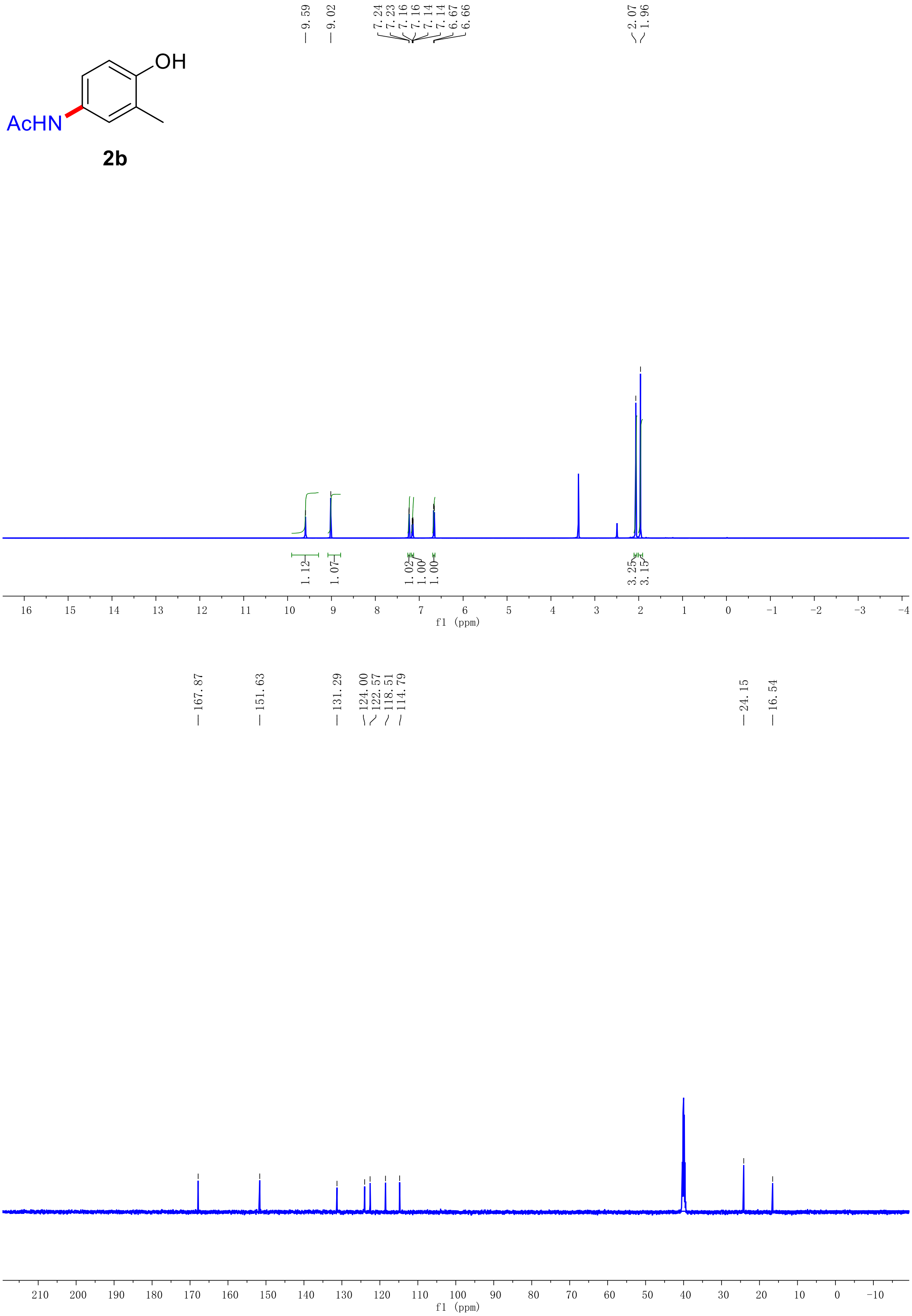

Supplementary Figure 31. ^{1}H and ^{13}C NMR spectra for compound **2b**

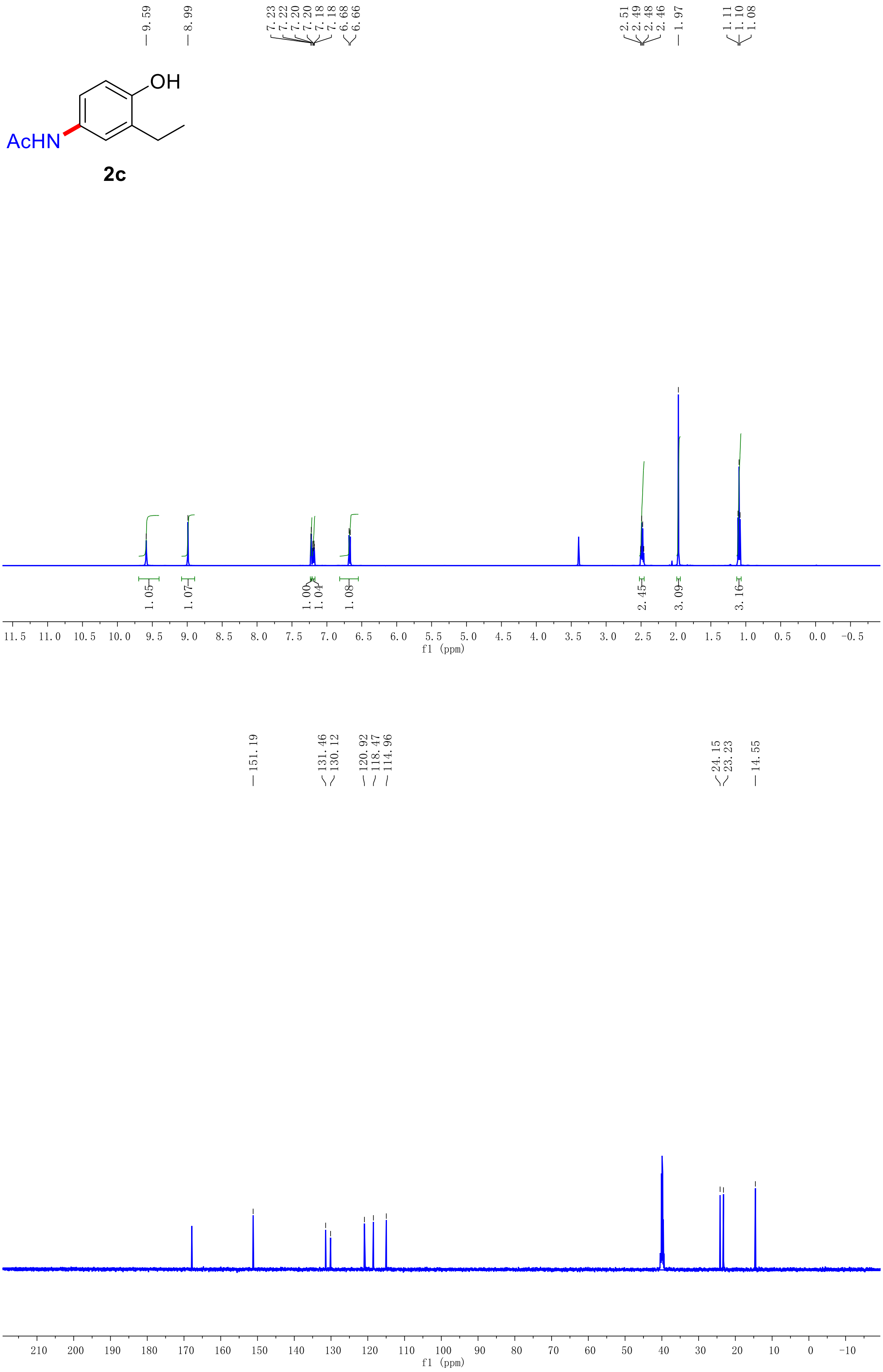

Supplementary Figure 32. ^{1}H and ^{13}C NMR spectra for compound **2c**

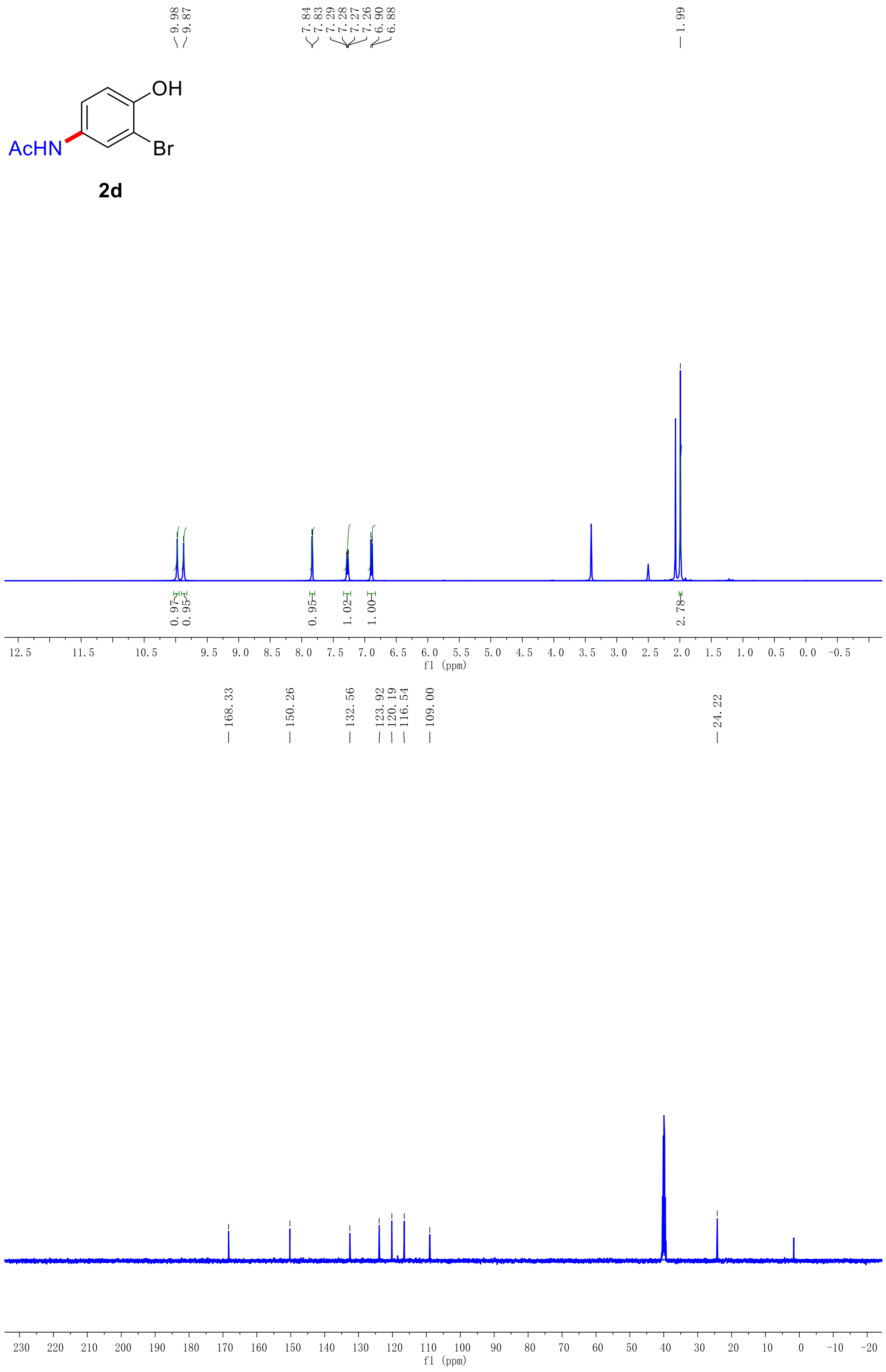

Supplementary Figure 33. ^{1}H and ^{13}C NMR spectra for compound **2d**

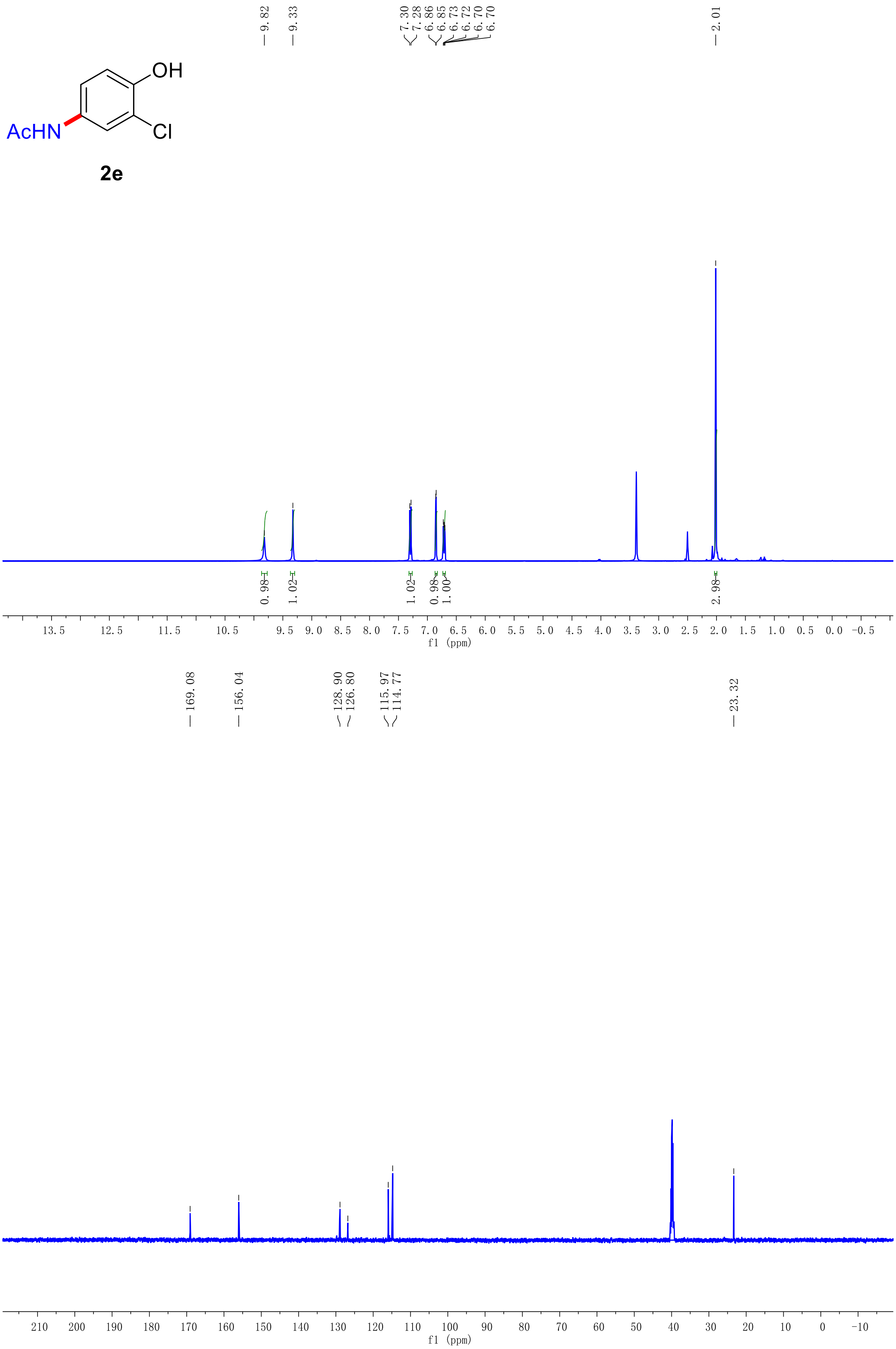

Supplementary Figure 34. ^{1}H and ^{13}C NMR spectra for compound **2e**

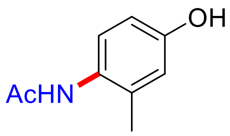

2f

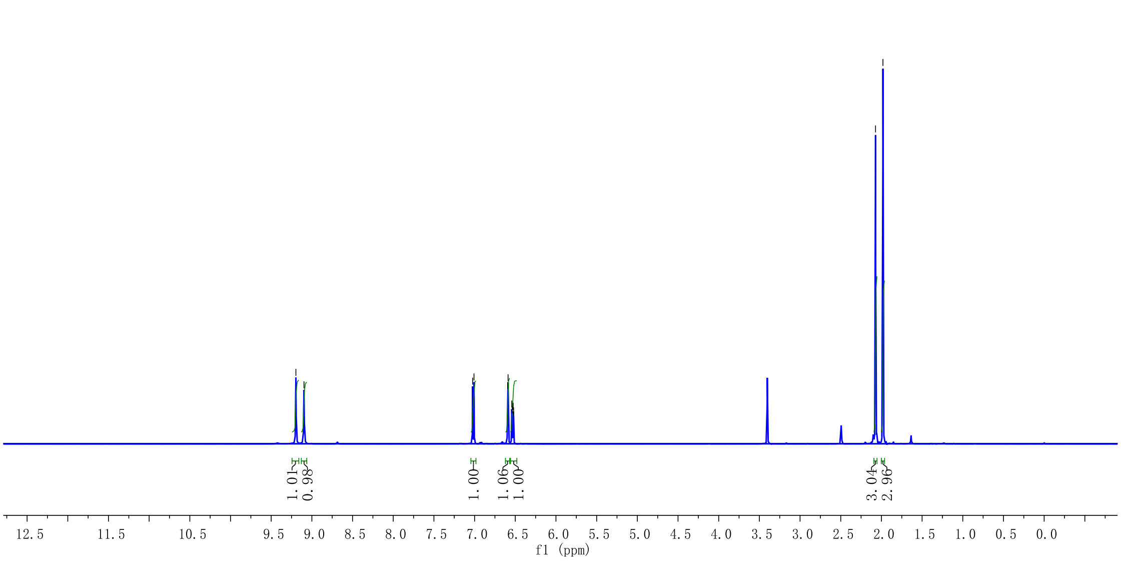

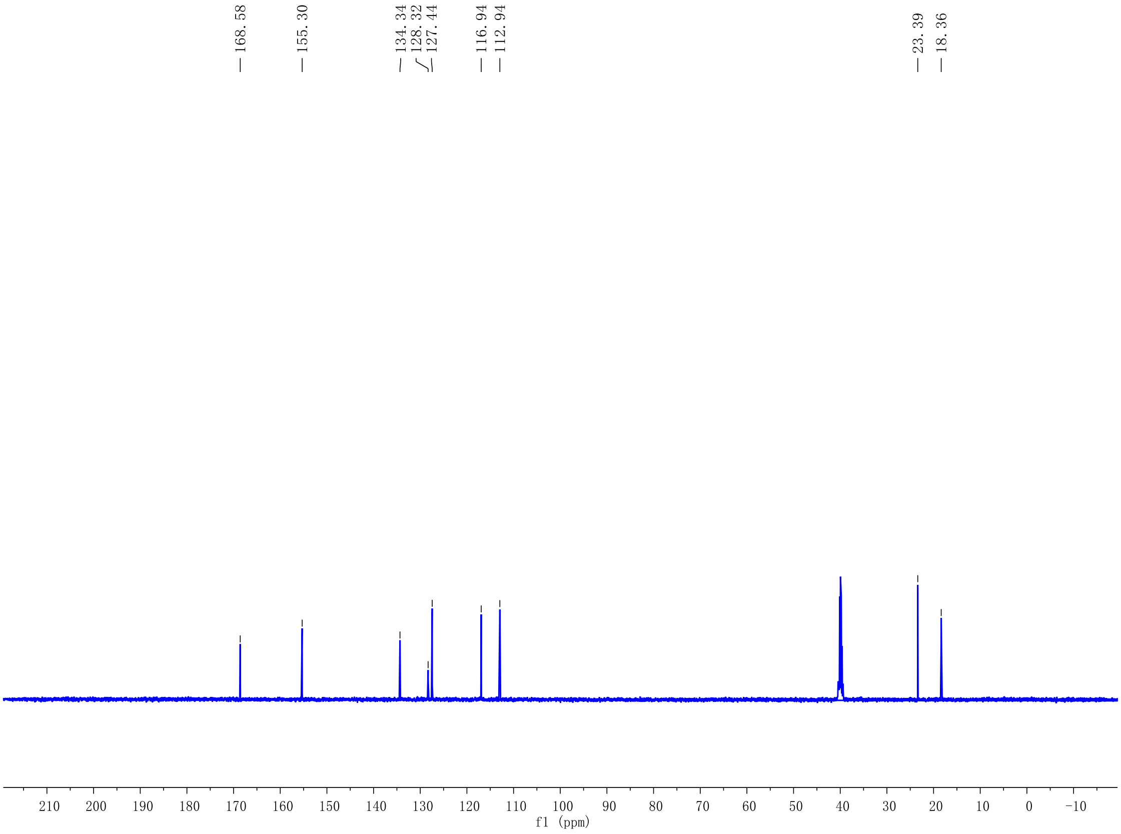

Supplementary Figure 35. ^{1}H and ^{13}C NMR spectra for compound **2f**

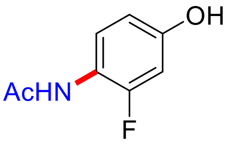
OH
AcHN
F

2g

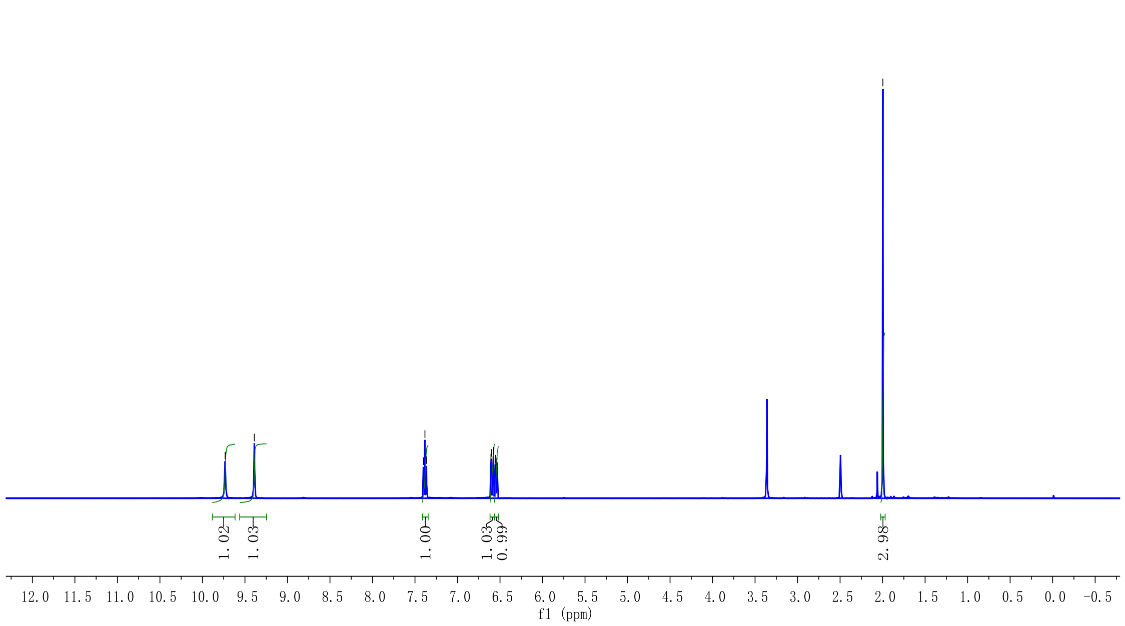
9.73
9.39
7.40
7.38
7.36
6.61
6.60
6.58
6.58
6.55
6.55
6.54
6.53
2.00
1.02
1.03
1.00
1.03
0.99
2.98
12.0 11.5 11.0 10.5 10.0 9.5 9.0 8.5 8.0 7.5 7.0 6.5 6.0 5.5 5.0 4.5 4.0 3.5 3.0 2.5 2.0 1.5 1.0 0.5 0.0 -0.5
f1 (ppm)

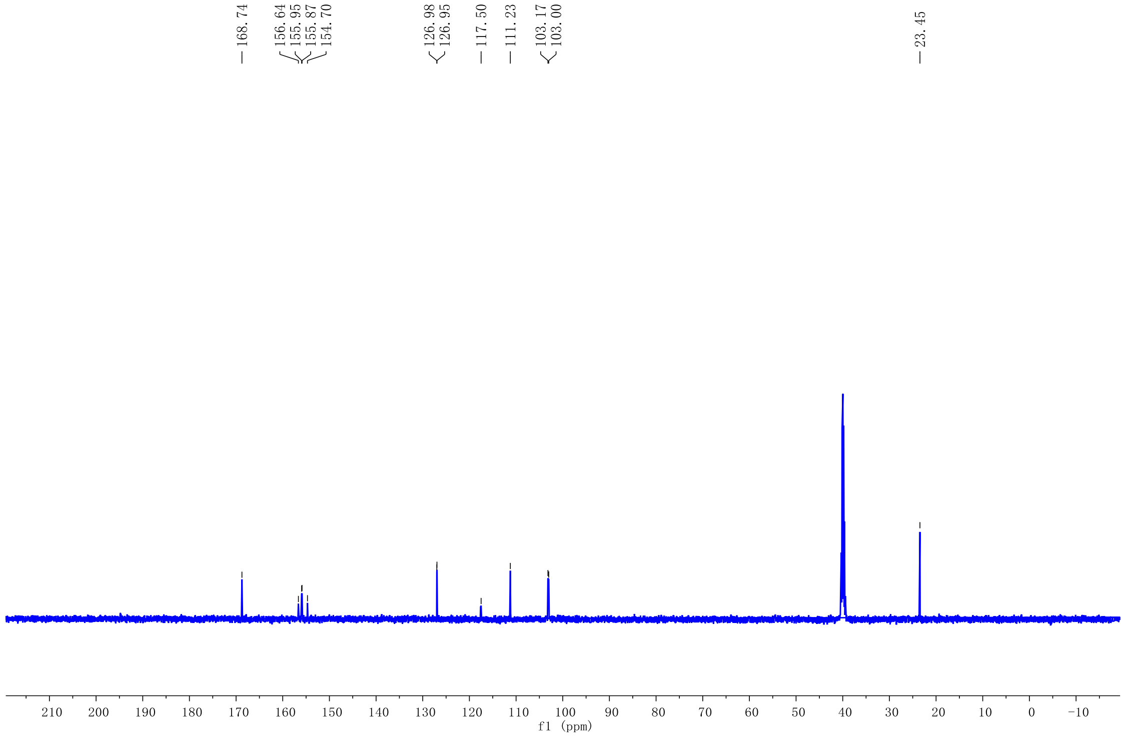
168.74
156.64
155.95
155.87
154.70
126.98
126.95
117.50
111.23
103.17
103.00
23.45
210 200 190 180 170 160 150 140 130 120 110 100 90 80 70 60 50 40 30 20 10 0 -10
f1 (ppm)

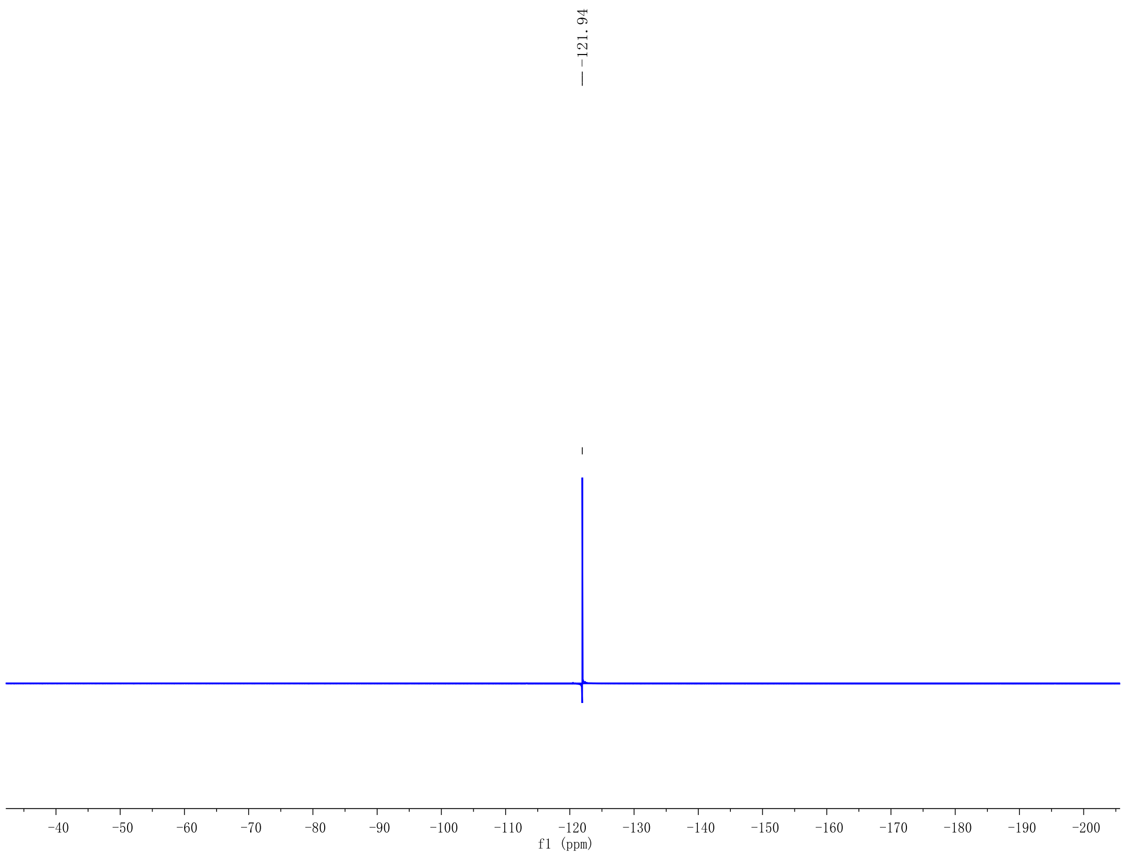

Supplementary Figure 36. ^{1}H, ^{13}C and ^{19}F NMR spectra for compound **2g**

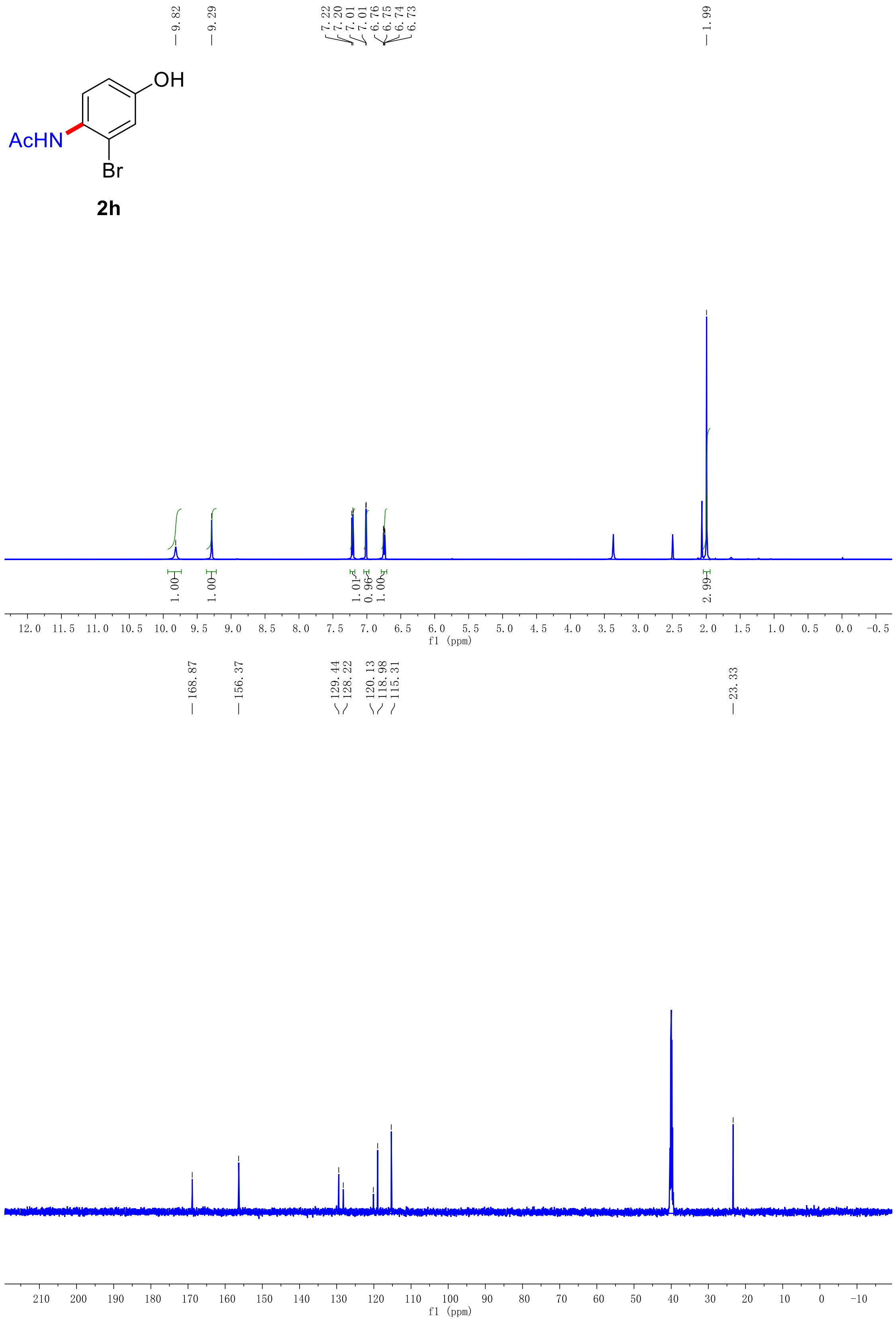

Supplementary Figure 37. ^{1}H and ^{13}C NMR spectra for compound **2h**

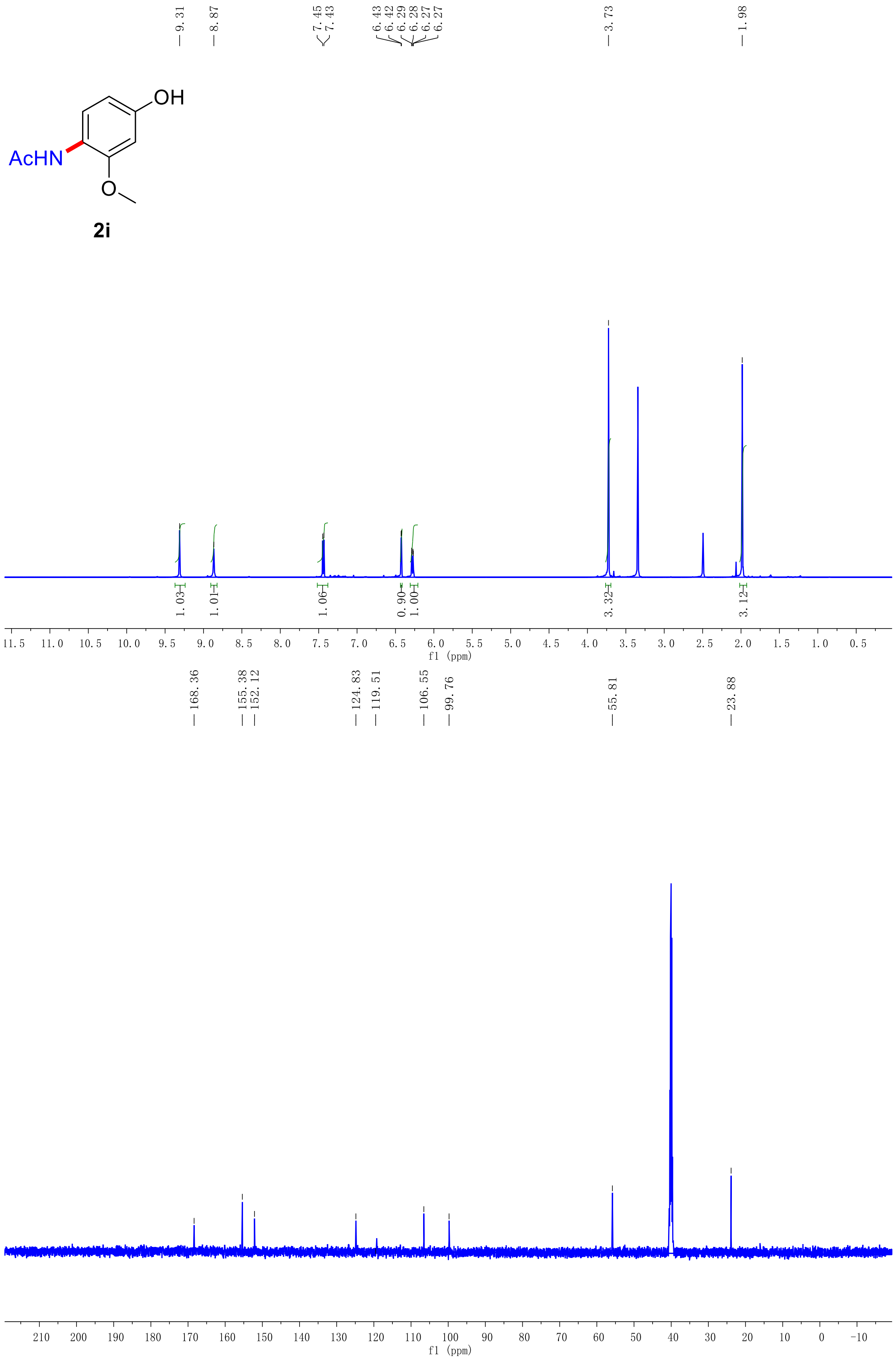

Supplementary Figure 38. ^{1}H and ^{13}C NMR spectra for compound **2i**

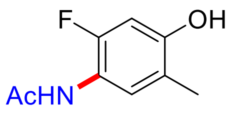
F
OH
AcHN

2j

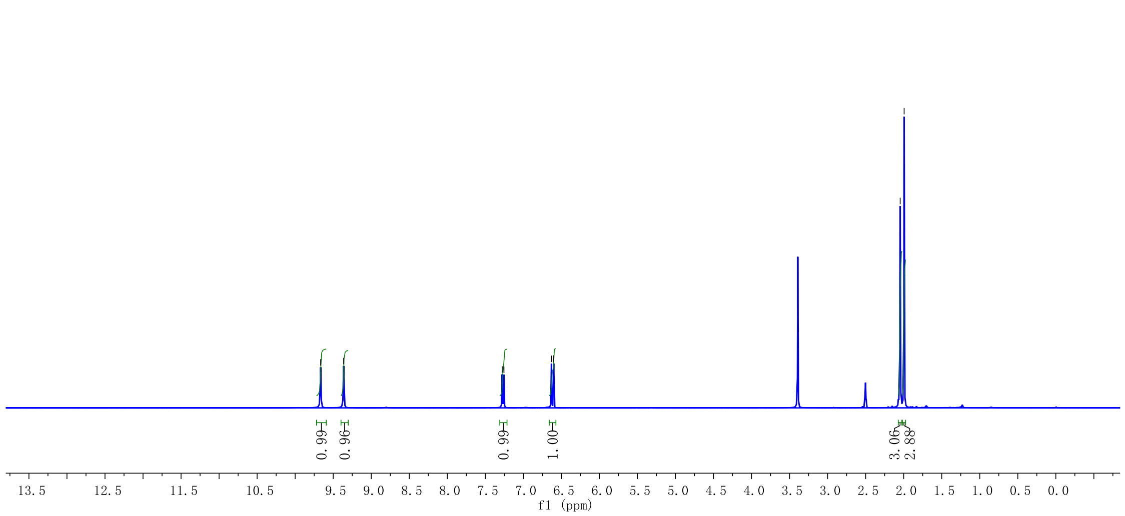
9.66
9.36
7.28
7.26
6.63
6.60
2.05
1.99
0.99
0.96
0.99
1.00
3.06
2.88
13.5 12.5 11.5 10.5 9.5 9.0 8.5 8.0 7.5 7.0 6.5 6.0 5.5 5.0 4.5 4.0 3.5 3.0 2.5 2.0 1.5 1.0 0.5 0.0
f1 (ppm)

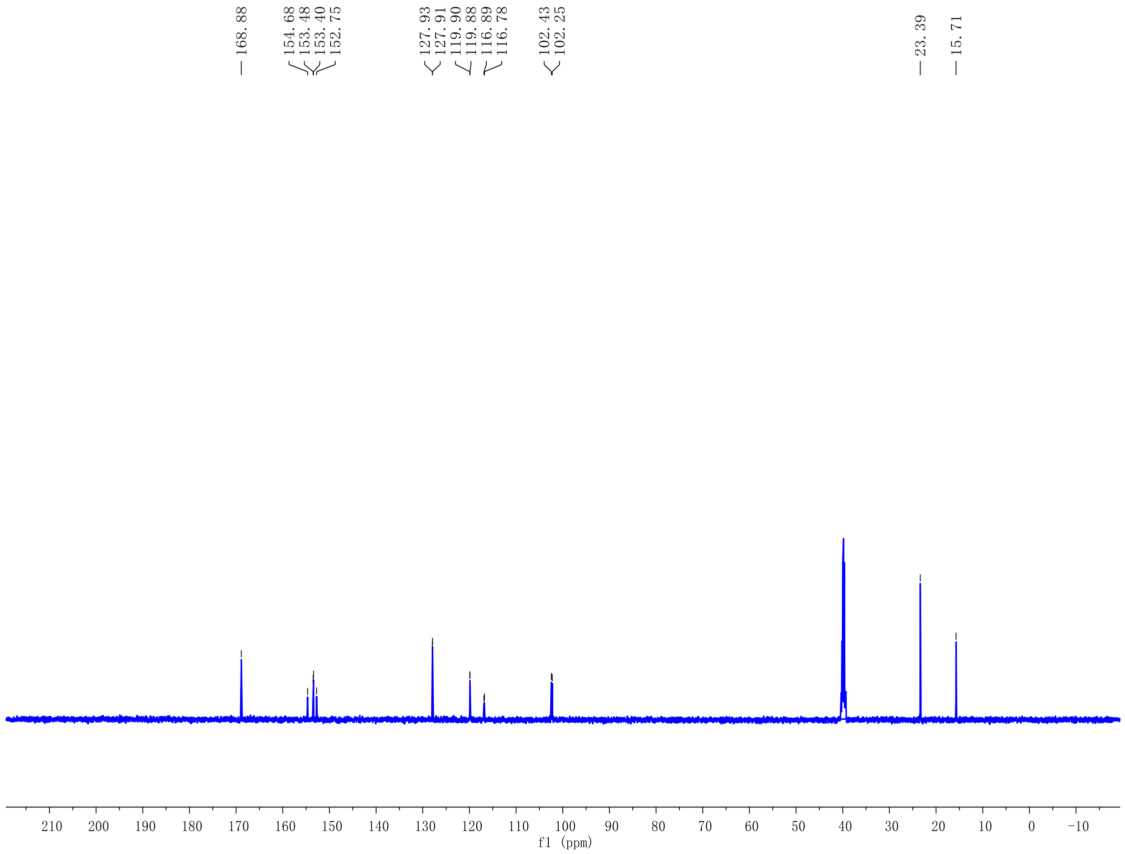
168.88
154.68
153.48
153.40
152.75
127.93
127.91
119.90
119.88
116.89
116.78
102.43
102.25
23.39
15.71
210 200 190 180 170 160 150 140 130 120 110 100 90 80 70 60 50 40 30 20 10 0 -10
f1 (ppm)

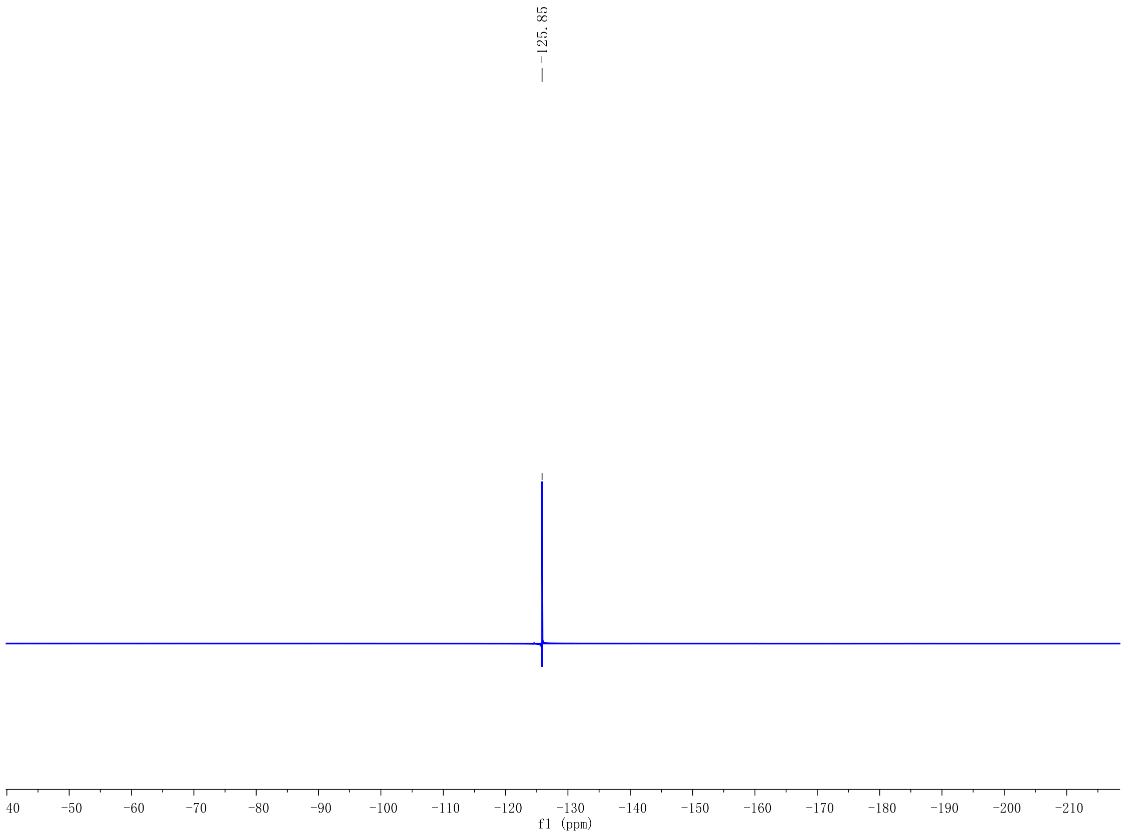

Supplementary Figure 39. ^{1}H, ^{13}C and ^{19}F NMR spectra for compound **2j**

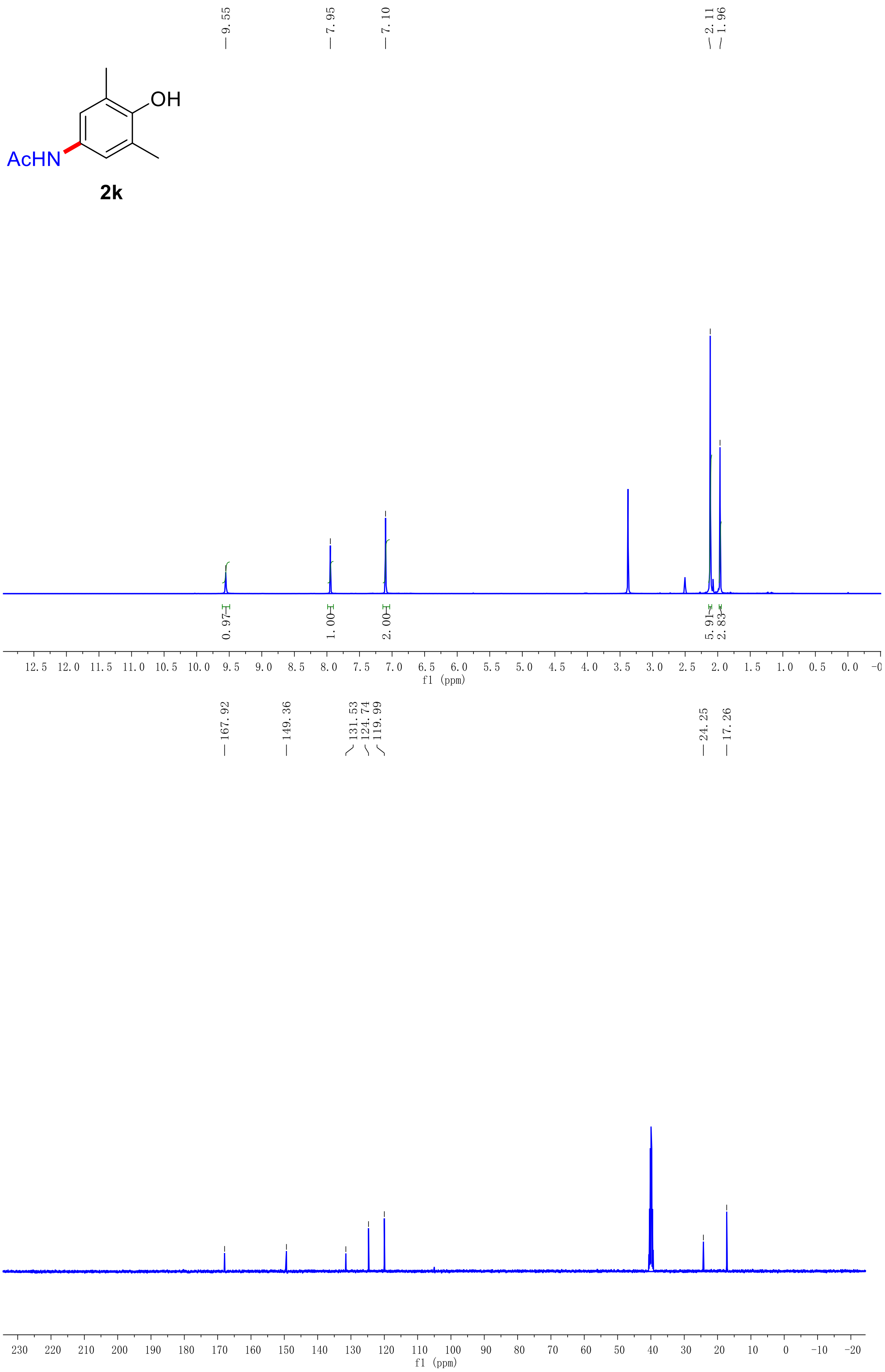

Supplementary Figure 40. ^{1}H and ^{13}C NMR spectra for compound **2k**

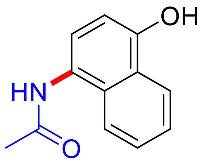

2l

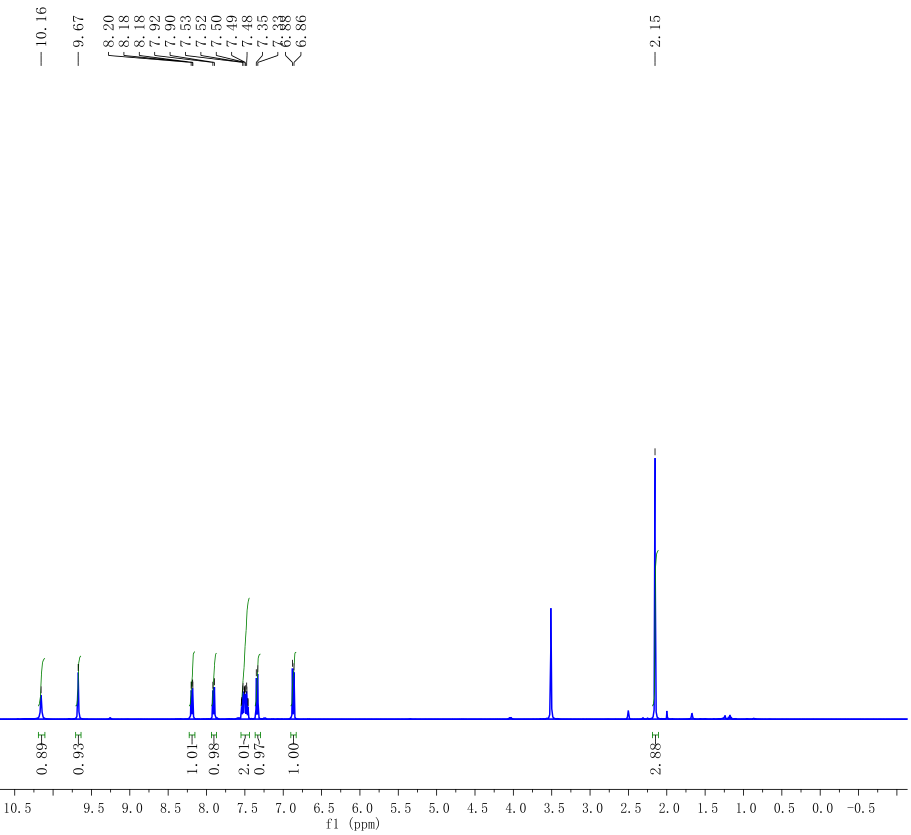

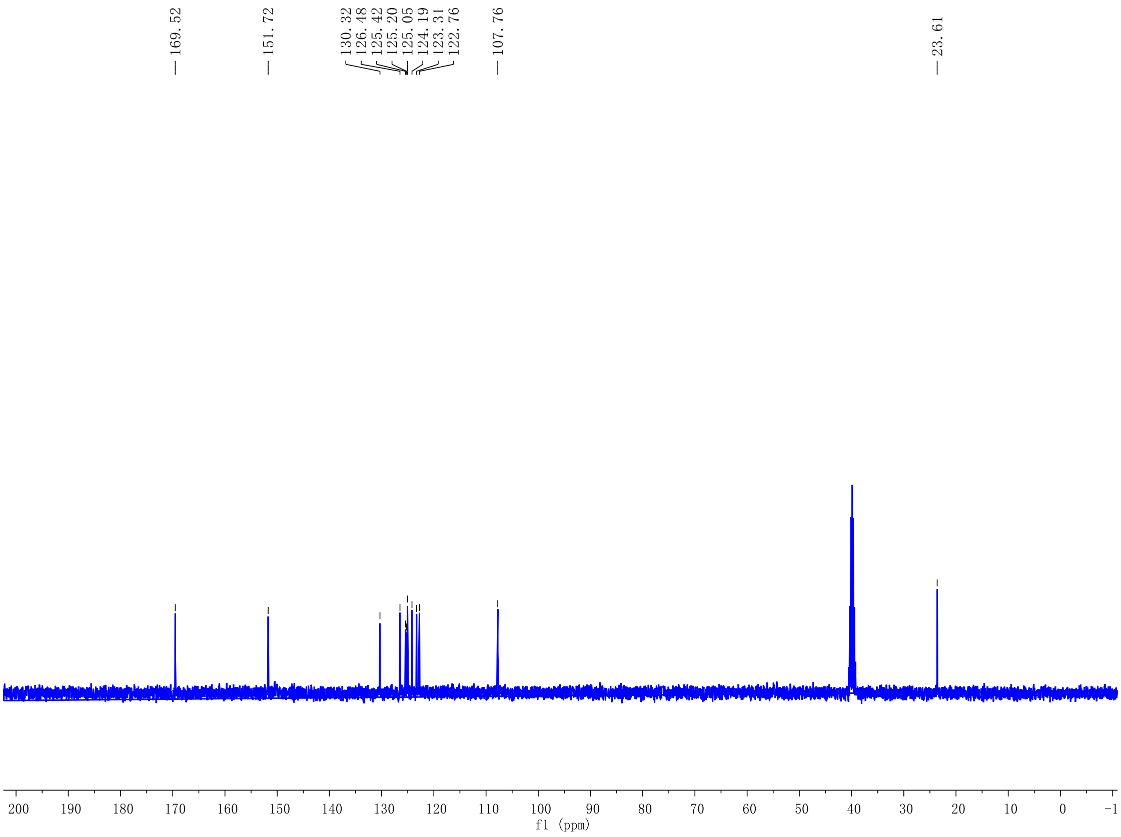

Supplementary Figure 41. ^{1}H and ^{13}C NMR spectra for compound **2l**

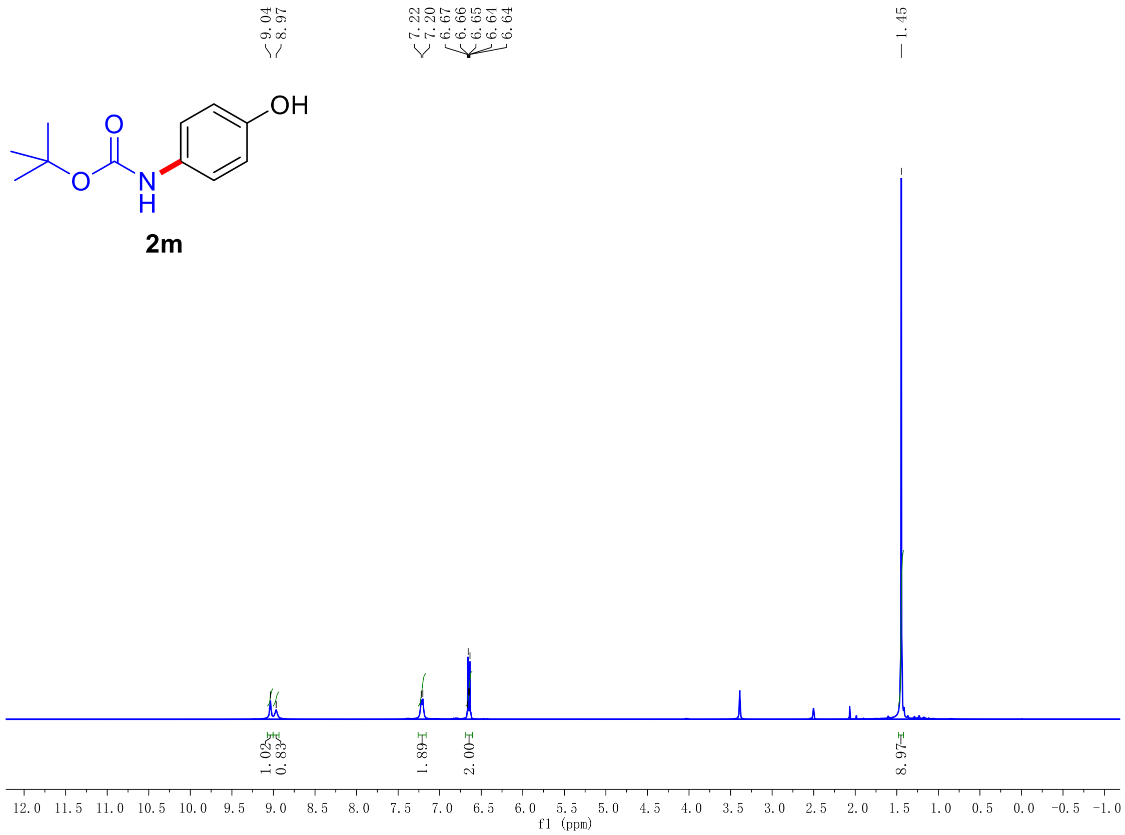

2m

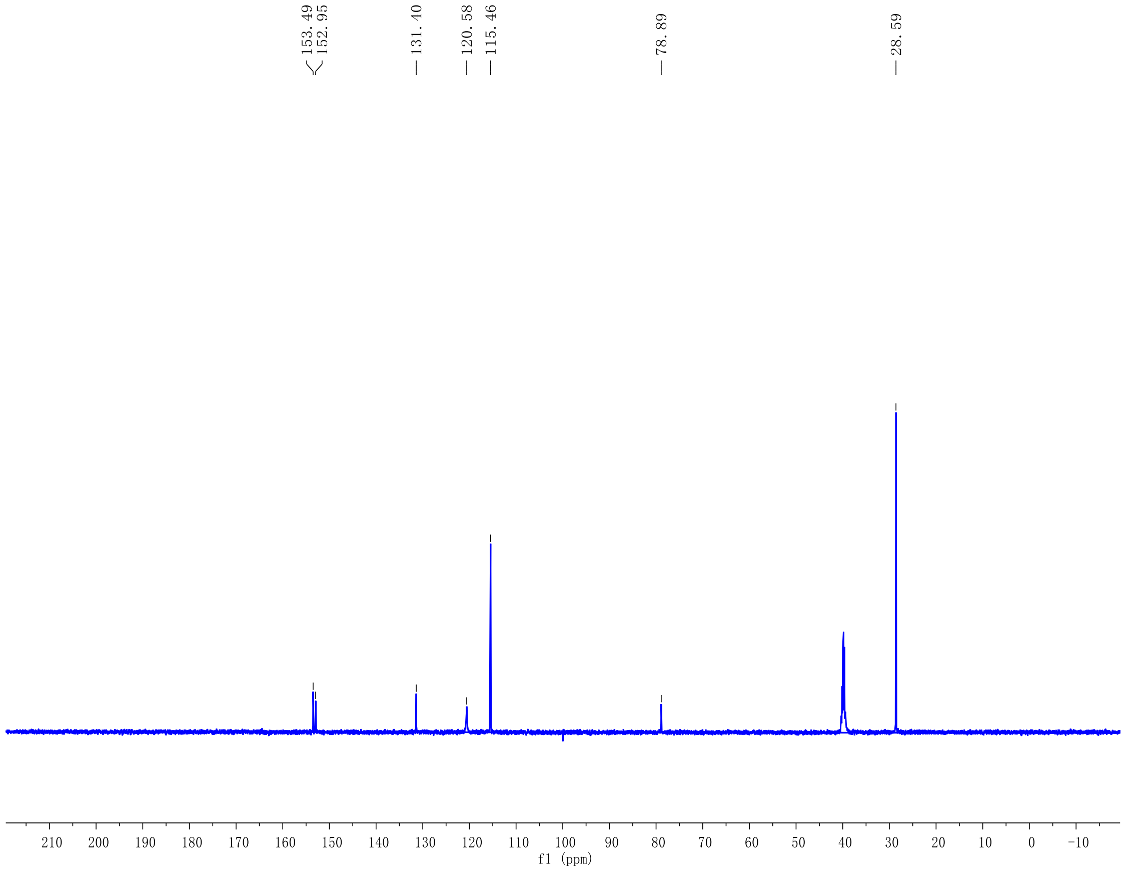

Supplementary Figure 42. ^{1}H and ^{13}C NMR spectra for compound **2m**

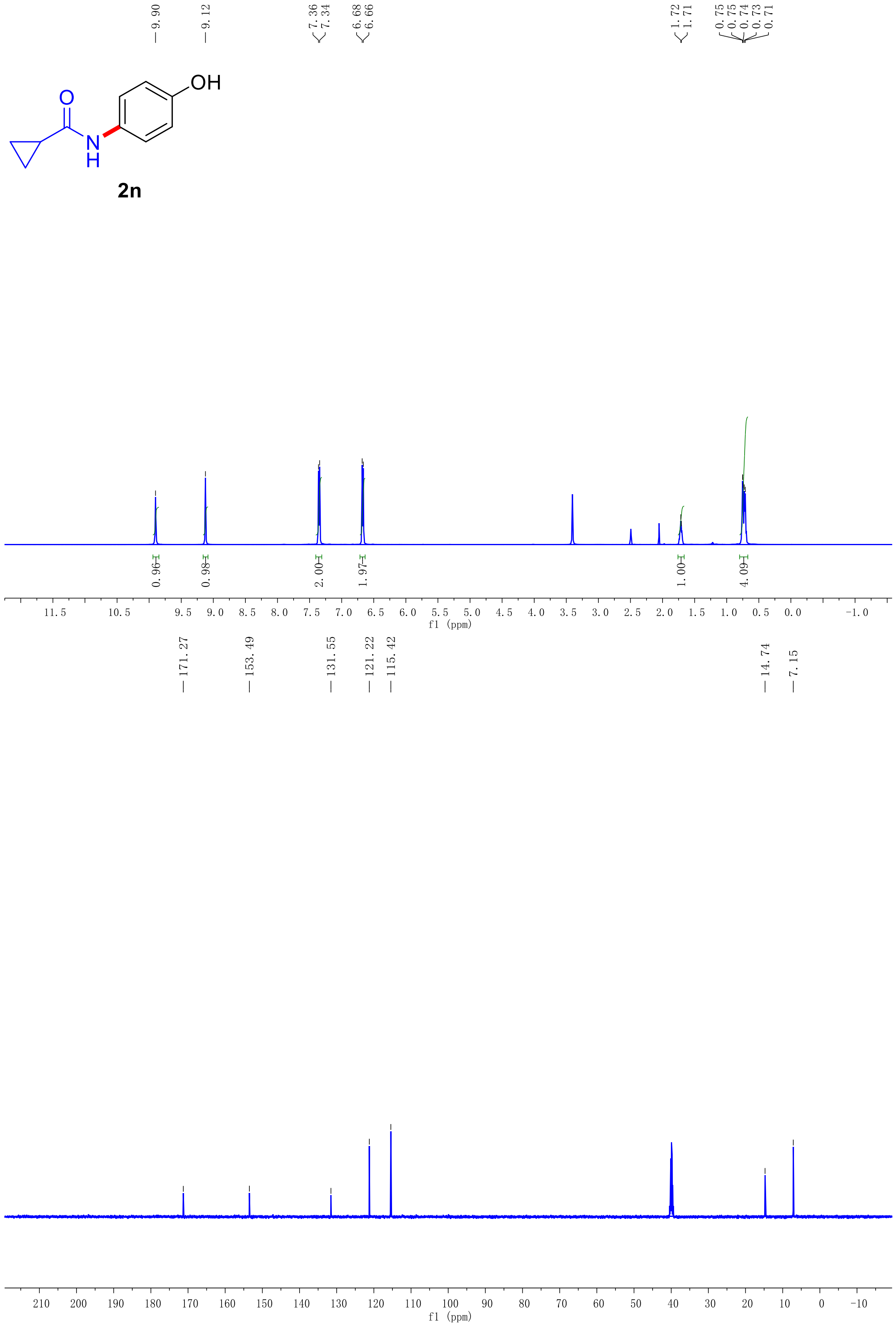

Supplementary Figure 43. ^{1}H and ^{13}C NMR spectra for compound **2n**

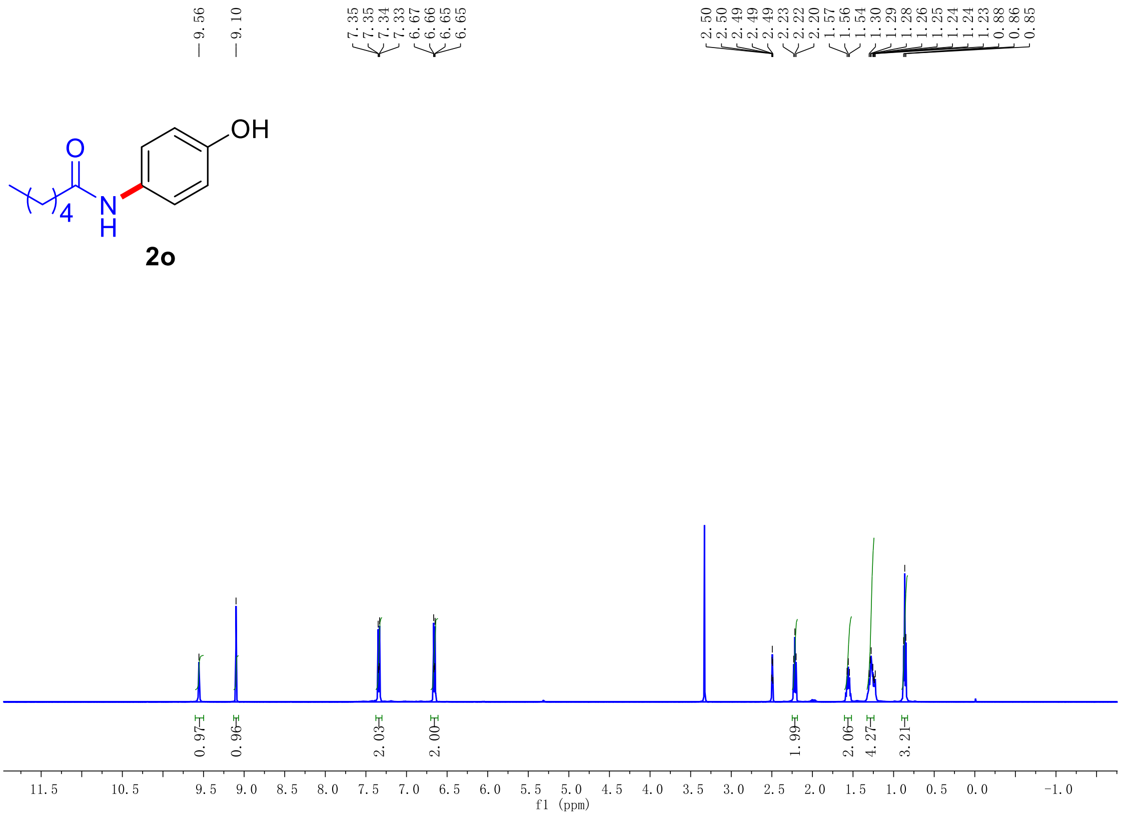

2o

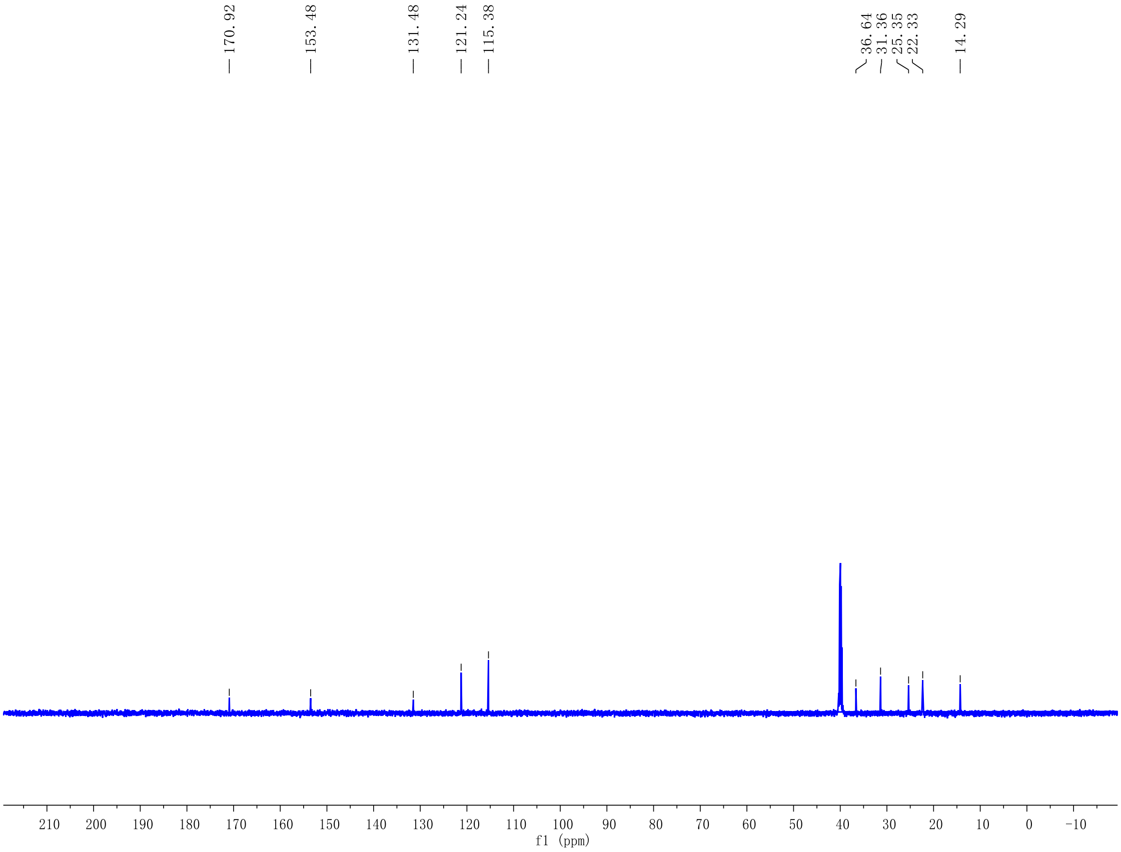

Supplementary Figure 44. ^{1}H and ^{13}C NMR spectra for compound **2o**

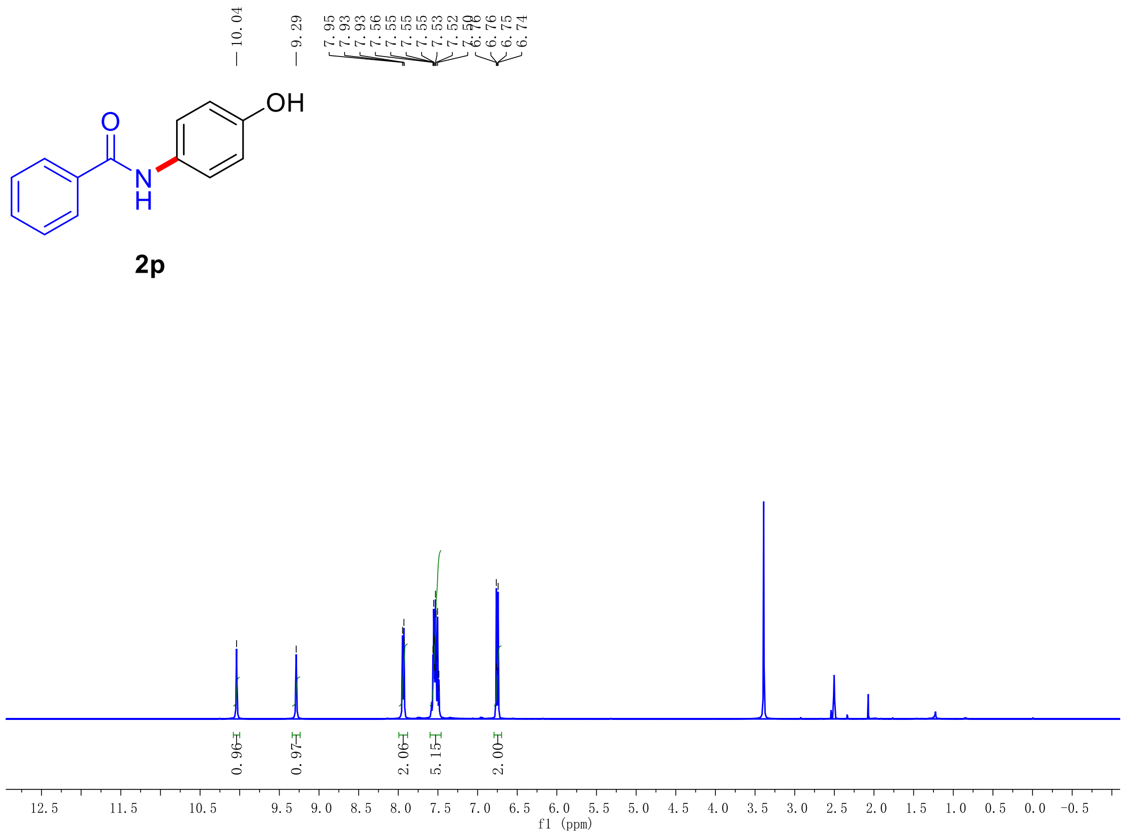

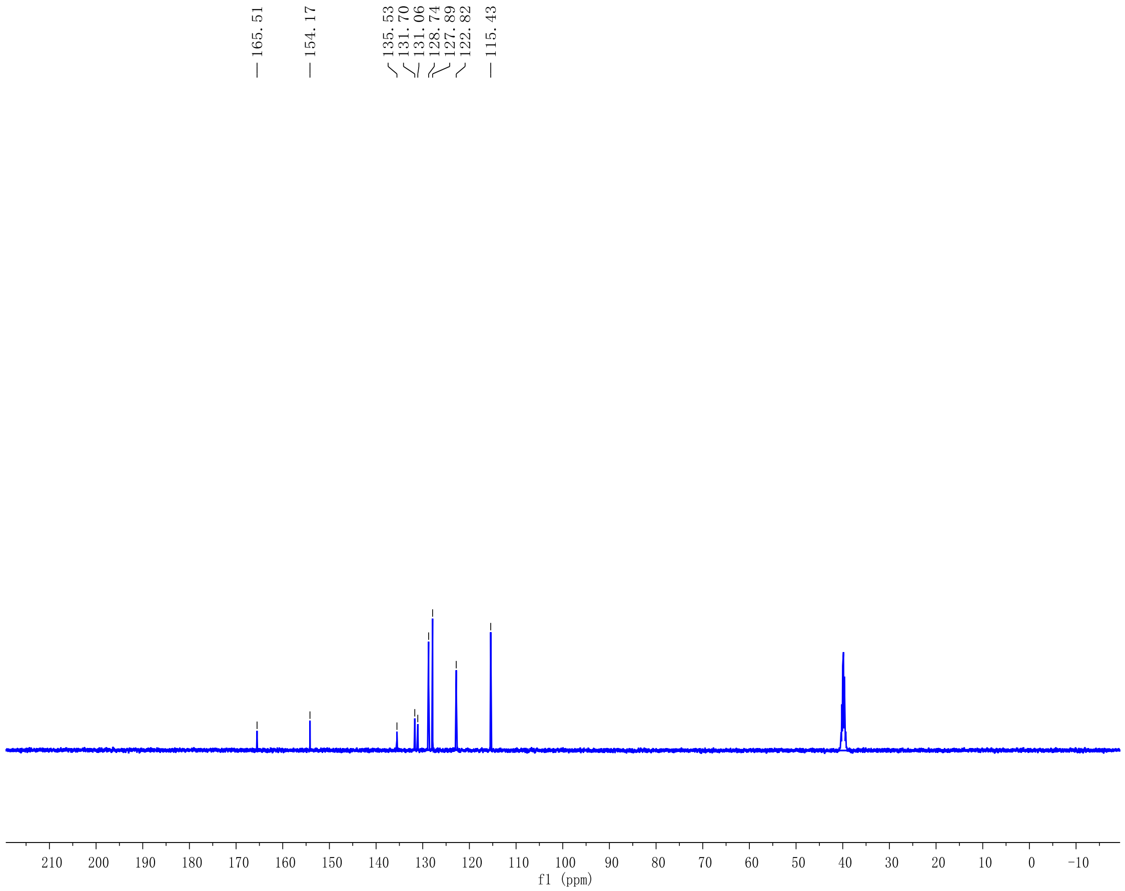

Supplementary Figure 45. ^{1}H and ^{13}C NMR spectra for compound **2p**

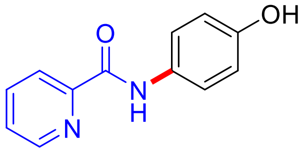

2q

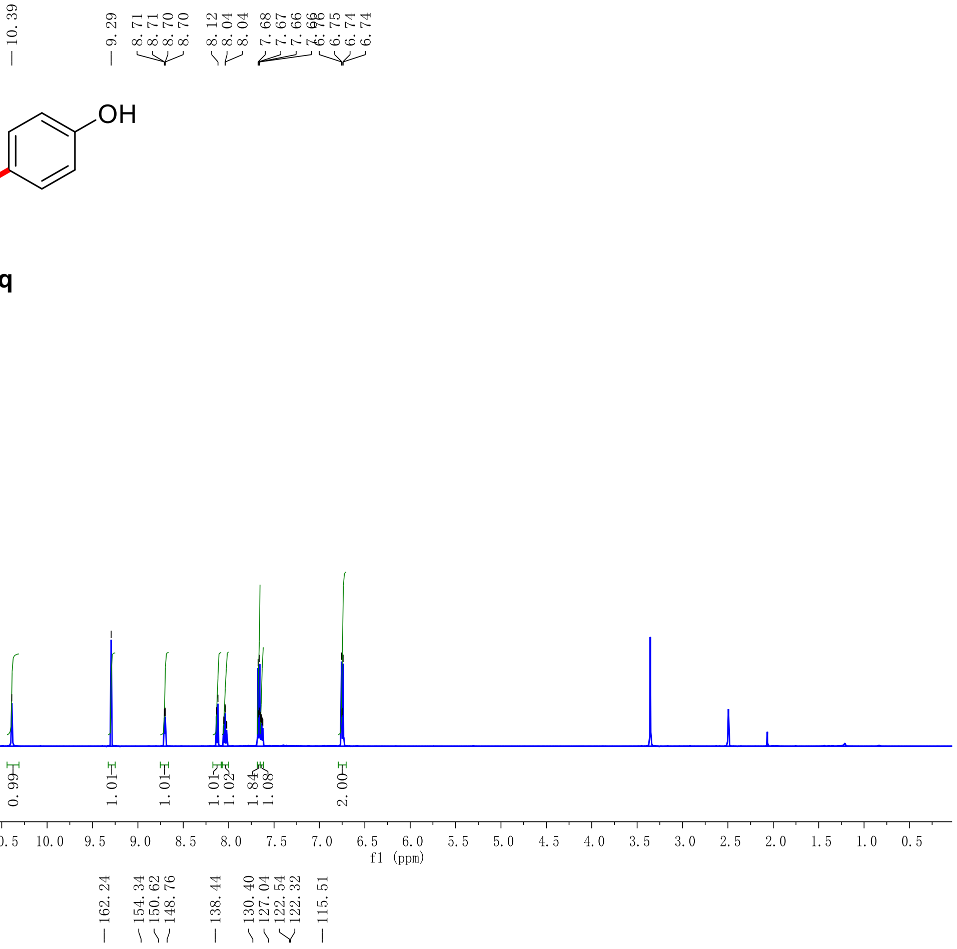

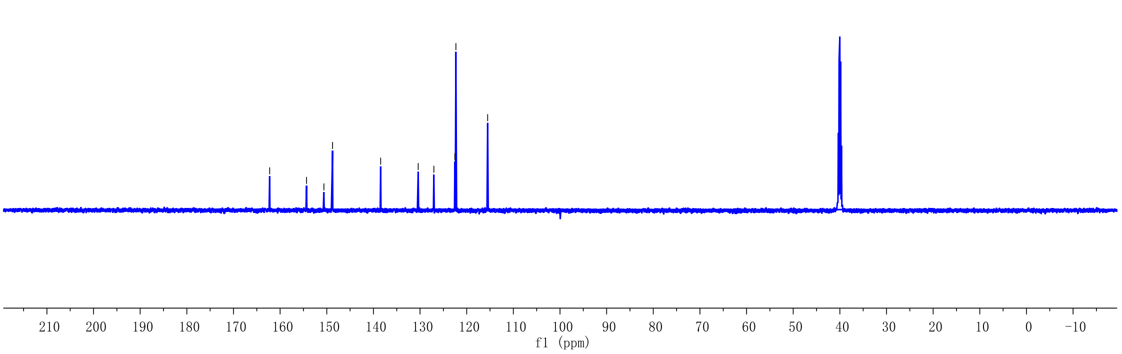

Supplementary Figure 46. ^{1}H and ^{13}C NMR spectra for compound **2q**

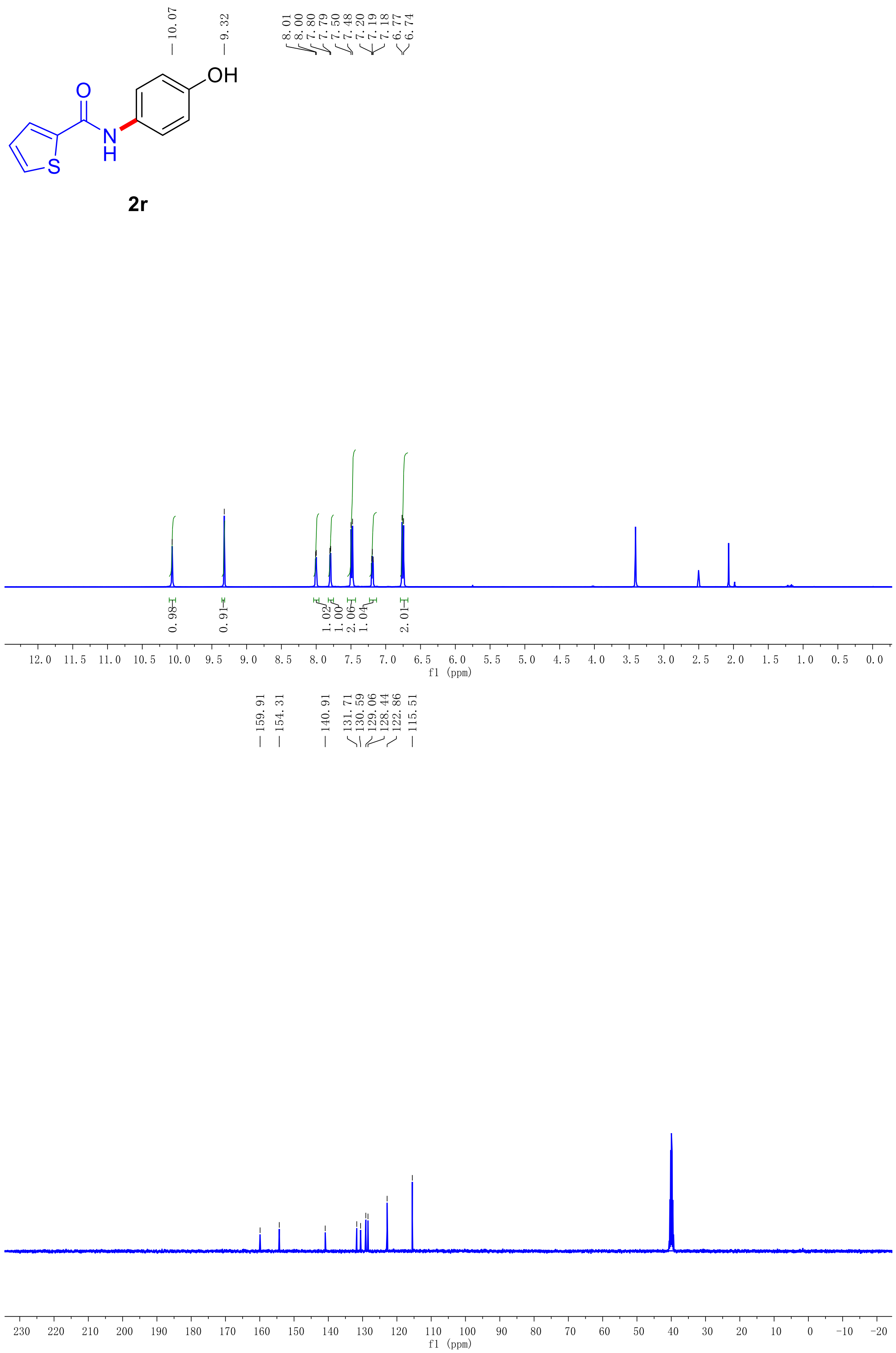

Supplementary Figure 47. ^{1}H and ^{13}C NMR spectra for compound **2r**

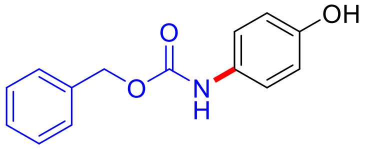

2s

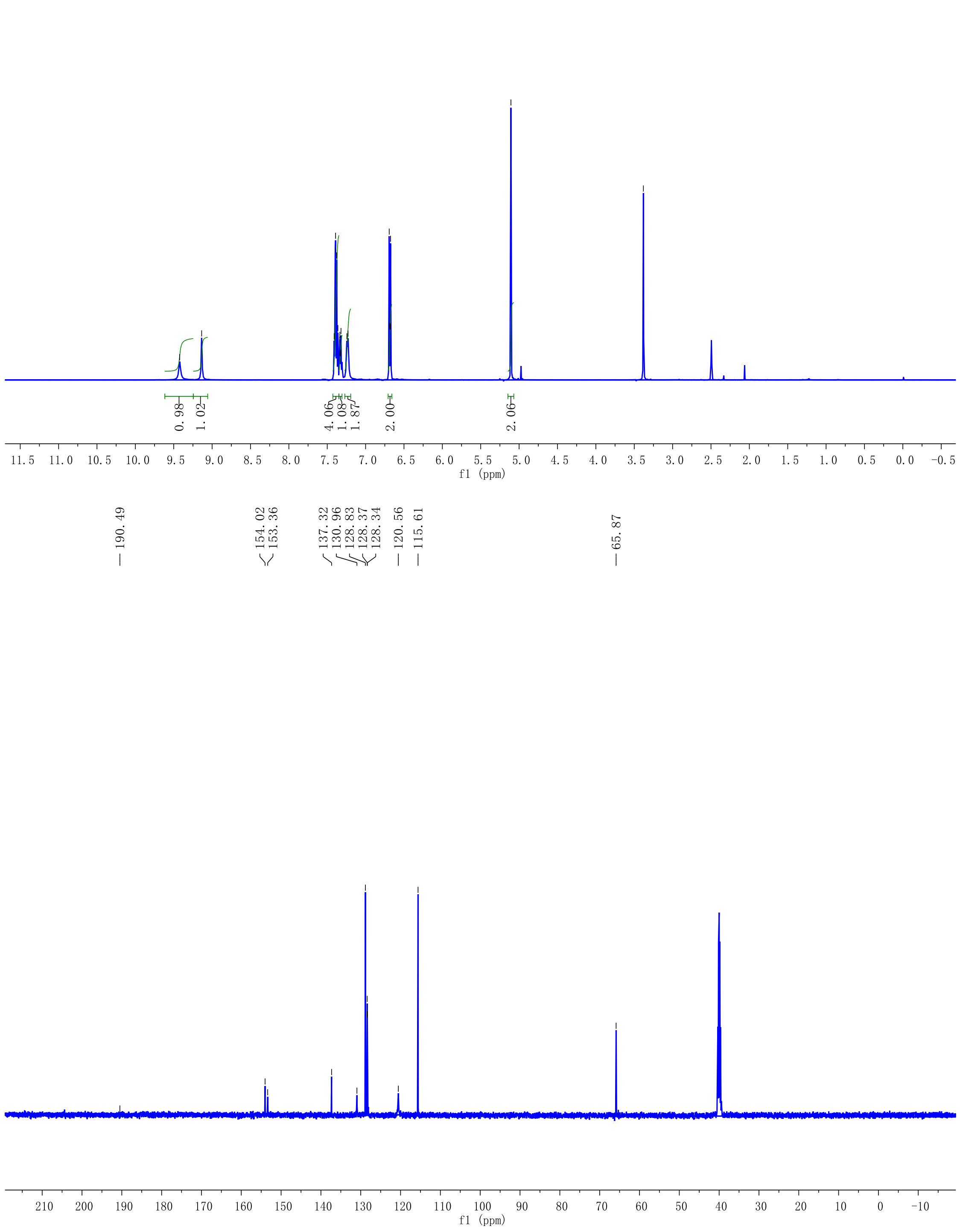

Supplementary Figure 48. ^{1}H and ^{13}C NMR spectra for compound **2s**

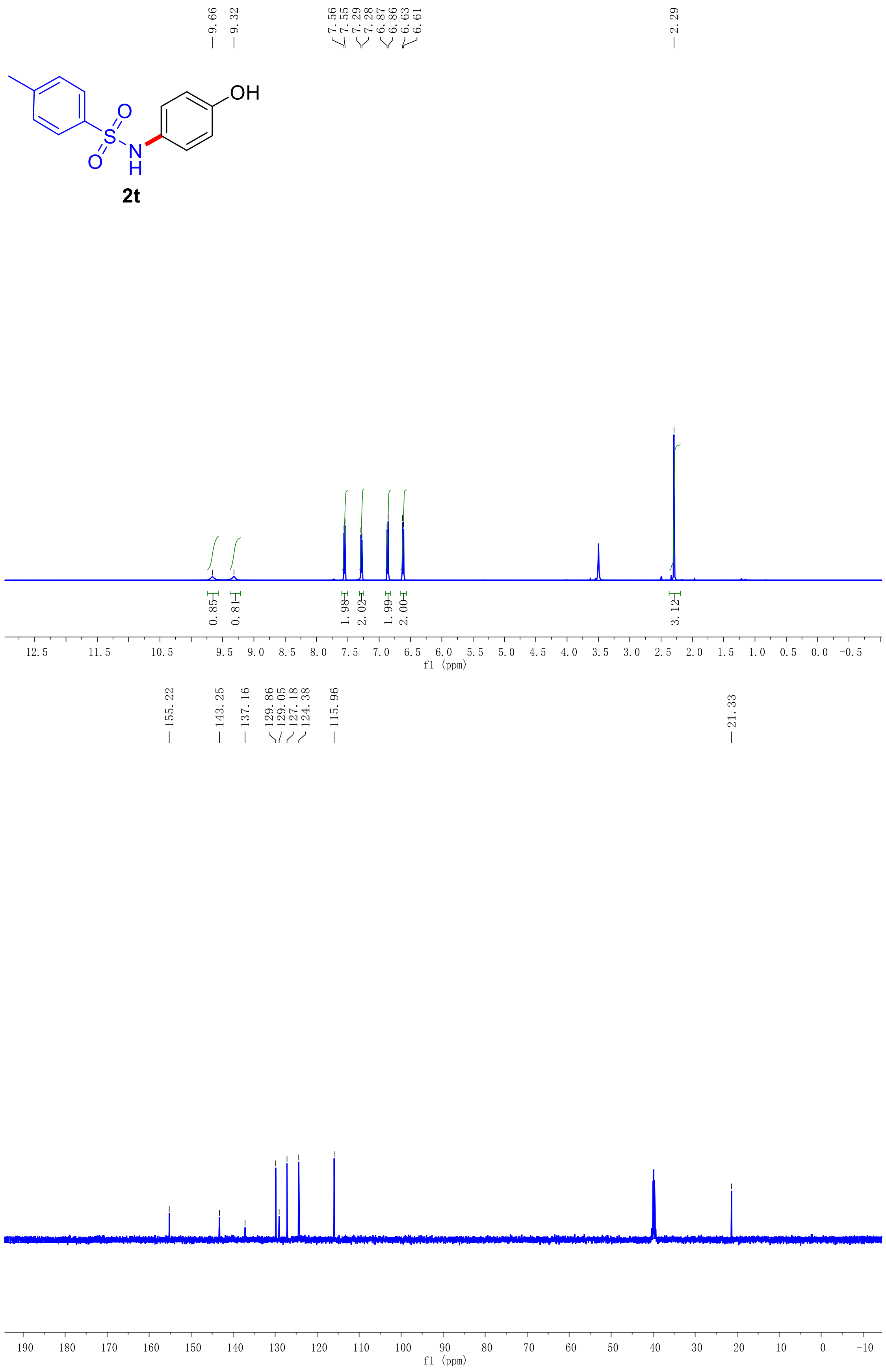

Supplementary Figure 49. ^{1}H and ^{13}C NMR spectra for compound **2t**

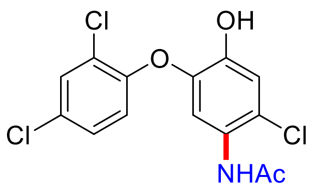

2u

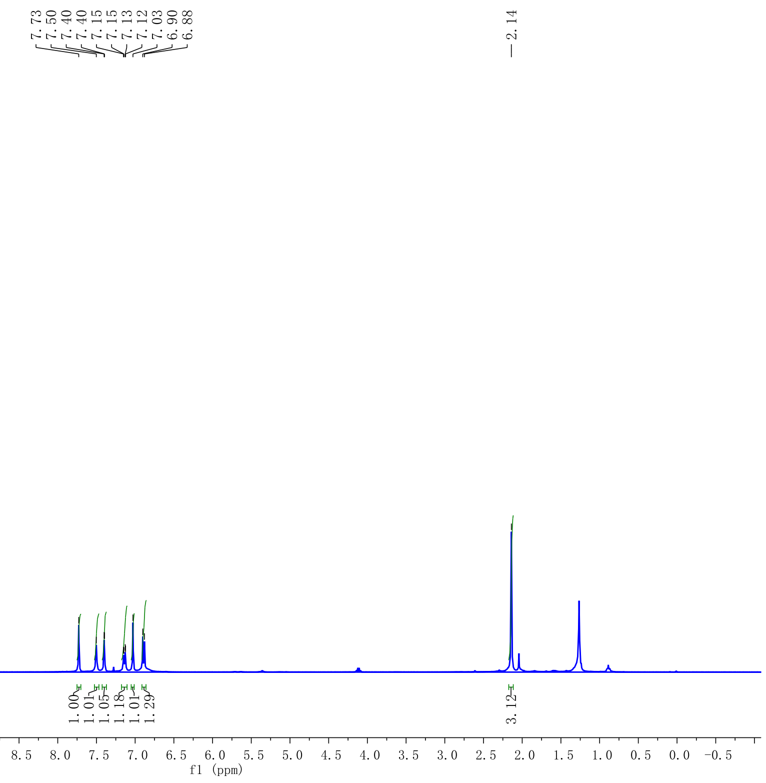

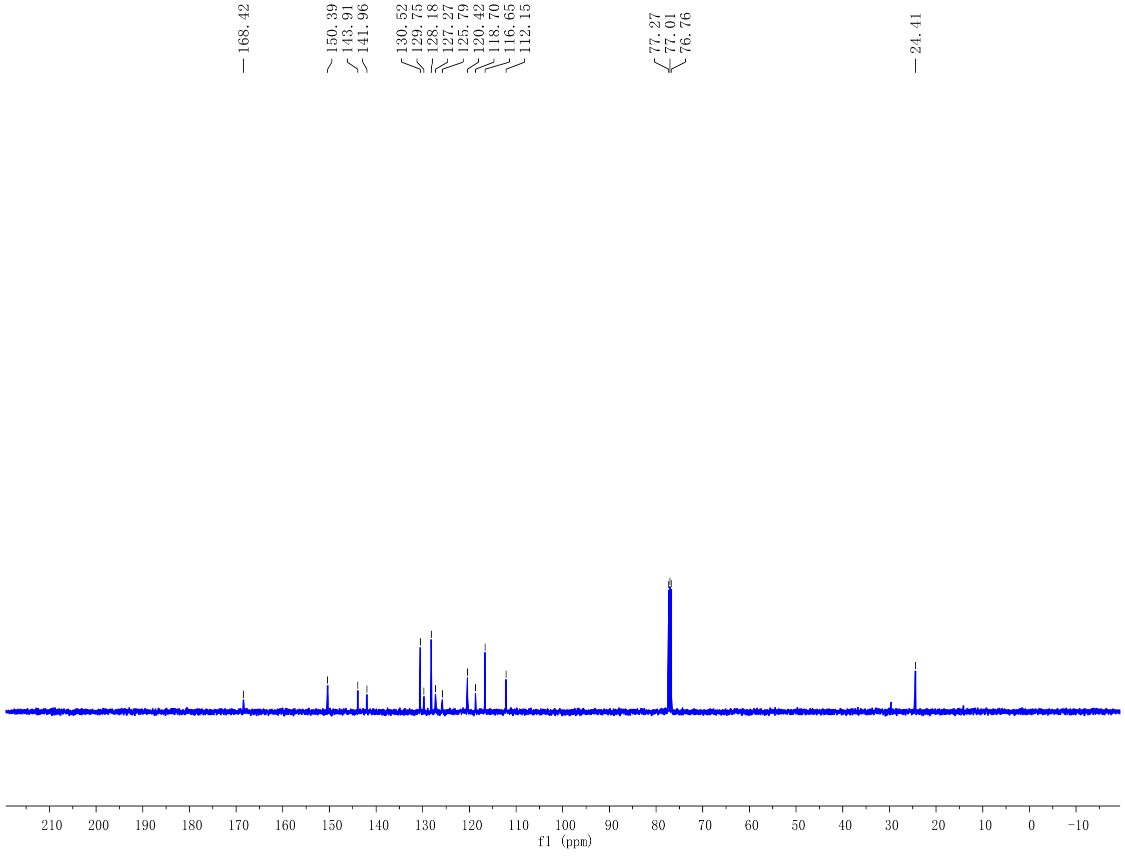

Supplementary Figure 50. ^{1}H and ^{13}C NMR spectra for compound **2u**

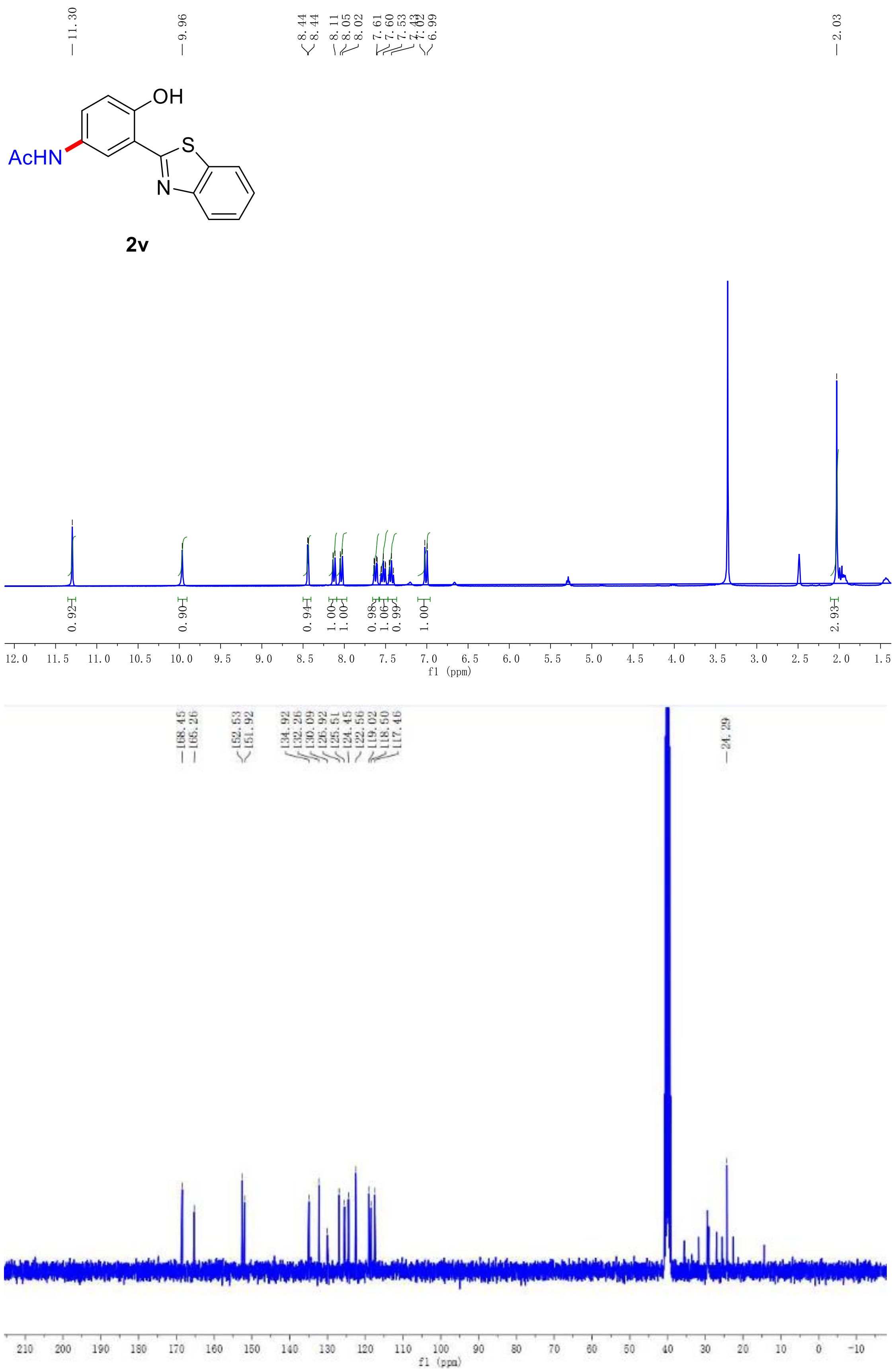

Supplementary Figure 51. ^{1}H and ^{13}C NMR spectra for compound **2v**

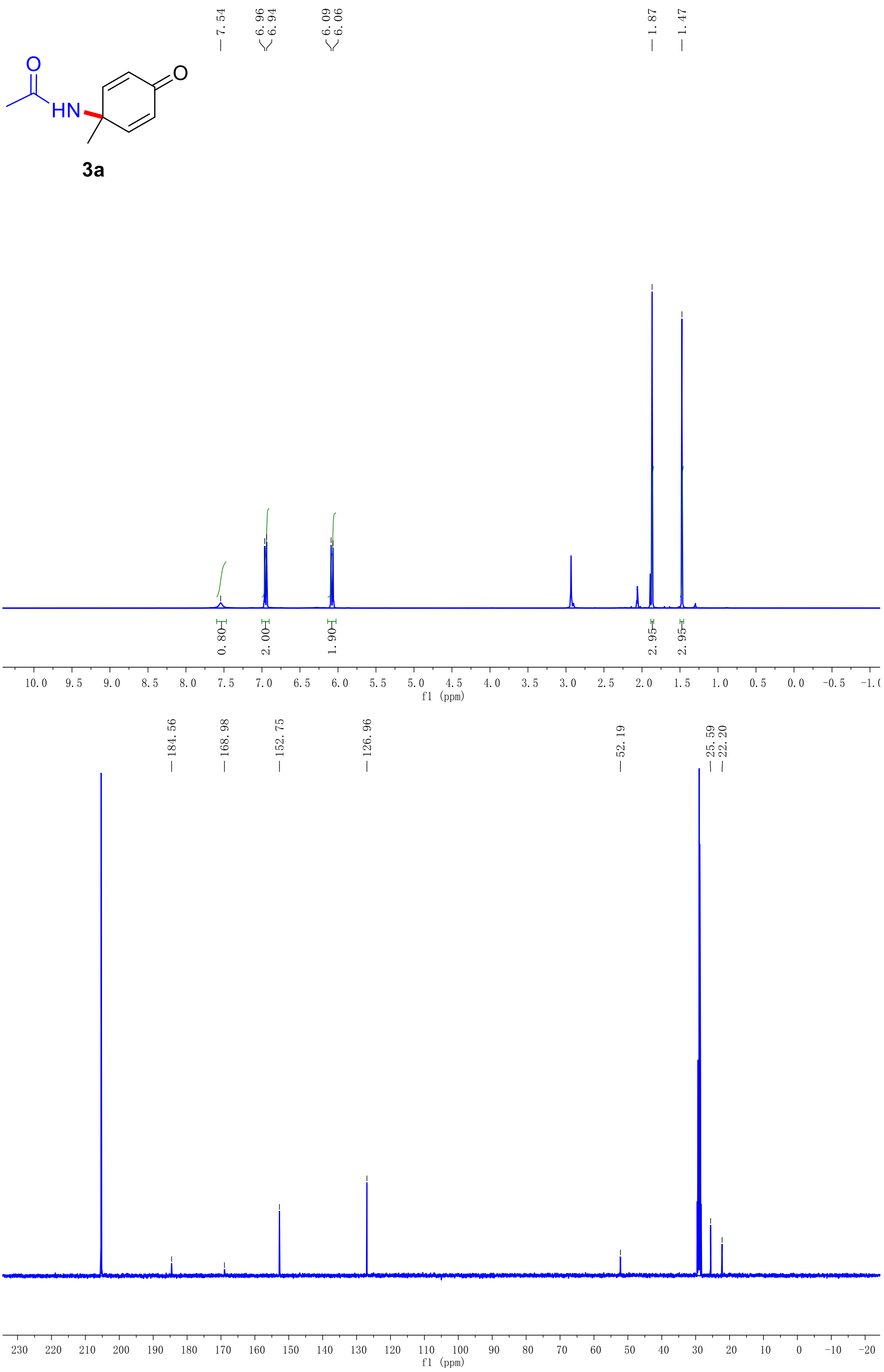

Supplementary Figure 52. ^{1}H and ^{13}C NMR spectra for compound **3a**

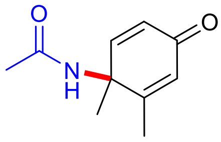

3b

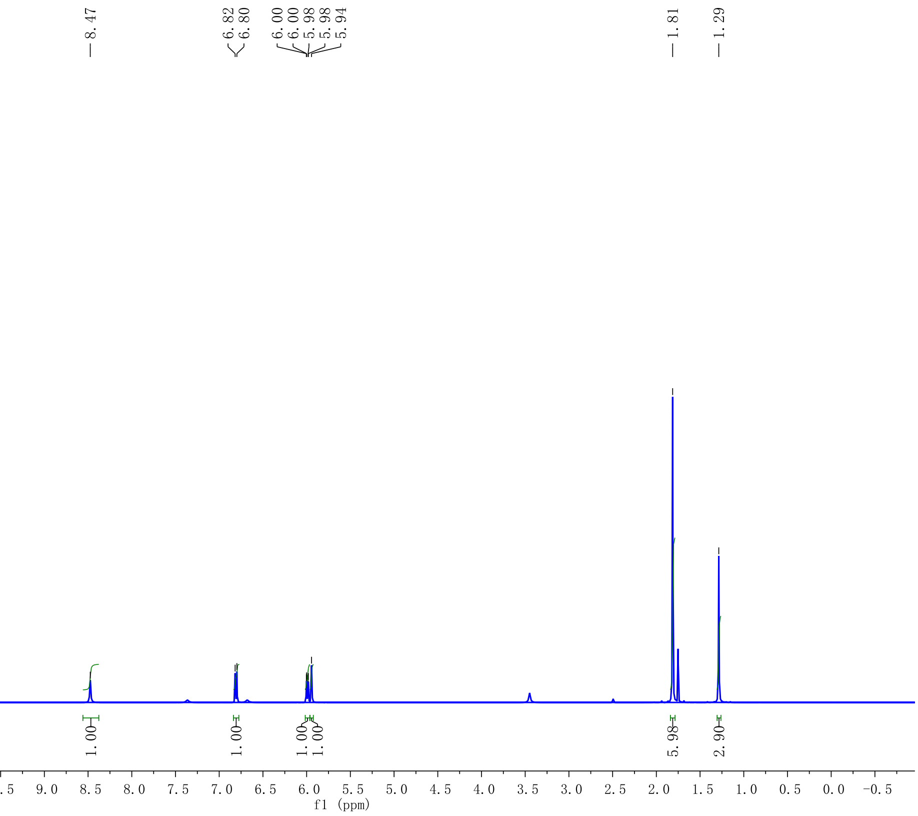

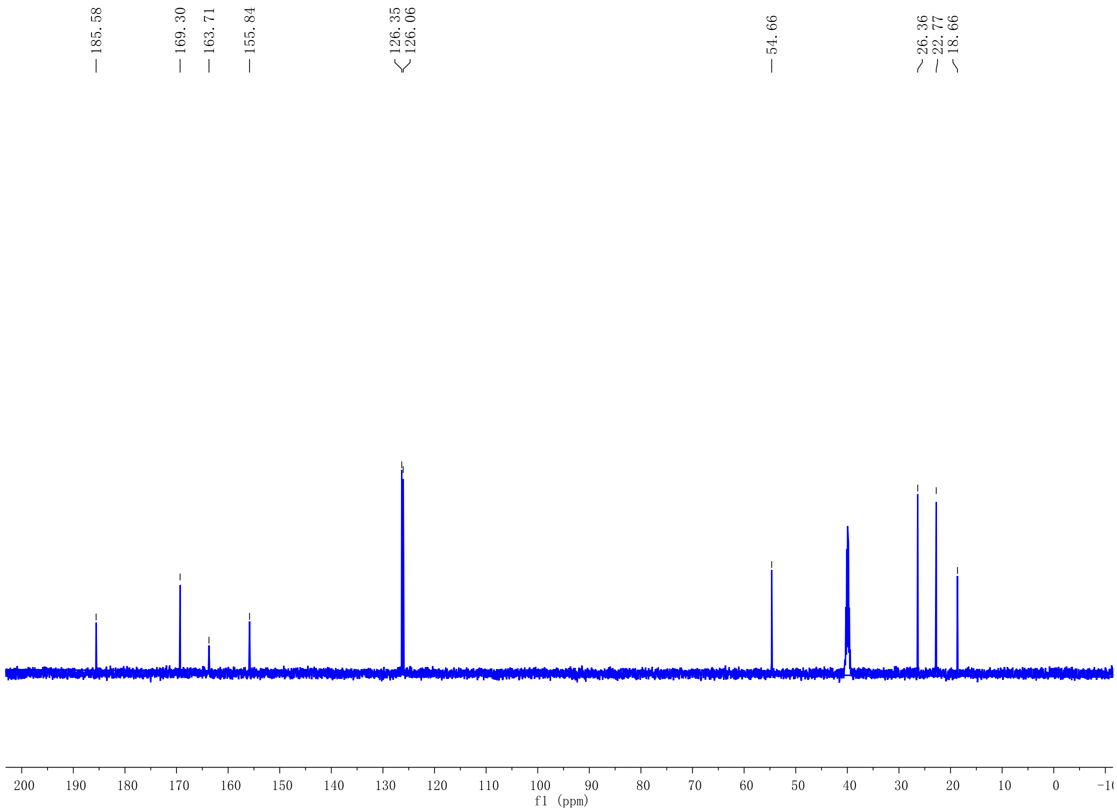

Supplementary Figure 53. ^{1}H and ^{13}C NMR spectra for compound **3b**

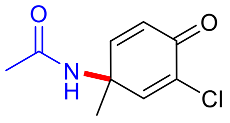

3c

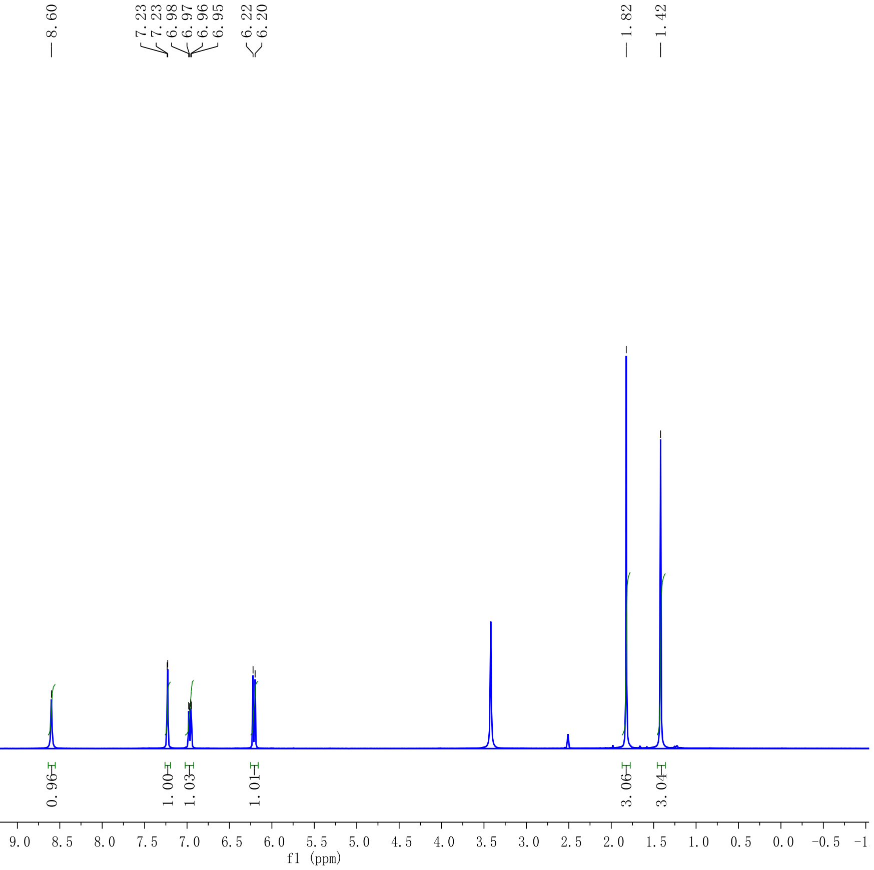

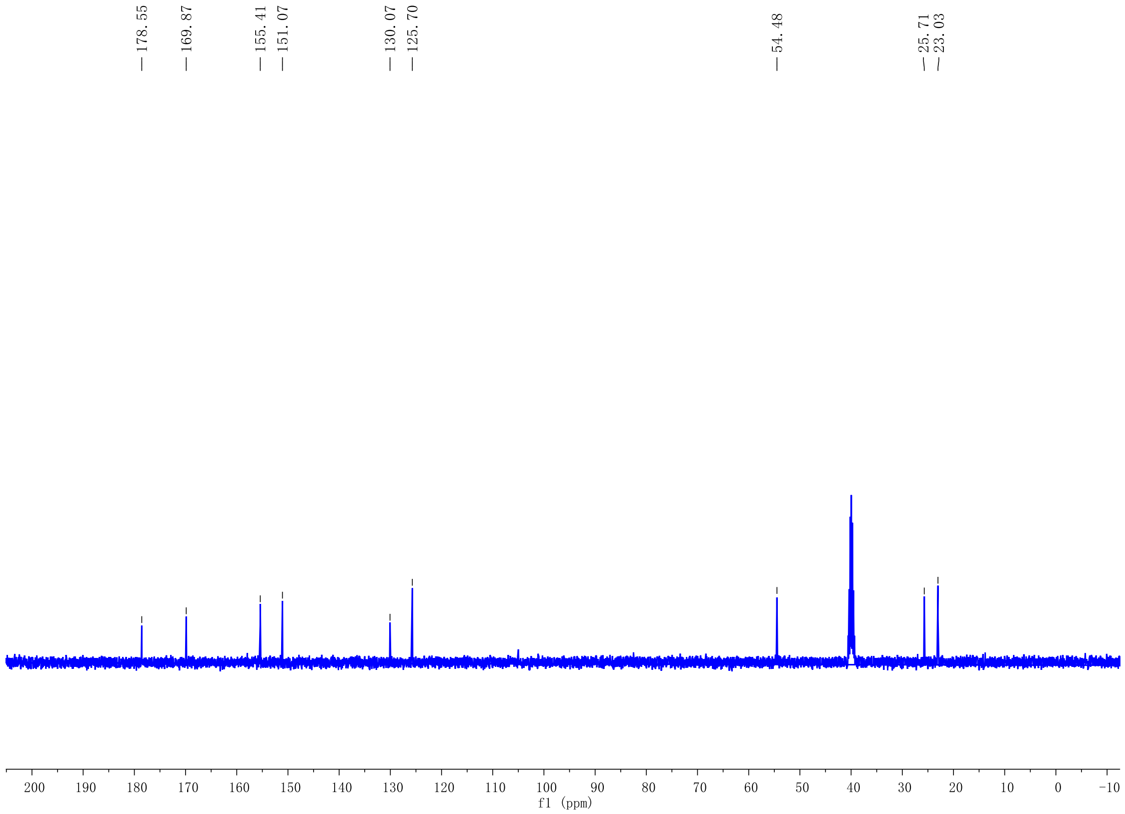

Supplementary Figure 54. ^{1}H and ^{13}C NMR spectra for compound **3c**

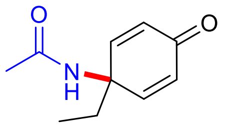

3d

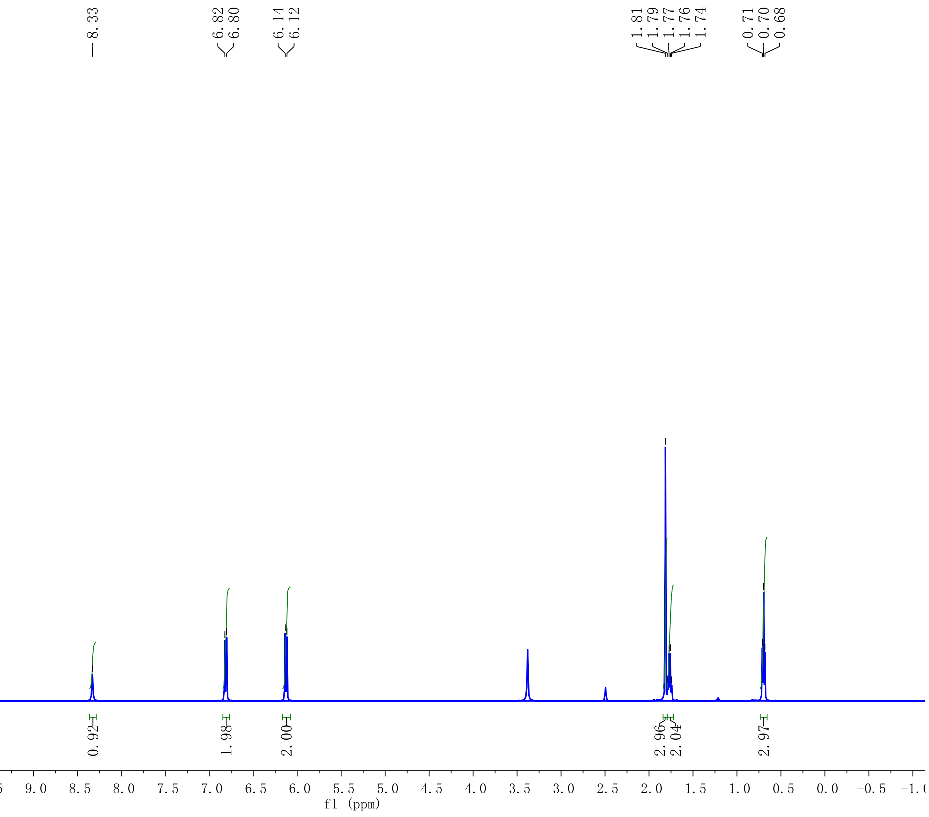

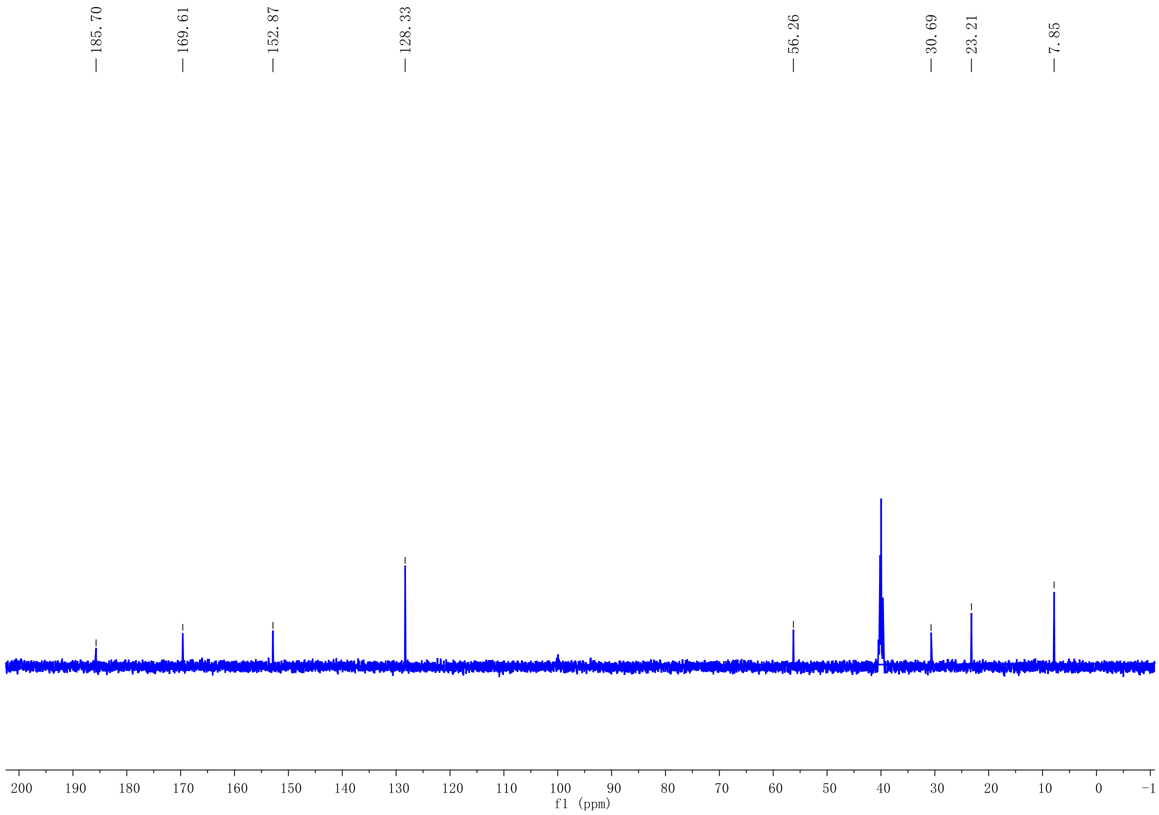

Supplementary Figure 55. ^{1}H and ^{13}C NMR spectra for compound **3d**

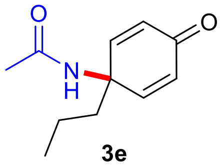

3e

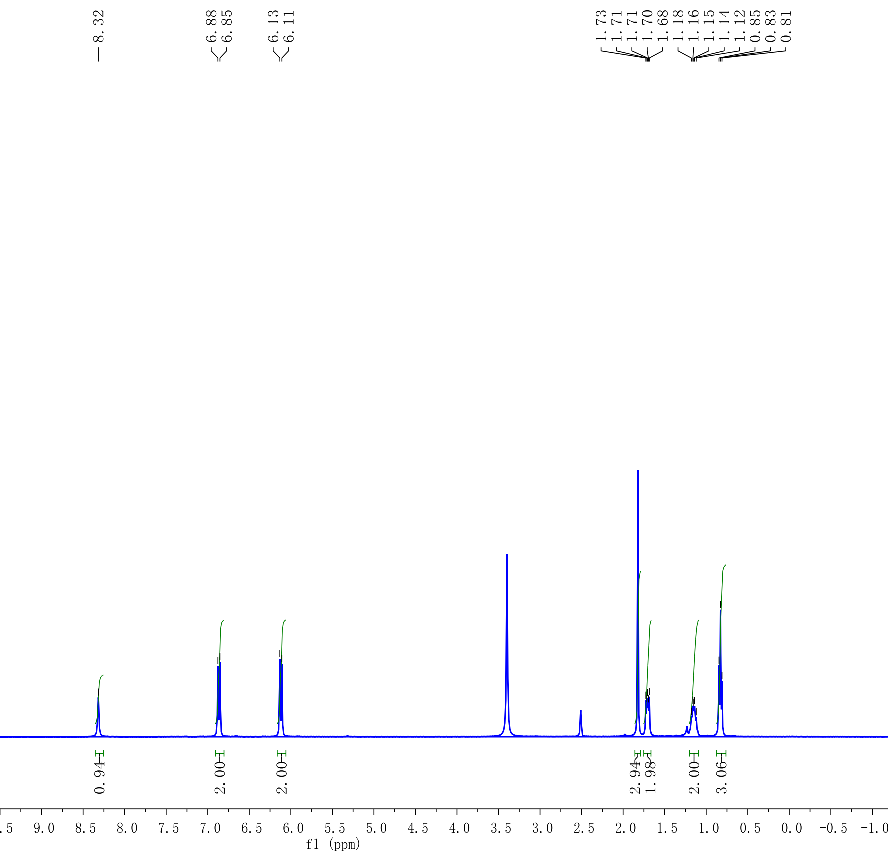

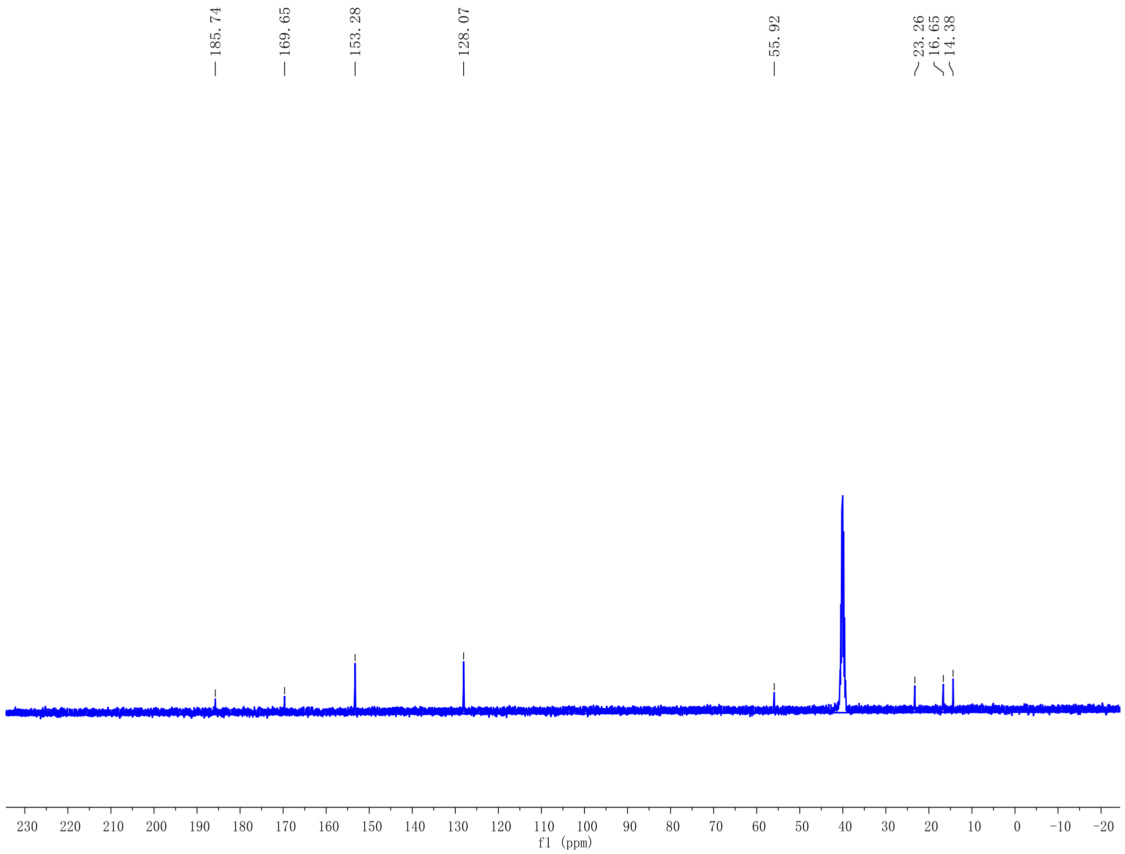

Supplementary Figure 56. ^{1}H and ^{13}C NMR spectra for compound **3e**

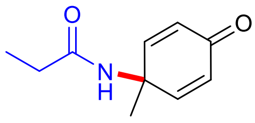

3f

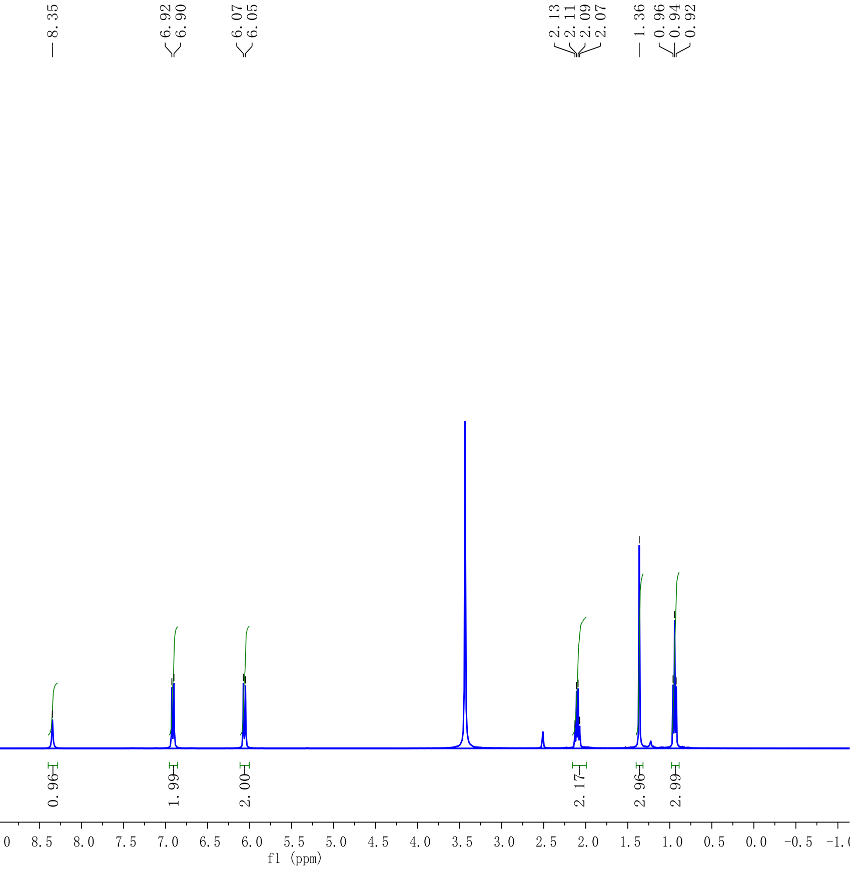

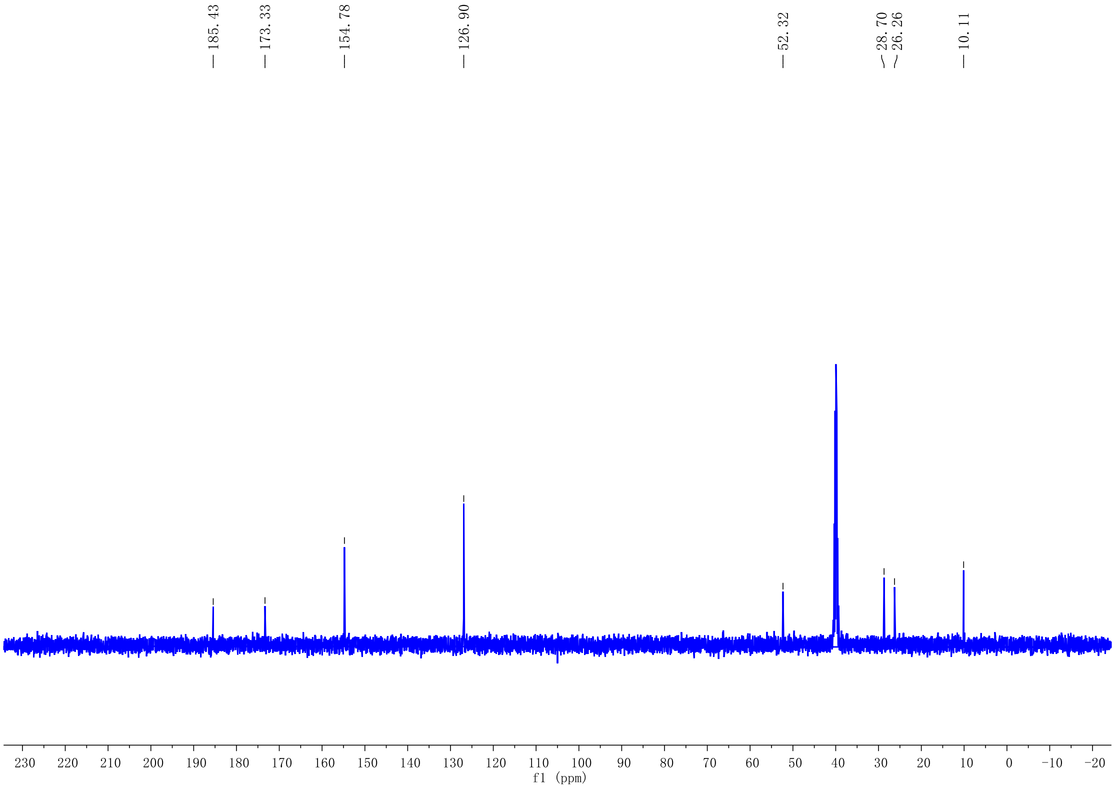

Supplementary Figure 57. ^{1}H and ^{13}C NMR spectra for compound **3f**

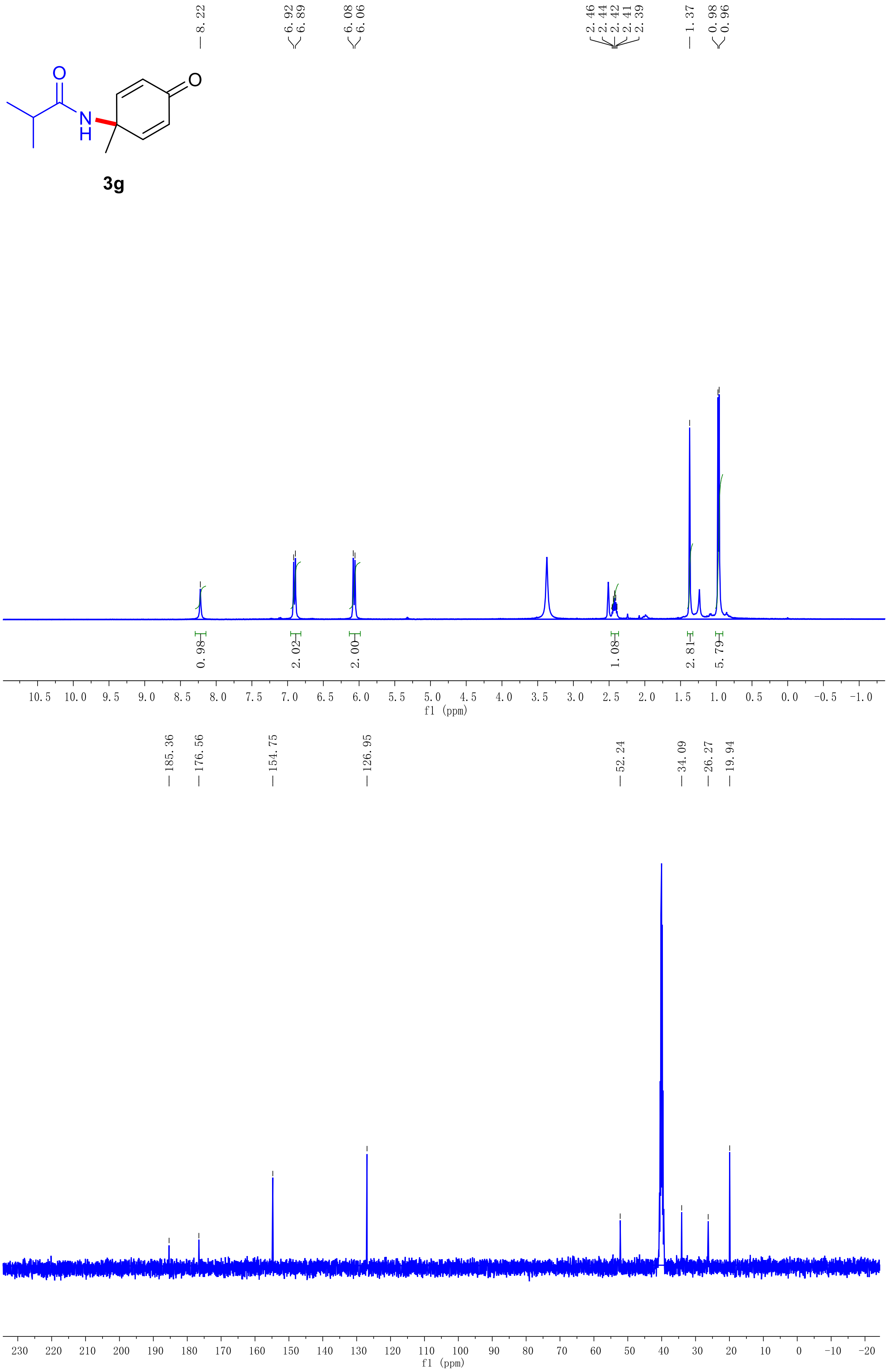

Supplementary Figure 58. ^{1}H and ^{13}C NMR spectra for compound **3g**

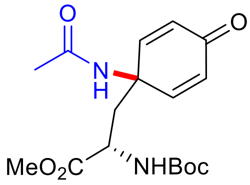

3h

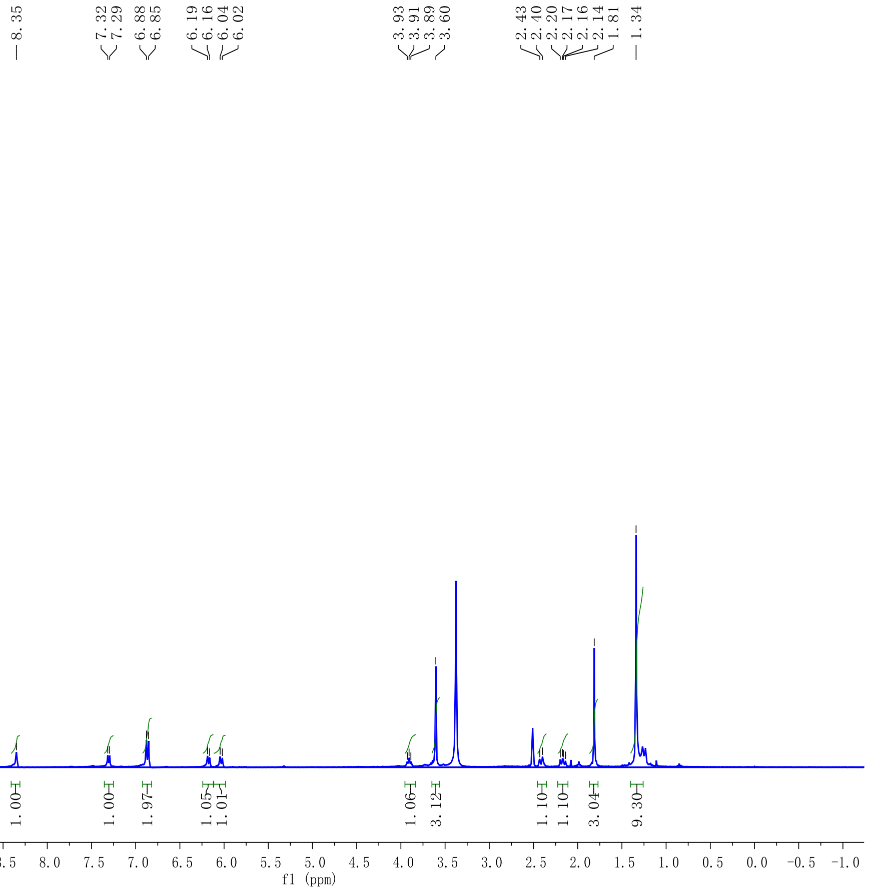

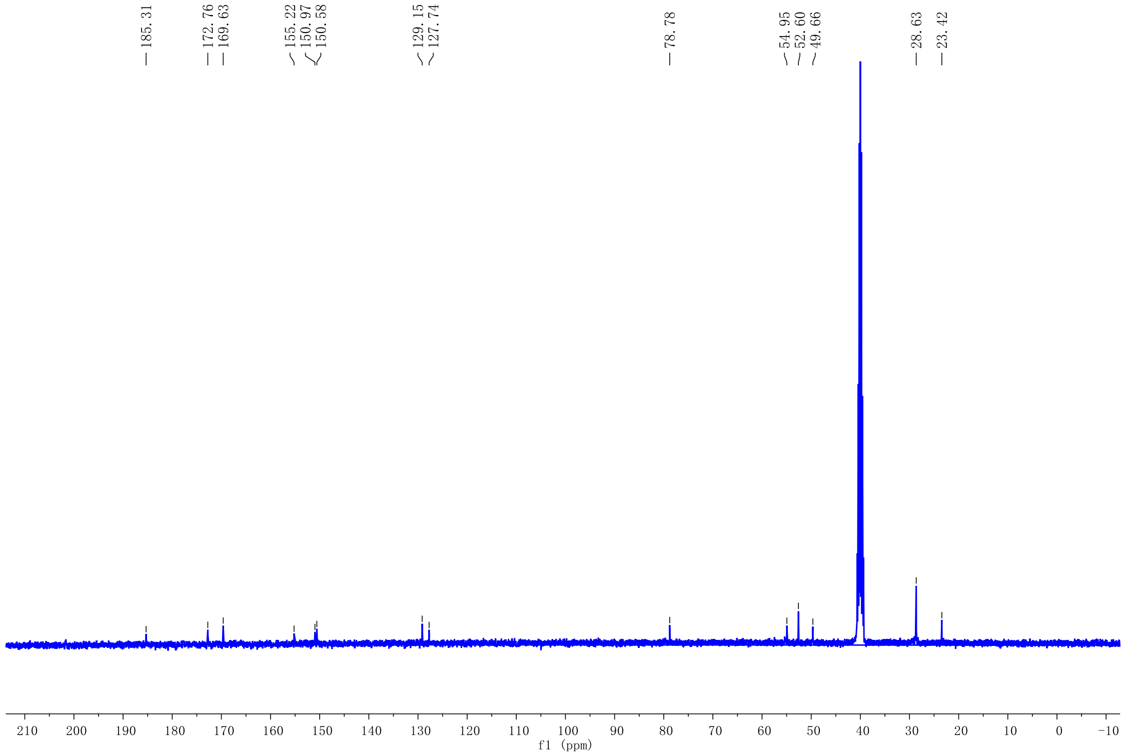

Supplementary Figure 59. ^{1}H and ^{13}C NMR spectra for compound **3h**

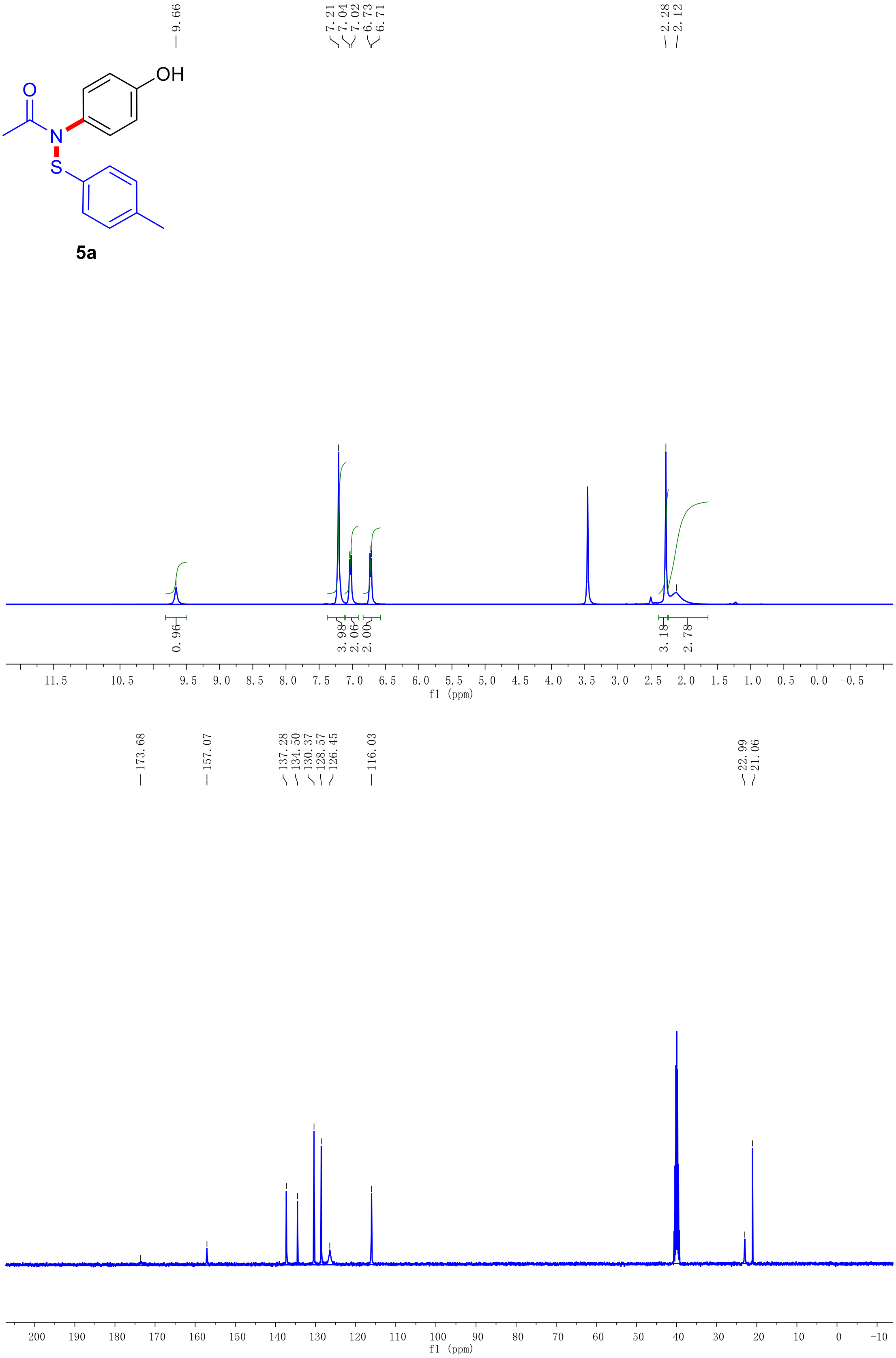

Supplementary Figure 60. ^{1}H and ^{13}C NMR spectra for compound **5a**

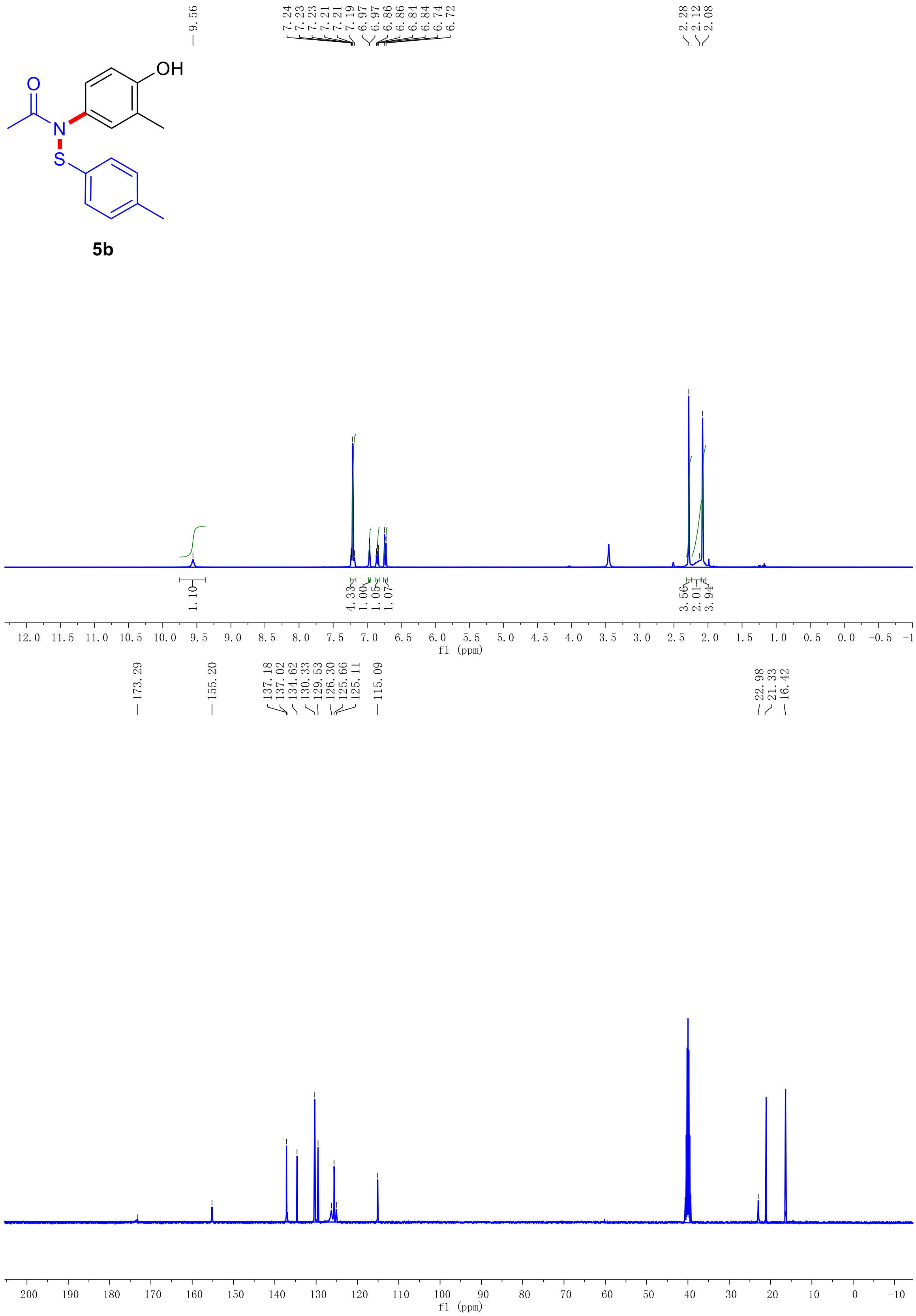

Supplementary Figure 61. ^{1}H and ^{13}C NMR spectra for compound **5b**

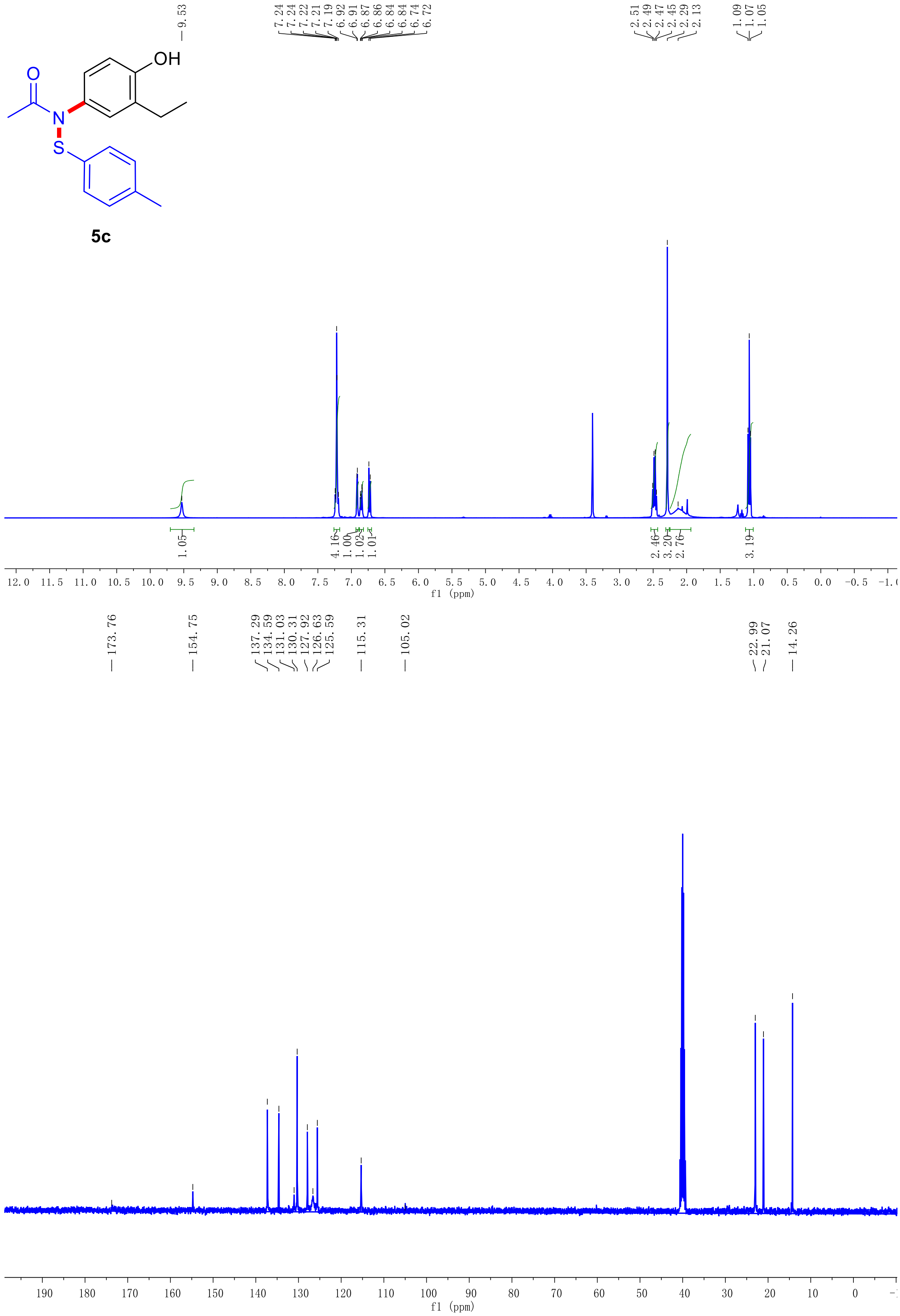

Supplementary Figure 62. ^{1}H and ^{13}C NMR spectra for compound **5c**

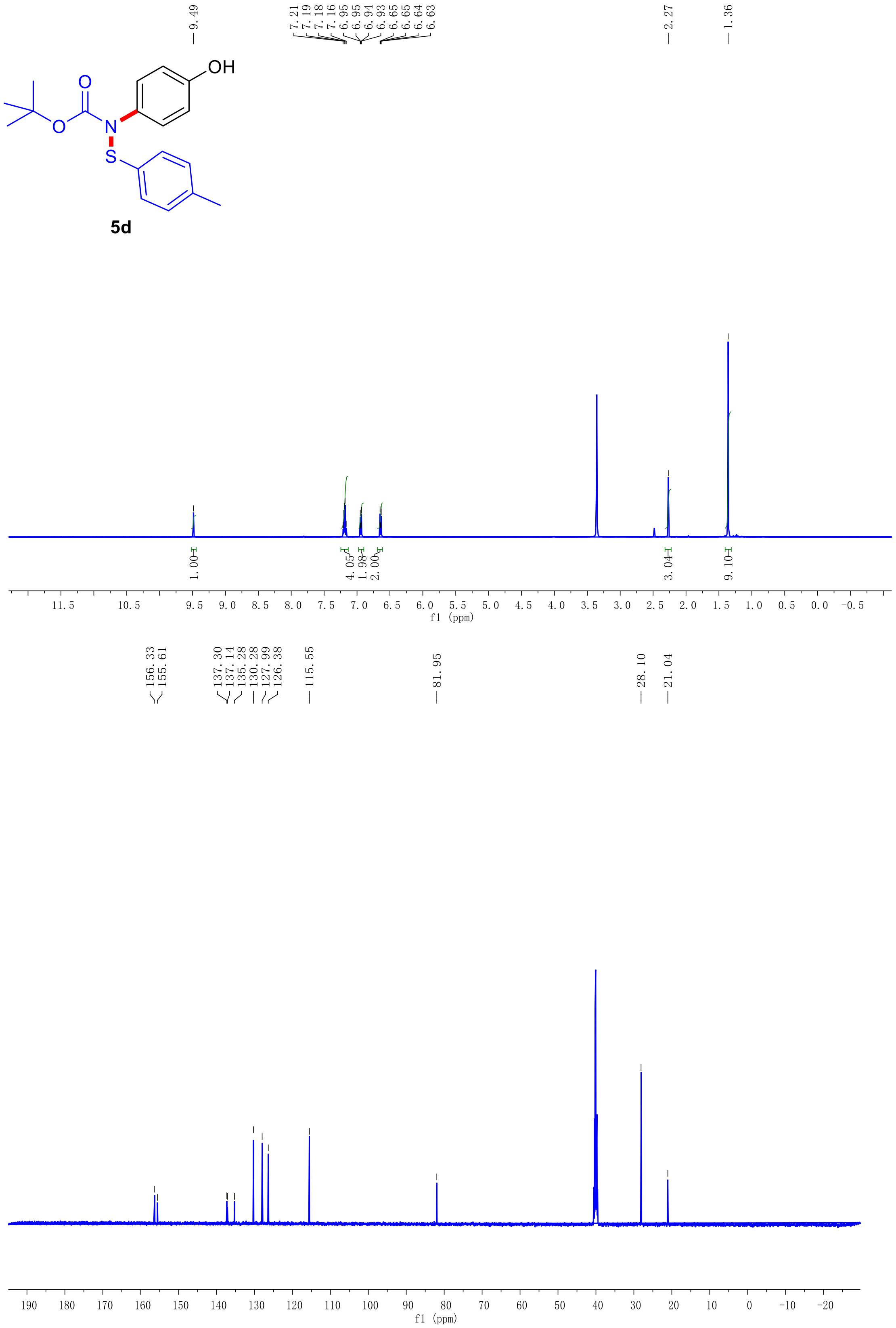

Supplementary Figure 63. ^{1}H and ^{13}C NMR spectra for compound **5d**

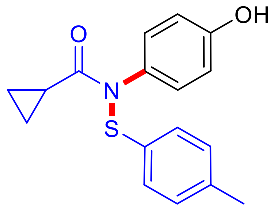

5e

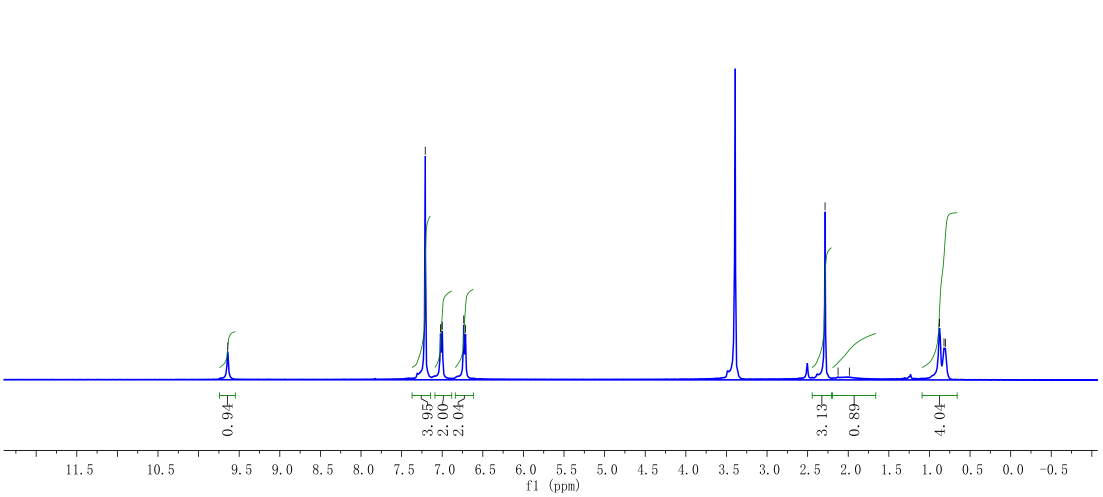

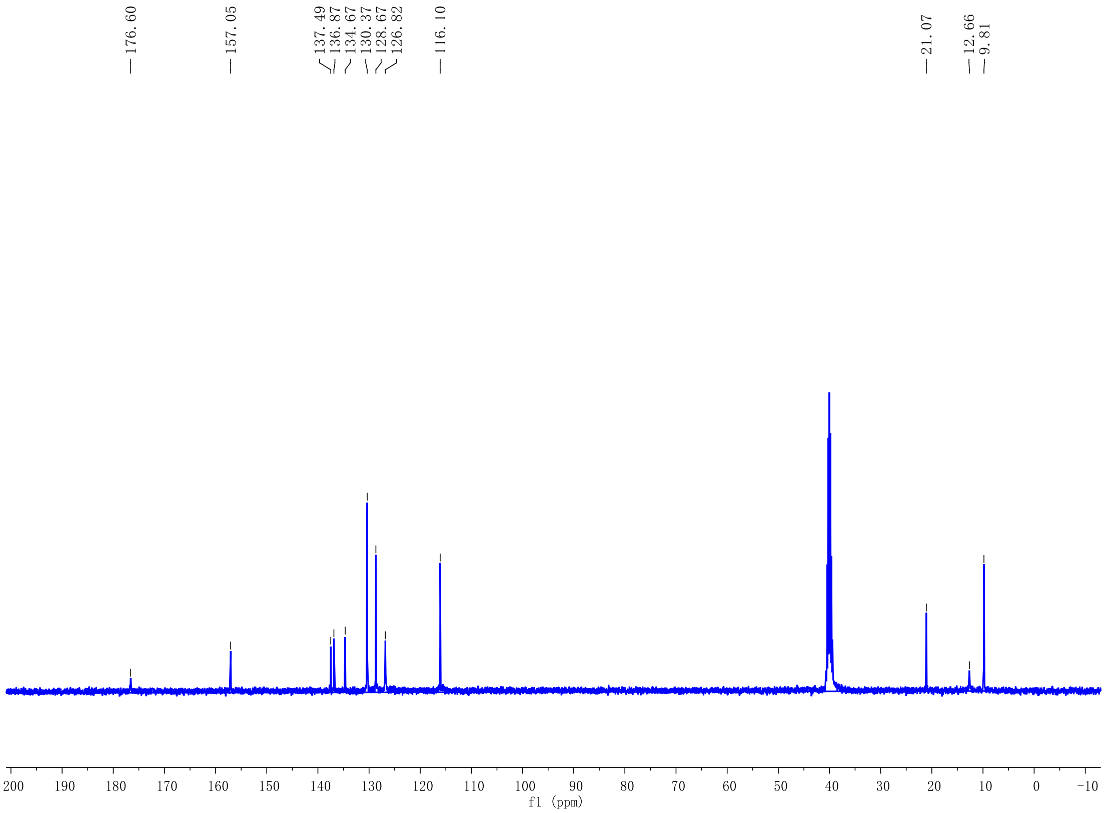

Supplementary Figure 64. ^{1}H and ^{13}C NMR spectra for compound **5e**

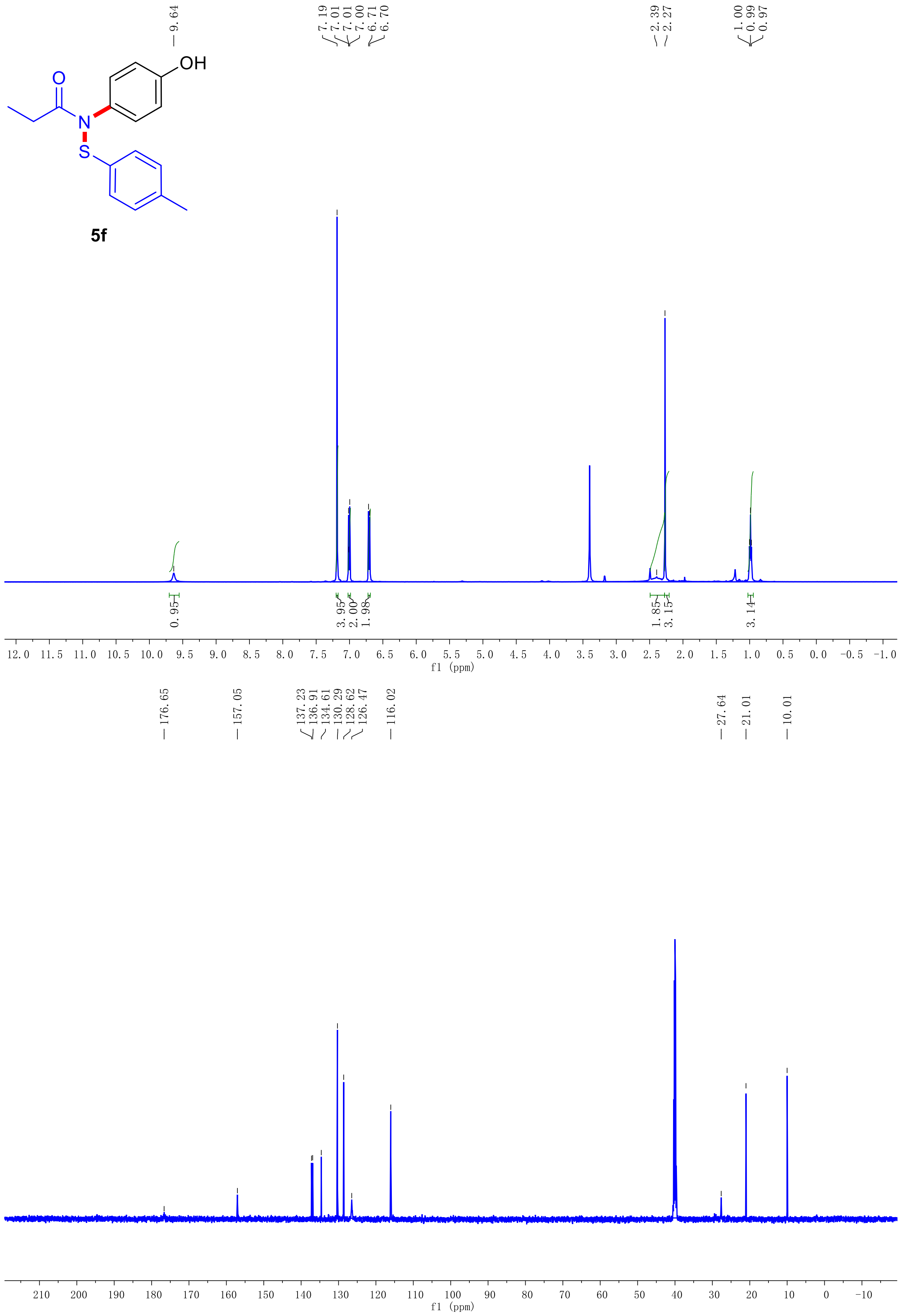

Supplementary Figure 65. ^{1}H and ^{13}C NMR spectra for compound **5f**

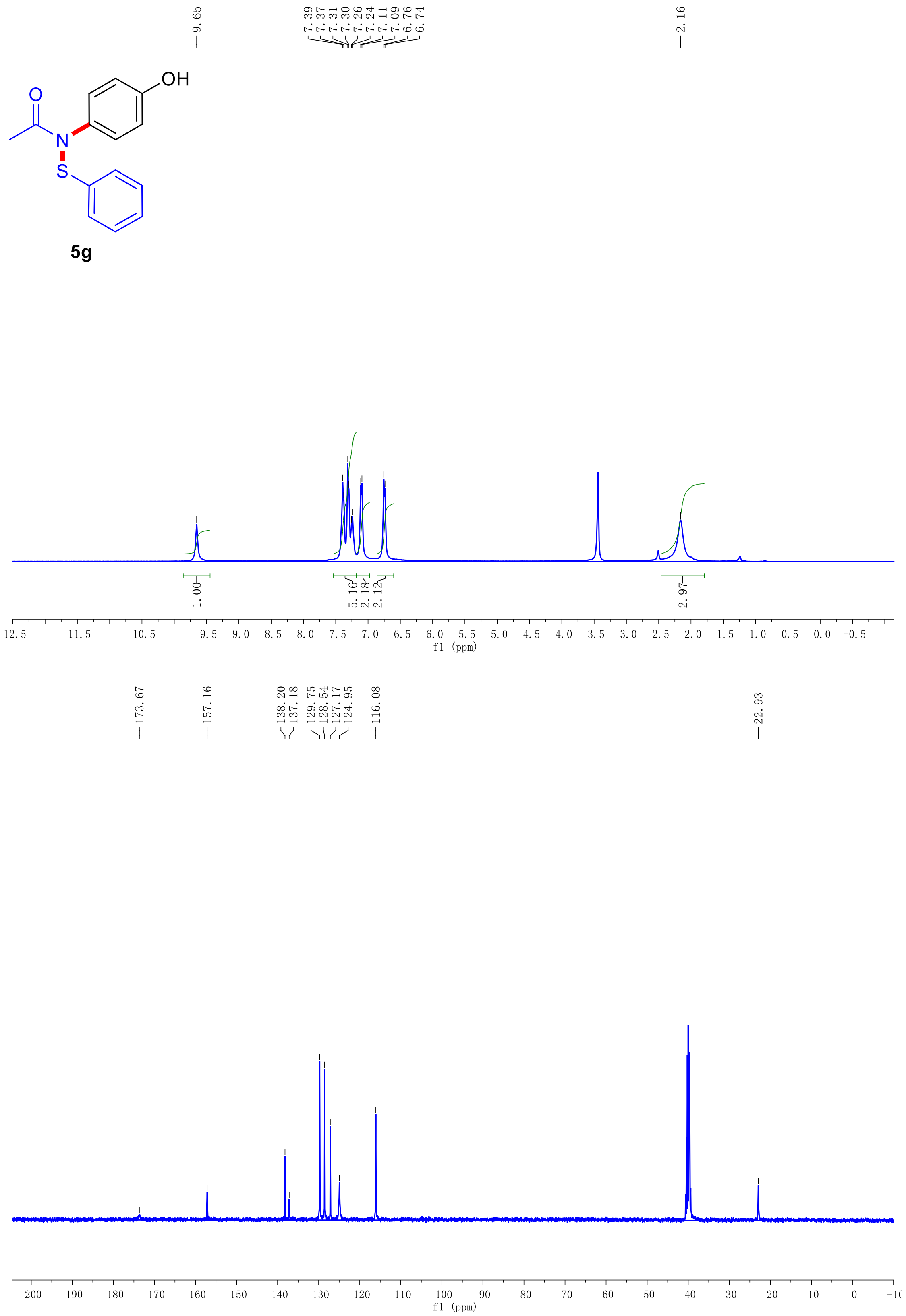

Supplementary Figure 66. ^{1}H and ^{13}C NMR spectra for compound **5g**

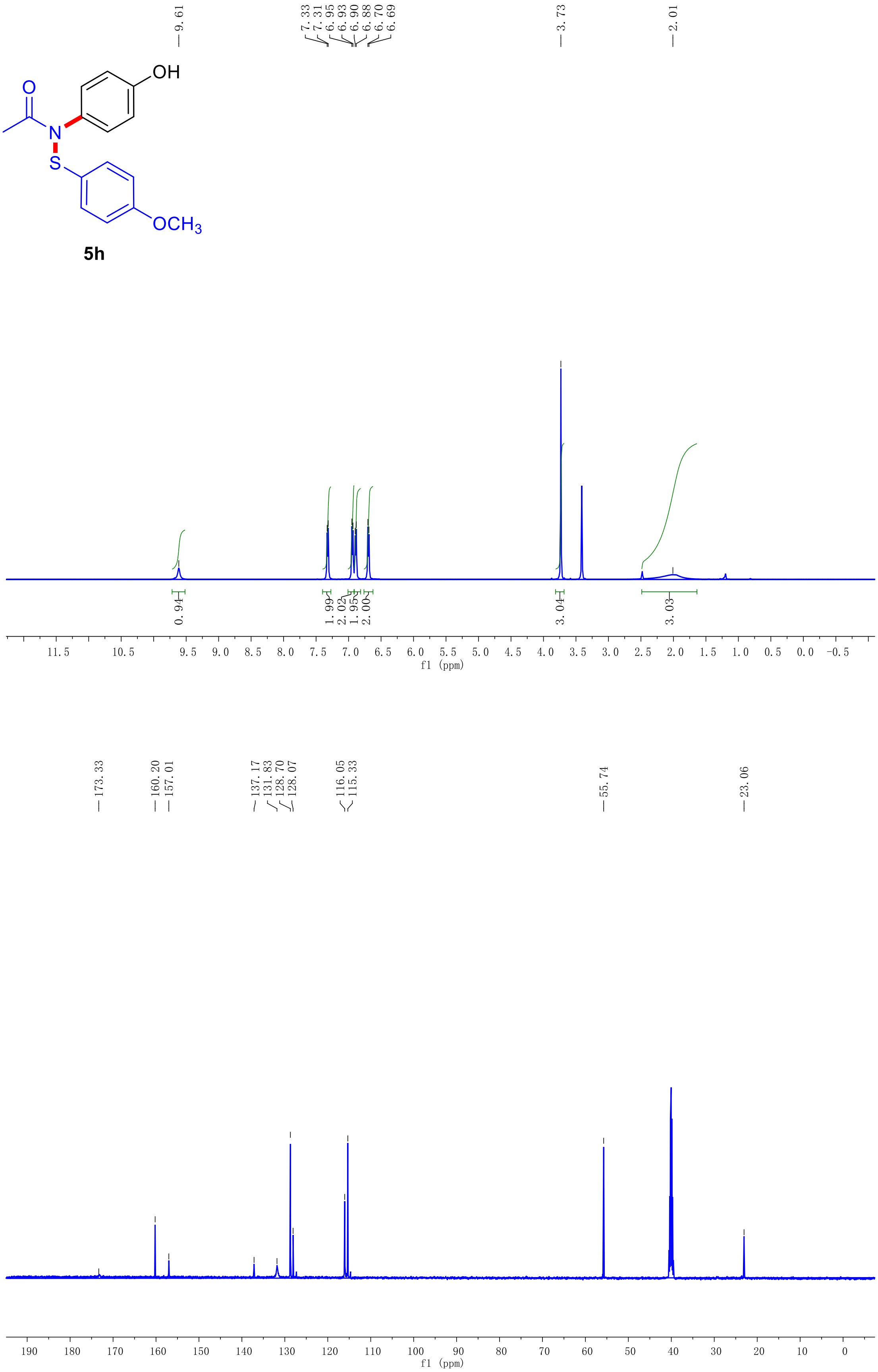

Supplementary Figure 67. ^{1}H and ^{13}C NMR spectra for compound **5h**

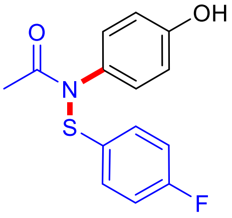
OH
O
N
S
F

5i

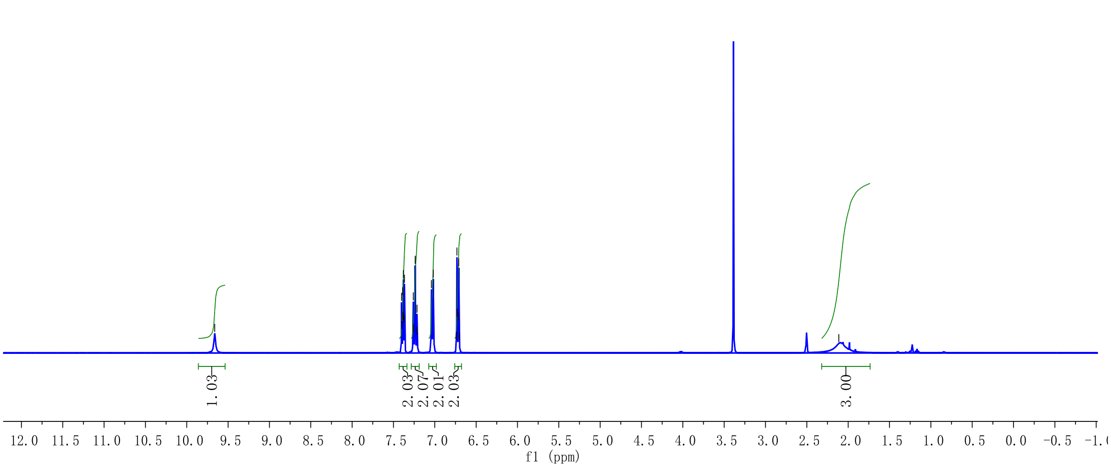
9.66
7.40
7.40
7.39
7.38
7.38
7.37
7.37
7.26
7.25
7.24
7.22
7.22
7.04
7.02
6.74
6.73
6.72
6.71
6.70
2.11
1.03
2.03
2.07
2.01
2.03
3.00
12.0 11.5 11.0 10.5 10.0 9.5 9.0 8.5 8.0 7.5 7.0 6.5 6.0 5.5 5.0 4.5 4.0 3.5 3.0 2.5 2.0 1.5 1.0 0.5 0.0 -0.5
f1 (ppm)

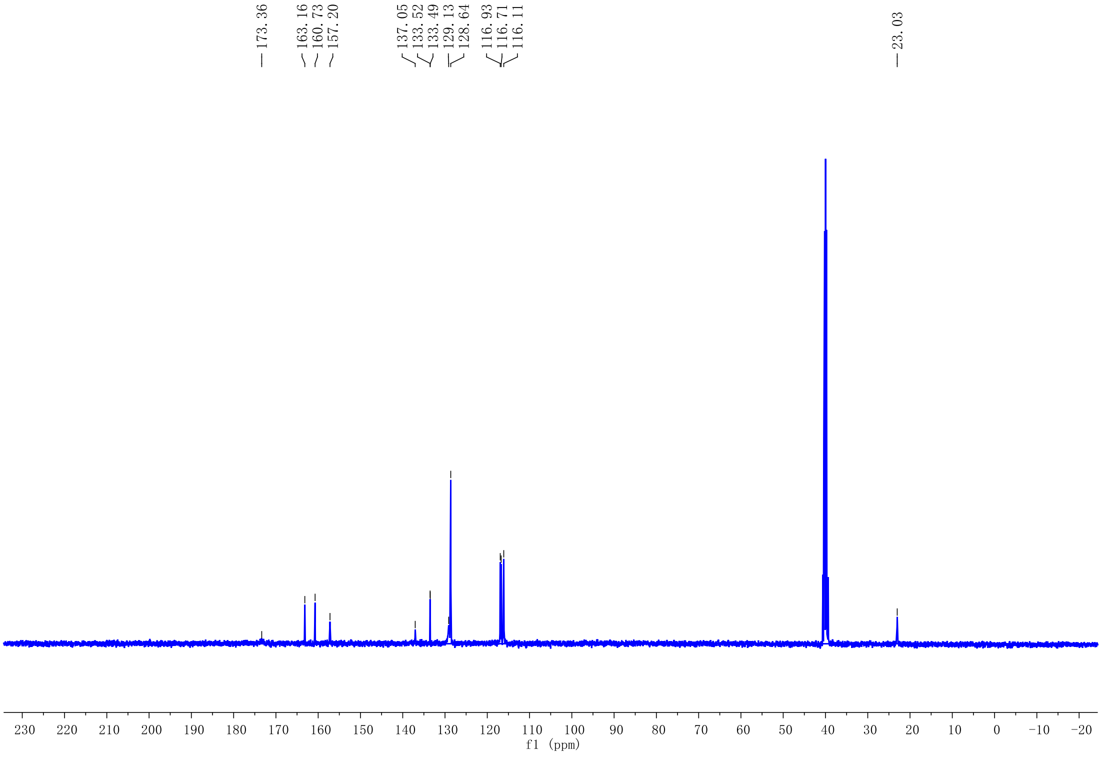
173.36
163.16
160.73
157.20
137.05
133.52
133.49
129.13
128.64
116.93
116.71
116.11
23.03
230 220 210 200 190 180 170 160 150 140 130 120 110 100 90 80 70 60 50 40 30 20 10 0 -10 -20
f1 (ppm)

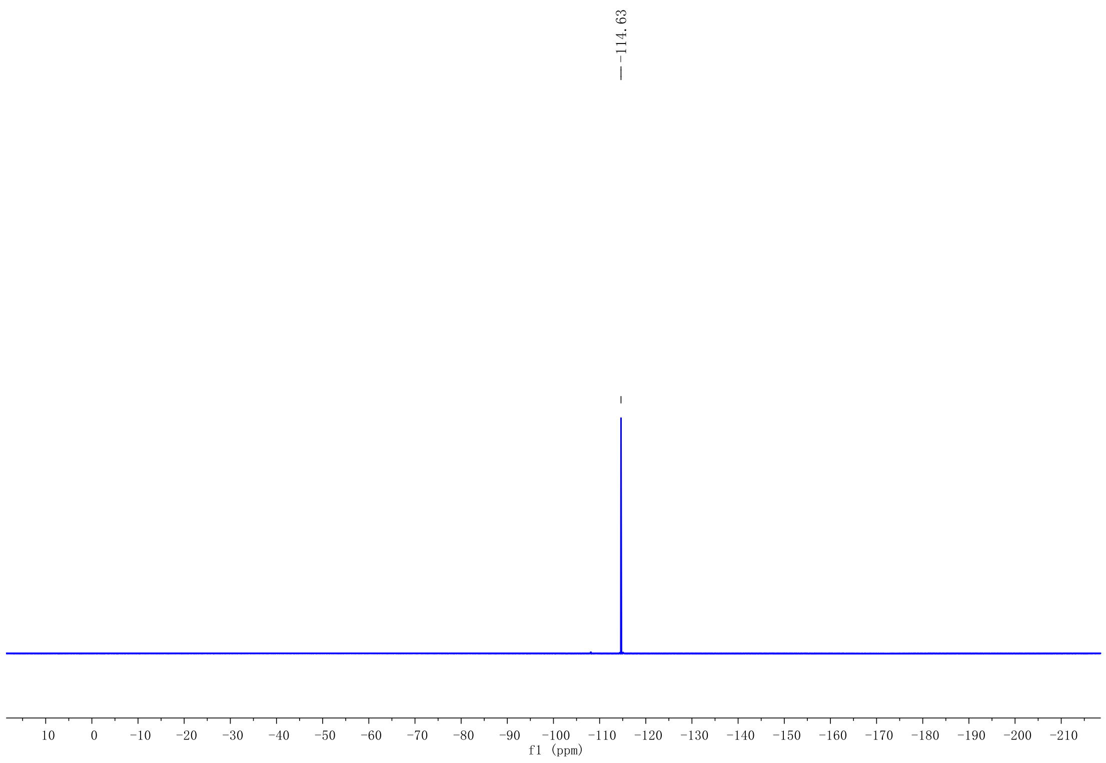

Supplementary Figure 68. ^{1}H, ^{13}C and ^{19}F NMR spectra for compound **5i**

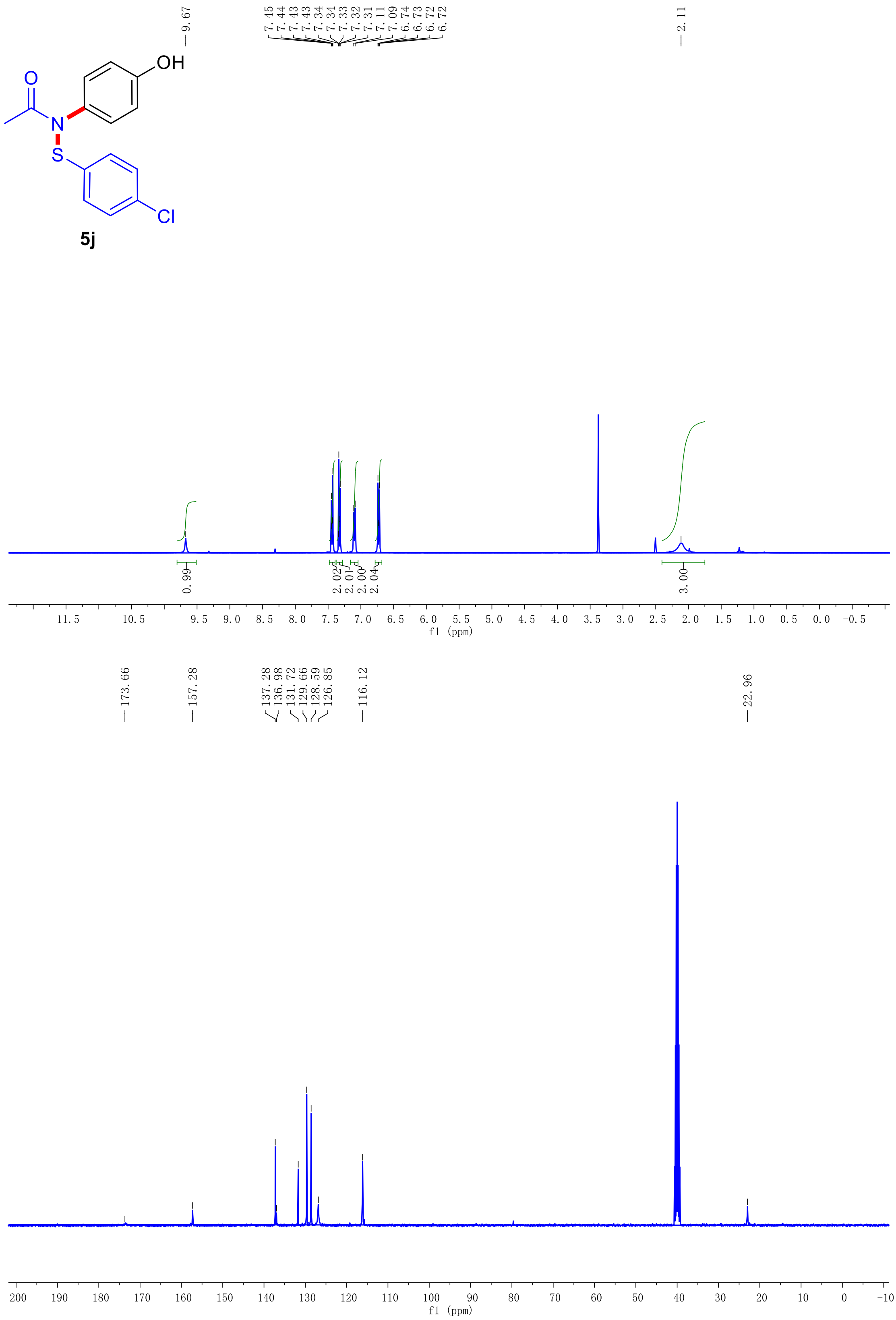

Supplementary Figure 69. ^{1}H and ^{13}C NMR spectra for compound **5j**

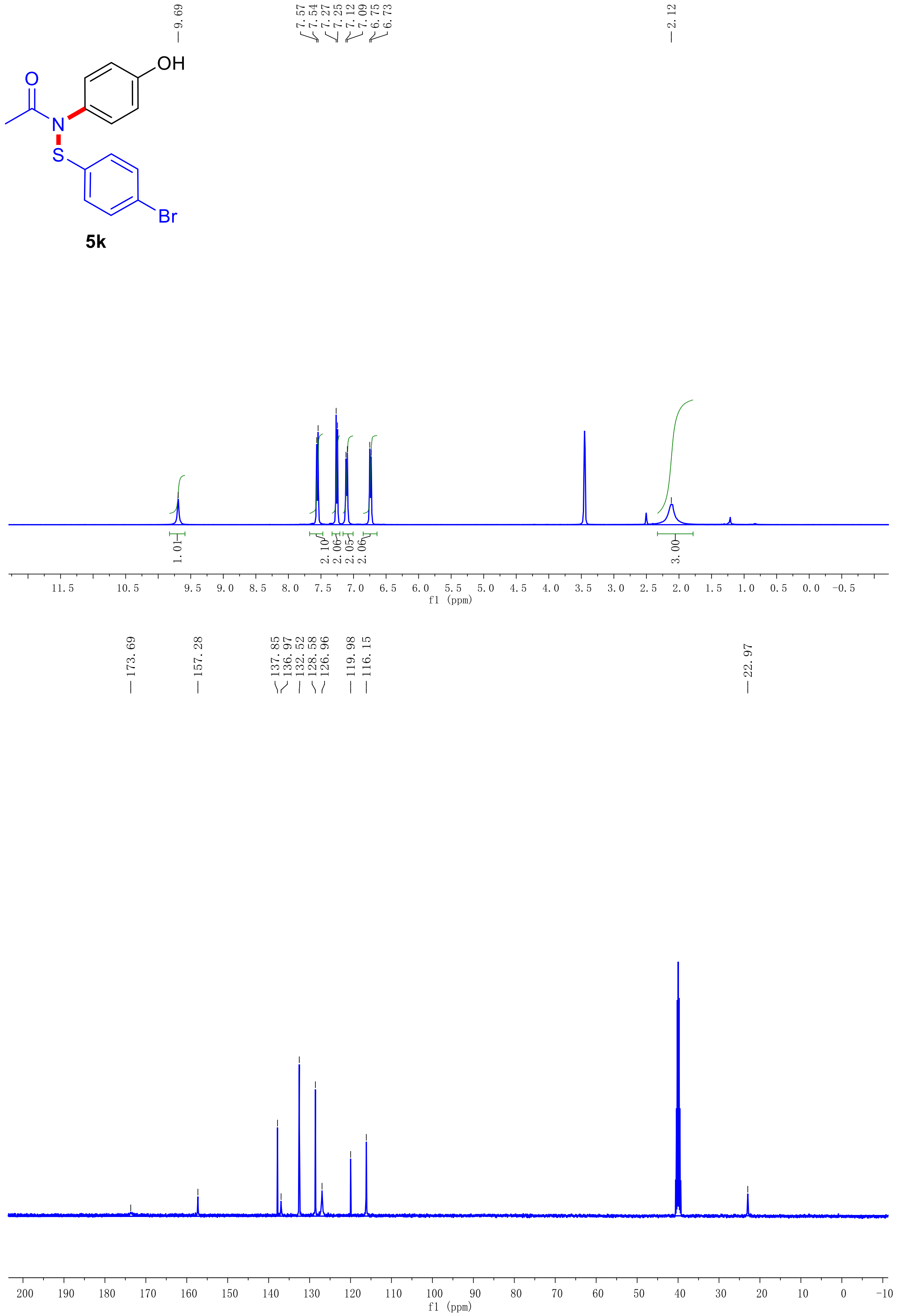

Supplementary Figure 70. ^{1}H and ^{13}C NMR spectra for compound **5k**

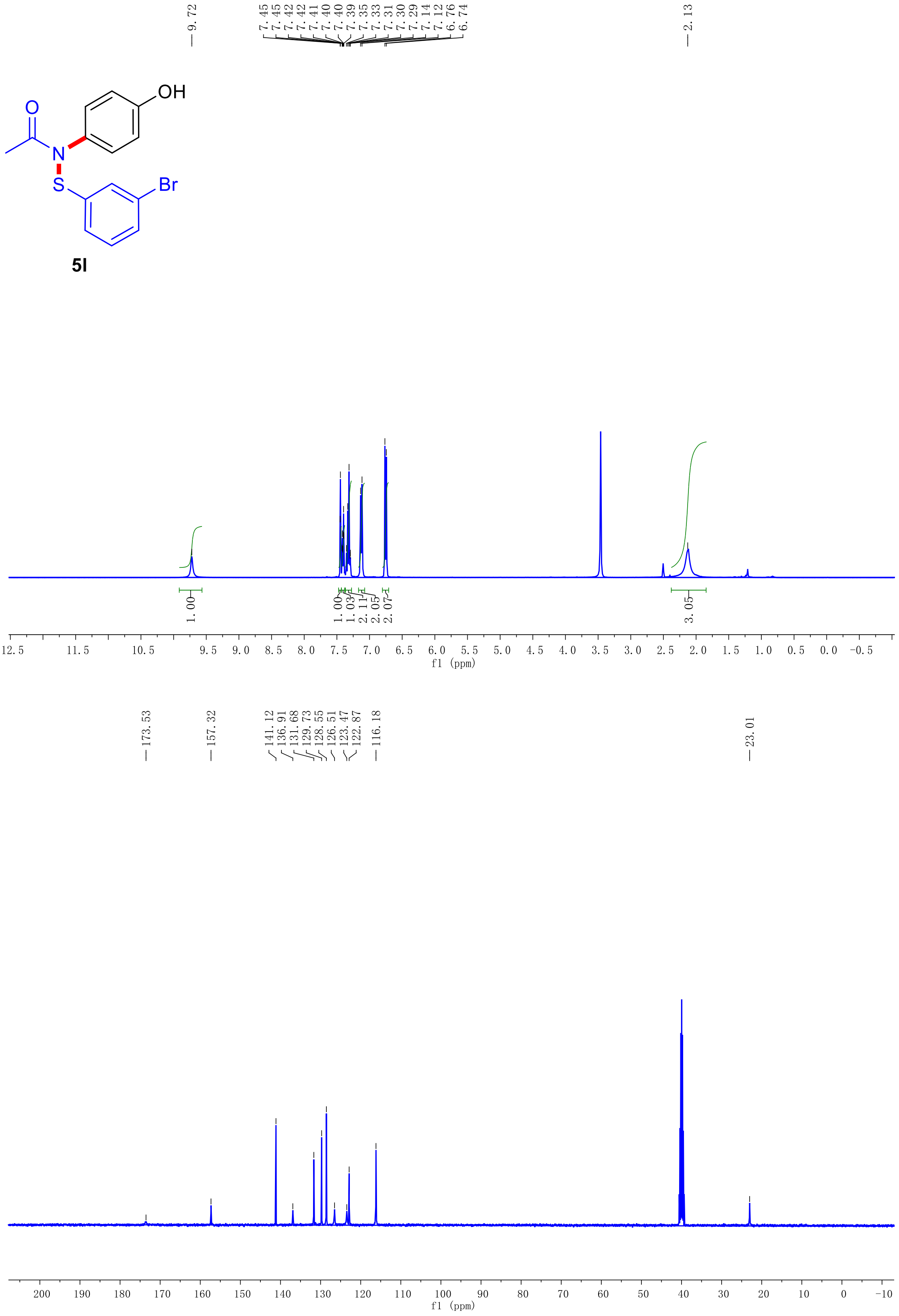

Supplementary Figure 71. ^{1}H and ^{13}C NMR spectra for compound **5l**

5m

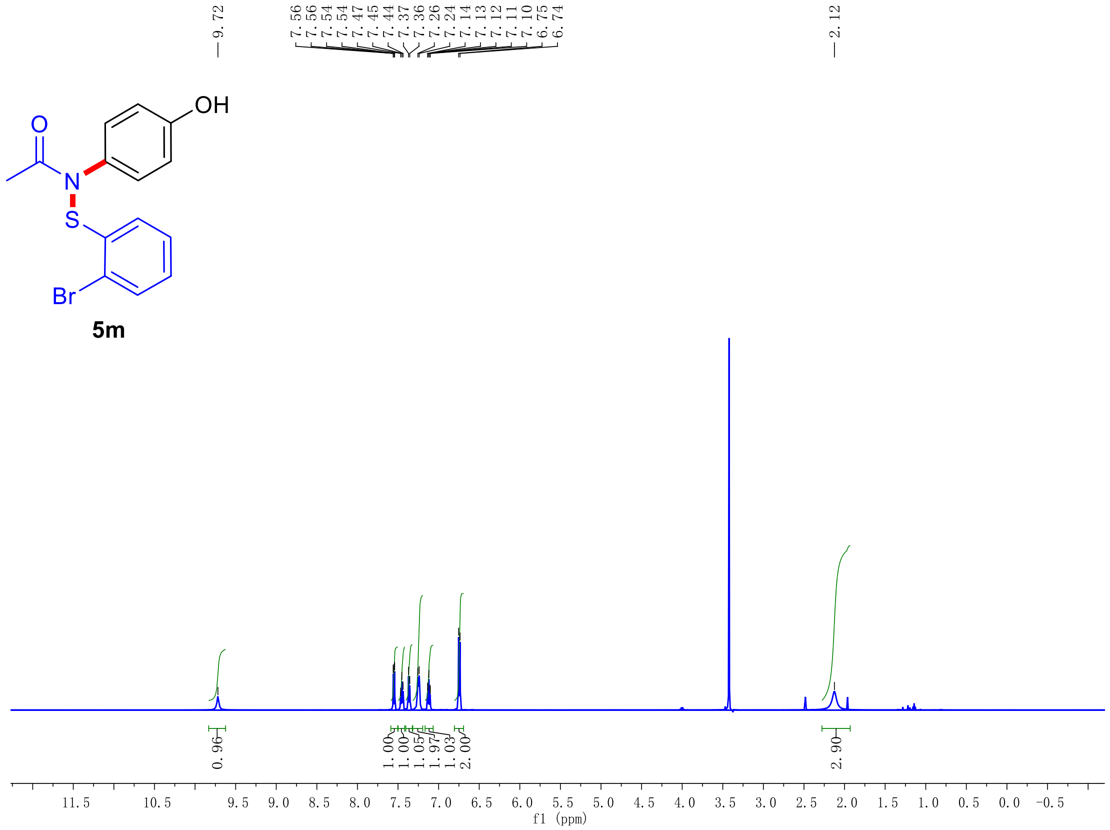

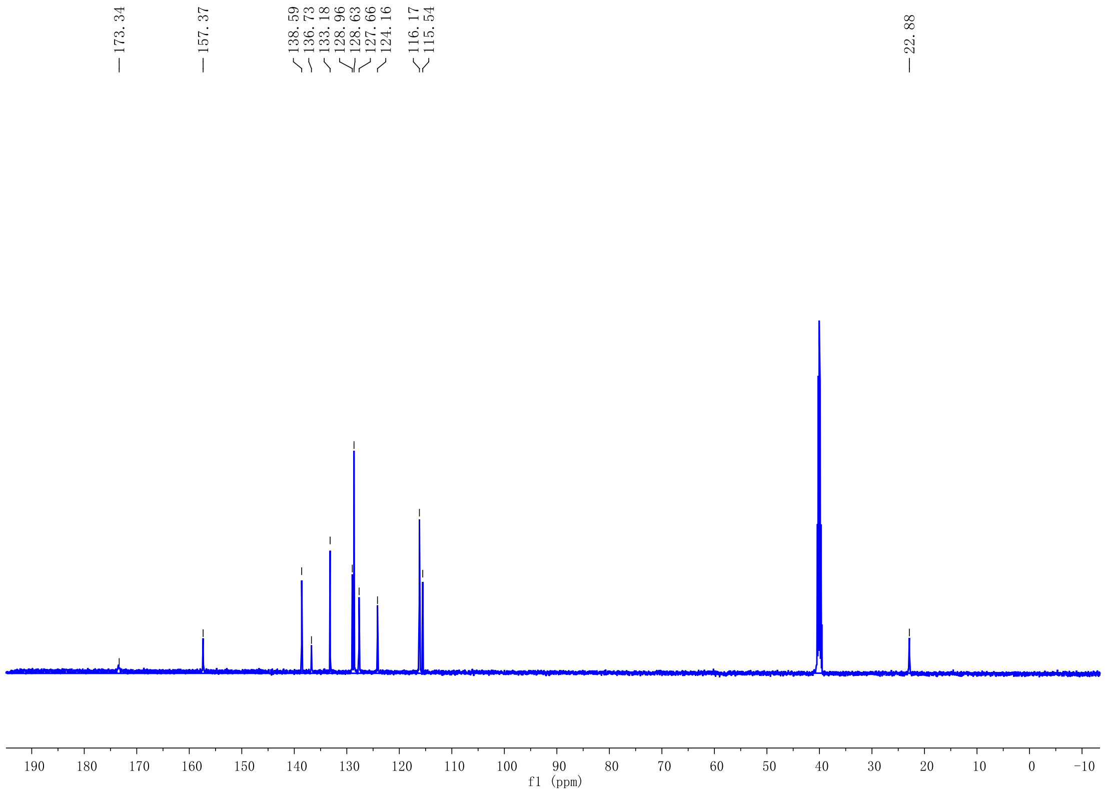

Supplementary Figure 72. ^{1}H and ^{13}C NMR spectra for compound **5m**

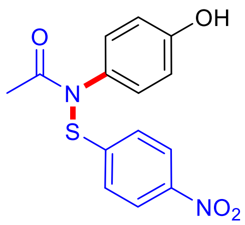

5n

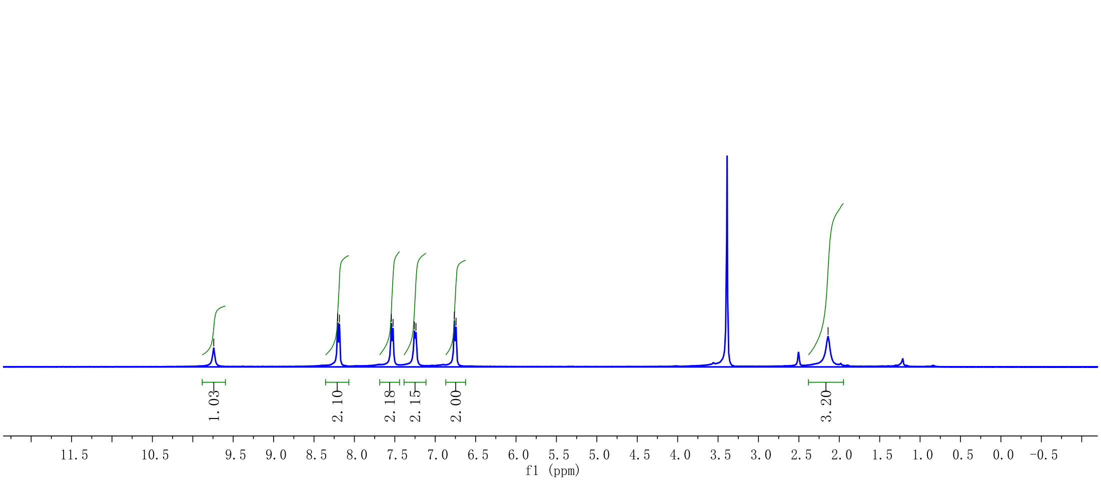

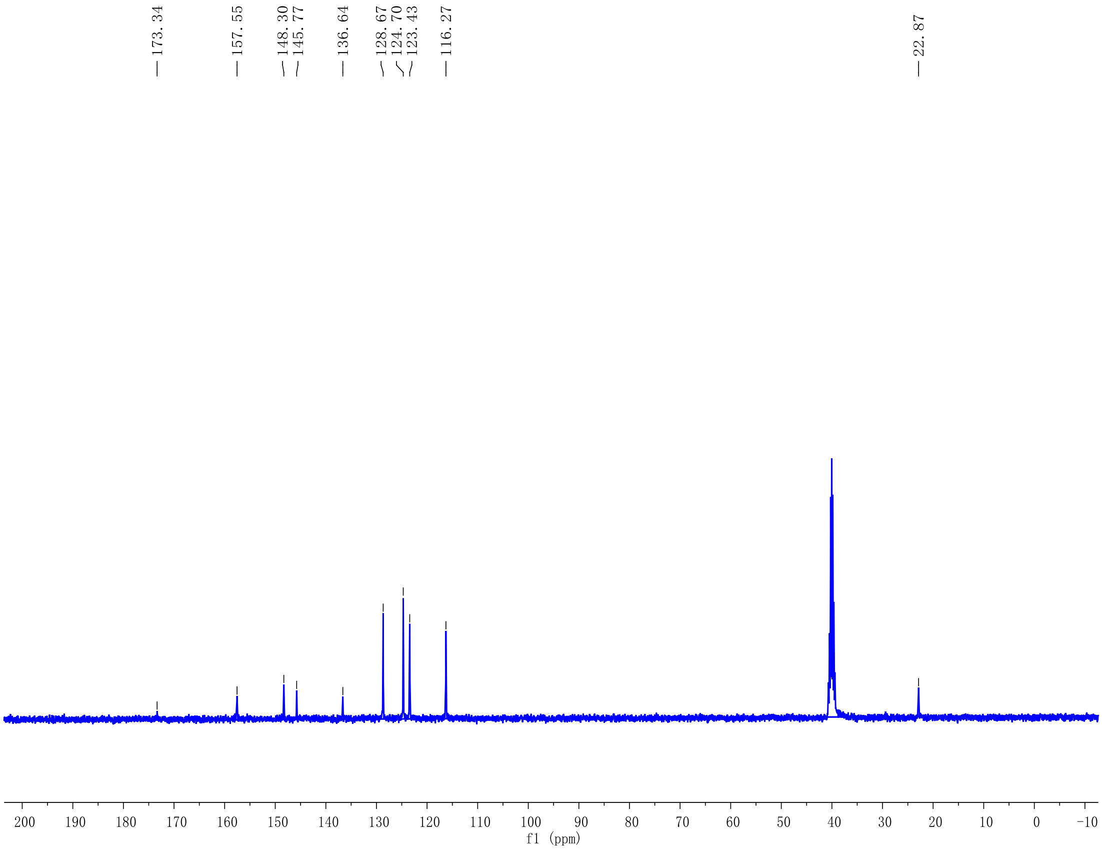

Supplementary Figure 73. ^{1}H and ^{13}C NMR spectra for compound **5n**

Supplementary References

1. Nicolaou, K. C., N. Pktasis., N. A. & ClaiumoL, D. A. *N*-phenylselenophthalimide (NPSP) : A valuable selenenylating agent. *Tetrahedron*, **41**, 4835-4841 (1985).
2. Petrassi, H. M., Sharpless, K. B. & Kelly, J. W. The copper-mediated cross-coupling of phenylboronic acids and *N*-hydroxyphthalimide at room temperature: synthesis of aryloxyamines. *Org. Lett.* **3**, 139-142 (2001).
3. Shen, Y., Liu, G., Zhou, Z. & Lu, X. Rhodium(III)-catalyzed C–H olefination for the synthesis of *ortho*-alkenyl phenols using an oxidizing directing group. *Org. Lett.* **15**, 3366-3369 (2013).
4. Li, B., Lan, J., Wu, D. & You, J. Rhodium(III)-catalyzed *ortho*-heteroarylation of phenols through internal oxidative C–H activation: rapid screening of single-molecular white-light-emitting materials. *Angew. Chem. Int. Ed.* **54**, 14008-14012 (2015).
5. Gillis, H. M., Greene, L. & Thompson, A. Preparation of sulfenyl pyrroles. *Synlett* **1**, 112-116 (2009)
6. Frish, M. J., Trucks, G. W., Schlegel, H. B., Scuseria, G. E., Robb, M. A., Cheeseman, J. R., Scalmani, G., Barone, V., Mennucci, B., Petersson, G. A., Nakatsuji, H., Caricato, M., Li, X., Hratchian, H. P., Izmaylov, A. F., Bloino, J., Zheng, G., Sonnenberg, J. L., Hada, M., Ehara, M., Toyota, K., Fukuda, R., Hasegawa, J., Ishida, M., Nakajima, T., Honda, Y., Kitao, O., Nakai, H., Vreven, T., Montgomery, J. A., Jr., Peralta, J. E., Ogliaro, F., Bearpark, M., Heyd, J. J., Brothers, E., Kudin, K. N., Staroverov, V. N., Keith, T., Kobayashi, R., Normand, J., Raghavachari, K., Rendell, A., Burant, J. C., Iyengar, S. S., Tomasi, J., Cossi, M., Rega, N., Millam, J. M., Klene, M., Knox, J. E., Cross, J. B., Bakken, V., Adamo, C., Jaramillo, J., Gomperts, R., Stratmann, R. E., Yazyev, O., Austin, A. J., Cammi, R., Pomelli, C., Ochterski, J. W., Martin, R. L., Morokuma, K., Zakrzewski, V. G., Voth, G. A., Salvador, P., Dannenberg, J. J., Dapprich, S., Daniels, A. D., Farkas, O., Foresman, J. B., Ortiz, J. V., Cioslowski, J. & Fox, D. J. *Gaussian 09, Rev. D.01*, Gaussian, Inc.: Wallingford, CT, 2010.
7. Becke, A. D. Density-functional exchange-energy approximation with correct asymptotic behavior. *Phys. Rev. A: Gen. Phys.* **38**, 3098-3100 (1988);
8. Becke, A. D. Density-functional thermochemistry. III. The role of exact exchange. *J. Chem. Phys*. **98**, 5648-5652 (1993);
9. Grimme, S., Antony, J., Ehrlich, S. & Krieg, H. *J. Chem. Phys.* 2010, **132**, 154104 (2010)
10. Hay, P. J. & Wadt, W. R. *J. Chem. Phys.* 1985, **82**, 299 (1985)
11. Tomasi, J. & Persico, M. Molecular interactions in solution: an overview of methods based on continuous distributions of the solvent. *Chem. Rev.* **94**, 2027-2094 (1994).
