# Supplementary material for: A selenium-catalysed para-amination of phenols
Source: Nat Commun. 2018 Oct 16;9:4293. doi: 10.1038/s41467-018-06763-4 (PMC6191425; doi:10.1038/s41467-018-06763-4)
Supplement: Supplementary file 3 — Supplementary Data 1 [file 41467_2018_6763_MOESM3_ESM.pdf]

Description of Additional Supplementary Files

File Name: Supplementary Data 1
Description: Theoretical Calculations
