# Supplementary material for: A selenium-catalysed para-amination of phenols
Source: Nat Commun. 2018 Oct 16;9:4293. doi: 10.1038/s41467-018-06763-4 (PMC6191425; doi:10.1038/s41467-018-06763-4)
Supplement: Supplementary file 4 — Supplementary Tables 16 and 17 [file 41467_2018_6763_MOESM4_ESM.pdf]

Supplementary Table 16. Calculated electronic energies of S-mediated reaction in 2,2,2-trifluoroethanol (TFE) at the B3LYP-D3/BS1 level (E_1, in a.u.), calculated Gibbs free energies in TFE at the B3LYP-D3/BS1 level (G_1, in a.u.), calculated electronic energies in TFE at the B3LYP-D3/BS2 level (E_2, in a.u.), corrected free energies ($G_{correct}$, in a.u.) for all stationary points.

Species	G_1	E_1	E_2	$G_{correct}$
**1a**	-515.35704	-515.47846	-515.63812	-515.51671
**4g**	-1142.25001	-1142.40324	-1142.6498	-1142.49659
2,6-lutidine	-326.85751	-326.97042	-327.06132	-326.94841
**1a′**	-514.87327	-514.98199	-515.15884	-515.05011
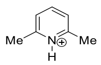	-327.30683	-327.43211	-327.51603	-327.39074
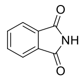	-513.06157	-513.14487	-513.30282	-513.21953
**TS$_{SN1}$**	-1657.11343	-1657.39952	-1657.81299	-1657.52690
**INT1**-S	-1144.54853	-1144.74199	-1144.99001	-1144.79655
**TS1**-S	-1144.52872	-1144.72192	-1144.97418	-1144.78098
**INT2**-S	-1144.54280	-1144.73629	-1144.99434	-1144.80085
**TS2**-S	-1144.53508	-1144.72803	-1144.98626	-1144.79330
**INT3**-S	-1144.57262	-1144.76905	-1145.02166	-1144.82523
**5g**	-1144.61126	-1144.80326	-1145.05579	-1144.8638
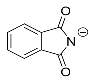	-512.57768	-512.64804	-512.82482	-512.75447
**TS$_{SN2}$**	-1657.16958	-1657.45410	-1657.87398	-1657.58946

**4a**	-515.42528	-515.54738	-515.71014	-515.58804
**TS2′-S**	-1471.3883	-1471.71201	-1472.05404	-1471.73037
**5g′**	-1144.57564	-1144.76761	-1145.02606	-1144.83409

1a

Center Number	Atomic Number	Atomic Type	Coordinates (in Å) X	Y	Z
1	6	0	-3.470531	-0.101513	-1.165843
2	6	0	-2.298284	0.014001	-0.416173
3	6	0	-2.179331	-0.576697	0.839197
4	6	0	-3.262982	-1.299885	1.345216
5	6	0	-4.442438	-1.428867	0.612511
6	6	0	-4.540093	-0.825770	-0.644954
7	1	0	-3.527710	0.373868	-2.139110
8	1	0	-1.261234	-0.474577	1.402824
9	1	0	-3.176065	-1.763433	2.322973
10	1	0	-5.277740	-1.992287	1.014757
11	1	0	-5.452587	-0.917956	-1.225468
12	8	0	-1.295806	0.764652	-1.024940
13	7	0	-0.111715	0.847651	-0.278273
14	1	0	0.636453	0.314729	-0.710969
15	6	0	0.224647	2.085839	0.239406
16	6	0	-0.916723	3.053793	0.425451
17	1	0	-1.318280	3.353840	-0.546584
18	1	0	-1.733048	2.591040	0.986715
19	1	0	-0.546514	3.928398	0.958642
20	8	0	1.385538	2.301765	0.562133

4g

Center Number	Atomic Number	Atomic Type	Coordinates (in Å) X	Y	Z
1	6	0	-6.020923	-1.507346	0.253753
2	6	0	-6.558600	-0.336666	0.796923
3	6	0	-7.896309	-0.250016	1.151894
4	6	0	-8.691559	-1.384696	0.946024
5	6	0	-8.152907	-2.557269	0.401842
6	6	0	-6.800657	-2.634935	0.044926
7	6	0	-4.578548	-1.283767	-0.028775
8	6	0	-5.482428	0.685599	0.886357

9	1	0	-8.306822	0.661690	1.571258
10	1	0	-9.743019	-1.356073	1.211178
11	1	0	-8.795351	-3.418718	0.254034
12	1	0	-6.376749	-3.538669	-0.378351
13	8	0	-5.543646	1.833211	1.273065
14	8	0	-3.769415	-2.036364	-0.529082
15	7	0	-4.313081	0.042285	0.402001
16	16	0	-2.779665	0.812480	0.262451
17	6	0	-2.921680	1.533397	-1.372336
18	6	0	-2.645817	0.754817	-2.503163
19	6	0	-3.298085	2.874689	-1.505705
20	6	0	-2.754010	1.323696	-3.771457
21	1	0	-2.362955	-0.284708	-2.381411
22	6	0	-3.395656	3.438082	-2.778242
23	1	0	-3.519209	3.460851	-0.620731
24	6	0	-3.125188	2.663759	-3.908411
25	1	0	-2.545143	0.723573	-4.651220
26	1	0	-3.686825	4.477877	-2.886686
27	1	0	-3.204019	3.104470	-4.897154

2,6-lutidine

Center Number	Atomic Number	Atomic Type	Coordinates (in Å) X	Y	Z
1	6	0	1.913685	0.269404	0.000282
2	6	0	2.648458	1.460494	-0.000302
3	6	0	1.963098	2.672326	-0.001142
4	6	0	0.570984	2.660104	-0.001288
5	6	0	-0.093325	1.428274	-0.000566
6	1	0	3.733238	1.431025	-0.000092
7	1	0	2.506101	3.612611	-0.001664
8	1	0	0.003116	3.584868	-0.001963
9	7	0	0.568645	0.257412	0.000206
10	6	0	2.608677	-1.069121	0.001830
11	1	0	3.253053	-1.180273	-0.877018
12	1	0	3.246558	-1.181500	0.885291
13	1	0	1.869531	-1.871814	-0.001286
14	6	0	-1.600036	1.361292	-0.000977
15	1	0	-2.017519	1.863029	0.878753
16	1	0	-2.016809	1.858859	-0.883426
17	1	0	-1.926007	0.320004	0.001281

1a′

Center Atomic Atomic Coordinates (in Å)

Number	Number	Type	X	Y	Z
1	6	0	-4.273319	0.002644	0.955149
2	6	0	-3.550229	-0.000397	-0.250055
3	6	0	-4.252373	-0.003979	-1.471393
4	6	0	-5.643130	-0.004499	-1.483474
5	6	0	-6.367778	-0.001479	-0.285767
6	6	0	-5.669292	0.002068	0.922689
7	1	0	-3.728441	0.005388	1.888358
8	1	0	-3.683324	-0.006306	-2.396112
9	1	0	-6.165875	-0.007286	-2.436184
10	1	0	-7.453158	-0.001891	-0.297510
11	1	0	-6.216303	0.004444	1.862229
12	8	0	-2.205928	-0.000187	-0.343975
13	7	0	-1.535860	0.003710	0.966884
14	6	0	-0.223523	0.003252	0.743317
15	6	0	0.543394	0.006967	2.070182
16	1	0	-0.115702	0.009327	2.941674
17	1	0	1.193304	-0.874131	2.116301
18	1	0	1.193161	0.888411	2.111426
19	8	0	0.419885	0.000059	-0.337964

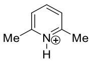

Center Number	Atomic Number	Atomic Type	Coordinates (in Å) X	Y	Z
1	6	0	-1.717004	2.302368	1.639257
2	6	0	-0.553890	3.058330	1.604445
3	6	0	0.038509	3.355569	0.377959
4	6	0	-0.528703	2.896567	-0.809837
5	6	0	-1.690214	2.138592	-0.766883
6	1	0	-0.124517	3.407619	2.534282
7	1	0	0.945917	3.948208	0.347854
8	1	0	-0.080396	3.119428	-1.769212
9	7	0	-2.225843	1.880468	0.453428
10	6	0	-2.448715	1.923112	2.885619
11	1	0	-2.510782	0.834745	2.981214
12	1	0	-1.934453	2.321585	3.759387
13	1	0	-3.468985	2.318876	2.869624
14	6	0	-2.387800	1.581543	-1.965118
15	1	0	-3.432744	1.904741	-1.992001
16	1	0	-1.894259	1.917244	-2.876398
17	1	0	-2.373022	0.487067	-1.943019
18	1	0	-3.079362	1.328139	0.480452

TS$_{SN1}$

Center Number	Atomic Number	Atomic Type	Coordinates (in Å) X	Y	Z
1	6	0	-4.676327	2.007029	1.892163
2	6	0	-4.649403	2.684907	0.669260
3	6	0	-4.017889	2.123088	-0.437555
4	6	0	-3.410070	0.865602	-0.325995
5	6	0	-3.434283	0.174067	0.888648
6	6	0	-4.065325	0.755971	1.989593
7	1	0	-5.163401	2.450374	2.754737
8	1	0	-5.118779	3.659860	0.575817
9	1	0	-3.983949	2.640630	-1.390802
10	1	0	-2.948106	-0.788270	0.963684
11	1	0	-4.073009	0.219013	2.933922
12	8	0	-2.827377	0.386738	-1.472462
13	7	0	-2.050649	-0.800598	-1.273413
14	6	0	-2.549069	-1.863960	-1.940032
15	6	0	-3.909784	-1.724508	-2.612020
16	1	0	-4.674157	-1.417463	-1.892296
17	1	0	-3.884711	-0.963675	-3.397223
18	1	0	-4.179255	-2.689282	-3.043319
19	8	0	-1.926573	-2.943639	-1.976296
20	16	0	0.007291	-0.397447	-0.980844
21	6	0	2.892854	-0.999081	-0.615386
22	6	0	2.420693	1.228837	-0.337621
23	7	0	1.894125	-0.029844	-0.598619
24	8	0	2.735312	-2.198046	-0.798045
25	8	0	1.789971	2.272776	-0.225055
26	6	0	3.904729	1.062242	-0.193178
27	6	0	4.901606	1.989110	0.067804
28	6	0	6.217561	1.513287	0.158883
29	6	0	6.505991	0.153356	-0.006725
30	1	0	7.534207	-0.185380	0.070291
31	6	0	5.487984	-0.775021	-0.268581
32	1	0	5.704244	-1.830538	-0.395688
33	6	0	4.191704	-0.292668	-0.357662
34	1	0	7.026443	2.207877	0.361704
35	1	0	4.670169	3.041209	0.196806
36	6	0	-0.212564	-0.949479	0.723723
37	6	0	-0.672404	-2.244207	0.981190
38	6	0	0.047142	-0.075171	1.783004
39	6	0	-0.883401	-2.654523	2.298507
40	1	0	-0.897900	-2.896933	0.144320
41	6	0	-0.154712	-0.496267	3.097766

42	1	0	0.395866	0.930133	1.574778
43	6	0	-0.624643	-1.784786	3.360667
44	1	0	-1.247106	-3.659111	2.494907
45	1	0	0.042490	0.189534	3.916557
46	1	0	-0.788956	-2.107892	4.384133

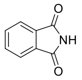

Center Number	Atomic Number	Atomic Type	Coordinates (in Å) X	Y	Z
1	6	0	-6.022714	-1.507646	0.251345
2	6	0	-6.560452	-0.337050	0.795031
3	6	0	-7.897532	-0.250417	1.150213
4	6	0	-8.693684	-1.384793	0.942989
5	6	0	-8.155177	-2.556977	0.398574
6	6	0	-6.802124	-2.634771	0.042706
7	6	0	-4.573774	-1.276414	-0.019520
8	6	0	-5.472093	0.679360	0.888681
9	1	0	-8.308438	0.660480	1.571439
10	1	0	-9.745217	-1.356263	1.208244
11	1	0	-8.797807	-3.418366	0.250537
12	1	0	-6.378765	-3.539472	-0.379522
13	8	0	-5.521437	1.821483	1.304228
14	8	0	-3.748898	-2.037772	-0.488028
15	7	0	-4.333054	0.041287	0.385187
16	1	0	-3.427326	0.486221	0.323174

INT1-S

Center Number	Atomic Number	Atomic Type	Coordinates (in Å) X	Y	Z
1	6	0	1.884852	-1.631350	-0.948751
2	6	0	2.244046	-1.002323	0.240383
3	6	0	3.555275	-1.035309	0.714236
4	6	0	4.524439	-1.714889	-0.021392
5	6	0	4.188296	-2.345836	-1.222106
6	6	0	2.870864	-2.298145	-1.678605
7	1	0	0.868085	-1.582575	-1.313656
8	1	0	5.546564	-1.741926	0.342507
9	1	0	4.947482	-2.865402	-1.797082
10	1	0	2.598314	-2.780327	-2.611959
11	8	0	1.344349	-0.324225	1.066016
12	7	0	0.126932	0.003375	0.441510

13	6	0	-0.989446	-0.740400	0.818896
14	6	0	-0.702853	-1.922331	1.714316
15	1	0	-0.247884	-1.595337	2.652263
16	1	0	-1.645168	-2.431610	1.911326
17	1	0	-0.003834	-2.611632	1.233277
18	8	0	-2.100685	-0.443300	0.408698
19	6	0	1.578378	1.064226	-4.091928
20	6	0	1.407589	1.446522	-2.760894
21	6	0	0.261678	1.042496	-2.069729
22	6	0	-0.715537	0.262728	-2.701525
23	6	0	-0.523236	-0.136217	-4.023009
24	6	0	0.620184	0.266222	-4.719171
25	1	0	2.467891	1.373901	-4.630988
26	1	0	2.163489	2.037464	-2.255151
27	1	0	-1.597917	-0.039178	-2.148254
28	1	0	-1.269751	-0.753159	-4.512960
29	1	0	0.762874	-0.042334	-5.749841
30	1	0	3.797873	-0.530625	1.642982
31	16	0	0.051500	1.524227	-0.356613

TS1-S

Center Number	Atomic Number	Atomic Type	Coordinates (in Å) X	Y	Z
1	6	0	-2.280140	-2.127846	0.341627
2	6	0	-1.615566	-1.756995	-0.853344
3	6	0	-2.345153	-1.079026	-1.865377
4	6	0	-3.689627	-0.813074	-1.693748
5	6	0	-4.336395	-1.184204	-0.499787
6	6	0	-3.637483	-1.846327	0.506670
7	1	0	-1.716693	-2.659240	1.099914
8	1	0	-4.246963	-0.297306	-2.469076
9	1	0	-5.388916	-0.955496	-0.366620
10	1	0	-4.143749	-2.134421	1.422276
11	8	0	-0.325548	-1.970949	-0.986290
12	7	0	0.541527	-0.497119	-0.138277
13	6	0	1.825338	-1.003119	0.151477
14	6	0	2.386767	-1.926903	-0.897579
15	1	0	2.139276	-1.579616	-1.901411
16	1	0	3.466075	-1.993925	-0.761606
17	1	0	1.936835	-2.916044	-0.778698
18	8	0	2.452211	-0.653200	1.141695
19	6	0	-3.440426	2.526417	0.213639
20	6	0	-2.626023	1.566635	0.816661
21	6	0	-1.357290	1.318898	0.286468

22	6	0	-0.893432	2.015894	-0.832719
23	6	0	-1.721744	2.960056	-1.432568
24	6	0	-2.994509	3.218160	-0.912068
25	1	0	-4.429652	2.717779	0.616540
26	1	0	-2.982309	1.002244	1.671511
27	1	0	0.089205	1.796791	-1.233768
28	1	0	-1.372105	3.500191	-2.306679
29	1	0	-3.634462	3.955639	-1.385441
30	1	0	-1.813509	-0.770395	-2.759136
31	16	0	-0.363725	0.083506	1.105199

INT2-S

Center Number	Atomic Number	Atomic Type	Coordinates (in Å) X	Y	Z
1	6	0	-1.590106	-1.454064	0.234832
2	6	0	-1.589403	-1.463632	-1.273506
3	6	0	-2.823577	-1.039437	-1.927975
4	6	0	-3.961053	-0.860405	-1.212703
5	6	0	-4.007177	-1.056955	0.224547
6	6	0	-2.891238	-1.367779	0.918259
7	1	0	-0.887718	-2.182788	0.641934
8	1	0	-4.878256	-0.575910	-1.719416
9	1	0	-4.962359	-0.966468	0.730238
10	1	0	-2.918402	-1.520062	1.992234
11	8	0	-0.565799	-1.766614	-1.890702
12	7	0	0.803158	0.204833	-0.168570
13	6	0	1.645572	-0.802924	0.203572
14	6	0	2.941215	-0.828655	-0.580726
15	1	0	3.756205	-1.156209	0.067991
16	1	0	2.840773	-1.557122	-1.392495
17	1	0	3.172083	0.143706	-1.017942
18	8	0	1.393052	-1.665786	1.064483
19	6	0	-3.434907	2.912924	0.019371
20	6	0	-2.622035	1.961925	0.638307
21	6	0	-1.533194	1.445033	-0.065782
22	6	0	-1.226962	1.869055	-1.360505
23	6	0	-2.040903	2.826053	-1.960426
24	6	0	-3.146042	3.341557	-1.276235
25	1	0	-4.286799	3.319455	0.553673
26	1	0	-2.834890	1.629676	1.648121
27	1	0	-0.368329	1.447381	-1.867425
28	1	0	-1.816425	3.165858	-2.966073
29	1	0	-3.778023	4.083928	-1.752689
30	1	0	-2.796769	-0.922610	-3.005887

31	16	0	-0.569464	0.175678	0.746901

TS2-S

Center Number	Atomic Number	Atomic Type	Coordinates (in Å) X	Y	Z
1	6	0	-1.450404	-2.027114	0.176053
2	6	0	-1.608165	-1.477727	-1.189566
3	6	0	-2.841377	-0.723949	-1.427072
4	6	0	-3.804285	-0.653071	-0.468063
5	6	0	-3.634635	-1.279193	0.811882
6	6	0	-2.510556	-2.045913	1.078873
7	1	0	-0.538259	-2.583501	0.365594
8	1	0	-4.718524	-0.098568	-0.655148
9	1	0	-4.434235	-1.209955	1.540391
10	1	0	-2.394701	-2.532032	2.040912
11	8	0	-0.746167	-1.651103	-2.062645
12	7	0	-2.042134	0.857750	1.606538
13	6	0	-2.678918	1.798321	0.799124
14	6	0	-3.973984	2.303718	1.396329
15	1	0	-3.763582	3.201306	1.988449
16	1	0	-4.661664	2.580053	0.595385
17	1	0	-4.430483	1.562431	2.052903
18	8	0	-2.238952	2.221319	-0.270017
19	6	0	2.024956	-1.619405	3.263915
20	6	0	1.398923	-0.964332	2.205834
21	6	0	0.094989	-0.483841	2.376902
22	6	0	-0.587024	-0.649267	3.584967
23	6	0	0.053633	-1.305335	4.634677
24	6	0	1.354173	-1.791466	4.477376
25	1	0	3.035696	-1.993528	3.140339
26	1	0	1.915726	-0.829006	1.260504
27	1	0	-1.593600	-0.263193	3.683531
28	1	0	-0.465917	-1.438245	5.578068
29	1	0	1.844960	-2.301975	5.299420
30	1	0	-2.967758	-0.267979	-2.402655
31	16	0	-0.642871	0.317088	0.971367

INT3-S

Center Number	Atomic Number	Atomic Type	Coordinates (in Å) X	Y	Z
1	6	0	-2.246974	-1.913681	0.453174
2	6	0	-1.790468	-2.497680	-0.823154

3	6	0	-2.248520	-1.833252	-2.060262
4	6	0	-3.055787	-0.765144	-2.029512
5	6	0	-3.598930	-0.195274	-0.750298
6	6	0	-3.058136	-0.848859	0.491350
7	1	0	-1.876367	-2.381417	1.359672
8	1	0	-3.375377	-0.270316	-2.942394
9	1	0	-4.680003	-0.378272	-0.761265
10	1	0	-3.371604	-0.410977	1.434299
11	8	0	-1.051642	-3.483648	-0.855596
12	7	0	-3.416945	1.270499	-0.702071
13	6	0	-4.450089	2.187187	-0.535367
14	6	0	-5.867484	1.644541	-0.549554
15	1	0	-6.541097	2.495978	-0.465320
16	1	0	-6.087333	1.103808	-1.474225
17	1	0	-6.045560	0.969701	0.293024
18	8	0	-4.228582	3.381765	-0.387387
19	6	0	0.292048	0.982799	2.592003
20	6	0	-0.149378	1.015962	1.268888
21	6	0	-1.267423	1.785600	0.929728
22	6	0	-1.949716	2.515557	1.912050
23	6	0	-1.519975	2.450385	3.236571
24	6	0	-0.397219	1.691067	3.577690
25	1	0	1.160205	0.386556	2.854330
26	1	0	0.357411	0.436205	0.504936
27	1	0	-2.805458	3.119269	1.633412
28	1	0	-2.054030	3.005103	4.001720
29	1	0	-0.062118	1.651346	4.609234
30	1	0	-1.884017	-2.247533	-2.994918
31	16	0	-1.780322	1.840829	-0.785903

5g

Center Number	Atomic Number	Atomic Type	Coordinates (in Å) X	Y	Z
1	6	0	-1.556044	-1.812050	0.300534
2	6	0	-1.936489	-1.928475	-1.041657
3	6	0	-3.294613	-1.956256	-1.386477
4	6	0	-4.261505	-1.872548	-0.391830
5	6	0	-3.885603	-1.765366	0.950585
6	6	0	-2.531242	-1.726827	1.291303
7	1	0	-5.315649	-1.892653	-0.647731
8	1	0	-2.245791	-1.630077	2.332943
9	8	0	-1.033317	-2.018656	-2.057558
10	7	0	-4.885167	-1.660451	1.974365
11	6	0	-5.272229	-2.737463	2.764984

12	6	0	-4.742570	-4.088372	2.325690
13	1	0	-5.247805	-4.856005	2.910790
14	1	0	-4.907173	-4.257364	1.259435
15	1	0	-3.665148	-4.150065	2.502615
16	8	0	-5.993935	-2.604749	3.744847
17	6	0	-2.422689	2.204375	3.790626
18	6	0	-3.451991	1.705016	2.989731
19	6	0	-4.136637	0.549779	3.380706
20	6	0	-3.785132	-0.114784	4.564348
21	6	0	-2.736555	0.373657	5.340798
22	6	0	-2.058744	1.536094	4.959811
23	1	0	-1.894086	3.102849	3.488275
24	1	0	-3.718783	2.202349	2.063159
25	1	0	-4.329856	-1.004444	4.859798
26	1	0	-2.456290	-0.144806	6.252395
27	1	0	-1.248730	1.917325	5.573390
28	1	0	-3.571993	-2.045299	-2.430983
29	16	0	-5.466498	-0.064280	2.351965
30	1	0	-0.135707	-1.993095	-1.696960
31	1	0	-0.503684	-1.786446	0.568072

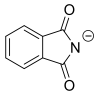

Center Number	Atomic Number	Atomic Type	Coordinates (in Å) X	Y	Z
1	6	0	-6.195602	-1.373689	0.128504
2	6	0	-6.733222	-0.513985	1.081706
3	6	0	-7.994120	-0.724032	1.617568
4	6	0	-8.716994	-1.839551	1.164517
5	6	0	-8.176515	-2.703997	0.205721
6	6	0	-6.897293	-2.478390	-0.327988
7	6	0	-4.823206	-0.819717	-0.204580
8	6	0	-5.682845	0.554649	1.317649
9	1	0	-8.408270	-0.050121	2.361894
10	1	0	-9.708694	-2.037247	1.560904
11	1	0	-8.756948	-3.559463	-0.127421
12	1	0	-6.472461	-3.145605	-1.072227
13	8	0	-5.829169	1.493024	2.112871
14	8	0	-4.056133	-1.342243	-1.024896
15	7	0	-4.586896	0.314570	0.528941

TS$_{SN2}$

Center Atomic Atomic Coordinates (in Å)

Number	Number	Type	X	Y	Z
1	6	0	-2.988190	0.033115	-0.817495
2	6	0	-2.860992	1.430602	-0.768946
3	6	0	-3.586875	2.202519	0.138226
4	6	0	-4.467196	1.585083	1.029132
5	6	0	-4.613380	0.194461	0.997104
6	6	0	-3.884718	-0.567315	0.088874
7	1	0	-2.164485	1.910736	-1.448272
8	1	0	-3.465165	3.283261	0.155063
9	1	0	-5.296308	-0.278389	1.696748
10	1	0	-3.999239	-1.642951	0.073422
11	8	0	-5.209447	2.286867	1.950838
12	7	0	-2.140403	-0.689549	-1.673476
13	6	0	-2.583110	-1.789162	-2.312814
14	6	0	-1.525176	-2.557085	-3.102030
15	1	0	-1.004373	-3.261961	-2.443715
16	1	0	-2.020596	-3.131013	-3.888202
17	1	0	-0.767815	-1.902530	-3.536090
18	8	0	-3.760451	-2.213755	-2.325294
19	16	0	0.043074	-0.325820	-1.277861
20	6	0	2.839979	-1.004176	-0.716590
21	6	0	2.382556	1.236984	-0.451125
22	7	0	1.860969	-0.012734	-0.779598
23	8	0	2.677042	-2.195841	-0.930730
24	8	0	1.761776	2.288844	-0.389232
25	6	0	3.841523	1.035567	-0.173562
26	6	0	4.827806	1.939356	0.189365
27	6	0	6.120960	1.436658	0.391804
28	6	0	6.398680	0.073643	0.232841
29	1	0	7.409359	-0.285932	0.397133
30	6	0	5.392282	-0.831244	-0.133643
31	1	0	5.599845	-1.888828	-0.257537
32	6	0	4.118202	-0.322675	-0.331677
33	1	0	6.920674	2.112621	0.676807
34	1	0	4.605065	2.994008	0.311713
35	6	0	-0.366249	-0.759000	0.419858
36	6	0	-0.653501	-2.083302	0.759129
37	6	0	-0.419839	0.238403	1.398029
38	6	0	-1.000124	-2.407373	2.070992
39	1	0	-0.613649	-2.851091	-0.005101
40	6	0	-0.756766	-0.090971	2.711664
41	1	0	-0.195933	1.262330	1.123242
42	6	0	-1.052485	-1.412651	3.050521
43	1	0	-1.225097	-3.438042	2.328890
44	1	0	-0.799725	0.688095	3.467098

45	1	0	-1.322926	-1.666786	4.070872
46	1	0	-5.002833	3.227486	1.863710

4a

Center Number	Atomic Number	Atomic Type	Coordinates (in Å) X	Y	Z
1	6	0	-5.789239	-2.647427	-1.894568
2	6	0	-6.013017	-1.355174	-2.375149
3	6	0	-5.959273	-0.274411	-1.488596
4	6	0	-5.686800	-0.493564	-0.144311
5	6	0	-5.462018	-1.789635	0.345569
6	6	0	-5.515291	-2.870154	-0.545270
7	1	0	-5.648200	0.352591	0.536208
8	1	0	-5.342847	-3.870258	-0.177125
9	8	0	-6.285903	-1.085126	-3.688270
10	7	0	-5.190686	-1.923762	1.725252
11	6	0	-4.959873	-3.060583	2.449584
12	6	0	-4.703983	-2.830516	3.927749
13	1	0	-3.725643	-3.247142	4.181655
14	1	0	-4.730589	-1.780364	4.225357
15	1	0	-5.454259	-3.380894	4.501810
16	8	0	-4.954100	-4.193881	1.968144
17	1	0	-6.132788	0.728890	-1.862233
18	1	0	-6.300185	-1.914017	-4.186753
19	1	0	-5.827149	-3.492293	-2.577143
20	1	0	-5.169379	-1.055221	2.240100

TS2′-S

Center Number	Atomic Number	Atomic Type	Coordinates (in Å) X	Y	Z
1	6	0	-2.057378	-1.133370	0.425558
2	6	0	-2.626483	-1.316328	-0.944156
3	6	0	-3.913489	-2.002885	-0.973357
4	6	0	-4.660421	-2.181688	0.151531
5	6	0	-4.208812	-1.736979	1.441470
6	6	0	-2.977440	-1.178141	1.561620
7	1	0	-5.643470	-2.638339	0.073659
8	1	0	-4.858454	-1.838509	2.303568
9	1	0	-2.623403	-0.821561	2.523481
10	8	0	-2.041423	-0.966529	-1.977687
11	7	0	0.636423	-0.332992	-0.052945
12	6	0	1.445393	-0.888933	0.888333

13	6	0	2.782580	-1.341959	0.324576
14	1	0	2.794838	-2.436348	0.293971
15	1	0	2.959015	-0.957995	-0.680742
16	1	0	3.584954	-1.021155	0.994187
17	8	0	1.179564	-1.082691	2.090905
18	6	0	-3.066032	2.975668	-1.181890
19	6	0	-2.647960	1.921539	-0.372374
20	6	0	-1.340030	1.448835	-0.498876
21	6	0	-0.441795	2.019166	-1.396191
22	6	0	-0.872938	3.078254	-2.197197
23	6	0	-2.180194	3.555483	-2.094058
24	1	0	-4.083449	3.343501	-1.098674
25	1	0	-3.335236	1.466306	0.333872
26	1	0	0.562426	1.620588	-1.465721
27	1	0	-0.183964	3.525920	-2.906327
28	1	0	-2.509452	4.377824	-2.720894
29	1	0	-4.293055	-2.270423	-1.954497
30	16	0	-0.801793	0.170913	0.644117
31	1	0	-1.296304	-2.249187	0.543740
32	6	0	0.208930	-3.866399	-0.393333
33	6	0	1.118223	-4.915524	-0.244328
34	6	0	1.337627	-5.458289	1.017448
35	6	0	0.646139	-4.940705	2.107749
36	6	0	-0.251149	-3.890569	1.914181
37	1	0	1.643526	-5.290275	-1.114788
38	1	0	2.042595	-6.272423	1.150006
39	1	0	0.797081	-5.334036	3.106025
40	7	0	-0.456182	-3.385684	0.679027
41	6	0	-0.044365	-3.238080	-1.730282
42	1	0	0.644347	-3.643797	-2.473165
43	1	0	-1.067220	-3.429855	-2.065077
44	1	0	0.077287	-2.154411	-1.666614
45	6	0	-0.993015	-3.273295	3.061443
46	1	0	-2.071446	-3.286476	2.887686
47	1	0	-0.778733	-3.809538	3.987053
48	1	0	-0.671709	-2.233798	3.172766

5g′

Center Number	Atomic Number	Atomic Type	Coordinates (in Å) X	Y	Z
1	6	0	-2.143801	-0.917426	0.286080
2	6	0	-2.180050	-1.474229	-1.011729
3	6	0	-3.226122	-2.357632	-1.312896
4	6	0	-4.183364	-2.672737	-0.353864

5	6	0	-4.122875	-2.132590	0.935947
6	6	0	-3.092550	-1.254524	1.255805
7	1	0	-4.983131	-3.359379	-0.612388
8	1	0	-4.866660	-2.393982	1.679776
9	1	0	-3.020875	-0.818909	2.247209
10	8	0	-1.274290	-1.185958	-1.964414
11	7	0	0.468371	-0.201821	-0.197955
12	6	0	1.271947	-1.030625	0.537236
13	6	0	2.515073	-1.469596	-0.216018
14	1	0	2.498264	-2.558057	-0.325083
15	1	0	2.588471	-1.007496	-1.200757
16	1	0	3.397174	-1.211674	0.376818
17	8	0	1.046525	-1.422268	1.690942
18	6	0	-3.141575	3.165342	-1.033392
19	6	0	-2.756971	2.017653	-0.340988
20	6	0	-1.401147	1.695110	-0.274981
21	6	0	-0.426533	2.498303	-0.865830
22	6	0	-0.828424	3.643501	-1.554610
23	6	0	-2.180931	3.977289	-1.639566
24	1	0	-4.194014	3.419793	-1.100673
25	1	0	-3.501878	1.380255	0.121178
26	1	0	0.616837	2.215523	-0.805185
27	1	0	-0.080247	4.270609	-2.028036
28	1	0	-2.486849	4.867722	-2.178779
29	1	0	-3.261629	-2.788055	-2.307789
30	16	0	-0.881351	0.270423	0.710951
31	1	0	-0.463060	-0.792058	-1.530064

Supplementary Table 17. Calculated electronic energies of Se-catalyzed reaction in 1,4-dioxane at the B3LYP-D3/BS1 level (E_1, in a.u.), calculated Gibbs free energies in 1,4-dioxane at the B3LYP-D3/BS1 level (G_1, in a.u.), calculated electronic energies in 1,4-dioxane at the B3LYP-D3/BS2 level (E_2, in a.u.), corrected free energies ($G_{correct}$, in a.u.) for all stationary points.

**Species**	G_1	E_1	E_2	$G_{correct}$
**1a**	-515.34993	-515.47085	-515.63064	-515.50972
**PhSeBr**	-254.05004	-254.10553	-1021.93283	-1021.87734
**INT1-Se**	-755.59348	-755.78502	-1120.01629	-1119.82475

**TS1-Se**	-755.57126	-755.76225	-1119.99551	-1119.80452
**INT2-Se**	-755.58838	-755.77911	-1120.01525	-1119.82452
**TS2-Se**	-755.58007	-755.77008	-1120.00758	-1119.81757
**INT3-Se**	-755.61684	-755.80885	-1120.04335	-1119.85134
**INT3-Se′**	-769.41950	-769.62041	-1537.59565	-1537.39474
**TS-Se-re**	-1284.77452	-1285.11494	-2053.24152	-2052.90109
**$TS_{SeN2'}$**	-769.38565	-769.587744	-1537.56551	-1537.36341
**2a′**	-755.65371	-755.84414	-1120.07932	-1119.88889
**2a**	-515.41384	-515.53645	-515.69972	-515.57711
**TS2′-Se**	-1270.93027	-1271.25712	-1635.64272	-1635.31588
**INT2′-Se**	-755.61832	-755.80894	-1120.04527	-1119.85464
**TS_{SeN2}**	-769.42780	-769.62790	-1537.60681	-1537.40671
**HBr**	-13.79125	-13.77791	-417.52884	-417.542187
**TS2′**	-515.29415	-515.41446	-515.57482	-515.45451
**INT5**	-515.34226	-515.46494	-515.62582	-515.50314
**TS3′**	-515.30116	-515.42183	-515.58378	-515.46311

1a

Center Number	Atomic Number	Atomic Type	Coordinates (in Å) X	Y	Z
1	6	0	1.807854	-1.239560	0.098933
2	6	0	0.716361	-0.434634	-0.231909

3	6	0	0.853927	0.943134	-0.376315
4	6	0	2.113232	1.516404	-0.186490
5	6	0	3.216228	0.730030	0.142968
6	6	0	3.055536	-0.650698	0.284988
7	1	0	1.662192	-2.308707	0.208110
8	1	0	-0.003695	1.551445	-0.631284
9	1	0	2.224650	2.590823	-0.296529
10	1	0	4.189679	1.185933	0.290342
11	1	0	3.904889	-1.275468	0.543416
12	8	0	-0.476640	-1.125269	-0.400374
13	7	0	-1.563642	-0.312838	-0.732766
14	1	0	-1.959274	-0.612938	-1.616064
15	6	0	-2.418698	0.019857	0.308272
16	6	0	-3.755011	0.554041	-0.170256
17	1	0	-3.799363	0.709562	-1.250913
18	1	0	-4.538506	-0.151729	0.120552
19	1	0	-3.953407	1.500295	0.336871
20	8	0	-2.092209	-0.054964	1.477006

PhSeBr

Center Number	Atomic Number	Atomic Type	Coordinates (in Å) X	Y	Z
1	6	0	3.255095	-0.543478	1.219361
2	6	0	2.028324	0.117204	1.209572
3	6	0	1.406301	0.403446	-0.014586
4	6	0	2.010622	0.028003	-1.223409
5	6	0	3.237394	-0.632347	-1.202592
6	6	0	3.857681	-0.917586	0.016035
7	1	0	3.739279	-0.767230	2.164403
8	1	0	1.547831	0.406347	2.137502
9	1	0	1.516577	0.248781	-2.162934
10	1	0	3.707915	-0.925075	-2.135612
11	1	0	4.812841	-1.433041	0.027954
12	34	0	-0.255900	1.358022	-0.037267
13	35	0	-1.785177	-0.508258	0.040097

INT1-Se

Center Number	Atomic Number	Atomic Type	Coordinates (in Å) X	Y	Z
1	6	0	-2.132336	2.628355	-1.616772
2	6	0	-1.548101	1.830550	-0.632783
3	6	0	-2.322799	1.151970	0.304696

4	6	0	-3.712074	1.274416	0.241565
5	6	0	-4.316088	2.069110	-0.732140
6	6	0	-3.519479	2.747515	-1.658190
7	1	0	-1.495941	3.138322	-2.331679
8	1	0	-4.319650	0.736299	0.962218
9	1	0	-5.396720	2.156661	-0.773686
10	1	0	-3.977916	3.366459	-2.423025
11	8	0	-0.154763	1.801470	-0.657799
12	7	0	0.411429	0.774813	0.114594
13	6	0	0.992967	1.143093	1.321580
14	6	0	0.813908	2.595372	1.707536
15	1	0	-0.247835	2.846064	1.782463
16	1	0	1.298651	2.747538	2.670906
17	1	0	1.252829	3.254137	0.954473
18	8	0	1.575447	0.321686	2.011225
19	6	0	-1.828168	-2.730943	2.152777
20	6	0	-0.752902	-2.100313	1.527503
21	6	0	-0.818521	-1.833784	0.154030
22	6	0	-1.937006	-2.214626	-0.594049
23	6	0	-2.996326	-2.867296	0.036825
24	6	0	-2.945885	-3.117831	1.409094
25	1	0	-1.789146	-2.927961	3.219603
26	1	0	0.120356	-1.789741	2.089783
27	1	0	-1.986759	-1.980203	-1.651750
28	1	0	-3.867008	-3.162441	-0.540262
29	1	0	-3.777558	-3.613808	1.899831
30	34	0	0.640346	-0.900750	-0.702517
31	1	0	-1.858477	0.520364	1.049335

TS1-Se

Center Number	Atomic Number	Atomic Type	Coordinates (in Å) X	Y	Z
1	6	0	-2.284813	-2.074666	0.341932
2	6	0	-1.610568	-1.654275	-0.832268
3	6	0	-2.332374	-0.929042	-1.817499
4	6	0	-3.676661	-0.668102	-1.644549
5	6	0	-4.333648	-1.093189	-0.474238
6	6	0	-3.644331	-1.800327	0.506718
7	1	0	-1.730267	-2.650021	1.074106
8	1	0	-4.226189	-0.113736	-2.398347
9	1	0	-5.387967	-0.871862	-0.340862
10	1	0	-4.159698	-2.132695	1.402384
11	8	0	-0.324685	-1.872597	-0.969928
12	7	0	0.572840	-0.431242	-0.090485

13	6	0	1.806442	-1.042735	0.214085
14	6	0	2.402805	-1.836344	-0.922445
15	1	0	2.193034	-1.369546	-1.885338
16	1	0	3.475939	-1.931650	-0.755427
17	1	0	1.943747	-2.828476	-0.936241
18	8	0	2.373241	-0.892288	1.287700
19	6	0	-3.604686	2.535828	0.197582
20	6	0	-2.769696	1.638109	0.866071
21	6	0	-1.479881	1.412820	0.380865
22	6	0	-1.014519	2.074200	-0.758909
23	6	0	-1.862096	2.954001	-1.425567
24	6	0	-3.156591	3.187477	-0.949820
25	1	0	-4.610522	2.708731	0.567206
26	1	0	-3.132209	1.099019	1.735229
27	1	0	-0.015568	1.870224	-1.126462
28	1	0	-1.510652	3.463975	-2.317248
29	1	0	-3.811220	3.876373	-1.474144
30	34	0	-0.373667	0.155005	1.329619
31	1	0	-1.790716	-0.581978	-2.690436

INT2-Se

Center Number	Atomic Number	Atomic Type	Coordinates (in Å) X	Y	Z
1	6	0	-1.583766	-1.520976	0.279049
2	6	0	-1.544522	-1.426014	-1.222714
3	6	0	-2.766666	-0.950196	-1.871273
4	6	0	-3.915918	-0.797241	-1.169448
5	6	0	-3.991521	-1.078111	0.250749
6	6	0	-2.891926	-1.458102	0.940679
7	1	0	-0.869296	-2.247850	0.665964
8	1	0	-4.818779	-0.468924	-1.675968
9	1	0	-4.953769	-1.003956	0.746450
10	1	0	-2.945206	-1.686502	2.000932
11	8	0	-0.513975	-1.689819	-1.842250
12	7	0	0.915911	0.268566	-0.155515
13	6	0	1.701540	-0.794577	0.177268
14	6	0	2.950408	-0.931154	-0.666727
15	1	0	3.756876	-1.351298	-0.062831
16	1	0	2.730357	-1.628992	-1.481310
17	1	0	3.254353	0.021815	-1.101676
18	8	0	1.420592	-1.632228	1.058743
19	6	0	-3.543148	2.908373	-0.059523
20	6	0	-2.718291	2.011058	0.621372
21	6	0	-1.569115	1.532001	-0.010680

22	6	0	-1.219539	1.942202	-1.299243
23	6	0	-2.046181	2.845594	-1.962510
24	6	0	-3.207975	3.322341	-1.348420
25	1	0	-4.441058	3.284142	0.420173
26	1	0	-2.974818	1.686835	1.624595
27	1	0	-0.322469	1.541570	-1.755872
28	1	0	-1.787404	3.172634	-2.964529
29	1	0	-3.849325	4.022379	-1.874575
30	34	0	-0.518911	0.215142	0.923678
31	1	0	-2.714232	-0.769067	-2.939181

TS2-Se

Center Number	Atomic Number	Atomic Type	Coordinates (in Å) X	Y	Z
1	6	0	-1.800040	-1.183672	0.512049
2	6	0	-1.506703	-1.005471	-0.922615
3	6	0	-2.667010	-0.669094	-1.763500
4	6	0	-3.933950	-0.689154	-1.255076
5	6	0	-4.159308	-0.905181	0.134217
6	6	0	-3.088015	-1.230807	0.986534
7	1	0	-0.949450	-1.369544	1.159147
8	1	0	-4.780664	-0.476262	-1.898898
9	1	0	-5.174242	-0.930501	0.511903
10	1	0	-3.276293	-1.403708	2.040404
11	8	0	-0.368611	-1.147850	-1.383553
12	7	0	-3.778779	1.572640	0.696999
13	6	0	-4.592384	2.022772	-0.337882
14	6	0	-6.074323	1.893990	-0.038389
15	1	0	-6.516694	2.894473	-0.028683
16	1	0	-6.558163	1.333553	-0.843305
17	1	0	-6.261070	1.408628	0.919417
18	8	0	-4.191183	2.501455	-1.398560
19	6	0	0.595483	1.315993	3.480820
20	6	0	0.038484	1.481041	2.213785
21	6	0	-1.353394	1.521806	2.076316
22	6	0	-2.190065	1.405988	3.188619
23	6	0	-1.619343	1.242279	4.450235
24	6	0	-0.231346	1.195100	4.599649
25	1	0	1.674418	1.279414	3.591765
26	1	0	0.681845	1.562153	1.342026
27	1	0	-3.264140	1.439970	3.051055
28	1	0	-2.262646	1.152455	5.320168
29	1	0	0.204886	1.066190	5.584961
30	34	0	-2.047384	1.729514	0.294240

31	1	0	-2.475426	-0.484021	-2.814883

INT3-Se

Center Number	Atomic Number	Atomic Type	Coordinates (in Å) X	Y	Z
1	6	0	-2.178892	-1.979941	0.286442
2	6	0	-1.789101	-2.555087	-1.018198
3	6	0	-2.310217	-1.873925	-2.223010
4	6	0	-3.112530	-0.805199	-2.138421
5	6	0	-3.593424	-0.248484	-0.827747
6	6	0	-2.986594	-0.915952	0.376213
7	1	0	-1.757376	-2.454929	1.166667
8	1	0	-3.475644	-0.298280	-3.028760
9	1	0	-4.673735	-0.439426	-0.788873
10	1	0	-3.244265	-0.483492	1.338831
11	8	0	-1.056981	-3.539239	-1.097940
12	7	0	-3.418884	1.214136	-0.772060
13	6	0	-4.431959	2.111931	-0.465275
14	6	0	-5.850859	1.564113	-0.391963
15	1	0	-6.513510	2.412515	-0.228321
16	1	0	-6.142844	1.052893	-1.313784
17	1	0	-5.966885	0.861776	0.439609
18	8	0	-4.209275	3.297299	-0.267104
19	6	0	0.081764	0.912145	2.817073
20	6	0	-0.264314	0.908175	1.465399
21	6	0	-1.187348	1.843798	0.983464
22	6	0	-1.764877	2.781957	1.849303
23	6	0	-1.429363	2.762459	3.202764
24	6	0	-0.504741	1.833445	3.686172
25	1	0	0.798999	0.188208	3.191170
26	1	0	0.163456	0.174672	0.791062
27	1	0	-2.476676	3.498756	1.457704
28	1	0	-1.882514	3.481335	3.878471
29	1	0	-0.241228	1.828477	4.739333
30	34	0	-1.640791	1.851209	-0.894891
31	1	0	-1.990281	-2.277240	-3.178478

2a′

Center Number	Atomic Number	Atomic Type	Coordinates (in Å) X	Y	Z
1	6	0	-1.624542	-1.669554	0.338302
2	6	0	-1.958366	-1.796627	-1.014407

3	6	0	-3.301611	-1.901919	-1.397755
4	6	0	-4.298476	-1.884078	-0.430187
5	6	0	-3.971928	-1.763525	0.924648
6	6	0	-2.630430	-1.647087	1.301262
7	1	0	-5.342403	-1.962623	-0.715359
8	1	0	-2.380868	-1.534115	2.350523
9	8	0	-1.021765	-1.823871	-2.005295
10	7	0	-5.002597	-1.724776	1.914952
11	6	0	-5.240369	-2.781770	2.788195
12	6	0	-4.602871	-4.104536	2.395981
13	1	0	-5.062075	-4.888403	2.997474
14	1	0	-4.728279	-4.317614	1.332341
15	1	0	-3.528208	-4.080272	2.598395
16	8	0	-5.924130	-2.667430	3.794042
17	6	0	-2.214138	1.926142	3.625069
18	6	0	-3.322117	1.530741	2.873205
19	6	0	-4.211522	0.584618	3.394278
20	6	0	-3.989757	0.024900	4.660324
21	6	0	-2.868955	0.409922	5.394370
22	6	0	-1.984827	1.363032	4.880751
23	1	0	-1.524866	2.662161	3.222786
24	1	0	-3.490315	1.940168	1.883014
25	1	0	-4.682991	-0.715800	5.042306
26	1	0	-2.690130	-0.026843	6.372118
27	1	0	-1.117593	1.665052	5.459897
28	1	0	-3.541260	-1.997964	-2.450838
29	1	0	-0.140766	-1.739325	-1.616205
30	1	0	-0.583109	-1.582479	0.636916
31	34	0	-5.710391	-0.015213	2.336866

2a

Center Number	Atomic Number	Atomic Type	Coordinates (in Å) X	Y	Z
1	6	0	-2.028534	-1.270261	0.193802
2	6	0	-2.898960	-0.304998	-0.320714
3	6	0	-2.429190	0.989361	-0.570333
4	6	0	-1.108283	1.313374	-0.286157
5	6	0	-0.228065	0.352396	0.229090
6	6	0	-0.697454	-0.946092	0.447912
7	1	0	-2.382868	-2.281519	0.376231
8	1	0	-0.749343	2.323281	-0.458734
9	1	0	-0.018017	-1.710171	0.807539
10	8	0	-4.209228	-0.563757	-0.603709
11	7	0	1.123842	0.705619	0.475726

12	6	0	1.870957	0.553468	1.621134
13	6	0	1.203239	-0.087981	2.821521
14	1	0	1.721091	0.265704	3.713183
15	1	0	0.138107	0.141867	2.885678
16	1	0	1.312176	-1.176163	2.776738
17	8	0	3.036673	0.934195	1.650693
18	1	0	-3.112733	1.728868	-0.972413
19	1	0	1.610774	1.226375	-0.243390
20	1	0	-4.404999	-1.486955	-0.394600

TS2′-Se

Center Number	Atomic Number	Atomic Type	Coordinates (in Å) X	Y	Z
1	6	0	-1.305058	1.820169	-0.468870
2	6	0	-1.738763	2.265468	0.866774
3	6	0	-2.885420	3.136166	0.920519
4	6	0	-3.550744	3.505053	-0.216177
5	6	0	-3.147503	3.057295	-1.512731
6	6	0	-2.090703	2.203449	-1.626454
7	1	0	-4.418423	4.154550	-0.136373
8	1	0	-3.689451	3.390525	-2.390969
9	1	0	-1.788158	1.828137	-2.601152
10	8	0	-1.099158	1.944022	1.907594
11	7	0	0.389957	-0.672142	0.581065
12	6	0	1.617351	-0.474100	0.050723
13	6	0	2.744457	-1.103573	0.852409
14	1	0	2.373163	-1.680800	1.699345
15	1	0	3.327578	-1.749743	0.190034
16	1	0	3.402427	-0.312272	1.219329
17	8	0	1.868769	0.176676	-0.988982
18	6	0	-4.754376	-1.205686	0.358570
19	6	0	-3.601338	-0.832804	-0.331503
20	6	0	-2.396608	-0.724304	0.366322
21	6	0	-2.319328	-0.980176	1.735224
22	6	0	-3.480271	-1.354483	2.411764
23	6	0	-4.692935	-1.466087	1.728784
24	1	0	-5.695573	-1.295389	-0.174296
25	1	0	-3.644337	-0.623725	-1.396217
26	1	0	-1.368558	-0.878690	2.241396
27	1	0	-3.436110	-1.554537	3.477715
28	1	0	-5.591087	-1.757827	2.264157
29	34	0	-0.852052	-0.118726	-0.625590
30	1	0	-3.208801	3.463764	1.903022
31	1	0	0.456487	1.938333	1.599269

32	6	0	3.568657	2.250192	4.256996
33	6	0	2.416717	2.436696	3.494052
34	6	0	1.331215	3.167065	3.970850
35	6	0	1.420606	3.728846	5.246709
36	6	0	2.563663	3.559801	6.027240
37	6	0	3.634976	2.814670	5.528532
38	1	0	4.389639	1.671744	3.847490
39	1	0	0.579558	4.298225	5.629727
40	1	0	2.619625	4.001456	7.016631
41	1	0	4.529824	2.675636	6.126860
42	8	0	2.437494	1.803771	2.249907
43	7	0	1.448686	2.245871	1.378222
44	6	0	1.840436	2.776354	0.228375
45	6	0	3.290832	3.025773	-0.030798
46	1	0	3.830122	3.307569	0.872748
47	1	0	3.704566	2.088596	-0.417257
48	1	0	3.381918	3.793431	-0.798523
49	8	0	0.989346	3.067457	-0.671376
50	1	0	0.430308	3.275756	3.379767
51	1	0	-0.089152	2.471294	-0.590498

INT2′-Se

Center Number	Atomic Number	Atomic Type	Coordinates (in Å) X	Y	Z
1	6	0	-2.054796	1.276722	-0.263114
2	6	0	-2.492760	1.460510	1.066587
3	6	0	-3.306864	2.571239	1.335897
4	6	0	-3.649120	3.461359	0.324584
5	6	0	-3.186599	3.283027	-0.983975
6	6	0	-2.381307	2.185947	-1.273285
7	1	0	-4.277333	4.314338	0.561890
8	1	0	-3.446938	3.989717	-1.763946
9	1	0	-2.007677	2.027675	-2.280625
10	8	0	-2.192966	0.625196	2.076006
11	7	0	-0.110744	-0.596792	0.855747
12	6	0	1.030080	0.146286	0.803047
13	6	0	1.919678	0.000263	2.024672
14	1	0	2.912876	-0.321779	1.699654
15	1	0	2.029795	0.979021	2.500296
16	1	0	1.520131	-0.713797	2.745232
17	8	0	1.344794	0.899579	-0.135242
18	6	0	-4.649101	-2.369281	-0.774563
19	6	0	-3.670803	-1.389923	-0.941721
20	6	0	-2.403295	-1.598748	-0.396009

21	6	0	-2.091930	-2.767064	0.298416
22	6	0	-3.081314	-3.738779	0.457790
23	6	0	-4.355042	-3.542431	-0.076836
24	1	0	-5.641319	-2.211292	-1.184723
25	1	0	-3.902282	-0.468559	-1.466224
26	1	0	-1.105428	-2.893260	0.727865
27	1	0	-2.854429	-4.646926	1.007087
28	1	0	-5.120334	-4.300892	0.053184
29	34	0	-1.015395	-0.274823	-0.721321
30	1	0	-3.648544	2.714496	2.355259
31	1	0	-1.414710	0.051521	1.824242

TS-Se-re

Center Number	Atomic Number	Atomic Type	Coordinates (in Å) X	Y	Z
1	6	0	-3.111695	0.415920	0.801455
2	6	0	-2.378166	-0.801854	0.709087
3	6	0	-2.186210	-1.425262	-0.557404
4	6	0	-2.695832	-0.851938	-1.686918
5	6	0	-3.562971	0.302151	-1.612324
6	6	0	-3.627182	0.976427	-0.331038
7	1	0	-3.197708	0.897072	1.769163
8	1	0	-2.533739	-1.301706	-2.660467
9	1	0	-4.651748	-0.438539	-1.546086
10	1	0	-4.160872	1.917429	-0.275866
11	8	0	-1.845992	-1.383204	1.752700
12	7	0	-3.623957	1.136120	-2.801156
13	6	0	-4.784949	1.811535	-3.093101
14	6	0	-4.789061	2.758180	-4.269958
15	1	0	-5.823145	3.038398	-4.466393
16	1	0	-4.206346	3.655208	-4.043247
17	1	0	-4.351658	2.302279	-5.161087
18	8	0	-5.814148	1.631959	-2.433136
19	6	0	0.032899	1.917293	0.244243
20	6	0	-0.341743	1.566162	-1.051854
21	6	0	-1.272869	2.352885	-1.739994
22	6	0	-1.807899	3.503986	-1.144317
23	6	0	-1.435549	3.840959	0.155407
24	6	0	-0.524004	3.043655	0.852233
25	1	0	0.737589	1.298305	0.789249
26	1	0	0.051770	0.668312	-1.513921
27	1	0	-2.521602	4.114378	-1.687092
28	1	0	-1.860524	4.720611	0.627557
29	1	0	-0.252134	3.296176	1.871445
30	34	0	-1.915353	1.748192	-3.451681

31	1	0	-1.602160	-2.337122	-0.595637
32	1	0	-2.016662	-0.864286	2.614651
33	35	0	-2.413858	0.277409	4.319254
34	1	0	-6.511598	0.896154	-1.003218
35	6	0	-8.724007	1.470417	2.406248
36	6	0	-7.655871	0.894084	1.722689
37	6	0	-6.414650	0.711194	2.322520
38	6	0	-6.240267	1.121828	3.647142
39	6	0	-7.296094	1.700263	4.350714
40	6	0	-8.535126	1.871234	3.727536
41	1	0	-9.675605	1.597147	1.901755
42	1	0	-5.269940	0.976970	4.113406
43	1	0	-7.154585	2.015694	5.378972
44	1	0	-9.361214	2.322161	4.268392
45	8	0	-7.956769	0.486830	0.410983
46	7	0	-6.842084	0.172580	-0.348853
47	6	0	-6.523643	-1.107783	-0.549844
48	6	0	-7.264930	-2.170998	0.199969
49	1	0	-7.077516	-2.062137	1.272638
50	1	0	-8.341047	-2.069169	0.043277
51	1	0	-6.916213	-3.145858	-0.135938
52	8	0	-5.581566	-1.396031	-1.339076
53	1	0	-5.591967	0.266750	1.779441

INT3-Se′

Center Number	Atomic Number	Atomic Type	Coordinates (in Å) X	Y	Z
1	6	0	-1.382932	-0.730381	-1.864625
2	6	0	-0.558661	-0.112319	-2.870074
3	6	0	-1.022598	1.050292	-3.593703
4	6	0	-2.208968	1.611438	-3.289104
5	6	0	-3.132446	1.008921	-2.290847
6	6	0	-2.574216	-0.174481	-1.563023
7	1	0	-0.991667	-1.595735	-1.342937
8	1	0	-2.549594	2.509708	-3.793078
9	1	0	-4.002270	0.608392	-2.847477
10	1	0	-3.210468	-0.604120	-0.797781
11	8	0	0.613558	-0.543259	-3.163781
12	7	0	-3.705853	2.016189	-1.383875
13	6	0	-4.996900	1.771198	-0.931739
14	6	0	-5.589307	2.731849	0.073575
15	1	0	-6.616375	2.422189	0.261579
16	1	0	-5.022687	2.719272	1.008166
17	1	0	-5.577198	3.759631	-0.298270

18	8	0	-5.619168	0.810399	-1.371990
19	6	0	0.844885	0.960097	0.072139
20	6	0	-0.008952	1.966042	-0.377595
21	6	0	-1.299547	2.063489	0.153156
22	6	0	-1.724102	1.184649	1.157623
23	6	0	-0.865976	0.175421	1.593819
24	6	0	0.410877	0.053827	1.043063
25	1	0	1.834291	0.854457	-0.359132
26	1	0	0.304191	2.639337	-1.168247
27	1	0	-2.722662	1.273786	1.572646
28	1	0	-1.198438	-0.521982	2.355826
29	1	0	1.064737	-0.753850	1.350222
30	34	0	-2.537663	3.311406	-0.640591
31	1	0	-0.359357	1.465858	-4.343348
32	1	0	0.950526	-1.337741	-2.547288
33	35	0	1.602857	-2.714058	-1.325825

TS$_{SeN2'}$

Center Number	Atomic Number	Atomic Type	Coordinates (in Å) X	Y	Z
1	6	0	-1.573667	-0.963310	1.445381
2	6	0	-1.636218	-0.172043	0.197405
3	6	0	-2.896502	-0.252113	-0.576133
4	6	0	-3.932132	-0.998373	-0.174814
5	6	0	-3.861836	-1.877121	1.040598
6	6	0	-2.598535	-1.720632	1.849144
7	1	0	-4.858304	-1.034195	-0.740535
8	1	0	-2.553565	-2.265799	2.787679
9	8	0	-0.700987	0.531576	-0.169605
10	7	0	-5.051074	-1.652562	1.903976
11	6	0	-5.575361	-2.611413	2.636569
12	6	0	-5.163570	-4.048698	2.527425
13	1	0	-5.747059	-4.634455	3.235189
14	1	0	-5.345024	-4.419508	1.514631
15	1	0	-4.100612	-4.169838	2.748903
16	8	0	-6.511343	-2.394334	3.520315
17	6	0	-2.211852	2.169185	2.794629
18	6	0	-3.423467	1.727937	2.265618
19	6	0	-4.226737	0.865971	3.018147
20	6	0	-3.854589	0.475057	4.309584
21	6	0	-2.636911	0.913223	4.824749
22	6	0	-1.814277	1.750325	4.065721
23	1	0	-1.574056	2.821712	2.208234
24	1	0	-3.728895	2.021313	1.268080

25	1	0	-4.518431	-0.145317	4.900520
26	1	0	-2.335867	0.612462	5.822883
27	1	0	-0.865926	2.087135	4.472056
28	1	0	-2.944833	0.349906	-1.477497
29	1	0	-0.666959	-0.867434	2.032362
30	34	0	-5.817608	0.159728	2.216905
31	1	0	-6.733852	-1.425854	3.626731
32	35	0	-7.753853	1.006377	4.680678
33	1	0	-3.914579	-2.916233	0.685267

TS_{SeN2}

Center Number	Atomic Number	Atomic Type	Coordinates (in Å) X	Y	Z
1	6	0	-1.531344	-1.918286	0.572556
2	6	0	-1.738928	-1.800665	-0.806565
3	6	0	-3.036005	-1.616487	-1.309475
4	6	0	-4.113476	-1.553642	-0.438351
5	6	0	-3.904579	-1.686291	0.938205
6	6	0	-2.613711	-1.851045	1.444504
7	1	0	-5.118626	-1.401139	-0.816161
8	1	0	-2.457921	-1.911382	2.515770
9	8	0	-0.726986	-1.851009	-1.710142
10	7	0	-5.006341	-1.559476	1.838501
11	6	0	-5.462823	-2.560677	2.559440
12	6	0	-4.997457	-3.968974	2.383740
13	1	0	-5.835738	-4.640326	2.574451
14	1	0	-4.587805	-4.130419	1.388526
15	1	0	-4.218588	-4.180823	3.123239
16	8	0	-6.362160	-2.401100	3.492383
17	6	0	-1.928599	1.618226	3.104774
18	6	0	-3.110013	1.274677	2.447973
19	6	0	-4.197351	0.803556	3.194585
20	6	0	-4.125093	0.699505	4.588863
21	6	0	-2.941596	1.055178	5.233090
22	6	0	-1.844825	1.506338	4.493583
23	1	0	-1.080389	1.979989	2.532553
24	1	0	-3.187482	1.361634	1.370054
25	1	0	-4.999065	0.384241	5.148819
26	1	0	-2.879308	0.986906	6.314337
27	1	0	-0.927095	1.783024	5.003227
28	1	0	-3.175494	-1.522836	-2.380164
29	1	0	0.116980	-1.965583	-1.251710
30	1	0	-0.526815	-2.049489	0.963989
31	34	0	-5.782156	0.232079	2.269414

32	1	0	-6.611747	-1.443385	3.616163
33	35	0	-7.808789	1.011589	4.748902

HBr

Center Number	Atomic Number	Atomic Type	Coordinates (in Å) X	Y	Z
1	35	0	-2.222641	-0.015501	0.010800
2	1	0	-0.832726	-0.301143	0.233843

TS2′

Center Number	Atomic Number	Atomic Type	Coordinates (in Å) X	Y	Z
1	6	0	1.560354	1.323923	-0.101337
2	6	0	0.475042	0.692916	0.615750
3	6	0	0.507847	-0.750414	0.768551
4	6	0	1.639197	-1.481114	0.357958
5	6	0	2.669480	-0.826834	-0.280011
6	6	0	2.628626	0.582357	-0.514964
7	1	0	1.492089	2.392527	-0.270398
8	1	0	-0.246516	-1.202999	1.398837
9	1	0	1.683100	-2.551047	0.527603
10	1	0	3.539709	-1.385925	-0.610014
11	1	0	3.458631	1.052584	-1.032397
12	8	0	-0.527398	1.344740	1.019297
13	7	0	-1.905236	1.143046	-0.689453
14	6	0	-1.908441	-0.180551	-0.532101
15	6	0	-3.004914	-0.911787	0.219182
16	1	0	-2.776473	-0.886174	1.291834
17	1	0	-3.982866	-0.444307	0.076483
18	1	0	-3.049959	-1.955074	-0.098243
19	8	0	-0.886047	-0.837716	-0.917620
20	1	0	-2.736581	1.540707	-0.240469

INT5

Center Number	Atomic Number	Atomic Type	Coordinates (in Å) X	Y	Z
1	6	0	2.128557	1.207109	-0.051631
2	6	0	0.681676	0.986780	-0.081226
3	6	0	0.200198	-0.435529	0.271449
4	6	0	1.222546	-1.516962	0.101066

5	6	0	2.530895	-1.224856	0.026021
6	6	0	2.984759	0.158644	-0.016564
7	1	0	2.472433	2.229253	-0.168681
8	1	0	0.005710	-0.379846	1.358583
9	1	0	0.851621	-2.536765	0.101979
10	1	0	3.272298	-2.014655	-0.031635
11	1	0	4.055043	0.339996	-0.064940
12	8	0	-0.129057	1.872649	-0.318139
13	7	0	-3.089596	-0.430112	-1.005892
14	6	0	-2.199498	-0.283390	-0.112781
15	6	0	-2.370369	0.310696	1.263827
16	1	0	-1.752082	1.203590	1.379067
17	1	0	-3.413427	0.592312	1.412574
18	1	0	-2.097464	-0.410948	2.041430
19	8	0	-0.969426	-0.801366	-0.438460
20	1	0	-3.979210	-0.071631	-0.660016

TS3′

Center Number	Atomic Number	Atomic Type	Coordinates (in Å) X	Y	Z
1	6	0	-1.904061	0.581286	-0.729108
2	6	0	-1.687860	-0.685005	-0.011497
3	6	0	-0.597952	-0.682896	1.022479
4	6	0	-0.125875	0.528102	1.536461
5	6	0	-0.224584	1.631668	0.708533
6	6	0	-1.185782	1.667899	-0.393497
7	1	0	-2.650582	0.586964	-1.515140
8	1	0	-0.486582	-1.612955	1.566793
9	1	0	0.540135	0.540397	2.392420
10	1	0	0.249846	2.565695	0.989428
11	1	0	-1.304317	2.604051	-0.928699
12	8	0	-2.350692	-1.692882	-0.219560
13	7	0	1.438733	0.994645	-0.810789
14	6	0	1.676843	-0.252366	-0.470432
15	6	0	3.029547	-0.740535	0.004905
16	1	0	3.214166	-1.752922	-0.361937
17	1	0	3.844450	-0.084558	-0.312673
18	1	0	3.021576	-0.782990	1.101784
19	8	0	0.715883	-1.118810	-0.427091
20	1	0	2.281491	1.558531	-0.676773
